# Enantioselective Organocatalytic Synthesis of Bicyclic Resorcinols *via* an Intramolecular Friedel−Crafts‐Type 1,4‐Addition: Access to Cannabidiol Analogues

**DOI:** 10.1002/adsc.202100647

**Published:** 2021-07-12

**Authors:** Laura A. Bryant, Kenneth Shankland, Hannah E. Straker, Callum D. Johnston, Nicholas R. Lees, Alexander J. A. Cobb

**Affiliations:** ^1^ Department of Chemistry King's College London 7 Trinity Street London SE1 1DB UK; ^2^ School of Chemistry Food and Pharmacy (SCFP) University of Reading Whiteknights Reading, Berks RG6 6AD UK; ^3^ GW Pharmaceuticals Kent Science Park Sittingbourne Kent ME9 8AG UK

**Keywords:** organocatalysis, resorcinols, Michael addition, cannabinoids

## Abstract

The organocatalytic transformation of resorcinols is extremely rare. In this article, we report a highly enantioselective, organocatalytic intramolecular cyclization of these systems by a Friedel–Crafts‐type 1,4‐addition using a Jørgensen‐Hayashi‐like organocatalyst with a large silyl protecting group, and show that heat improves reaction yield with virtually no detriment to enantioselectivity. A variety of bicyclic resorcinols were obtained with excellent enantioselectivities (up to 94%). To show the utility of these constructs, and as part of a wider project involving the synthesis of cannabinoid‐like compounds, the resorcinol formed was used to generate both ‘normal’ and ‘abnormal’ cannabidiol (CBD) derivatives which were shown to have anticonvulsant activity.

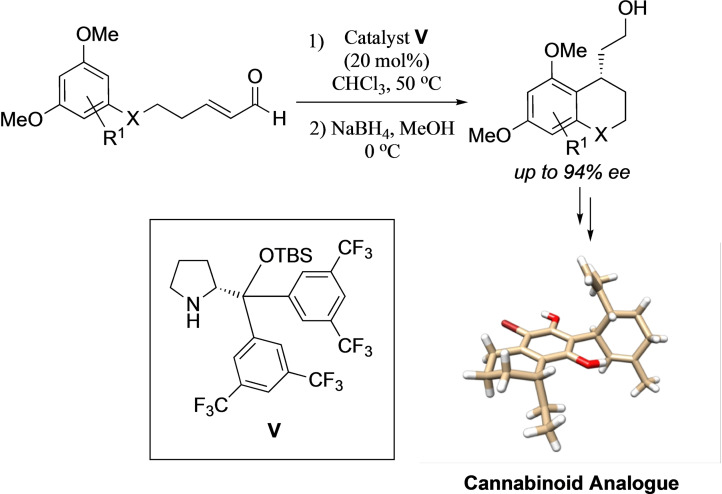

## Introduction

**Figure 1:**
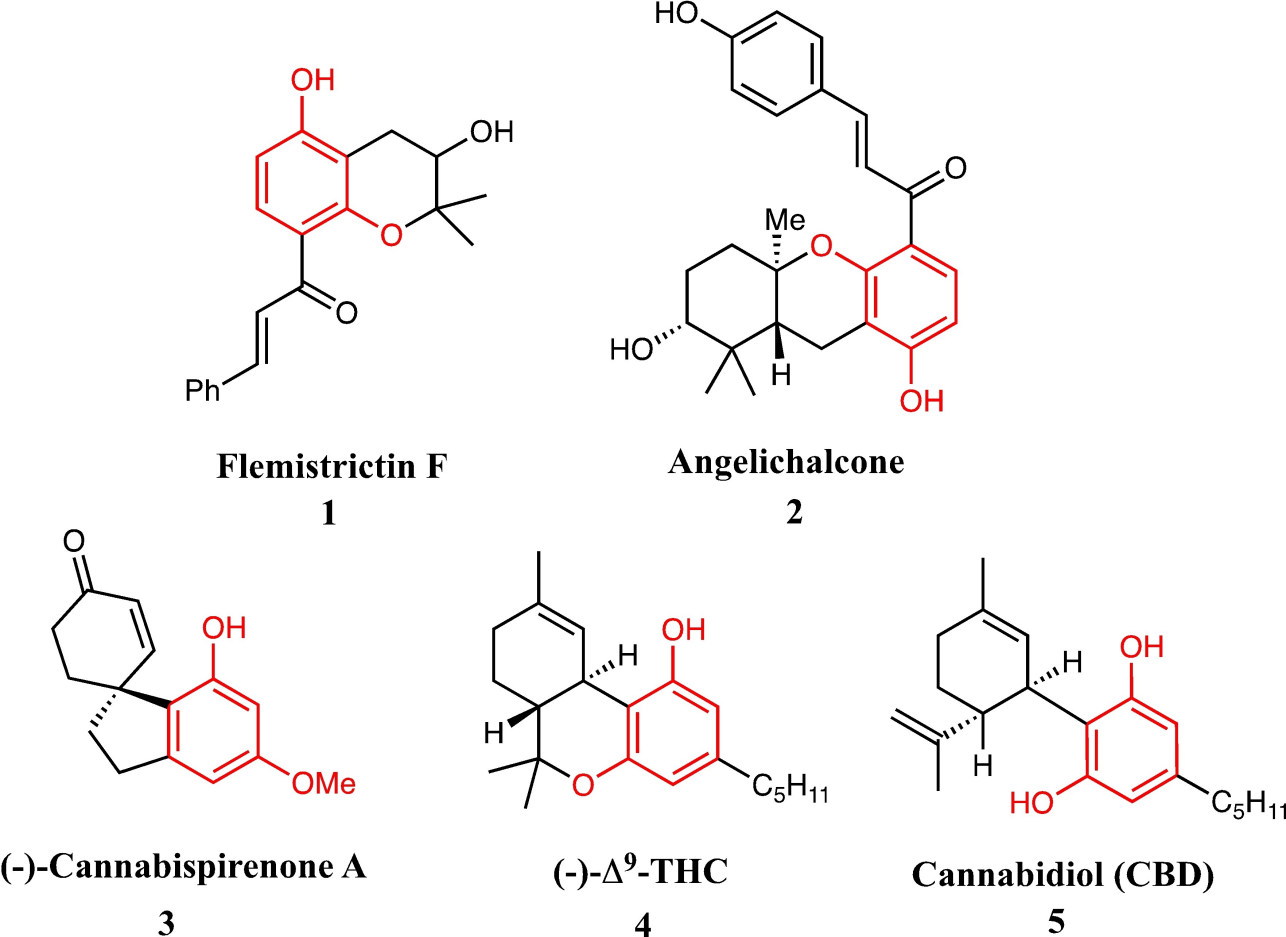
Typical natural products containing the resorcinol unit.

The resorcinol unit is a constituent of many natural products, in particular the flavanoids, isoflavanoids, and stilbenes (e. g. flemistrictin F **1**, and angelichalcone **2**, Figure 1), as well as most of the cannabinoids (e. g. **3**–**5**).^[1]^ The latter class, which originate from *Cannabis sativa L*.,^[2]^ has received a great deal of attention, not just because of the recreational uses of the main psychoactive component, Δ^9^‐THC **4**, but also due to the incredible medicinal properties of cannabidiol (CBD) **5** which in recent years has come to some prominence.^[3]^ This has been demonstrated with the recent FDA approval in June 2018 of Epidiolex®, an oral CBD solution indicated for the treatment of Lennox‐Gastaut syndrome or Dravet syndrome – two difficult‐to‐treat forms of childhood onset epilepsy.^[4]^ The success of this compound has driven us to find new ways of accessing analogues with improved pharmacological profiles,^[5]^ with our current focus being on modifications to the alkyl chain in CBD.

**Scheme 1:**
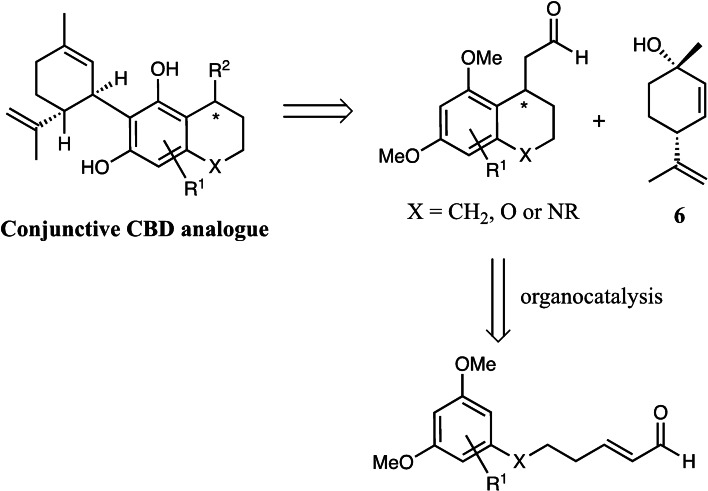
Concept: Intramolecular asymmetric organocatalytic Friedel−Crafts alkylation towards the generation of new CBD derivatives.

In that respect, we believed we could exploit the nucleophilic properties of the resorcinol unit to facilitate an intramolecular Friedel−Crafts‐type reaction so as to generate conjunctive analogues of CBD. We expected to achieve this *via* intramolecular 1,4‐conjugate addition, ultimately leading to an S_N_2’ reaction with commercially available terpene **6** (Scheme 1).^[6]^ However, the organocatalytic transformation of resorcinols in particular is a challenging and rare feat. To the best of our knowledge, only a few examples capable of exploiting this motif in such a way exist, including Yoshida and Takao's intramolecular Friedel−Crafts‐type construction of spiroindane derivatives,^[6b]^ and Nicolaou's intramolecular α‐arylation of aldehydes using organo‐SOMO catalysis.[^6c^, ^7^]

## Results and Discussion

In order to test our hypothesis, we accessed (*E*)‐6‐(3,5‐dimethoxyphenyl)hex‐2‐enal **7 a** in a 4‐step synthesis from 3,5‐dimethoxybenzyl bromide (see supporting information). A variety of catalysts were screened (Table 1) including primary amine **I** (Entry 1), a bifunctional thiourea **II** (Entry 2), – a system with which we have much experience, a selection of chiral phosphoric acids **III** (Entry 3) and a range of secondary amines **IV**–**X** (Entries 4–10). Interestingly, the cinchona alkaloid derived primary amine **I**, used by Takao and co‐workers in the only other successful organocatalytic resorcinol derived Friedel−Crafts process known to date,^[6b]^ gave very poor selectivity (entry 9). Pleasingly, however the variously silylated Jørgensen‐Hayashi type catalysts^[8]^ did give the desired cyclized product. Although the unoptimized yields for these systems were low, we found that the selectivities were very encouraging. As described by Hayashi, Seebach and co‐workers, the bulkier protecting group led to higher selectivity in such 1,4‐additions,^[9]^ although interestingly the non‐trifluoromethyl system **VI** and **VIII** did not work at all (Entry 3). In contrast, the MacMillan catalysts **IX** and **X** gave better yields but poorer enantioselectivities.

**Table 1:** Catalyst screening for the Friedel−Crafts‐type 1,4‐addition.

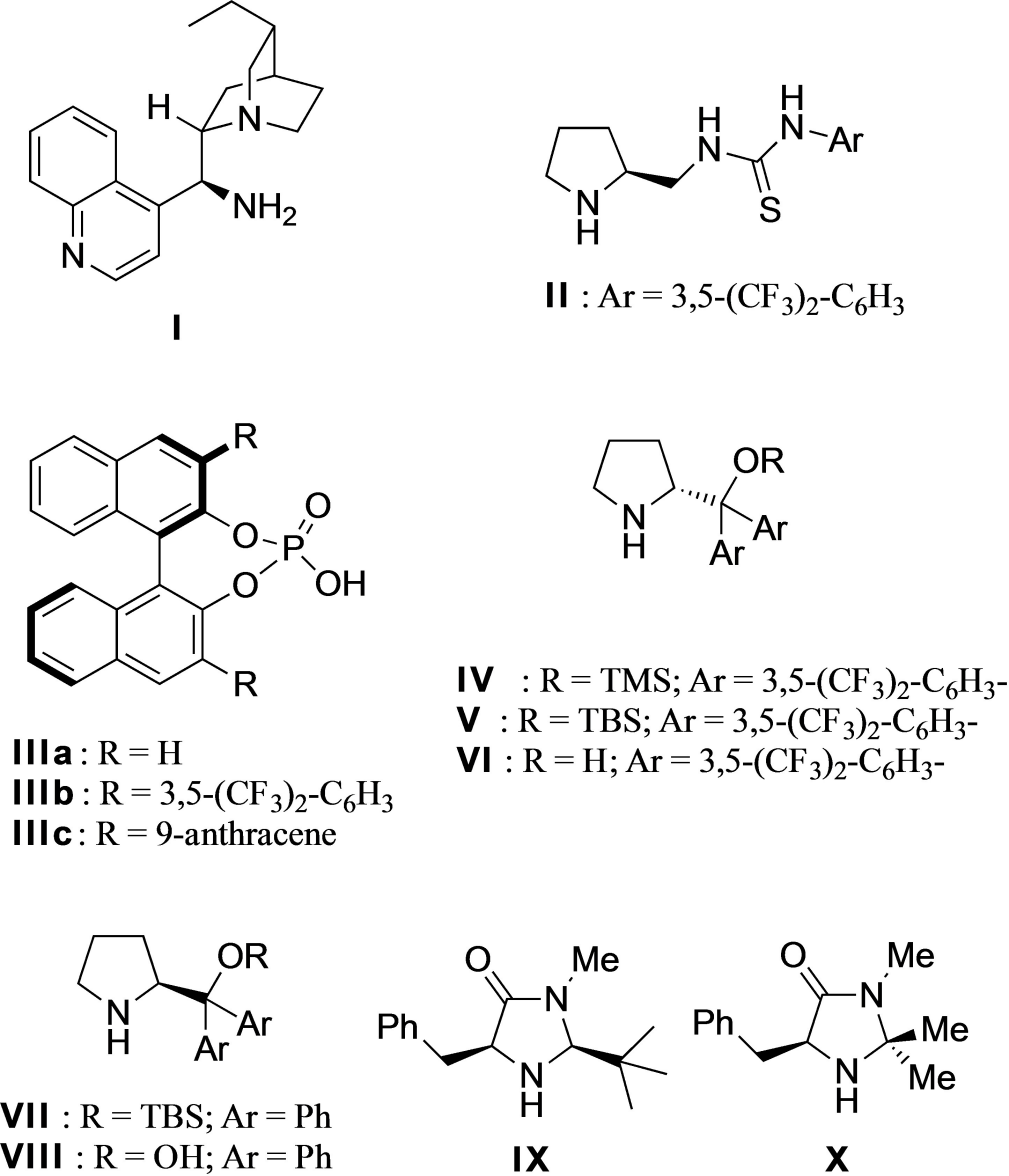				
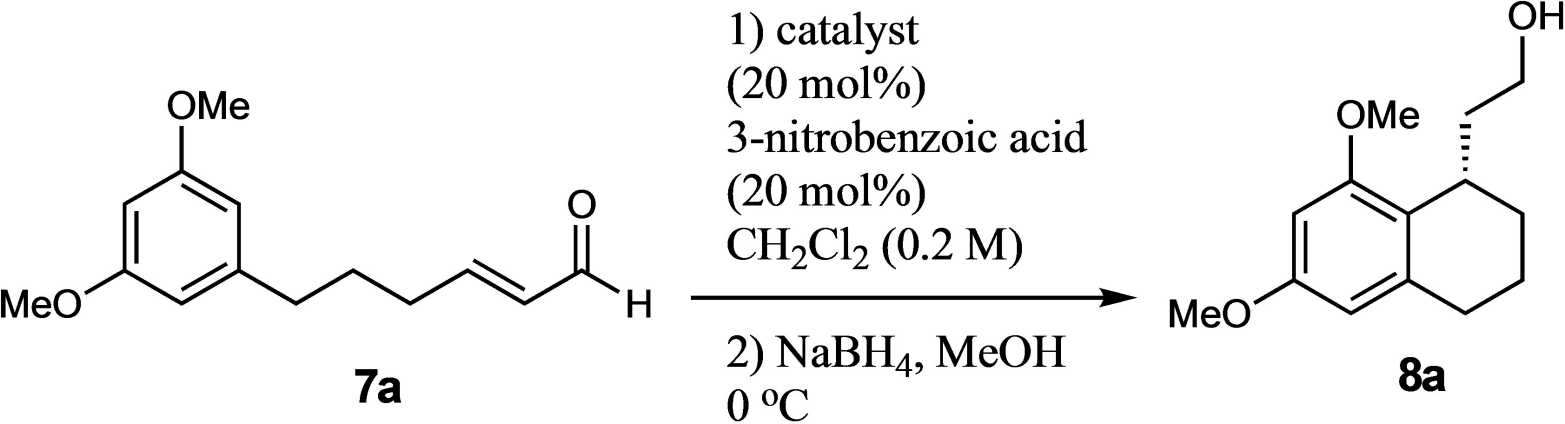				
Entry	Catalyst	Time, d^[a]^	Yield^[b]^	ee (%)^[c,d]^
1	**I**	1	69	9
2	**II**	nr	–	–
3	**IIIa‐c**	nr	–	–
4	**IV**	1	15	72
5	**V**	1	29	81
6	**VI**	nr	–	–
7	**VII**	nr	–	–
8	**VIII**	nr	–	–
9	**IX**	7	68	−68
10	**X**	1	58	−43
11^e^	**V**	7	26	92

^[a]^ Complete consumption of starting material as indicated by NMR. ^[b]^ Isolated yield over two steps. ^[c]^ Determined by chiral HPLC using a Chiralcel OD column (see supporting information). ^[d]^ Absolute structure was determined by X‐ray crystallography of the corresponding (1S)‐camphorsulfonyl derivative (compound **8 aa** in supporting information). Other compounds assigned by analogy ^[e]^ Performed with no co‐catalyst.

We also found that removal of the co‐catalyst led to much improved selectivity (entry 11). This was confirmed insomuch that a screen of additives including acid co‐catalysts and bases did not significantly improve the reaction (see supporting information for complete study), leading us to conclude that such additives might be catalyzing the non‐enantioselective background process.

In view of this, and because of its superior selectivity, we decided our best option was to optimize the yield of the conditions described in entry 10. To begin with, we performed a solvent screen (Table 2) which showed that chlorinated solvents worked best with our reaction, with chloroform being optimal and giving improved yield and further improved enantioselectivity (96%, entry 2). Fascinatingly, in other solvents, both of these variables suffered ‐ with toluene, THF and ether giving no reactivity whatsoever (entries 4 and 5). Pleasingly however, modulation of concentration (Table 2, entries 7–9) showed that yield could be further improved with no detriment to the excellent enantioselectivity, whilst at the same time improving reaction time. Our last attempt to improve the yield was to vary the temperature (entries 10–11), where intriguingly not only did the yield and reaction rate improve with increased temperature, but the enantioselectivity remained the same, even at the optimal conditions of 50 °C (entry 11).

**Table 2:** Further optimization for the Friedel−Crafts‐type 1,4‐addition.

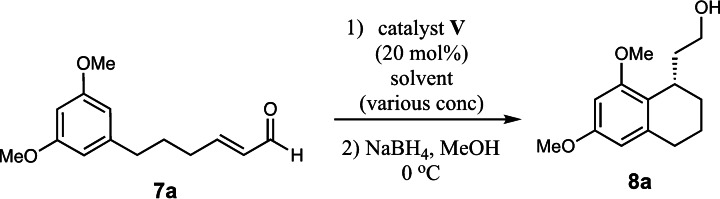					
Entry	Solvent (conc, M)	T, °C	t	Yield: (%)^[b]^	ee (%)^[c]^
1	CH_2_Cl_2_ (0.2)	rt	7 d	26	92
2	CH_2_Cl_2_ (0.2)	rt	6 d	35	96
3	MeOH (0.2)	rt	2 d	38	47
4	THF (0.2)	rt	nr	–	–
5	Et_2_O (0.2)	rt	nr	–	–
6	MeCN (0.2)	rt	6 d	6	69
7	CHCl_3_ (0.05)	rt	2 d	31	86
8	CHCl_3_ (0.5)	rt	3 d	48	96
9	CHCl_3_ (1.0)	rt	2 d	31	95
10	CHCl_3_ (0.5)	40	2 d	50	94
11	CHCl_3_ (0.5)	50	7 h	68	94

^[a]^ Complete consumption of starting material as indicated by NMR. ^[b]^ Isolated yield over two steps. ^[c]^ Determined by chiral HPLC using a Chiralcel OD column (see supporting information).

To expand the scope of this reaction, we explored the use of various substrates (Table 3). Compounds varied from the nature of the saturated ring to the substitution of the resorcinol. Heterocyclic compounds worked well, including oxygen and various *N*‐substituted systems. All gave excellent enantioselectivities, including **8 c** and **8 d**, which can open the door to constrained δ‐amino acids which are of potential use within foldamer systems.^[10]^ Interestingly, catechol **8 k** whilst suffering from poorer conversion gave a reasonable enantioselectivity. Substrates containing electron‐withdrawing substituents, unfortunately, failed to convert (see supporting information). However, post‐cyclization modification to introduce these is possible as demonstrated in compound **14** discussed *vide infra*.

**Table 3:** Scope of the Friedel−Crafts‐type 1,4‐addition.^[a,b]^

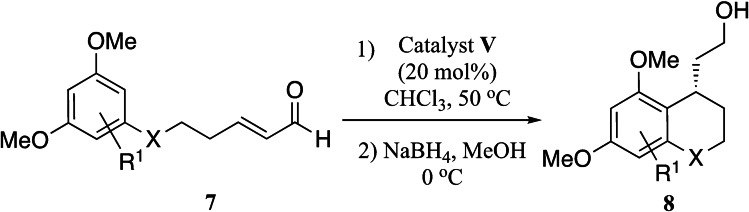		
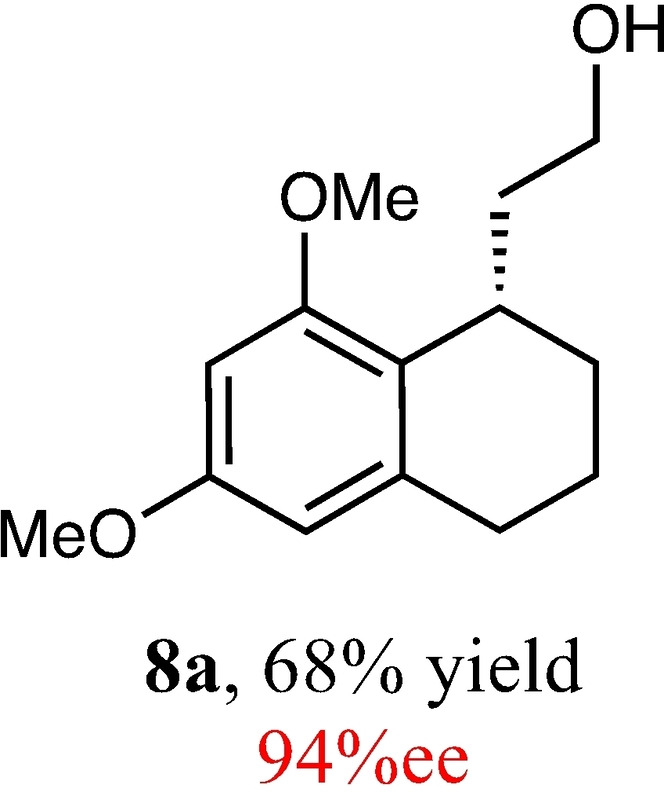	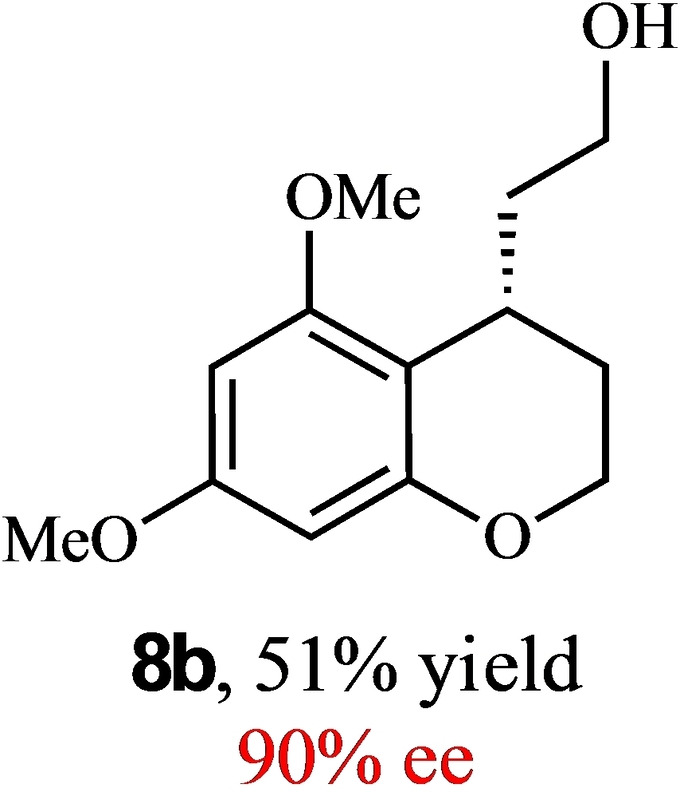	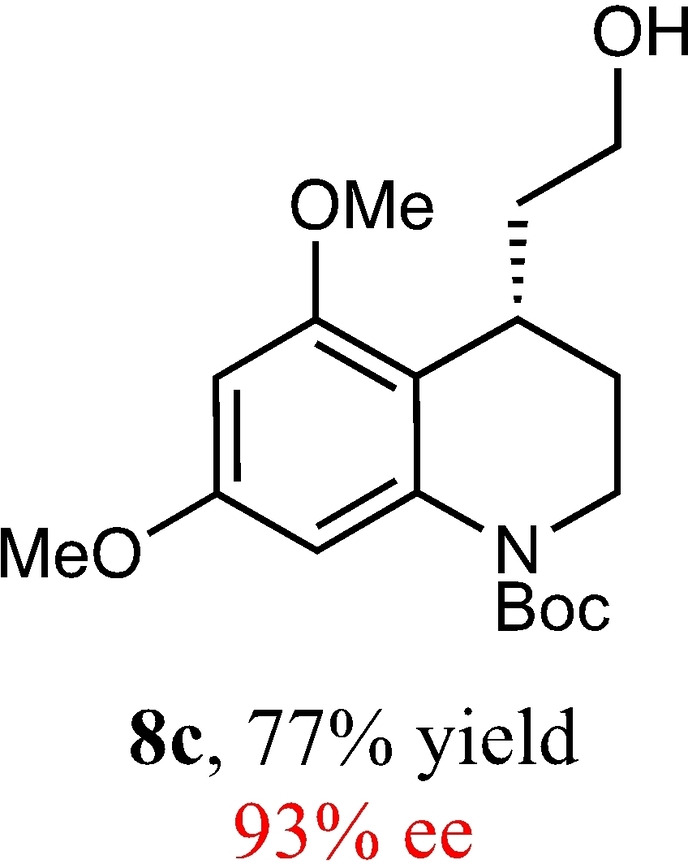
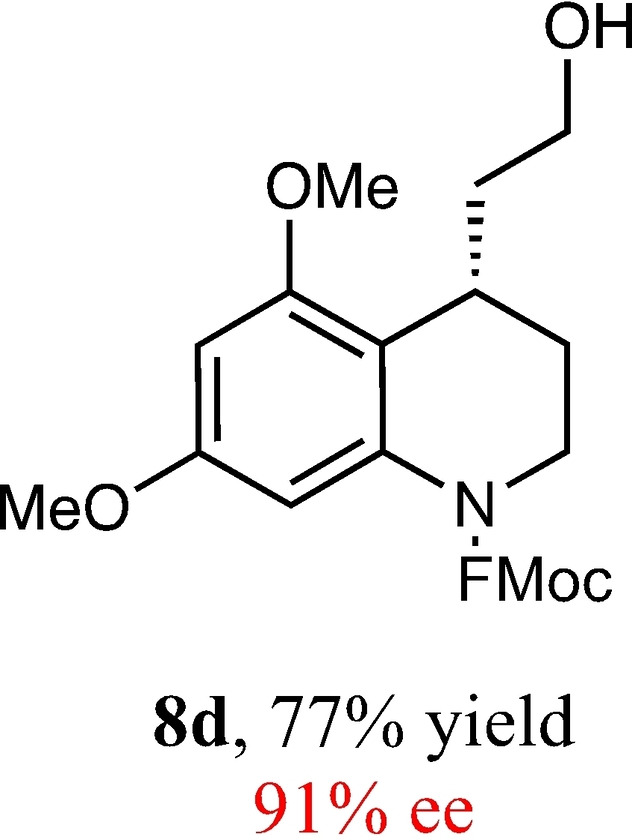	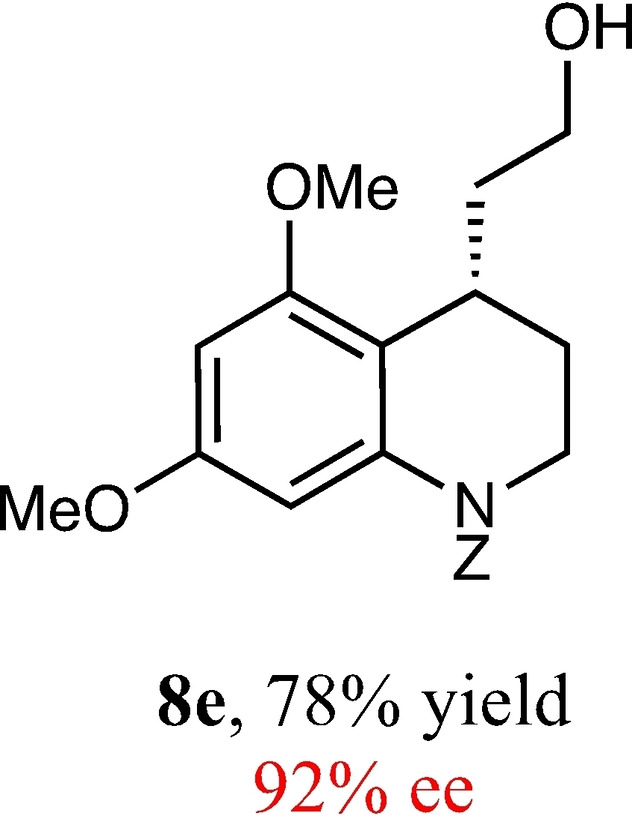	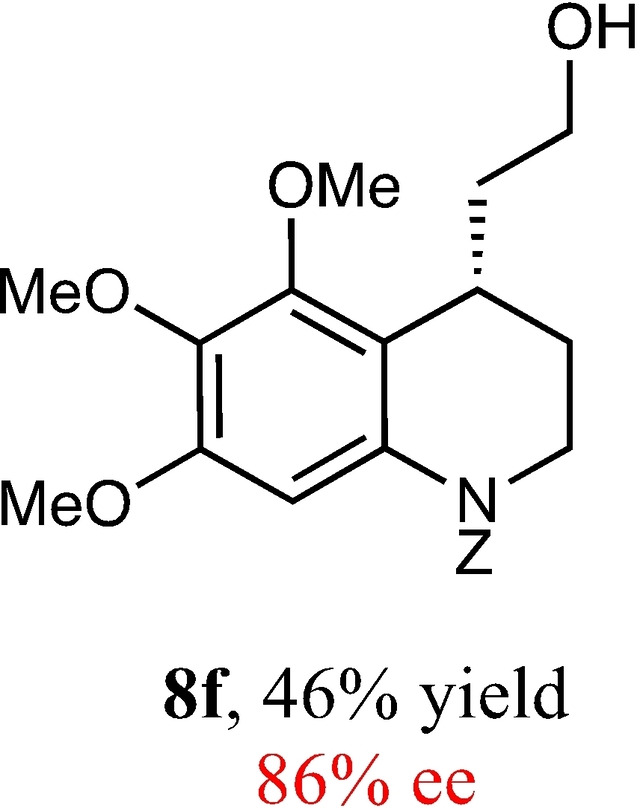
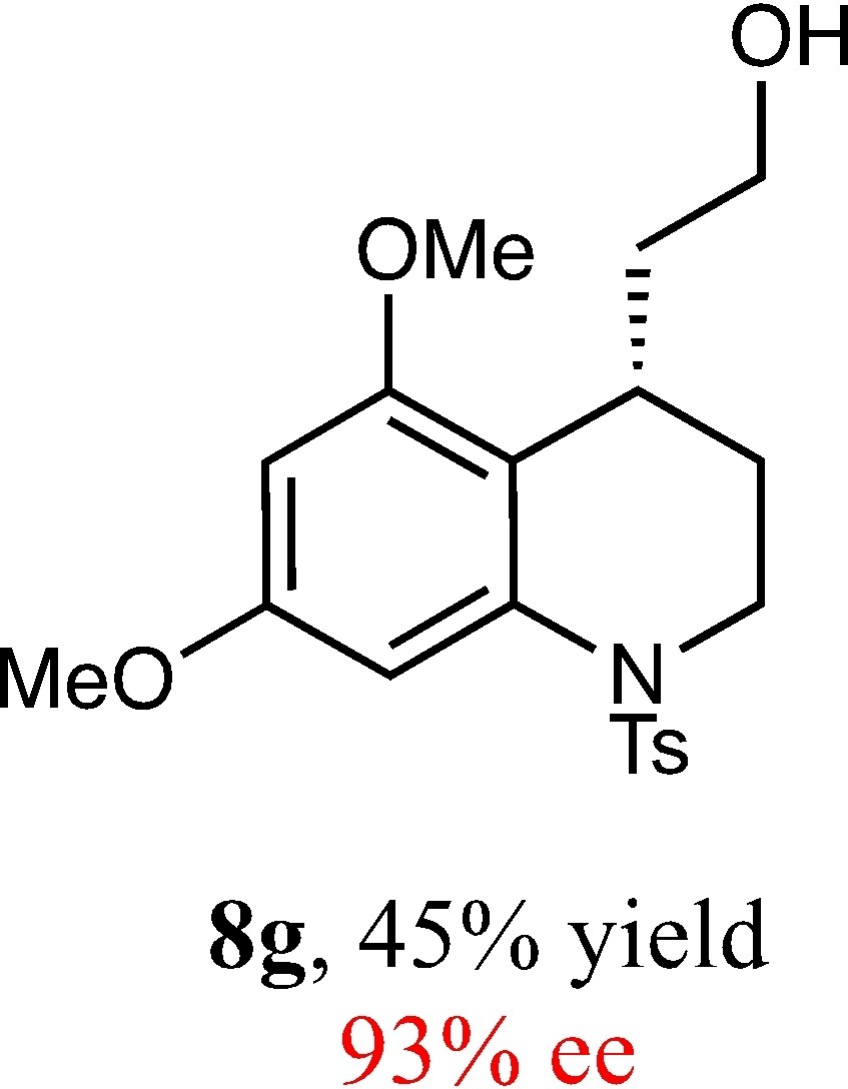	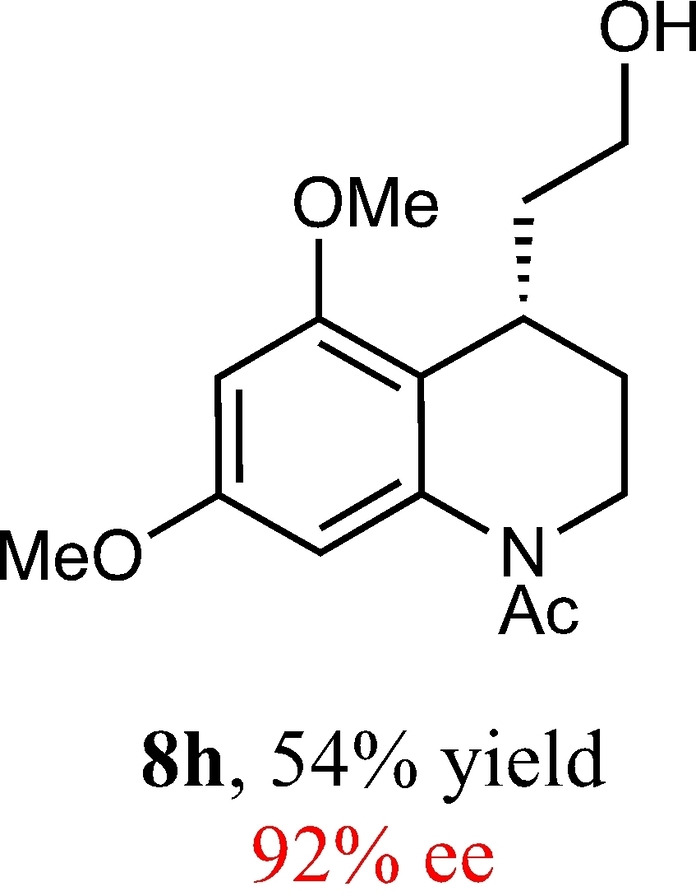	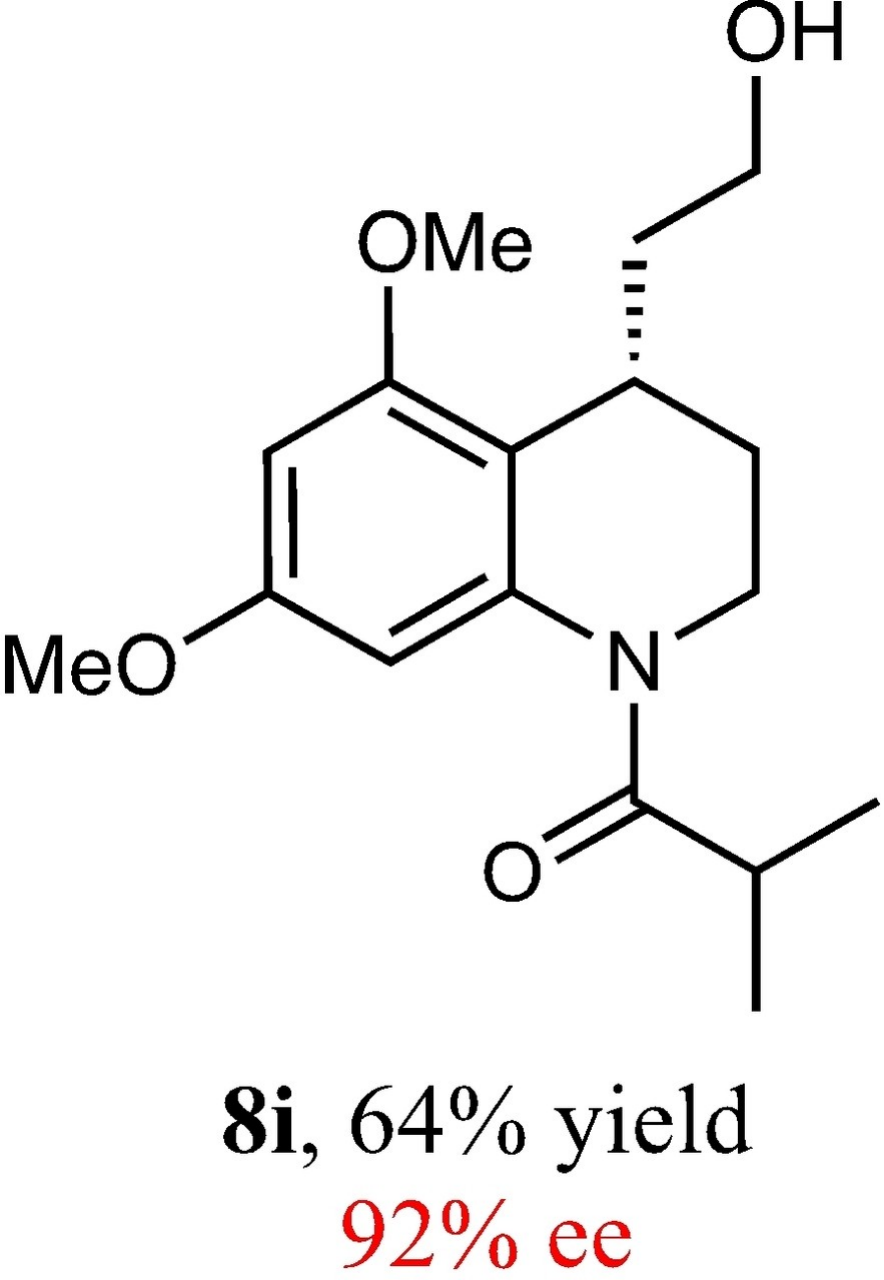
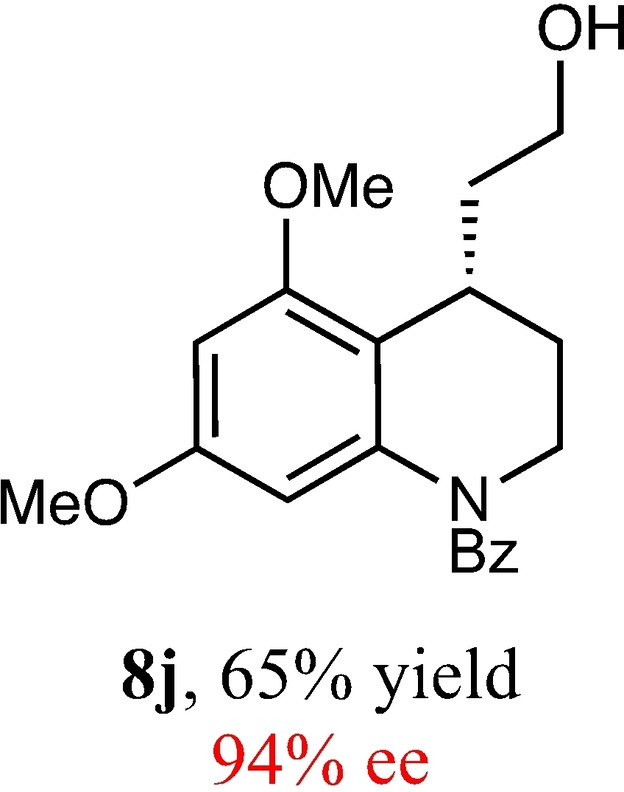	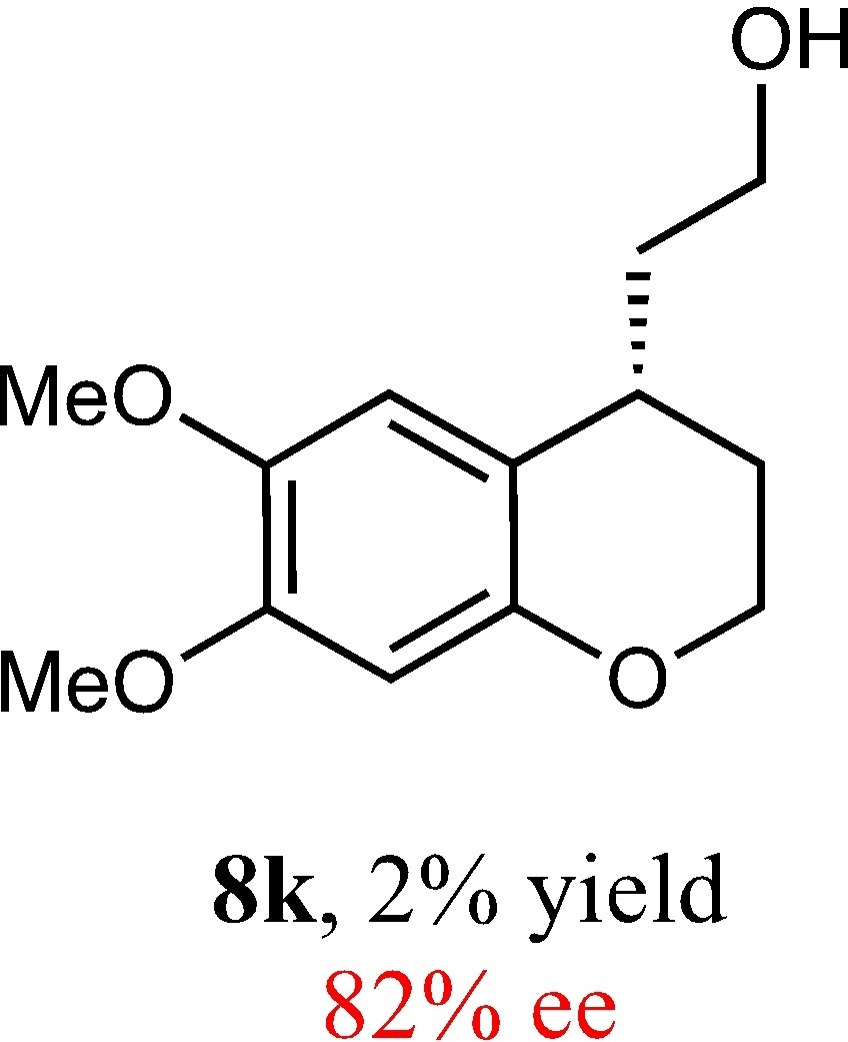

^[a]^ Isolated yield over two synthetic steps. ^[b]^ Determined by chiral HPLC (see Supporting Information).

**Figure 2:**
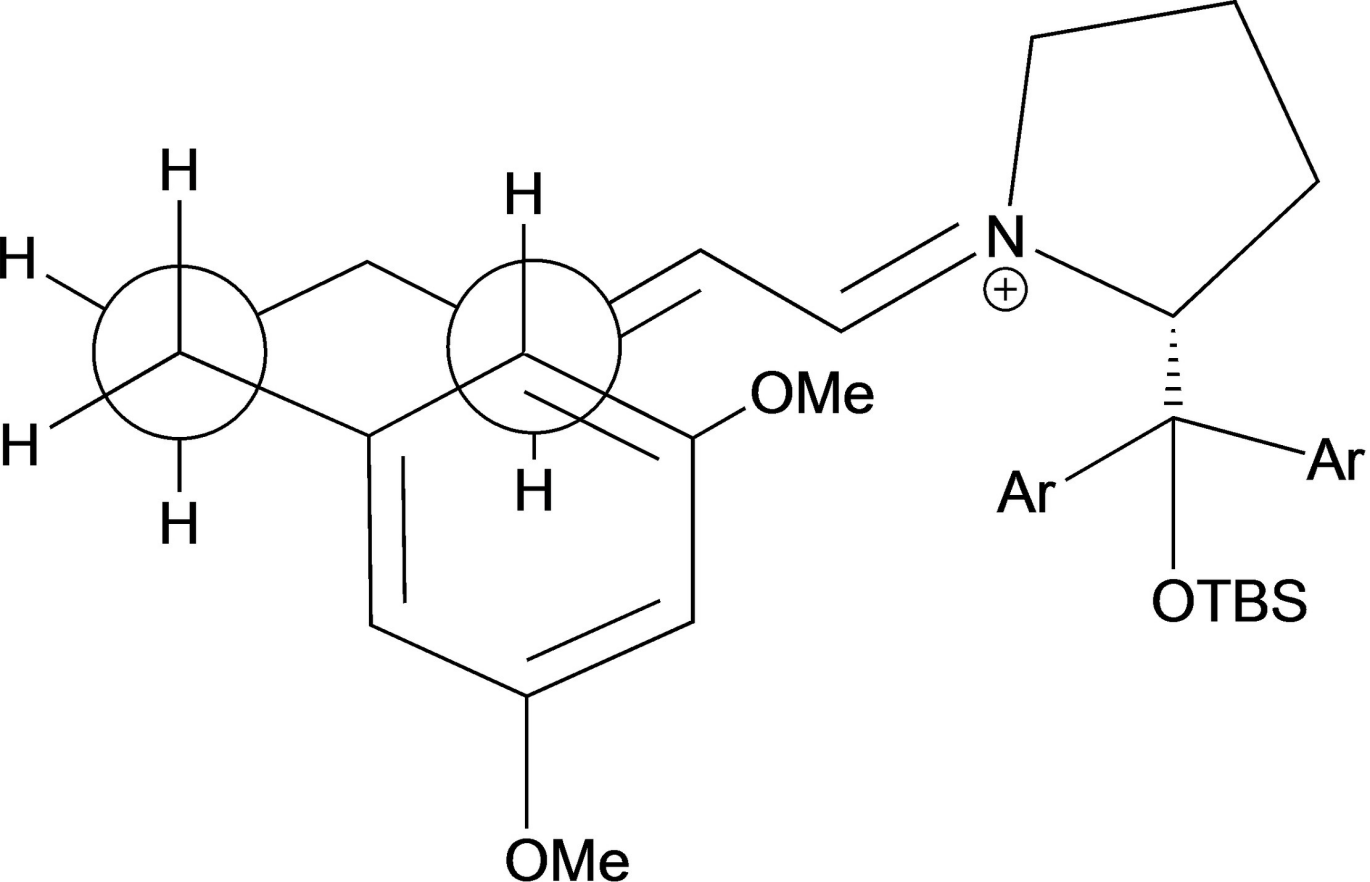
Proposed transition state for the Friedel−Crafts type 1,4‐addition. The diarylsilylether substituent blocks one face of the electrophilic centre.

Based on the observed stereochemical outcome (determined by X‐ray crystallographic analysis of the corresponding camphorsulfonyl system of compound **8 a** – see supporting information), we propose the transition state shown in figure 2, whereby the bulky silyl group of the expected *E*‐iminium ion blocks the approach of the resorcinol nucleophile from the *re*‐face. Cyclization *via* S_N_Ar thus leads to the (*S*)‐tetrahydronaphthalene system reported.

**Scheme 2:**
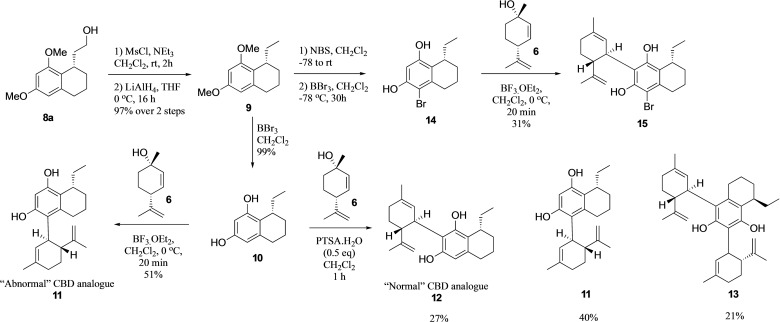
Synthesis of ‘normal’ and ‘abnormal’ isomers of cannabidiol.

We then demonstrated the utility of our resorcinol systems by using the Friedel–Crafts type 1,4‐addition in the synthesis of a CBD derivative, one of the major cannabinoids found in cannabis both in its ‘normal’ and ‘abnormal’ forms (Scheme 2). The medicinal utility of cannabinoids has led to the development of multiple cannabinoid‐derived medications such as Nabilone and Sativex.^[11]^ Cannabinoid analogues have also attracted widespread medical attention because of their interesting pharmacological properties including analgesic, antiemetic and as an appetite stimulant.[^12^, ^13^, ^14^] Since the discovery and characterization of the CB1 and CB2 receptors and the human endocannabinoid system in the early 90s, the pursuit for small molecules capable of modulating these systems has accelerated astonishingly.[^15^, ^16^, ^17^] Interestingly, although it is known that CBD itself does not interact with this system, its clearly beneficial pharmacological properties have in turn established a need for creating new, useful and easily accessible routes to enantiopure unnatural analogues.

**Figure 3:**
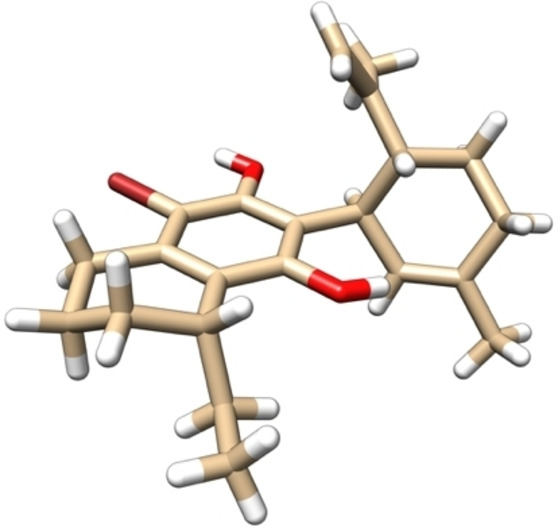
X‐ray crystal structure of compound **15** (CCDC no: 1881707).

In our first synthesis, removal of the primary alcohol of **8 a** by simple mesylation and treatment with lithium aluminium hydride gave deoxygenated system **9** in 97% overall yield. Subsequent *O*‐demethylation with boron tribromide at 0 °C afforded deprotected chiral resorcinol **10** in 99% yield. This was then reacted with *cis*‐isolimonenol **6** in either one of two conditions. The first was in the presence of boron trifluoride diethyl etherate ‐ conditions first used by Petrzilka, Haefliger and Sikemeier^[18]^ to access abnormal CBD derivative **11**. The second set of conditions, however, allowed access to the normal CBD analogue, through the use of a substoichiometric quantity of PTSA. Not only did we obtain the desired target **12**, but also the abnormal derivative **11** and a small quantity of what is speculated to be the bis‐limonene system **13** identified by LC‐MS. Exclusive access to the ‘normal’ system, however, was achieved through bromination of chiral bicyclic system **9**, to give us the arylbromide system **14** upon demethylation. Coupling with the same terpene gave the ‘normal’ regioisomer of the bromo‐CBD compound **15** in 31% yield. The structure and absolute configuration of this was also unambiguously determined by X‐ray crystallographic analysis (using a molybdenum X‐ray source, Figure 3) owing to the known configuration of the limonene (1*S*,4*R*), and further confirmed the previously assigned absolute stereochemistry. Additionally, not only did this system allow us to obtain more crystalline material for XRD, but it also has two further clear advantages – first of course, that only the ‘normal’ regioisomer is obtained, and second that the aryl bromide can be derivatized to further systems *via* aryl coupling methodologies.

## Conclusion

In conclusion, we have developed a highly enantioselective organocatalytic method for the synthesis of bicyclic resorcinols. Such transformations are rare, but we have exploited the nucleophilic character of the resorcinol towards an organocatalytic intramolecular Friedel−Crafts type 1,4‐addition. The key features of this reaction are that it requires a catalyst with a bulky silyl protecting group and can tolerate high temperatures. Furthermore, we have applied our reaction to the synthesis of cannabidiol analogues, a class of compound our group has had a long‐term interest in owing to their biological character and with the aim of developing new therapies towards the treatment of epilepsy and other conditions. The cannabinoid compounds within this report have been shown to have promising anticonvulsant activity and this will be the subject of a future communication elsewhere.

## Experimental Section

**General procedure for the organocatalytic cyclization of resorcinols**. To a solution of (*R*)‐α,α‐Bis[3,5‐bis(trifluoromethyl)phenyl]‐2‐pyrrolidinemethanol *tert*‐butyldimethylsilyl ether (0.2 equiv.) in CHCl_3_ (0.5 M) in a sealed tube was added the α,β‐unsaturated aldehyde (1 equiv.). The mixture was heated to 50 °C and stirred until consumption of starting which was monitored by NMR. Once the reaction was complete, the solution was cooled to 0 °C and reduced using NaBH_4_ (1.5 equiv.) and MeOH (0.2 M). After 20 min the reaction was quenched with saturated NH_4_Cl and distilled water. The solution was diluted with EtOAc and extracted three times. The combined organic layers were washed with brine and dried over MgSO_4_. The crude mixture was purified using silica gel on column chromatography.

### (*S*)‐2‐(6,8‐Dimethoxy‐1,2,3,4‐Tetrahydronaphthalen‐1‐yl)Ethan‐1‐ol (8 a)

Yield: 1.19 g, 68% (yellow oil). Purified using column conditions: Hex/EtOAc, 3:1. ^1^H NMR (400 MHz, CDCl_3_) δ 6.30 (d, J=2.31, 1H, ArH), 6.24 (d, J=2.25, 1H, ArH), 3.81 (s, 3H, OMe), 3.77 (s, 3H, OMe), 3.73–3.62 (m, 2H, CH_2_), 3.11–3.08 (m, 1H, CH), 2.80–2.66 (m, 2H, CH_2_), 1.88–1.63 (m, 7H, 3×CH_2_ and OH); ^13^C NMR (101 MHz, CDCl_3_) δ 158.45 (Ar), 158.00 (Ar), 138.75 (Ar), 122.28 (Ar), 104.88 (Ar), 96.33 (Ar), 61.81 (CH_2_OH), 55.52 (OMe), 55.38 (OMe), 38.61 (CH_2_CH_2_OH), 29.70 (Cy), 27.53 (Cy), 27.40 (Cy), 18.14 (Cy). [α]_D_^20^+28.0 (c 0.06, CH_3_OH) IR (diamond) υ 3357, 3285, 2992, 2939, 2916, 2865, 2837, 1605, 1591,1067, 822 cm^−1^; HRMS (EI) Exact mass calculated for C_14_H_20_O_3_ [M+H]^+^ 237.1491; Found 237.1490; HPLC analysis: Daicel Chiralpak OD, hexane/iso‐propanol=99:1, flow rate=0.8 mL/min, λ=210 nm

### (*R*)‐2‐(5,7‐Dimethoxychroman‐4‐yl)Ethan‐1‐ol (8 b)

Yield: 0.073 g, 51% (orange solid). Purified using column conditions: Hex/EtOAc, 65:35. ^1^H NMR (400 MHz, CDCl_3_) δ 6.06 (d, 1H, J=2.4 Hz, ArH), 6.03 (d, 1H, J=2.4 Hz, ArH), 4.27–4.22 (m, 2H, CH_2_), 4.17–4.11 (m, 1H, CHH), 3.80 (s, 3H, OMe), 3.75–3.66 (m, 5H, OMe and CH_2_OH), 3.10–3.05 (m, 1H, CH), 2.04–1.86 (m, 3H, 2×CH and OH), 1.80 (qd, 1H, J=14.0 and 2.3 Hz, CH), 1.75–1.67 (m, 1H, CH), 1.60 (s, 1H, OH) ppm; ^13^C NMR (101 MHz, CDCl_3_) δ 159.60 (Ar), 158.46 (Ar), 155.55 (Ar), 107.15 (Ar), 93.77 (Ar), 91.42 (Ar), 62.29 (Cy), 61.11 (CH_2_OH), 55.71 (OMe), 55.42 (OMe), 38.90 (CH_2_CH_2_OH), 26.72 (Cy), 24.30 (Cy). [α]_D_^20^+50.5 (c 0.2, CH_2_Cl_2_). m.p: 82.1–83.1 °C. HRMS (ES‐Tof) Exact mass calculated for C_13_H_18_O_4_ [M+H]^+^ 239.1283, Found 239.1272 IR (Diamond) υ 3387, 3303, 2982, 2958, 1269, 1612, 1588, 1051, 818, 810, 789 cm^−1^; HRMS (ES‐Tof) Exact mass calculated for C_13_H_18_O_4_ [M+H]^+^ 239.1283, Found 239.1272 HPLC analysis: Daicel Chiralpak OD, hexane/iso‐propanol=99:1, flow rate=1.0 mL/min, λ=210 nm

### *tert*‐Butyl (*S*)‐4‐(2‐Hydroxyethyl)‐5,7‐Dimethoxy‐3,4‐Dihydroquinoline‐1(2*H*)‐Carboxylate (8 c)

Yield: 0.17 g, 77% (pale yellow oil). Purified using column conditions: Hex/EtOAc, 75:25. ^1^H NMR (400 MHz, CDCl_3_) δ 7.01 (d, 1H, J=2.0 Hz, ArH), 6.23 (d, 1H, J=2.4 Hz, ArH), 3.81 (s, 3H, OMe), 3.79 (s, 3H, OMe), 3.73–3.60 (m, 3H, CH and CH_2_), 3.53–3.47 (m, 1H, CH), 3.32–3.30 (m, 1H, CH), 2.32 (m, 1H, OH), 1.95–1.89 (m, 1H, CH), 1.87–1.78 (m, 1H, CH), 1.73–1.67 (m, 2H, CH_2_), 1.52 (s, 9H, 3×CH_3_) ppm; ^13^C NMR (101 MHz, CDCl_3_) δ 158.37 (Ar), 156.87 (Ar), 154.10 (C=O), 139.35 (Ar), 114.65 (Ar), 101.59 (Ar), 94.25 (Ar), 80.97 (C(CH_3_)_3_), 61.17 (Cy), 55.86 (OMe), 55.53 (OMe), 42.17 (CH_2_OH), 36.13 (Cy), 28.58 (CH_3_), 28.48 (CH_2_CH_2_OH), 26.02 (Cy). [α]_D_^20^−24.0 (c 0.2, CH_2_Cl_2_) HRMS (ES‐Tof) IR (Diamond) υ 3512, 2938, 1689, 1609, 1204, 825, 744 cm^−1^ Exact mass calculated for C_18_H_27_NO_5_ [M+H]^+^ 338.1967, Found 338.1967 HPLC analysis: Daicel Chiralpak OD, hexane/iso‐propanol=96.5:3.5 flow rate=1.0 mL/min, λ=210 nm

### (9*H*‐Fluoren‐9‐yl)Methyl (*S*)‐4‐(2‐Hydroxyethyl)‐5,7‐Dimethoxy‐3,4‐Dihydroquinoline‐1(2*H*)‐Carboxylate (8 d)

Yield: 0.28 g, 69% (white solid). Purified using column conditions: Hex/EtOAc, 55:45. ^1^H NMR (400 MHz, CDCl_3_) δ 7.77 (d, 2H, J=7.6 Hz, ArH), 7.55 (t, 2H, J=6.8 Hz, ArH), 7.40 (td, 2H, J=3.3 and 7.5 Hz, ArH), 7.32–7.28 (m, 2H, ArH), 7.01 (s, 1H, ArH), 6.27 (d, 1H, J=2.4 Hz, ArH), 4.61–4.51 (m, 2H, CH_2_), 4.28 (t, 1H, J=6.7 Hz, ArH), 3.83 (s, 3H, OMe), 3.74 (s, 3H, OMe), 3.70–3.61 (m, 3H, CH and CH_2_), 3.52–3.46 (m, 1H, CH), 3.36–3.30 (m, 1H, CH), 2.27 (s, 1H, OH), 1.93–1.82 (m, 2H, CH_2_), 1.72–1.59 (m, 2H, CH_2_). ^13^C NMR (101 MHz, CDCl_3_) δ 158.62 (Ar), 156.95 (Ar), 154.93 (8‐C), 144.02 and 143.97 (2×rotamer Ar), 141.53 (Ar), 138.78 (Ar), 127.89 and 127.88 (2×rotamer Ar), 127.24 and 127.22 (2×rotamer Ar) 125.15 (2×rotamer Ar), 120.15 (Ar), 115.01 (Ar), 101.54 (Ar), 94.85 (Ar), 67.64 (OCH_2_CH), 61.13 (CH_2_OH), 55.54 (OMe), 55.55 (OMe), 47.45 (CH(Fmoc)), 42.25 (Cy), 36.11 (CH_2_CH_2_OH), 28.37 (Cy), 25.92 (Cy). [α]_D_^20^+6.5 (c 0.2, CH_2_Cl_2_) m.p: 50.5–51.0 °C. IR (Diamond) υ 3457, 3001, 2968, 2943, 2866, 2827, 1700, 1696, 1608, 1492, 1002, 726 cm^−1^ HRMS (EI) Exact mass calculated for C_28_H_29_NO_5_ [M+H]^+^ 460.2124, Found 460.2118 HPLC analysis: Daicel Chiralpak AD‐H, hexane/iso‐propanol=92.5:7.5, flow rate=1.0 mL/min, λ=210 nm

### Benzyl (*S*)‐4‐(2‐hHydroxyethyl)‐5,7‐Dimethoxy‐3,4‐Dihydroquinoline‐1(2*H*)‐Carboxylate (8 e)

Yield: 0.26 g, 78% (pale yellow oil). Purified using column conditions: Hex/EtOAc, 7:3. ^1^H NMR (400 MHz, CDCl_3_) δ 7.41–7.30 (m, 5H, ArH), 7.01 (s, 1H, ArH), 6.24 (d, 1H, J=2.3 Hz, ArH), 5.29 (d, AB system, 1H, J=12.4 9‐CH), 5.20 (d, AB system, 1H, J=12.4 Hz, 9‐CH), 3.85–3.72 (m, 5H, OMe and CH_2_), 3.68 (s, 3H, OMe), 3.65–3.57 (m, 1H, CH), 3.53–3.46 (m, 1H, CH), 3.36–3.30 (m, 1H, CH), 2.32–3.27 (m, 1H, OH), 1.98–1.92 (m, 1H, CH), 1.90–1.80 (m, 1H, CH), 1.76–1.66 (m, 2H, CH_2_) ppm; ^13^C NMR (101 MHz, CDCl_3_) δ 158.50 (Ar), 156.90 (Ar), 154.88 (C=O), 138.71 (Ar), 136.40 (Ar), 128.70 (Ar), 128.32 (Ar), 128.24 (Ar), 114.79 (Ar),101.16 (Ar), 94.82 (Ar), 67.72 (CH_2_Ph), 61.10 (CH_2_OH), 55.89 (OMe), 55.42 (OMe), 42.36 (Cy), 36.01 (CH_2_CH_2_OH), 28.27 (Cy), 25.91 (Cy). [α]_D_^20^ −21.0 (c 0.2, CH_2_Cl_2_). IR (Diamond) υ 3417, 2943, 2844, 1696, 1607, 1587, 1206, 826, 697 cm^−1^ HRMS (ES‐Tof) Exact mass calculated for C_21_H_25_NO_5_ [M+H]^+^ 372.1811, Found 372.1816 HPLC analysis: Daicel Chiralpak OD, hexane/iso‐propanol=94:6, flow rate=1.0 mL/min, λ=210 nm

### Benzyl (*S*)‐4‐(2‐Hydroxyethyl)‐5,6,7‐Trimethoxy‐3,4‐Dihydroquinoline‐1(2*H*)‐Carboxylate (8 f)

Yield: 0.16 g, 46% (dark yellow oil). Purified using column conditions: Hex/EtOAc, 55:45. ^1^H NMR (400 MHz, CDCl_3_) δ 7.41–7.31 (m, 5H, ArH), 7.20 (s, 1H, ArH), 5.32 (d, 1H, J=12.4 Hz, CH), 5.18 (d, 1H, AB system, 12.4 Hz, CH) 3.89 (s, 3H, OMe), 3.82–3.72 (m, 8H, 2×OMe and CH_2_), 3.65–3.61 (m, 1H, CH), 3.50–3.44 (m, 1H, CH), 3.31–3.26 (m, 1H, CH), 2.57 (s, 1H, OH), 2.02–1.96 (m, 1H, CH), 1.91–1.82 (m, 1H, CH), 1.74 (ddt, 1H, J=14.51, 10.8 and 14.3 Hz, CH), 1.66–1.58 (m. 1H, CH) ppm; ^13^C NMR (101 MHz, CDCl_3_) δ 154.90 (C=O), 151.43 (Ar), 150.02 (Ar), 138.02 (Ar), 136.37 (Ar), 132.96 (Ar), 128.73 (Ar),128.39 (Ar), 128.27 (Ar), 119.51 (Ar), 104.44 (Ar), 67.75 (CH_2_Ph), 61.45 (OMe), 61.04 (OMe), 60.88 (CH_2_OH), 55.96 (OMe), 42.25 (Cy), 35.91 (CH_2_CH_2_OH), 28.31 (Cy), 26.78 (Cy). [α]_D_^20^ −14.5 (c 0.2, CH_2_Cl_2_). [M+H]^+^ 402.1917, Found 402.1908 IR (Diamond) υ 3480, 3011, 2941, 2866, 2827, 1696, 1685, 1207, 833 cm^−1^ HRMS (EI) Exact mass calculated for C_22_H_27_NO_6_ HPLC analysis: Daicel Chiralpak OD, hexane/iso‐propanol=96:4, flow rate=1.0 mL/min, λ=210 nm

### (*S*)‐2‐(5,7‐Dimethoxy‐1‐Tosyl‐1,2,3,4‐Tetrahydroquinolin‐4‐yl)Ethan‐1‐ol (8 g)

Yield: 0.055 g, 45% (pale yellow oil). Purified using column conditions: Pentane/Et_2_O, 9:1. ^1^H NMR (400 MHz, CDCl_3_) δ 7.49 (d, 2H, J=8.4 Hz, ArH), 7.19 (m, 3H, ArH), 6.28 (d, J=2.3 Hz, ArH), 3.94–3.89 (m, 1H, CH), 3.83 (s, 3H, OMe), 3.78 (s, 3H, OMe), 3.54 (td, 1H, J=12.4 and 5.12 Hz, CH), 3.25–3.12 (m, 2H, CH_2_), 3.08–3.02 (m, 1H, CH), 2.42 (s, 3 H, CH_3_), 2.07 (s, 1H, OH), 1.75–1.60 (m, 2H, CH_2_), 1.27–1.22 (m, 1H, CH), 0.80 (ddt, 1H, J=13.7, 8.4 and 5.0 Hz, CH) ppm; ^13^C NMR (101 MHz, CDCl_3_) δ 158.75 (Ar), 157.27 (Ar), 143.88 (Ar), 137.69 (Ar), 135.58 (Ar), 129.62 (Ar), 127.27 (Ar), 114.82 (Ar), 101.20 (Ar), 95.61 (Ar), 60.72 (CH_2_OH), 55.87 (OMe), 55.65 (OMe), 43.44 (Cy), 36.61 (CH_2_CH_2_OH), 27.22 (Cy), 25.71 (Cy), 21.62 (ArCH_3_). [α]_D_^20^ +66.0 (c 0.2, CH_2_Cl_2_), IR (Diamond) υ 3557, 3386, 2941, 2882, 1607, 1585, 1127, 1120, 669 cm^−1^. HRMS (ES‐Tof) Exact mass calculated for C_20_H_25_ NO_5_S [M+H]+ 392.1532, Found 392.1531 HPLC analysis: Daicel Chiralpak OD, hexane/iso‐propanol=95.5:4.5, flow rate=1.0 mL/min, λ=210 nm. Amended conditions of hexane/iso‐propanol 95:5 were used for the racemic sample.

### (*S*)‐1‐(4‐(2‐Hydroxyethyl)‐5,7‐Dimethoxy‐3,4‐Dihydroquinolin‐1(2*H*)‐yl)Ethan‐1‐One (8 h)

Yield: 0.14 g, 54% (yellow oil). Purified using column conditions: Hex/EtOAc, gradient, 75:25 to 9:1. ^1^H NMR (400 MHz, CDCl3) δ 6.42–6.32 (m, 2H, ArH), 4.14–3.96 (m, 1H, CH), 3.84 (s, 3H, OMe), 2.78 (s, 3H, OMe), 3.63–3.57 (m, 1H, CH), 3.50–3.35 (m, 2H, 2×CH), 2.21 (s, 3H, CH_3_), 2.05–1.96 (m, 1H, CH), 1.93–1.81 (m, 1H, CH), 1.72 (ddt, 1H, J=14.2, 8.7 and 5.8 Hz, CH), 1.64–1.56 (m, 1H, CH). ^13^C NMR (101 MHz, CDCl_3_) δ 170.35 (C=O), 158.69 (Ar), 157.26 (Ar), 140.19 (Ar), 103.02 (Ar), 95.52 (Ar), 61.20 (CH_2_OH), 55.98 (OMe), 55.61 (OMe), 41.85 (Cy), 36.21 (CH_2_CH_2_OH), 29.11 (Cy), 26.39 (Cy), 23.54 (C(O)CH_3_). [α]_D_^20^ −105.0 (c 0.2, CH_2_Cl2). IR (Diamond) υ 3408, 2937, 2878, 2839, 1634, 1607, 1048, 827 cm^−1^ HRMS (EI) Exact mass calculated for C_15_H_21_NO_4_ [M+H]^+^ 280.1549, Found 280.1546 HPLC analysis: Daicel Chiralpak OD, hexane/iso‐propanol=92.5:7.5, flow rate=1.0 mL/min, λ=210 nm

### (*S*)‐1‐(4‐(2‐Hydroxyethyl)‐5,7‐Dimethoxy‐3,4‐Dihydroquinolin‐1(2*H*)‐yl)‐2‐Methylpropan‐1‐One (8 i)

Yield: 0.18 g, 64% (Yellow solid). Purified using column conditions: Hex/EtOAc, 2:3. ^1^H NMR (400 MHz, CDCl_3_) δ 6.34 (6.38–6.34 (m, 2H, ArH), 4.23–4.15 (m, 1H, CH), 3.85 (s, 3H, OMe), 3.79 (s, 3H, OMe), 3.61–3.56 (m, 1H, CH), 3.50–3.43 (m, 1H, CH), 3.42–3.30 (m, 2H, 2×CH), 3.17 (sept, 1H, J=6.7 Hz, CH(CH_3_)_2_), 2.24 (s, 1H, OH), 1.99–1.85 (m, 2H, CH_2_), 1.74–1.58 (m, 2H, CH_2_), 1.25 (d, 3H, J=6.6 Hz, CH_3_), 1.01 (d, 3 H, J=6.6 Hz, CH_3_) ppm; ^13^C NMR (101 MHz, CDCl_3_) δ 177.72 (C=O), 158.69 (Ar), 157.48 (Ar), 140.41 (Ar), 117.36 (Ar), 102.52 (Ar), 95.61 (Ar), 61.21 (CH_2_OH), 56.00 (OMe), 55.58 (OMe), 41.90 (Cy), 36.43 (CH_2_CH_2_OH), 31.48 (CH(CH_3_)_2_), 29.43 (Cy), 26.55 (Cy), 20.52 (CH_3_), 19.92 (CH_3_). [α]_D_^20^ −124.0 (c 0.1, MeOH), m.p: 60.2‐62.3 °C. HRMS (Es‐Tof) IR (Diamond) υ 3405, 2956, 2937, 2872, 1653, 1634, 1494, 1046, 821 cm^−1^ Exact mass calculated for C_17_H_25_NO_4_ [M+H]^+^ 308.1862, Found 308.1864 HPLC analysis: Daicel Chiralpak AD‐H, hexane/iso‐propanol=92.5:7.5, flow rate=1.0 mL/min, λ=210 nm

### (*S*)‐(4‐(2‐Hydroxyethyl)‐5,7‐Dimethoxy‐3,4‐Dihydroquinolin‐1(2*H*)‐yl)(Phenyl)Methanone (8 j)

Yield: 0.13 g, 65% (Yellow solid). Purified using column conditions: Hex/EtOAc, 65:35. ^1^H NMR (400 MHz, CDCl_3_) δ 7.30–7.18 (m, 5H, ArH), 6.12 (d, 1H, J=2.2 Hz, ArH), 5.67 (d, 1H, J=1.7 Hz, ArH), 4.08–3.94 (m. 1H, CH), 3.76–3.67 (m, 5H, 2×CH and OMe), 3.58–3.52 (m, 1H, CH), 3.42–3.36 (m, 1H, CH), 3.20 (s, 3H, OMe), 2.32 (s, 1H, OH), 2.07–2.01 (m, 1H, CH), 1.92–1.74 (m, 3H, CH and CH_2_). ^13^C NMR (101 MHz, CDCl3) δ 170.44 (C=O), 158.00 (Ar), 157.06 (Ar), 139.92 (Ar), 136.69 (Ar), 130. 19 (Ar), 128.49 (Ar), 128.30 (Ar), 115.76 (Ar), 103.58 (Ar), 95.61 (Ar), 61.39 (CH_2_OH), 55.94 (OMe), 55.23 (OMe), 42.51 (Cy), 35.48 (CH_2_CH_2_OH), 31.04 (Cy), 26.69 (Cy). [α]_D_^20^ −99.5 (c 0.2, CH_2_Cl_2_) m.p: 103–104.5 °C IR (Diamond) υ 3429, 2939, 2843, 1637, 1611, 1586, 1250, 1043, 825 cm^−1^ HRMS (Es‐Tof) Exact mass calculated for C_20_H_23_NO_4_ [M+H]+ 342.1705, HPLC analysis: Daicel Chiralpak AD‐H, hexane/iso‐propanol=9:1, flow rate=1.0 mL/min, λ=210 nm

### (*S*)‐2‐(5,6‐Dimethoxy‐1,2,3,4‐Tetrahydroquinolin‐4‐yl)Ethan‐1‐ol (8 k)

Yield: 0.005 g, 2% (brown oil). Purified using column conditions: Hex/Et_2_O/CH_2_Cl_2_, 3.5:4:2.5. ^1^H NMR (400 MHz, CDCl_3_) δ 6.64 (s, 1H, ArH), 6.37 (1H, ArH), 4.15–4.12 (m, 2H, CH_2_OAr), 3.85–3.78 (m, 8H, 2×OMe and CH_2_), 2.95 (dq, 1H, J=10.3 and 5.4 Hz, CHAr), 2.12–2.01 (m, 2H, 2×CH), 1.83–1.74 (m, 2H, 2×CH), 1.62 (1H, OH); ^13^C NMR (101 MHz, CDCl3) δ 148.7 (Ar), 148.6 (Ar), 143.2 (Ar), 116.5 (Ar), 112.2 (Ar), 100.95 (Ar), 63.5 (CH_2_OAr), 60.6 (CH_2_OH), 56.7 (OMe), 56.0 (OMe), 39.5 (CH_2_CH_2_OH), 29.9 (CHAr), 27.4 (CH_2_CH_2_OAr) ppm; [α] _D_^20^ +36.00 (c 0.1, CH_2_Cl_2_), IR (diamond) υ 3443, 2989, 2956, 2862, 2833, 1619, 1054, 1449, 1371, 1262, 1218, 1054, 1034, 855 cm^−1^ HRMS (EI) Exact mass calculated for C_13_H_18_O_4_ [M+H]^+^ 239.1283, Found 239.1293

CCDC‐1881707 contains the supplementary crystallographic data for this paper. These data can be obtained free of charge from The Cambridge Crystallographic Data Centre via https://www.ccdc.cam.ac.uk/structures/

## Supporting information

As a service to our authors and readers, this journal provides supporting information supplied by the authors. Such materials are peer reviewed and may be re‐organized for online delivery, but are not copy‐edited or typeset. Technical support issues arising from supporting information (other than missing files) should be addressed to the authors.

Supporting Information
