# Supplementary material for: Enantioselective Organocatalytic Synthesis of Bicyclic Resorcinols via an Intramolecular Friedel−Crafts‐Type 1,4‐Addition: Access to Cannabidiol Analogues
Source: Adv Synth Catal. 2021 Jul 12;363(16):4067–74. doi: 10.1002/adsc.202100647 (PMC8457227; doi:10.1002/adsc.202100647)
Supplement: Supplementary file 1 — Supporting Information [file ADSC-363-4067-s001.pdf]

Advanced Synthesis & Catalysis

Supporting Information

Enantioselective Organocatalytic Synthesis of Bicyclic Resorcinols *via* an Intramolecular Friedel–Crafts-Type 1,4-Addition: Access to Cannabidiol Analogues

Laura A. Bryant, Kenneth Shankland, Hannah E. Straker, Callum D. Johnston, Nicholas R. Lees, and Alexander J. A. Cobb*© 2021 The Authors. Advanced Synthesis & Catalysis published by Wiley-VCH GmbH. This is an open access article under the terms of the Creative Commons Attribution License, which permits use, distribution and reproduction in any medium, provided the original work is properly cited.

Supporting Information

Enantioselective Organocatalytic Synthesis of Bicyclic Resorcinols via an Intramolecular Friedel-Crafts-Type 1,4-Addition : Access to Cannabidiol Analogues

Laura A. Bryant,[a] Kenneth Shankland,[b] Hannah E. Straker,[c] Callum D. Johnston,[a] Nicholas R. Lees,[a] and Alexander J. A. Cobb,[a*]

Department of Chemistry, King's College London, 7 Trinity Street, London SE1 1DB, UK. andre.cobb@kcl.ac.uk

Table of Contents

1. General ... 1
2. Synthesis of Substrates ... 2
2.1 Synthesis and Characterisation of **7a** ... 2
2.2 Synthesis and characterization of **7g** ... 3
2.3 Synthesis and characterization of remaining substrates ... 5
3. General method for the generation of racemic products ... 10
4. HPLC Traces for Cyclised and Reduced Products ... 11
5. Synthesis and characterization of camphorsulfonyl derivative of **8a** ... 21
6. Synthesis and characterization of cannabinoid systems ... 22
7. NMR Spectra ... 25
8. XRD CheckCIF Data of Compound **17** ... 69
9. Unsuccessful Substrates ... 71
10. References ... 71

1. General

All reagents were supplied by Sigma Aldrich, Alfa-Aesar, Fisher and VWR and were used as supplied unless otherwise stated. Anhydrous DCM, THF, MeOH were obtained from a dry solvent system.

NMR data: Nuclear Magnetic Resonance spectra (NMR) were recorded in deuterated chloroform ($CDCl_3$) or methanol (CD_3OH) using a Bruker Ascend 400 (400 MHz) or a Bruker NEO 600 (600 MHz) spectrometer using TMS as internal standard. ^{1}H NMR splitting patterns are designated as singlet (s), doublet (d), doubledoubledoublet (ddd), doublet of triplets (dt), triplet (t), triplet of doublets (td), quintet (quint), quartet (q), doublet of doublet of triplets (ddt) and triplet of doublets of doublets (tdd). All first-order splitting patterns were assigned on the basis of the appearance of the multiplet. Splitting patterns that could not be easily interpreted are designated as multiplet (m) or broad (br) and the coupling constants *J* are quoted in Hz. All assignments are confirmed by 2D-COSY, 2D-HSQC and HSQC-TOCSY and NOESY measurements where necessary. ^{13}C NMR spectra were recorded at 100 MHz on a Bruker Ascend 400 (400 MHz). or a Bruker NEO 600 (150 MHz). NMRs taken on the Bruker NEO 600 (600MHz) were carried out at the NMR Facility of the Centre for Biomolecular Spectroscopy at King's College London. Thank you to Dr R.A. Atkinson for assistance in setting up the experiments.

IR data: IR spectra were recorded on a Shimadzu IRAffinity-1S FTIR Spectrophotometer as a thin film. The selected absorptions are quoted in wavenumbers (cm^{-1}).

MS Data: High-resolution mass spectra were recorded on either Waters LCT Premier (Es-Tof), Thermo Scientific Q-Exactive (APCI) and Micromass Autospec Premier (EI) by Imperial College London, Department of Chemistry Mass Spectrometry Service.

Specific Rotation: Specific rotations were taken using an Anton Parr MCP100 Polarimeter. Specific rotations ($[\alpha]_D^T$) were recorded at the sodium D line (589 nm) in methanol or dichloromethane and are quoted in : deg cm^2 g^{-1}. Solution

concentrations (c) are given in units of 10^{-2} g mL^{-1}. Temperatures are in degrees Celsius (°C). The prefixes (+) and (-) indicate the sign of the optical rotation. Correct units: deg cm^2 g^{-1}

Melting Point: Melting points were determined on a Stuart SMP30 melting point apparatus and are uncorrected.

HPLC Profiles: HPLC analysis was determined on Agilent Technologies 1200 Series HPLC, using a ratio of HPLC grade hexanes and propan-1-ol as the eluent, using a Chiralpak AD-H, OD or AS column (0.46 cm x 25 cm) and detection by UV at 210 nm.

Chromatography: Reactions were monitored by thin layer chromatography on silica gel precoated aluminium sheets (TLC Silica Gel 60 F_{254}, Merck). Visualisation was accomplished by irradiation by UV light at 254 nm and/or ninhydrin stain,potrassium permanganate stain or *p*-anisaldehyde. Column chromatography was performed on Merck silica gel (60 Å, 230 -400 mesh, 40 - 63 μm).

Catalyst V was purchased from Sigma Aldrich (CAS 1446629-74-4) as well as synthesised according to literature procedure.

2. Synthesis of Substrates

NB: Numbering follows that within the main manuscript, except if preceded by "S"

2.1 Synthesis and Characterisation of 7a

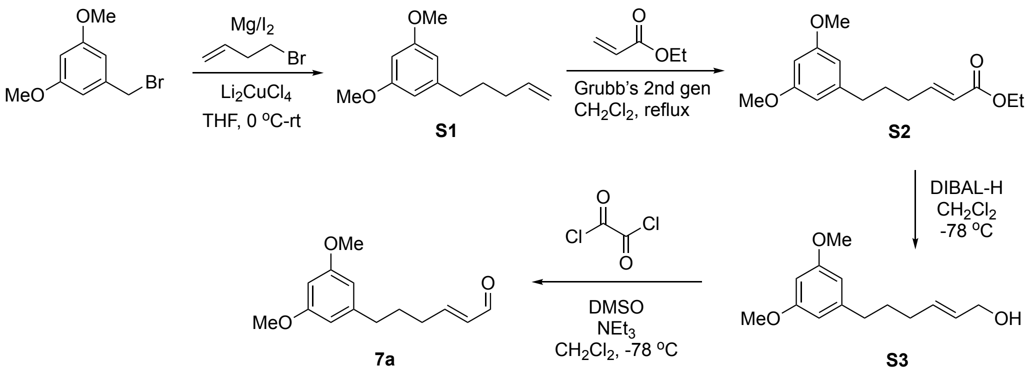

1,3-Dimethoxy-5-(pent-4-en-1-yl)benzene S1 : To a solution of magnesium turnings (1.44 g, 60.00 mmol) and iodine (~5 crystals) in dry THF (22.0 mL) at 0 °C was added bromobutene (5.08 mL, 50.00 mmol) dropwise over 10 min. The solution was warmed to room temperature and stirred for 2 h 20 min. To a separate flask was added lithium chloride (0.17 g, 4.00 mmol), cupper (II) chloride (0.27 g, 2.00 mmol) and 3,5-dimethoxybenzyl bromide (4.62 g, 20.00 mmol) in dry THF (200 mL). The Grignard solution was added dropwise at 0 °C to the dilithium tetrachlorocuprate (II) solution over 20 min. A further 18 mL of THF was used to transfer the Grignard solution to the reaction mixture. The reaction was warmed to room temperature and stirred for 2 h 40 min before being quenched with saturated ammonium chloride (aq) (150.0 mL). The layers were separated, and the aqueous layer extracted with Et_2O (x2), the combined organic layers were washed with brine and dried over $MgSO_4$. The crude material was purified used automated silica column chromatography (gradient, 100% hex-Hex:Et_2O 96.5:3.5) to yield 1,3-dimethoxy-5-(pent-4-en-1-yl)benzene (2.4 g, 59 % yield) as a clear oil.

^{1}H NMR (400 MHz, $CDCl_3$) δv6.36 (d, 2 H, J = 2.1 Hz, b-aromatic), 6.31 (t, 1 H, J = 2.2 Hz, d-aromatic), 5.83 (ddt, 1 H, J = 16.9, 6.7,10.3 and 6.7 Hz, 2-CH), 5.06-4.97 (m, 2 H, 1-CH_2), 3.78 (s, 6 H, 5-OMe), 2.59-2.55 (m, 2 H, 5-CH_2), 1.76-1.68 (m, 2 H, 3-CH_2), 1.76-1.68 (m, 2 H, 4-CH_2) ppm; ^{13}C NMR (101 MHz, $CDCl_3$) δ 160.87 (c-aromatic), 145.03 (a-aromatic), 138.72 (2-C), 114.87 (1-C), 106.68 (b-aromatic), 97.83 (d-aromatic), 55.37 (6-OMe), 35.77 (5-CH_2), 33.42 (3-C), 30.51 (4-CH_2) ppm

Ethyl (*E*)-6-(3,5-dimethoxyphenyl)hex-2-enoate S2 : To a solution of yield 1,3-dimethoxy-5-(pent-4-en-1-yl)benzene (4.87 g, 23.60 mmol) in dry DCM (234.0 mL) was added ethyl acrylate (25.73 mL, 235.96 mmol) and Grubbs 2nd Generation catalyst (0.38 g, 0.45 mmol). The solution was set to reflux for 30 h. Once all of the starting material had been consumed the solvent was removed under reduced pressure and the crude material was purified by automated silica gel chromatography (gradient, 100% hex-8:2 hex/Et_2O) to yield (E)-7-(3,5-dimethoxyphenyl)hept-3-enoate (5.4 g, 82 % yield) as an orange oil.

^{1}H NMR (400 MHz, $CDCl_3$) δ 6.97 (dt, 1 H, J = 15.6 and 6.9 Hz, 3-CH), 6.33-6.30 (m, 3 H, b-CH and d-CH), 5.81 (dt, 1 H, J = 15.6 and 1.5 Hz, 2-CH), 4.17 (q, 2 H, J = 7.1 Hz, 8-CH_2), 3.78 (s, 6 H, 7-OMe), 2.60-2.56 (m, 2 H, 6-CH_2), 2.26-2.20 (m, 2 H, 4-CH_2), 1.82-1.74 (m, 2 H, 5-CH_2), 1.28 (t, 3 H, J = 7.2 Hz, 8-CH_2) ppm; ^{13}C NMR (101 MHz, $CDCl_3$) δ 166.80 (q, 1-C), 160.90 (q-aromatic), 148.87 (3-C), 144.24 (q-aromatic), 121.80 (2-C), 106.59 b-aromatic), 97.94 (d-aromatic), 60.30 (8-C), 55.36 (7-OMe), 35.63 (6-C), 31.70 (4-C), 29.50 (5-C), 14.39 (8-C) ppm

(*E*)-6-(3,5-dimethoxyphenyl)hex-2-en-1-ol S3 : To a solution of ethyl (E)-7-(3,5-dimethoxyphenyl)hept-3-enoate (3.87 g, 13.91 mmol) in anhydrous DCM (56 mL) at -78 °C was added DIBAL-H (1.0 M in hexanes, 29.2 mL, 29.2 mmol) dropwise. The solution was stirred at -78 °C for 1 h at which point all of the starting material had been consumed. The reaction was quenched with 1 M HCl (40 mL) and left stirring at room temperature for 4 h. The aqueous phase was extracted with DCM (4 x 50 mL). The combined organic layers were dried over $MgSO_4$ and concentrated under reduced pressure to yield a pale yellow oil (3.2 g, 97% yield). No further purification was required.

^{1}H NMR (400 MHz, $CDCl_3$) δ 6.34 (d, 2 H, J = 2.22 Hz, b-CH), 6.30 (t, 1 H, J = 2.2 Hz, d-CH), 5.70-5.65 (m, 2 H, 2-CH and 3-CH), 4.09 (d, 2 H, J = 5.2 Hz, 1-CH_2), 3.78 (s, 6H, 7-OMe), 2.58-2.54 (m, 2 H, 6-CH_2), 2.11-2.06 (m, 2 H, 4-CH_2), 1.74-1.67 (m, 2 H, 5-CH_2), 148 (s, 1 H, OH), ppm; ^{13}C NMR (101 MHz, $CDCl_3$) δ 160.82 (q, aromatic), 144.86 (q, aromatic), 132.89 (3-C), 129.51 (2-C), 106.64 (b-aromatic), 97.78 (aromatic, d), 63.87 (1-C), 55.35 (7-OMe), 35.78 (6-C), 31.84 (4-C), 30.63 (5-C) ppm

(*E*)-6-(3,5-dimethoxyphenyl)hex-2-enal 7a : To a solution of oxalyl chloride (1.3 mL, 15.46 mmol) in anhydrous DCM (39 mL) at -78 °C was added dropwise a solution of anhydrous DMSO (2.74 mL, 38.64 mmol) in anhydrous DCM (11.5 mL). The mixture was left to stir for 10 min at which point a solution of (E)-6-(3,5-dimethoxyphenyl)hex-2-en-1-ol (2.28 g, 9.66 mmol) in anhydrous DCM (11.5 mL) was added dropwise. The reaction was left stirring at -78 °C for 1.5 h. A solution of anhydrous triethylamine (6.73 mL, 48.30 mmol) in anhydrous DCM (11.5 mL) was added dropwise to the reaction which was then warmed to room temperature. Once warmed, the reaction was quenched with distilled water (80 mL) and extracted with DCM (2 x 50 mL). The organic layers were combined and washed with distilled water (3 x 100 mL). The organic layer was dried over $MgSO_4$ and solvent was removed under reduced pressure to yield an orange oil (2.3 g, Quantitative yield). No further purification was required.
^{1}H NMR (400 MHz, $CDCl_3$) δ 9.51 (d, 1 H, J = 7.9 Hz, 1-CH), 6.85 (dt, 1 H, J = 15.7 and 6.8 Hz, 3-CH), 6.33-6.31 (m, 3 H, b-CH and d-CH), 6.12 (ddt, 1 H, J = 15.6, 7.8 and 1.4 Hz, 2-CH), 3.78 (s, 3 H, 7-OMe), 2.63-2.59 (m, 2 H, 6-CH_2), 2.39-2.33 (m, 2 H, 4-CH_2), 1.88-1.80 (m, 2 H, 5-CH_2) ppm; ^{13}C NMR (101 MHz, $CDCl_3$) δ 194.16 (1-C), 160.98 (q, aromatic), 158.38 (q, aromatic), 143.88 (3-C), 133.35 (2-C), 106.66 (aromatic), 97.97 (aromatic), 55.39 (7-OMe), 35.63 (6-C), 32.21 (4-C), 29.29 (5-C) ppm; Found 235.1339; IR (diamond) υ 2999, 2938, 2838, 1684, 1636, 1593, 972, 830 cm^{-1}; HRMS (EI) Exact mass calculated for $C_{14}H_{24}O_3$ [M+H]+235.1334;

2.2 Synthesis and characterization of 7g

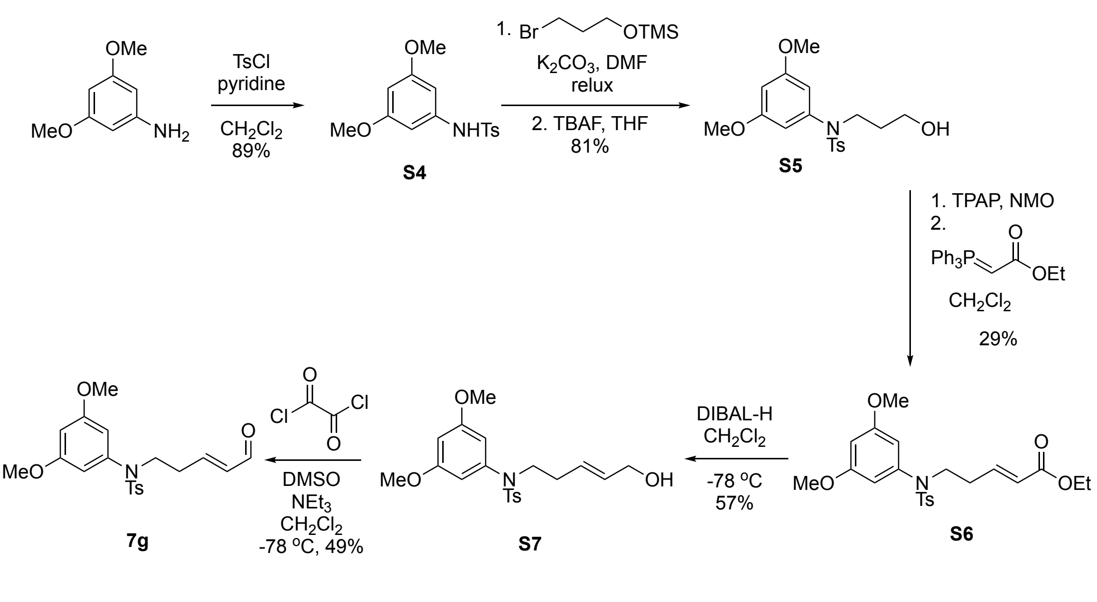

***N*-(3,5-dimethoxyphenyl)-4-methylbenzenesulfonamide S4**[1] : To a solution of 3,5-dimethoxyaniline (3.83 g, 25.00 mmol) in dry DCM (83 mL) was added anhydrous pyridine (12.13 mL, 150.00 mmol) and tosyl chloride (5.72 g, 30.00 mmol). After stirring for 20h, the reaction was quenched with distilled water (50 mL), washed with brine (50 mL) and dried over Na_2SO_4. Solvent was removed under vacuum. The crude mixture was diluted with EtOAc (20 mL) and

washed again with 1M HCl (20 mL), distilled water (20 mL) and brine (20 mL) to remove residual pyridine. The organic layer was dried over Na_2SO_4 and solvent removed via reduced vacuum to yield 7.5 g (89% yield) of N-(3,5-dimethoxyphenyl)-4-methylbenzenesulfonamide as a brown oil which required no further purification.

^{1}H NMR (400 MHz, $CDCl_3$) δ 7.74 (d, 2 H, J = 8.1 Hz, b'-aromatic), 7.27 (s, 1 H, 1-NH), 7.23 (d, 2 H, J = 8.1 Hz, c'-aromatic), 6.28 (d, 2 H, J = 2.2 Hz, b-aromatic), 6.16 (t, 1 H, J = 2.2 Hz, d-aromatic), 3.69 (s, 6 H, 2-OMe), 3.36 (s, 3 H, 3-CH_3) ppm; ^{13}C NMR (101 MHz, $CDCl_3$) δ 161.26 (c-aromatic), 144.08 (q-aromatic), 138.58 (q-aromatic), 136.03 (q-aromatic), 129.81 (c'-aromatic), 127.45 (b'-aromatic), 99.00 (b-aromatic), 97.18 (d-aromatic), 55.48 (2-OMe), 21.66 (3-C) ppm

***N*-(3,5-dimethoxyphenyl)-*N*-(3-hydroxypropyl)-4-methylbenzenesulfonamide S5** : To solution of N-(3,5-dimethoxyphenyl)-4-methylbenzenesulfonamide (6.84 g, 22.24 mmol) in dry DMF (37.00 mL) was added potassium carbonate (6.15 g, 44.48 mmol), and 3-bromopropoxy-trimethlsilane (7.30 mL, 31.51 mmol). The solution was heated to reflux for 18 h. The reaction was quenched with distilled water (30 mL), extracted with diethyl ether (2 x 30 mL), washed with brine and dried over Na_2SO_4. The crude material was dissolved in dry THF (44 mL). To the solution was added TBAF solution (1.0 M, 44.50 mL, 44.50 mmol). The reaction was stirred at room temperature for 4 h and quenched with 1M HCl (40 mL). The aqueous phase was extracted with diethyl ether (2 x 40 mL) and dried over Na_2SO_4. The residue was purified by automated column chromatography on silica gel (gradient 100% hex to 55% EtOAc) to yield 6.5 g (81 % yield) of N-(3,5-dimethoxyphenyl)-N-(3-hydroxypropyl)-4-methylbenzenesulfonamide as a pale yellow oil.

^{1}H NMR (400 MHz, $CDCl_3$) δ 7.54.7.52 (m, 2 H, b'-aromatic), 7.26-7.24 (m, 2 H, c'-aromatic), 6.38 (t, 1 H, J = 2.2 Hz, d-aromatic), 6.20 (d, 2 H, J = 2.3 Hz, b-aromatic), 3.76-3.74 (m, 2 H, 1-CH_2), 3.70 (s, 6 H, 4-OMe), 3.62-3.58 (t, 2 H, J = 6.3 Hz, 3-CH_2), 2.40 (s, 3 H, 5-CH_3), 2.24 (m, 1 H, 1-OH), 1.61 (m, 2 H, 2-CH_2) ppm; ^{13}C NMR (101 MHz, $CDCl_3$) δ 160.87 (4-OMe), 143.72 (a-aromatic), 140.85 (d'-aromatic), 135.28 (a'-aromatic), 129.60 (c'-aromatic), 127.84 (b'-aromatic), 107.19 (b-aromatic), 100.30 (d-aromatic), 58.90 (1-C), 55.56 (4-OMe), 47.29 (3-C), 30.66 (2-C), 21.68 (5-C) ppm

Ethyl (*E*)-5-((*N*-(3,5-dimethoxyphenyl)-4-methylphenyl)sulfonamido)pent-2-enoate S6 : N-(3,5-dimethoxyphenyl)-N-(3-hydroxypropyl)-4-methylbenzenesulfonamide (0.28 g, 0.75 mmol) was oxidised with TPAP (0.013 g, 0.038 mmol) in the presence of NMO (0.13 g, 1.13 mmol) and homologated with (carbethoxymethylene) triphenylphosphorane (0.37 g, 1.05 mmol) to yield 0.09 g (29 % yield) of ethyl (E)-5-((N-(3,5-dimethoxyphenyl)-4-methylphenyl)sulfonamido)pent-2-enoate as a pale yellow oil after automated column chromatography (gradient, Hex/Et_2O, 100%-1:1).

^{1}H NMR (400 MHz, $CDCl_3$) δ 7.53-7.51 (m, 2 H, b'-aromatic), 7.27-7.25 (m, 2 H, c'-aromatic), 6.83 (dt, 1 H, J = 15.7 and 7.1 Hz, 3-CH), 6.39 (t, 1 H, J = 2.3 Hz, d-aromatic), 6.17 (d, 2 H, J = 2.3 Hz, b-aromatic), 5.80 (dt, 1 H, J = 15.6 and 1.4 Hz, 2-CH), 4.17 (q, 2 H, J = 7.1 Hz, 8-CH_3), 3.70 (s, 6 H, 6-OMe), 3.59 (t, 2 H, J = 7.2 Hz, 5-CH_2), 2.42 (s, 3 H, 7-CH_3), 2.39-2.33 (m, 2 H, 4-CH_2), 1.27 (t, 3 H, J = 7.1 Hz, 9-CH_3) ppm; ^{13}C NMR (101 MHz, $CDCl_3$) δ 166.23 (1-C), 160.87 (c-aromatic), 144.50 (3-C), 143.74 (q-aromatic), 140.69 (q-aromatic), 135.06 (q-aromatic), 129.55 (c'-aromatic), 127.95 (b'-aromatic), 123.59 (2-C), 107.16 (b-aromatic), 100.54 (d-aromatic), 60.43 (8-C), 55.55 (6-OMe), 49.24 (5-C), 31.39 (4-C), 21.67 (7-C), 14.35 (9-C) ppm;

(*E*)-*N*-(3,5-Dimethoxyphenyl)-*N*-(5-hydroxypent-3-en-1-yl)-4-methylbenzenesulfonamide S7 : -(3,5-dimethoxyphenyl)-4-methylphenyl)sulfonamido)pent-2-enoate (1.05 g, 2.41 mmol) was reduced with DIBAL-H (1.0 M in hexanes, 6.51 mL, 6.51 mmol).The crude mixture was purified using silica gel on column chromatography (Et_2O/Hex, 9:1) to yield 0.6 g (57 % yield) of (E)-N-(3,5-dimethoxyphenyl)-N-(5-hydroxypent-3-en-1-yl)-4-methylbenzenesulfonamide as a clear oil.

^{1}H NMR (400 MHz, $CDCl_3$) δ 7.53-7.51 (m, 2 H, b'-aromatic), 7.26-7.24 (m, 2 H, c'-aromatic), 6.39 (t, 1 H, J = 2.3 Hz, b-aromatic), 6.19 (d, 1 H, J = 2.3 Hz, d-aromatic), 5.63-5.60 (m, 2 H, 2-CH and 3-CH), 4.05 (m, 2 H, 1-CH_2), 3.71 (s, 6 H, 6-OMe), 3.53 (t, 2 H, J = 7.2 Hz, 5-CH_2), 2.41 (s, 3 H, 7-CH_3), 2.21-2.16 (m, 2 H, 4-CH_2), 1.61 (m, 1 H, 1-OH) ppm; ^{13}C NMR (101 MHz, $CDCl_3$) δ 160.77 (6-OMe), 143.57 (q-aromatic), 140.85 (q-aromatic), 135.36 (q-aromatic), 131.82 (2-C), 129.48 (c'-aromatic), 128.58 (3-C), 127.87 (b'-aromatic), 107.25 (b-aromatic), 100.30 (d-aromatic), 63.54 (1-C), 55.54 (6-OMe), 50.31 (5-C), 31.31 (4-C), 21.65 (7-C) ppm

(*E*)-*N*-(3,5-dimethoxyphenyl)-*N*-(5-hydroxypent-3-en-1-yl)-4-methylbenzenesulfonamide 7g : (E)-N-(3,5-dimethoxyphenyl)-N-(5-hydroxypent-3-en-1-yl)-4-methylbenzenesulfonamide (0.61 g, 1.56 mmol) was oxidised with oxalyl chloride (0.21 mL, 2.50 mmol) and DMSO (0.44 mL, 6.25 mmol). The crude mixture was purified using silica gel on column chromatography (Hex/Et_2O, 3:1) to yield 0.3 g (49 % yield) of (E)-N-(3,5-dimethoxyphenyl)-4-methyl-N-(5-oxopent-3-en-1-yl)benzenesulfonamide as a yellow oil which required no further purification.

m.p: 104.9-106.3 °C ^{1}H NMR (400 MHz, $CDCl_3$) δ 9.51 (d, 1 H, J = 7.8 Hz, 1-CH), 7.55-7.53 (m, 2 H, b'-aromatic), 7.30-7.28 (m, 2 H, c'-aromatic), 6.81 (dt, 1 H, J = 6.9 and 15.8 Hz, 3-CH), 6.43 (t, 1 H, J = 2.2 Hz, d-aromatic), 6.19 (d, 2 H, J = 2.2 Hz, b-aromatic), 6.10 (ddt, 1 H, J = 15.8, 7.9 and 1.3 Hz, 2-CH), 3.75-2.68 (m, 8 H, 5-CH_2 and 6-OMe), 2.54-2.49 (m, 2 H, 4-CH_2), 2.45 (s, 3 H, 7-CH_3) ppm; ^{13}C NMR (101 MHz, $CDCl_3$) δ 193.76 (1-C), 160.96 (6-OMe), 153.96 (3-C), 143.90 (q-aromatic), 140.47 (q-aromatic), 134.98 (q-aromatic), 134.96 (2-C), 129.62 (b'-aromatic), 127.92 (c'-aromatic), 107.16 (b-aromatic), 100.50 (d-aromatic), 55.59 (6-OMe), 48.97 (5-C), 31.71 (4-C), 21.69 (7-C) ppm; IR (Diamond) υ 3003, 2977, 2936, 2839, 1718, 1678, 1204, 964, 855, 815 cm^{-1} HRMS (ES-Tof) Exact mass calculated for $C_{20}H_{23}NO_5S$ [M+H]+ 390.1375, Found 390.1372

2.3 Synthesis and characterization of remaining substrates

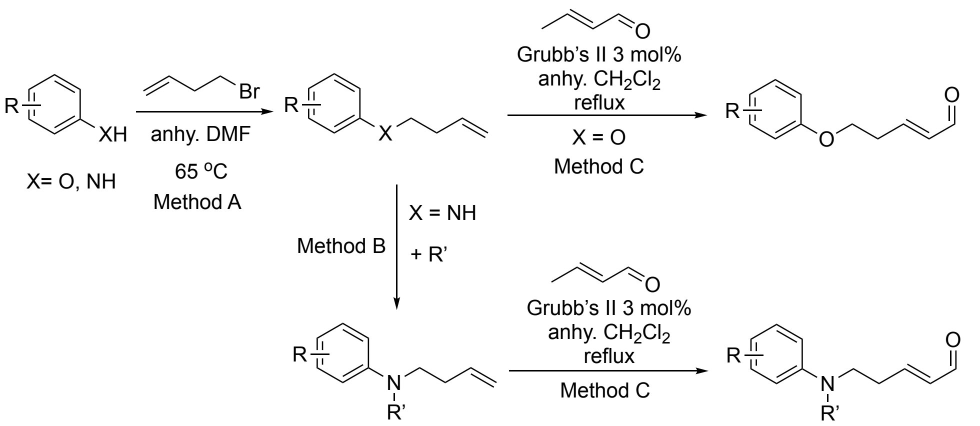

Method A

To a solution of phenol/aniline in (1 equiv) in dry DMF (0.6 M) was added 4-bromo-1-butene (1.5 equiv) and K_2CO_3 (2 equiv). The reaction was heated to 65 °C for between 17 and 36 h, quenched with distilled water and extracted with Et_2O (x2). The combined organic layers were washed with brine saturated LiCl and dried over $MgSO_4$. The crude mixture was concentrated under reduced pressure and purified by column chromatography on silica gel.

Method B

To a solution of homologated aniline (1 equiv) in dry DCM (0.5 M) at 0 °C was added dry diisopropylamine (1.5 equiv) and acylating or sulfonylating agent (1.1 equiv). The solution was warmed to room temperature and left stirring overnight. Once all of the starting material had been consumed, the reaction was quenched with saturated $NaHCO_3$ (aq) and extracted with DCM (x3). The combined organic layers were washed with 1 N HCl (aq) and dried over $MgSO_4$.

Method C

To a solution of homologated aniline/phenol (1 equiv) in dry DCM (0.1 M) was added crotonaldehyde (10 equiv) and Grubbs 2nd Generation catalyst (3 mol%). The mixture was heated to reflux for 16 h before the solvent was removed by reduced pressure and by N_2. The crude mixture was purified using silica gel on column chromatography (Pentane/ Et_2O).

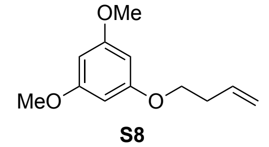

S8

1-(but-3-en-1-yloxy)-3,5-dimethoxybenzene S8 : Following method A, 3,5-dimethoxy phenol (1.00 g, 6.49 mmol) was reacted with 4-bromo-1-butene (0.99 mL, 9.73 mmol). The crude mixture was purified using silica gel on column chromatography (Hex/EtOAc, 9:1) to yield 0.7 g (55 %) of 1-(but-3-en-1-yloxy)-3,5-dimethoxybenzene as a clear oil.

^{1}H NMR (400 MHz, $CDCl_3$) δ 6.09 (s, 3 H, 2'-H and 4'-H), 5.90 (ddt, 1 H, J = 17.1, 10.3 and 6.7 Hz, 2-CH), 5.20 (m, 1 H,1''-H),), 5.12 (m, 1 H, 1'-H), 3.98 (t, 2 H, J = 6.7 Hz, 4-CH_2), 3.77 (s, 6 H, 5-OMe), 2.53 (qt, 2 H, J = 6.7 and 1.3 Hz, 3-$CH_2$2.53 (qt, 1 H, J = 6.6 and 1.3 Hz, 3-CH_2) ppm; ^{13}C NMR (101 MHz, $CDCl_3$) δ 161.59 (q-aromatic), 160.91 (q-aromatic), 134.53 (2-C), 117.13 (3-C), 93.51 (b-aromatic), 93.10 (d-aromatic), 67.30 (4-C), 55.51 (5-OMe), 33.67 (3-C) ppm

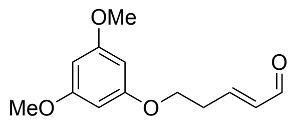

7b

(*E*)-5-(3,5-dimethoxyphenoxy)pent-2-enal 7b : Following method B, 1-(but-3-en-1-yloxy)-3,5-dimethoxybenzene (0.74 g, 3.54 mmol) was coupled with crotonaldehyde (2.93 mL, 35.36 mmol) using Grubbs 2nd Generation catalyst (0.09 g, 0.11 mmol). The reaction yielded 0.7 g of (E)-5-(3,5-dimethoxyphenoxy)pent-2-enal after column chromatography (pent/Et_2O, 9:1).

m.p: 35.2-36.0 °C ^{1}H NMR (400 MHz, $CDCl_3$) δ 9.53 (d, 1 H, J = 7.9 Hz, 1-CH), 6.93 (dt, 1 H, J = 15.7 and 6.7 Hz, 3-CH), 6.26-6.20 (m, 1 H, 2-CH), 6.10-6.06 (m, 3 H, b and d aromatic), 4.08 (t, 2 H, J = 6.1 Hz, 5-CH_2), 3.76 (s, 6 H, 6-OMe), 2.82-2.77 (m, 2 H, 4-CH_2) ppm; ^{13}C NMR (101 MHz, $CDCl_3$) δ 193.93 (1-C), 161.66 (q-aromatic), 160.41 (q-aromatic), 154.09 (3-C), 134.64 (2-C), 93.48 (b-aromatic), 93.39 (d-aromatic), 65.65 (5-C), 55.45 (6-OMe), 32.51 (4-C) ppmIR (Diamond) υ 3006, 2968, 2933, 2833, 2751, 1686, 1684, 1594, 1259, 1059, 984, 832 cm^{-1}; HRMS (EI) Exact mass calculated for $C_{13}H_{16}O_4$ [M+H]+ 237.1127

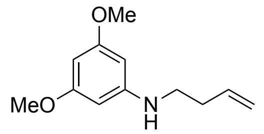

S9

***N*-(but-3-en-1-yl)-3,5-dimethoxyaniline S9** : Following method A, 3,5-dimethoxy aniline (7.71 g, 50.00 mmol) was reacted with 4-bromo-1-butene (2.03 mL, 20.00 mmol). The crude mixture was purified using automated silica gel on column chromatography (gradient, 100% hex-15% Et_2O) to yield 3.9 g (94 %) of but-3-en-1-yl(3,5-dimethoxyphenyl)-l2-azane as a pale yellow oil.

^{1}H NMR (400 MHz, $CDCl_3$) δ 5.88 (t, 1 H, J = 2.2 Hz, d-aromatic), 5.87-5.77 (m, 3 H, b-aromatic and 2-CH), 5.18-5.09 (m, 2 H, 1-CH_2), 3.75 (s, 6 H, 5-OMe), 3.70 (s, 1 H, NH), 3.16 (t, 2 H, J = 6.7 Hz, 4-CH_2), 2.37 (qt, 2 H, J = 1.2 and 6.7 Hz, 3-CH_2) ppm; ^{13}C NMR (101 MHz, $CDCl_3$) δ 161.85 (c-aromatic), 150.30 (a-aromatic), 135.85 (2-C), 117.26 (1-C), 91.81 b-aromatic), 89.86 (d-aromatic), 55.28 (5-OMe), 42.91 (4-C), 33.70 (3-C) ppm; HRMS (ES-Tof) Exact mass calculated for $C_{12}H_{17}NO_2$ [M+H]+ 208.1338, Found 208.1341 IR (Diamond) υ 3405, 2958, 2936, 2839, 1612, 1590, 1201, 1175, 915, 807, 681 cm^{-1}

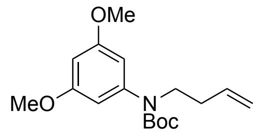

S10

***tert*-butyl but-3-en-1-yl(3,5-dimethoxyphenyl)carbamate S10** : Following method B, to a solution of N-(but-3-en-1-yl)-3,5-dimethoxyaniline (0.68 mmol, 3.30 mmol) in dry THF (33 mL) was added anhydrous trimethylamine (1.38 mL, 9.90 mmol) and di-*tert*-butyl dicarbonate (0.86 g, 3.96 mmol). The reaction was stirred for 24 h and monitored by thin layer chromatography. Due to the lack of complete consumption of starting material, a further 1.2 equiv (0.86 g, 3.96 mmol) of di-*tert*-butyl dicarbonate was added and the reaction was heated to 45 °C. Solvent was removed after a further 24 h. The crude mixture was partitioned between EtOAc and distilled water and washed with 1N HCl, water and brine. The organic layer was dried over $MgSO_4$ and solvent was removed under reduced pressure. The reaction yielded 0.5 g (45 %yield) of tert-butyl but-3-en-1-yl(3,5-dimethoxyphenyl)carbamate as a clear oil after purification using automated column chromatography (100% hex to 9:1 Hex/EtOAc).

^{1}H NMR (400 MHz, $CDCl_3$) δ 6.36 (d, 2 H, J = 2.2 Hz, b-aromatic), 6.33 (t, 1 H, J = 2.2 Hz, d-aromatic), 5.77 (ddt, 1 H, J = 17.2, 6.8 and 10.2 Hz, 2-CH), 5.08-5.00 (m, 2 H, 1-CH_2), 3.77 (s, 6 H, 5-OMe), 5.68-3.64 (m, 2 H, 4-CH_2), 2.33-2.27 (m, 1 H, 3-CH_2), 1.43 (s, 9 H, 8-CH_3) ppm; ^{13}C NMR (101 MHz, $CDCl_3$) δ 160.75 (c-aromatic), 154.61 (6-C), 144.26 (q-aromatic), 135.56 (2-C), 116.70 (1-C), 105.92 (b-aromatic), 98.37 (d-aromatic), 80.25 (7-C), 55.50 (5-OMe), 49.55 (4-C), 33.12 (3-C), 28.48 (8-C) ppm; HRMS (ES-Tof) Exact mass calculated for $C_{17}H_{25}NO_4$ [M+H]+ 308.1862, Found 308.1863 IR (Diamond) υ 3005, 2975, 2933, 2842, 1696, 1593, 913, 857, 830, 697 cm^{-1}

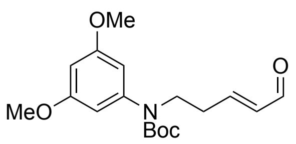

7c

***tert*-butyl (*E*)-(3,5-dimethoxyphenyl)(5-oxopent-3-en-1-yl)carbamate 7c** : Following method C, tert-butyl but-3-en-1-yl(3,5-dimethoxyphenyl)carbamate (0.46 g, 1.50 mmol) was coupled with crotonaldehyde (1.24 mL, 14.96 mmol) using Grubbs 2nd Generation catalyst (0.038 g, 0.045 mmol). The reaction yielded 0.4 g (70 %) of *tert*-butyl (E)-(3,5-dimethoxyphenyl)(5-oxopent-3-en-1-yl)carbamate as a pale yellow oil after automated column chromatography (gradient, 100% Pent-65:35 Pent/Et_2O).

^{1}H NMR (400 MHz, $CDCl_3$) δ 9.47 (d, 1 H, J = 7.8 Hz, 1-CH), 6.81 (dt, 1 H, J = 15.6 and 6.9 Hz, 3-CH), 6.34-6.30 (m, 3 H, b and d-aromatic), 6.15-6.09 (m, 1 H, 2-CH), 3.81 (t, 2 H, J = 6.9 Hz, 5-CH_2), 3.79 (s, 6 H, 6-OMe), 2.56 (m, 2 H, 4-CH_2), 1.41 (s, 9 H, 9-CH_3) ppm; ; ^{13}C NMR (101 MHz, $CDCl_3$) δ 193.84 (1-C), 160.91 (q-aromatic), 155.11 (3-C), 154.51 (q-aromatic), 143.63 (q-aromatic), 134.39 (2-C), 105.88 (b-aromatic), 98.51 (d-aromatic), 80. 75 (8-C), 55.52 (6-OMe), 48.24 (5-C), 32.20 (4-C), 28.42 (9-C) ppm; HRMS (ES-Tof) Exact mass calculated for $C_{18}H_{27}NO_5$ [M+H]+ 336.1811, Found 336.1819 IR (Diamond) υ 2982, 2969, 2929, 2866, 2827, 1690, 1205, 987, 847 cm^{-1}

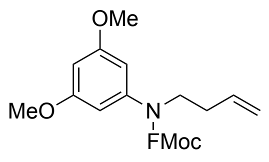

S11

(9*H*-fluoren-9-yl)methyl but-3-en-1-yl(3,5-dimethoxyphenyl)carbamate S11 : To a solution of but-3-en-1-yl(3,5-dimethoxyphenyl)-l2-azane (0.64 g, 3.10 mmol) and 10% $NaHCO_3$ (aq) (34 mL) in dioxane (34.00 mL) at 0 °C was slowly added a solution of fluorenylmethyloxycarbonyl chloride (2.01 g, 7.75 mmol) in dioxane (17.00 mL). The temperature of the reaction was maintained at 0 °C for 30 min before being raised to room temperature and stirred overnight. Once all of the starting material had been consume, the reaction was quenched with distilled water and extracted with diethyl ether. The combined organic layers were washed with brine and dried over $MgSO_4$. The crude mixture was purified using automated column chromatography (9:1, hex/Et_2O) to yield (9H-fluoren-9-yl)methyl but-3-en-1-yl(3,5-dimethoxyphenyl)carbamate as a clear oil in 87 % yield (1.16 g).

^{1}H NMR (400 MHz, $CDCl_3$) δ 7.22 (d, 2 H, J = 7.56 Hz, f'-aromatic), 7.38-7.21 (m, 6 H, c', d' and e'-aromatic), 6.46 (s, 1 H, d-aromatic), 6.39 (d, 2 H, J = 2.3 Hz, b-aromatic), 5.73 (s, 1 H, a'-aromatic), 5.08-5.03 (m, 2 H, 7-CH_2), 4.36 (s, 2 H, 1-CH_2), 4.13 (s, 1 H, 2-CH), 3.80 (s, 6 H, 5-OMe), 3.68 (s, 2 H, 4-CH_2), 2.29 (s, 2 H, 3-CH_2) ppm; ^{13}C NMR (101 MHz, $CDCl_3$) δ 161.18 (c-aromatic), 155.45 (6-C), 144.02 (g'-aromatic), 143.36 (a-aromatic), 141.41 (b'-aromatic), 135.30 (a'-aromatic), 127.71 (e'-aromatic), 127.02 (d'-aromatic), 125.34 (c'-aromatic), 119.99 (f'-aromatic), 116.97 (7-C), 106.55 (b-aromatic), 99.42 (d-aromatic), 67.63 (1-C), 55.61 (5-OMe), 49.79 (4-C), 47.23 (2-C), 32.82 (3-C) ppm; HRMS (EI) Exact mass calculated for $C_{27}H_{27}NO_4$ [M+H]+ 430.2018, Found 430.2009 IR (Diamond) υ 3067, 2938, 2843, 1702, 1605, 1594, 926, 758 cm^{-1}

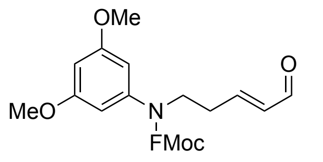

7d

(9*H*-Fluoren-9-yl)methyl (*E*)-(3,5-dimethoxyphenyl)(5-oxopent-3-en-1-yl)carbamate 7d : Following method C, (9H-fluoren-9-yl)methyl but-3-en-1-yl(3,5-dimethoxyphenyl)carbamate (1.12 g, 2.61 mmol) was coupled with crotonaldehyde (2.16 mL, 26.07 mmol) using Grubbs 2nd Generation catalyst (0.066 g, 0.078 mmol). The reaction yielded 1.1 g (89 %) of (9H-fluoren-9-yl)methyl (E)-(3,5-dimethoxyphenyl)(5-oxopent-3-en-1-yl)carbamate as viscous brown oil after automated column chromatography (gradient, 100% Pent-55:45 Pent/Et_2O).

^{1}H NMR (400 MHz, $CDCl_3$) δ 9.48 (d, 1 H, J = 8.1 Hz, 1-CH), 7.72 (d, 2 H, J = 7.7 Hz, f'-aromatic), 7.38-7.21 (m, 6 H, c', d', e'-aromatic), 6.73 (s, 1 H, 3-CH), 6.45 (s, 1 H, d-aromatic), 6.31 (d, 2 H, J = 2.0 Hz, b-aromatic), 6.10 (s, 1 H, 2-CH), 4.39 (s, 2 H, 8-CH_2), 3.96 (s, 1 H, a'-aromatic), 3.79 (s, 8 H, 5-CH_2 and 6-OMe), 2.53 (s, 2 H, 4-CH_2) ppm; ^{13}C NMR (101 MHz, $CDCl_3$) δ 193.79 (1-C), 161.34 (c-aromatic), 155.40 (), 154.51 (), 143.85 (g'-aromatic), 142.85 (a-aromatic), 141.43 (b'-aromatic), 134.53 (2-C), 127.78 (d'-aromatic), 127.06 (e'-aromatic), 125.21 (c'-aromatic), 120.03 (f'-aromatic), 106.32 (b-aromatic), 99.51 (d-aromatic), 67.71 (8-C), 55.63 (6-OMe), 48.61 (5-C), 47.16 (a'-aromatic), 31.87 (4-C) ppm; HRMS (EI) Exact mass calculated for $C_{28}H_{27}NO_5$ [M+H]+ 458.1967, Found 458.1978 IR (Diamond) υ 3017, 3004, 2971, 2839, 1740, 1684, 1592, 993, 758 cm^{-1}

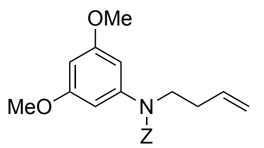

S12

Benzyl but-3-en-1-yl(3,5-dimethoxyphenyl)carbamate S12 : To a solution of N-(but-3-en-1-yl)-3,5-dimethoxyaniline (0.68 mmol, 3.30 mmol) in dry THF (8 mL) at 0 °C was added sodium bicarbonate (0.31 g, 3.63 mmol) and benzyl chloroformate (0.51 mL, 3.63 mmol). After 15 min, the reaction was warmed to rt and left stirring overnight. Once all of the starting material had been consumed the reaction was quenched with distilled water and the layers separated. The aqueous phase was extracted with EtOAc (x2) and dried over $MgSO_4$. The crude mixture was purified by column chromatography on silica gel (Hex/EtOAc, 8:2) to yield benzyl but-3-en-1-yl(3,5-dimethoxyphenyl)carbamate as a clear oil (1.0 g, 88 % yield).

^{1}H NMR (400 MHz, $CDCl_3$) δ 7.37-7.28 (m, 5 H, b', c' nd d'-aromatic), 6.40 (s, 3 H, b and d-aromatic), 5.78 (ddt, 1 H, J = 7.0, 10.1 and 17.1 Hz, 2-CH), 5.18 (s, 2 H, 7-CH_2), 5.10-5.03 (m, 2 H, 1-CH_2), 3.78-3.74 (m, 8 H, 5-OMe and 4-CH_2), 2.37-2.31 (m, 2 H, 3-CH_2) ppm; ^{13}C NMR (101 MHz, $CDCl_3$) δ 160.91 (q-aromatic), 155.33 (q-aromatic), 143.43 (q-aromatic), 136.85 (q-aromatic), 135.26 (2-C), 128.50 (d'-aromatic), 127.77 (c'aromatic), 127.96 (b'-aromatic), 116.95 (1-C), 105.97 (b-aromatic), 99.13 (d-aromatic), 67.22 (7-C), 55.50 (5-OMe), 49.90 (4-C), 32.89 (3-C) ppm; HRMS (ES-Tof) Exact mass calculated for $C_{20}H_{23}NO_4$ [M+H]+ 342.1705, Found 342.1720 IR (Diamond) υ 3074, 2006, 2944, 2842, 1701, 1592, 1204, 832, 694 cm^{-1}

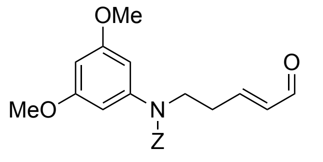

7e

Benzyl (*E*)-(3,5-dimethoxyphenyl)(5-oxopent-3-en-1-yl)carbamate 7e : Following method C, benzyl but-3-en-1-yl(3,5-dimethoxyphenyl)carbamate (1.00 g, 2.92 mmol) was coupled with crotonaldehyde (2.42 mL, 29.15 mmol) using Grubbs 2nd Generation catalyst (0.074 g, 0.087 mmol). The reaction yielded 1.0 g (79 %) of benzyl (E)-(3,5-dimethoxyphenyl)(5-oxopent-3-en-1-yl)carbamate as a pale brown solid after automated column chromatography (gradient, 100% Pent-6:4 Pent/Et_2O).

m.p: 51.6-52.5 °C ^{1}H NMR (400 MHz, $CDCl_3$) δ 9.43 (d, 1 H, J = 7.8 Hz, 1-CH), 7.34-7.26 (m, 5 H, a', b', c' and d'-aromatic), 6.76 (dt, 1 H, J = 15.7 and 7.1 Hz, 3-CH), 6.38 (t, 1 H, J = 2.3 Hz, d-aromatic), 6.31 (s, 2 H, b-aromatic), 6.09 (ddt, 1 H, J = 15.7, 7.8 and 1.3 Hz, 2-CH),5.15 (s, 2 H, 8-CH_2), 3.88 (t, 2 H, J = 7.1 Hz, 5-CH_2), 3.74 (s, 6 H, 6-OMe), 2.61-2.56 (m, 2 H, 4-CH_2) ppm; ^{13}C NMR (101 MHz, $CDCl_3$) δ 193.78 (1-C), 161.12 (c-aromatic), 155.32 (7-aromatic), 154.57 (3-C), 142.87 (a-aromatic), 136.57 (a'-aromatic), 134.58 (2-C), 128.62 (c'-aromatic), 128.20 (d'-aromatic), 127.89 (b'-aromatic), 105.92 (b-aromatic), 99.31 (d-aromatic), 67.53 (8-C), 55.56 (6-OMe), 48.70 (5-C), 31.99 (4-C), ppm; HRMS (ES-Tof) Exact mass calculated for $C_{21}H_{23}NO_5$ [M+H]+ 370.1654, Found 370.1655 IR (Diamond) υ 2993, 2944, 2821, 2745, 1696, 1679, 1591, 1231, 1148, 995, 964, 853, 743 cm^{-1}

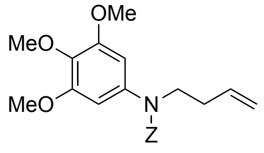

S13

Benzyl but-3-en-1-yl(3,4,5-trimethoxyphenyl)carbamate S13 : To a solution of N-(but-3-en-1-yl)-3,4,5-trimethoxyaniline (0.69 mmol, 2.89 mmol) in dry THF (7.20 mL) at 0 °C was added sodium bicarbonate (0.27 g, 3.18 mmol) and benzyl chloroformate (0.45 mL, 3.18 mmol). After 15 min, the reaction was warmed to rt and left stirring overnight. Once all of the starting material had been consumed the reaction was quenched with distilled water and the layers separated. The aqueous phase was extracted with EtOAc (x2) and the combined organic phases combined and dried over MgSO4. The crude pale yellow oil was used in the next step without further purification (1.0 g, 89 % yield).

^{1}H NMR (400 MHz, CDCl3) δ 7.40-7.31 (m, 5 H, b', c' and d'-aromatic), 6.41 (s, 2 H, b-aromatic), 5.76 (ddt, 1 H, J = 6.9 10.2 and 17.0 Hz, 2-CH), 5.16 (s, 2 H, 8-CH2), 5.08-5.02 (m, 2 H, 1-CH2), 3.85 (s, 3 H, 6-OMe), 3.79 (s, 6 H, 5-OMe), 3.74-3.71 (m, 2 H, 4-CH2), 2.35-2.30 (m, 2 H, 3-CH2) ppm; 13C NMR (101 MHz, CDCl3) δ 155.52 (7-C), 153.33 (c-aromatic), 136.95 (a-aromatic), 136.83 (d-aromatic), 135.36 (2-C), 129.49 (d'-aromatic), 128.54 (c'-aromatic), 128.05 (b'-aromatic), 127.85 (a'-aromatic), 117.00 (1-C), 105.24 (b-aromatic), 67.24 (8-C), 61.01(6-C), 56.23 (5-C), 50.09 (4-C), 46.40 (3-C) ppm; HRMS (EI) Exact mass calculated for $C_{21}H_{25}NO_5$ [M+H]+ 372.1811, Found 372.1811 IR (Diamond) υ 3075, 2939, 2832, 1777, 1698, 1641, 1228, 917, 831, 767 cm

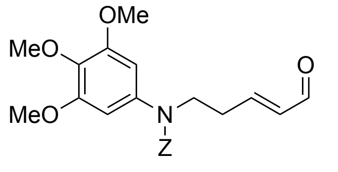

7f

Benzyl (*E*)-(5-oxopent-3-en-1-yl)(3,4,5-trimethoxyphenyl)carbamate 7f : Following method C, benzyl but-3-en-1-yl(3,4,5-trimethoxyphenyl)carbamate (1.02 g, 2.76 mmol) was coupled with crotonaldehyde (2.12 mL, 25.64 mmol) using Grubbs 2nd Generation catalyst (0.065 g, 0.077 mmol). The reaction yielded 0.9 g (77 %) of benzyl (E)-(5-oxopent-3-en-1-yl)(3,4,5-trimethoxyphenyl)carbamate as a dark brown oil after automated column chromatography (gradient, 100% Pent-7:3 Pent/Et_2O).

^{1}H NMR (400 MHz, $CDCl_3$) δ 9.44 (d, 1 H, J = 7.37 Hz, 1-CH), 7.31-7.26 (m, 5 H, a', b', c' and d'-aromatic), 6.81-6.74 (m, 1 H, 3-CH), 6.35 (s, 2 H, b-aromatic), 6.11 (dd, 1 H, J = 15.7 and 7.82 Hz, 2-CH), 5.15 (s, 2 H, 9-CH_2), 3.89-3.83 (m, 5 H, 5-CH_2 and 7-OMe), 3.78 (s, 6 H, 6-OMe), 2.62-2.57 (m, 2 H, 4-CH_2) ppm; ^{13}C NMR (101 MHz, $CDCl_3$) δ 193.66 (1-C), 155.45 (8-C), 154.51 (3-C), 153.50 (c-aromatic), 137.21 (d-aromatic), 136.84 (a-aromatic), 136.51 (a'-aromatic), 134.62 (2-C), 128.62 (c'-aromatic), 128.26 (d'-aromatic), 127.93 (d'-aromatic), 105.10 (b-aromatic), 67.52 (9-C), 61.01 (7-OMe), 56.27 (6-OMe), 48.89 (5-C), 32.02 (4-C) ppm; HRMS (EI) Exact mass calculated for $C_{22}H_{25}NO_6$ [M+H]+ 400.1760, Found 400.1777 IR (Diamond) υ 2941, 2829, 1686, 1684, 1229 974, 833 cm^{-1}

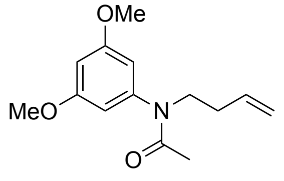

S14

***N*-(but-3-en-1-yl)-*N*-(3,5-dimethoxyphenyl)acetamide S14** : N-(but-3-en-1-yl)-N-(3,5-dimethoxyphenyl)acetamide was isolated in 86 % yield (0.6 g) as a clear oil from the reaction of N-(but-3-en-1-yl)-3,5-dimethoxyaniline (0.56 g, 2.70 mmol) and acetyl chloride (0.21 mL, 2.97 mmol). The crude material was used without further purification.

^{1}H NMR (400 MHz, $CDCl_3$) δ 6.44 (t, 1 H, J = 2.2 Hz, d-aromatic), 6.31 (d, 2 H, J = 2.2 Hz, b-aromatic), 5.77 (ddt, 1 H, J = 10.3, 17.1 and 7.0 Hz, 2-CH), 5.08-5.01 (m, 2 H, 1-CH_2), 3.79-3.72 (m, 8 H, 4-CH_2 and 5-OMe), 2.31-2.25 (m, 2 H, 3-CH_2), 1.87 (s, 3 H, 7-CH_3) ppm; ^{13}C NMR (101 MHz, $CDCl_3$) δ 170.30 (6-C), 161.48 (c-aromatic), 144.82 (a-aromatic), 135.82 (2-C), 116.74 (1-C), 106.72 (b-aromatic), 99.72 (d-aromatic), 55.63 (5-OMe), 48.15 (4-C), 32.43 (3-C), 22.77 (7-C) ppm;

HRMS (EI) Exact mass calculated for $C_{14}H_{19}NO_3$ [M+H]+ 250.1443, Found 250.1452 IR (Diamond) υ 2982, 2973, 2924, 2869, 2844, 1651, 1635,1247, 1153, 985, 833 cm^{-1}

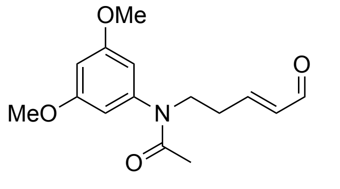

7h

(*E*)-*N*-(3,5-dimethoxyphenyl)-*N*-(5-oxopent-3-en-1-yl)acetamide 7h : Following method C, (N-(but-3-en-1-yl)-N-(3,5-dimethoxyphenyl)acetamide (0.58 g, 2.32 mmol) was coupled with crotonaldehyde (1.48 mL, 23.24 mmol) using Grubbs 2nd Generation catalyst (0.059 g, 0.07 mmol). The reaction yielded 0.5 g (80 %) of (E)-N-(3,5-dimethoxyphenyl)-N-(5-oxopent-3-en-1-yl)acetamide as brown oil after automated column chromatography (gradient, 100% Pent-9:1 Pent/Et_2O).

^1H NMR (400 MHz, $CDCl_3$) δ 9.48 (d, 2 H, J = 7.7 Hz, 1-CH), 6.80 (dt, 1 H, J = 15.8 and 6.9 Hz, 3-CH), 6.44 (t, 1 H, J = 2.2 Hz, d-aromatic), 6.27 (d, 2 H, J = 2.2 Hz, b-aromatic), 6.11 (ddt, 1 H, J = 15.7, 7.9 and 1.3 hz, 2-CH), 3.87 (t, 2 H, J = 7.1 Hz, 5-CH_2), 3.79 (s, 3 H, 6-OMe), 2.58 (qd, 2 H, J = 7.1 and 1.4 Hz, 4-CH_2), 1.88 (s, 3 H, 8-CH_3) ppm; ^{13}C NMR (101 MHz, $CDCl_3$) δ 193.88 (1-C), 170.58 (7-C), 161.68 (c-aromatic), 154.96 (3-C), 144.27 (a-aromatic), 134.37 (2-C), 106.55 (b-aromatic), 99.79 (d-aromatic), 55.65 (6-OMe), 47.05 (5-C), 31.63 (4-C), 22.64 (8-C) ppm; HRMS (EI) Exact mass calculated for $C_{15}H_{19}NO_4$ [M+H]+ 278.1392, Found 278.1398 IR (Diamond) υ 3101, 3067, 2943, 2810, 2727, 1684, 1639, 1635, 1165, 1138, 980, 961, 847 cm^{-1}

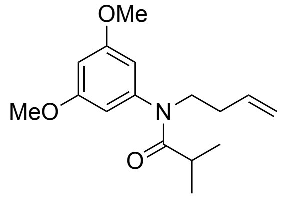

S15

***N*-(but-3-en-1-yl)-*N*-(3,5-dimethoxyphenyl)isobutyramide S15** : Following method B, N-(but-3-en-1-yl)-N-(3,5-dimethoxyphenyl)isobutyramide was isolated in 81 % yield (0.6 g) as a clear oil from the reaction of N-(but-3-en-1-yl)-3,5-dimethoxyaniline (0.0.52 g, 2.50 mmol) and isobutyryl chloride (0.29 mL, 2.75 mmol). The crude material was used purified using silica gel automated column chromatography (gradient, 100 % hex-85:15 hex/EtOAc).

^1H NMR (400 MHz, $CDCl_3$) δ 6.44 (t, 1 H, J = 2.3 Hz, d-aromatic), 6.31 (d, 2 H, J = 2.3 Hz, b-aromatic), 5.76 (ddt, 1 H, J = 17.2, 10.1 and 6.8 Hz, 2-CH), 5.07-5.00 (m, 2 H, 1-CH_2), 3.79 (s, 6 H, 5-OMe), 3.73-3.69 (m, 2 H, 4-CH_2), 2.55-2.45 (sept, 1 H, J = 6.6 Hz, 7-CH), 2.31-2.25 (m, 2 H, 3-CH_2), 1.02 (d, 6 H, J = 6.7 Hz, 8-CH_3) ppm; ^{13}C NMR (101 MHz, $CDCl_3$) δ 171.19 (6-C), 161.45 (c-aromatic), 144.56 (a-aromatic), 135.69 (2-C), 116.60 (1-C), 106.81 (b-aromatic), 99.63 (d-aromatic), 55.62 (5-OMe), 48.31 (4-C), 32.49 (3-C), 31.52 (7-C), 20.04 (8-C) ppm; HRMS (ES-Tof) Exact mass calculated for $C_{16}H_{23}NO_3$ [M+H]+ 278.1756, Found 278.1760 IR (Diamond) υ 3006, 2978, 2939, 2843, 1643, 1591, 1550, 1231, 916, 840 cm^{-1}

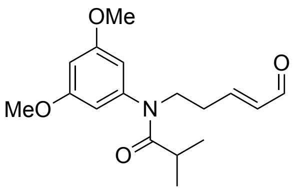

7i

(*E*)-*N*-(3,5-dimethoxyphenyl)-*N*-(5-oxopent-3-en-1-yl)isobutyramide 7h : Following method C, N-(but-3-en-1-yl)-N-(3,5-dimethoxyphenyl)isobutyramide (0.58 g, 2.01 mmol) was coupled with crotonaldehyde (1.28 mL, 20.01 mmol) using Grubbs 2nd Generation catalyst (0.051 g, 0.06 mmol). The reaction yielded 0.5 g (75 %) of (E)-N-(3,5-dimethoxyphenyl)-N-(5-oxopent-3-en-1-yl)isobutyramide as a dark orange oil after automated column chromatography (gradient, 100% Pent-65:35 Pent/Et_2O).

m.p: 63.2-64.4 °C ^1H NMR (400 MHz, $CDCl_3$) δ 9.49 (d, 1 H, J = 7.9 Hz, 1-CH), 6.82 (dt, 1 H, J = 15.7 and 6.9 Hz, 3-CH), 6.46 (t, 1 H, J = 2.2 Hz, d-aromatic), 6.28 (d, 2 H, J = 2.3 Hz, b-aromatic), 6.13 (ddt, 1 H, J = 15.7, 7.8 and 1.3 Hz, 2-CH), 3.86 (t, 2 H, J = 7.0 Hz, 5-CH_2), 3.80 (s, 6 H, 6-OMe), 2.62-2.49 (m, 3 H, 4-CH_2 and 8-CH), 1.02 (d, 6 H, J = 6.8 Hz, 9-CH_3) ppm; ^{13}C NMR (101 MHz, $CDCl_3$) δ 193.85 (1-C), 177.52 (7-C), 161.63 (c-aromatic), 155.15 (3-C), 143.98 (a-aromatic), 134.28 (2-C), 106.62 (b-aromatic), 99.70 (d-aromatic), 55.62 (6-OMe), 47.19 (5-C), 31.76 (4-C), 31.43 (8-C), 19.97 (9-C) ppm; IR (Diamond) υ 3000, 2983, 2966, 2843, 1686, 1683, 990, 928, 859 cm^{-1} HRMS (Es-Tof) Exact mass calculated for $C_{17}H_{23}NO_4$ [M+H]+ 306.1705, Found 306.1702

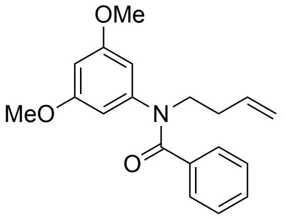

S16

***N*-(but-3-en-1-yl)-*N*-(3,5-dimethoxyphenyl)benzamide S16** : Following method B, N-(but-3-en-1-yl)-N-(3,5-dimethoxyphenyl)benzamide was isolated in 84 % yield (0.7 g) as a clear oil from the reaction of N-(but-3-en-1-yl)-3,5-dimethoxyaniline (0.52 g, 2.50 mmol) and benzoyl chloride (0.35 mL, 2.75 mmol). The crude material was used purified using automated column chromatography (gradient, 100 % hex-8:2 hex/EtOAc).

^1H NMR (400 MHz, $CDCl_3$) δ 7.35-7.33 (m, 2 H, c'-aromatic), 7.27-7.23 (m, 1 H, d'-aromatic), 7.20-7.16 (m, 2 H, b'-aromatic), 6.25 (t, 1 H, J = 2.3 Hz, d-aromatic), 6.18 (d, 2 H, J = 2.2 Hz, b-aromatic), 5.86 (ddt, 1 H, J = 17.1, 10.2 and 6.7 Hz, 2-CH), 5.13-5.06 (m, 2 H, 1-CH_2), 4.00-3.96 (m, 2 H, 4-CH_2), 3.64 (s, 6 H, 5-OMe), 2.45-2.40 (m, 2 H, 3-CH_2) ppm; ^{13}C NMR (101 MHz, $CDCl_3$) δ 170.48 (6-C), 160.92 (c-aromatic), 145.17 (a-aromatic), 136.58 (a'-aromatic), 135.53 (2-C), 129.68 (d'-aromatic), 128.43 (c'-aromatic), 127.86 (b'-aromatic), 116.92 (1-C), 106.54 (b-aromatic), 98.75 (d-aromatic), 55.50 (5-

OMe), 49.67 (4-C), 32.33 (3-C) ppm; HRMS (EI) Exact mass calculated for $C_{19}H_{21}NO_3$ [M+H]+ 312.1600, Found 312.1610 IR (Diamond) υ 3083, 3061, 3013, 2973, 2944, 2842, 1636, 1633, 1593, 1202, 916, 822 cm^{-1}

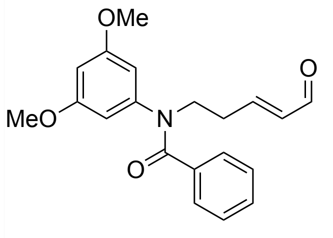

7j

(*E*)-*N*-(3,5-dimethoxyphenyl)-*N*-(5-oxopent-3-en-1-yl)benzamide 7j : Following method C, N-(but-3-en-1-yl)-N-(3,5-dimethoxyphenyl)benzamide (0.65 g, 2.09 mmol) was coupled with crotonaldehyde (1.32 mL, 20.09 mmol) using Grubbs 2nd Generation catalyst (0.053 g, 0.06 mmol). The reaction yielded 0.4 g (63 %) of (E)-N-(3,5-dimethoxyphenyl)-N-(5-oxopent-3-en-1-yl)benzamide as brown oil after automated column chromatography (gradient, 100% Pent-3:2 Pent/Et_2O).

^1H NMR (400 MHz, $CDCl_3$) δ 9.51 (d. 1 H, J = 7.9 Hz, 1-CH), 7.33-7.31 (m, 2 H, b'-aromatic), 7.28-7.25 (m, 2 H, d'-aromatic), 7.21-7.17 (m, 2 H, c'-aromatic), 6.89 (dt, 1 H, J = 15.7 and 7.0 Hz, 3-CH), 6.26 (t, 1 H, J = 2.2 Hz, d-aromatic), 6.17-6.11 (m, 3 H, 2-CH and b-aromatic), 4.12 (t, 2 H, J = 7.0 Hz, 5-CH_2), 3.63 (s, 6 H, 6-OMe), 2.70 (qd, 2 H, J = 7.1 and 1.2 Hz, 4-CH_2) ppm; ^{13}C NMR (101 MHz, $CDCl_3$) δ 193.82 (1-C), 170.63 (7-C), 161.10 (c-aromatic), 154.88 (3-C), 144.57 (a-aromatic), 135.93 (a'-aromatic), 134.93 (2-C), 130.00 (d'-aromatic), 128.45 (b'-aromatic). 127.94 (c'-aromatic), 106.40 (b-aromatic), 98.85 (d-aromatic), 55.51 (6-OMe), 48.38 (5-C), 31.48 (4-C) ppm; HRMS (Es-Tof) Exact mass calculated for $C_{20}H_{21}NO_4$ [M+H]+ 340.1549, Found 340.1559 IR (Diamond) υ 3060, 2943, 2845, 1686, 1684, 975, 832 cm^{-1}

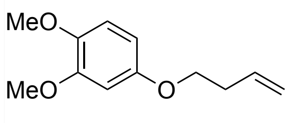

S17

4-(but-3-en-1-yloxy)-1,2-dimethoxybenzene S17 : Following method A, 3,4-dimethyphenol (1.54 g, 10.00 mmol) was reacted with 4-bromo-1-butene (1.52 mL, 15.00 mmol). The crude mixture was purified using silica gel on column chromatography (Hex/Et2O, 8:2) to yield 0.64 g (31 %) of 4-(but-3-en-1-yloxy)-1,2-dimethoxybenzene as a clear oil.

^1H NMR (400 MHz, $CDCl_3$) δ 6.78 (d, 1 H, J = 8.8 Hz, c-aromatic), 6.53 (d, 1 H, J = 2.7 Hz, f-aromatic), 6.39 (dd, 1 H, J = 8.8 and 2.7 Hz, b-aromatic), 5.90 (ddt, 1 H, J = 17.1, 10.1 and 6.7 Hz, 2-CH), 5.20-5.14 (m, 1 H, 1''-CH), 5.12-5.09 (m, 1 H, 1'-CH), 3.97 (t, 2 H, J = 6.6 Hz, 4-CH2), 3.85 (s, 3 H, 6-OMe), 3.83 (5-OMe), 2.55-2.50 (m, 2 H, 3-CH2) ppm; 13C NMR (101 MHz, $CDCl_3$) δ 153.6 (e-aromatic), 149.9 (d-aromatic), 143.6 (a-aromatic), 134.7 (2-C), 117.1 (1-C), 111.9 (c-aromatic), 103.9 (b-aromatic), 101.1 (f-aromatic), 67.8 (4-C), 56.5 (5-OMe), 55.9 (6-OMe), 33.9 (3-C) ppm.

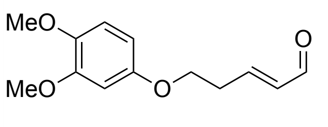

7k

(*E*)-5-(3,4-dimethoxyphenoxy)pent-2-enal 7k : Following method C, 4-(but-3-en-1-yloxy)-1,2-dimethoxybenzene (0.64 g, 3.07 mmol) was coupled with crotonaldehyde (2.54 mL, 30.71 mmol) using Grubbs 2nd Generation catalyst (0.078 g, 0.092 mmol). The reaction yielded 0.60 g (83 %) of benzyl (E)-(3,5-dimethoxyphenyl)(5-oxopent-3-en-1-yl)carbamate as a pale brown oil after automated column chromatography (gradient, 100% Pent-6:4 Pent/Et_2O).

^1H NMR (400 MHz, $CDCl_3$) δ 9.55 (d, 1 H, J = 7.9 Hz, 1-CH), 6.95 (dt, 1 H, J = 15.7 and 6.7 Hz, 3-CH), 6.77 (d, 1 H, J = 8.6 Hz, c-aromatic), 6.51 (d, 1 H, J = 2.8 Hz, f-aromatic), 6.39 (dd, 1 H, J = 8.9 and 2.9 Hz, b-aromatic), 6.24 (ddt, 1 H, J = 15.7, 7.8 and 1.5 Hz, 2-CH), 4.09 (t, 2 H, J = 6.6 Hz, 5-CH2), 3.85 (s, 3 H, 7-OMe), 3.83 (s, 3 H, 6-OMe), 2.80 (m, 2 H, 4-CH2) ppm; 13C NMR (101 MHz, $CDCl_3$) δ 193.9 (1-C), 154.2 (3-C), 153.1 (a-aromatic), 150.0 (e-aromatic), 144.0 (d-aromatic), 134.6 (2-C), 111.8 (c-aromatic), 103.8 (b-aromatic), 101.1 (f-aromatic), 66.2 (5-C), 56.5 (7-OMe), 56.0 (6-OMe), 32.7 (4-C) ppm; IR (Diamond) υ 3006, 2983, 2958, 2866, 2844, 1740, 1684, 1596, 1509, 1507, 1261, 1226, 1034, 994, 991, 832 cm^{-1} HRMS (ES-Tof) Exact mass calculated for $C_{13}H_{16}O_4$ $[M+H]^+$ 237.1127, Found 237.1133

3. General method for the generation of racemic products

To a solution of α,β-unsaturated aldehyde (1 equiv) in THF (0.2 M) was added *p*-TSA *(*0.5 equiv). The mixture was stirred at room temperature until consumption of starting material Once the reaction was complete, the reaction was diluted with ethyl acetate and washed with sodium bicarbonate (aq) (x2). The solution was cooled to 0 °C and diluted with methanol (0.2 M). Sodium borohydride (1.5 equiv) was added to the reaction and the reaction stirred for 1 h at 0 °C. Once all of the cyclized starting material had been consumed, the reaction was quenched with saturated ammonium chloride (aq). The layers were separated and the aqueous extracted with ethyl acetate. The organic layers were combined, washed with brine and dried over $MgSO_4$.

See experimental within main manuscript for details of the organocatalysed reactions.

4. HPLC Traces for Cyclised and Reduced Products

HPLC traces are shown in the order of enantiopure, racemic and spiked samples. The temperature of the HPLC wasn't controlled therefore leading to some fluctuations in retention time between the three traces.

8a	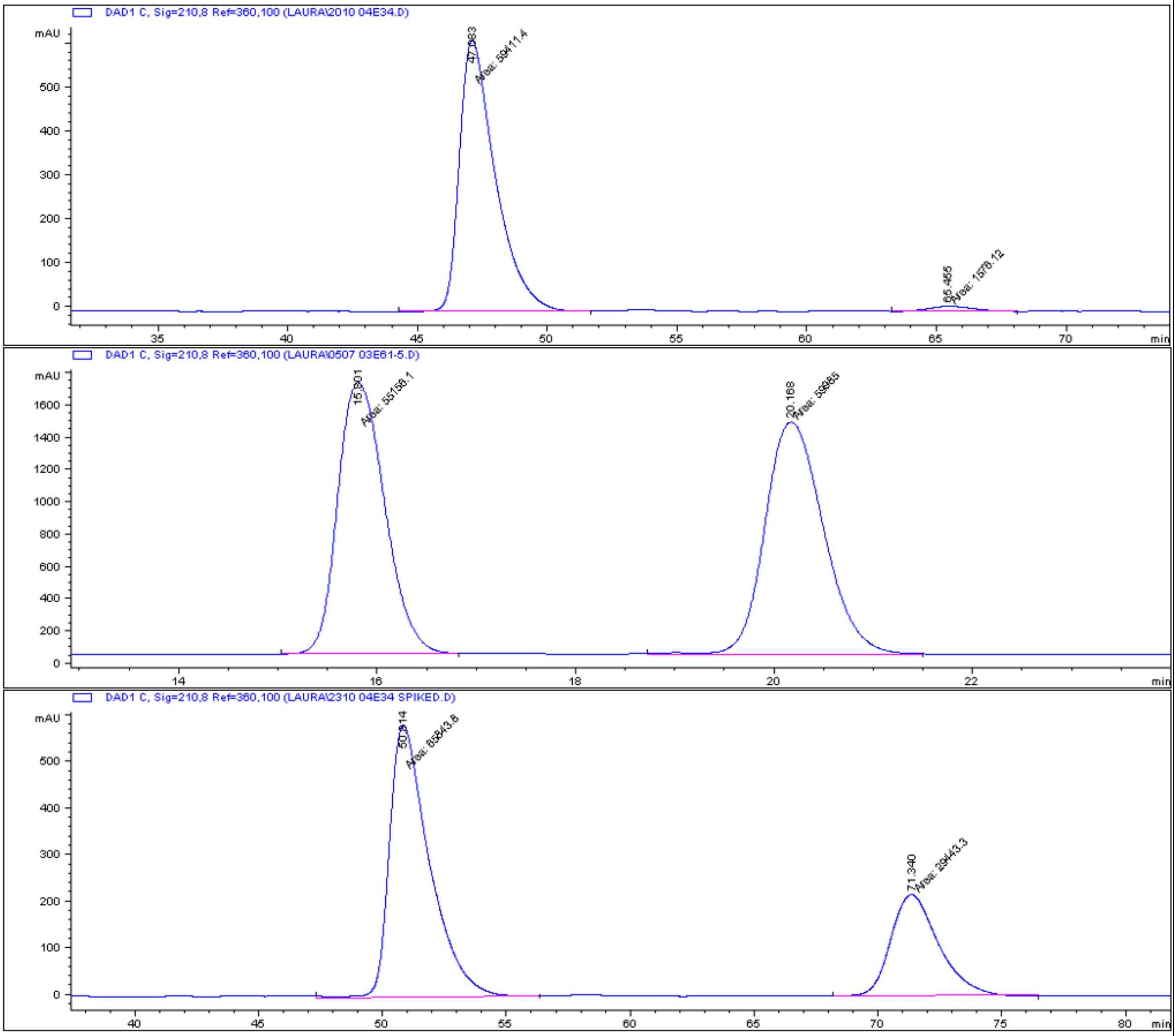

8b

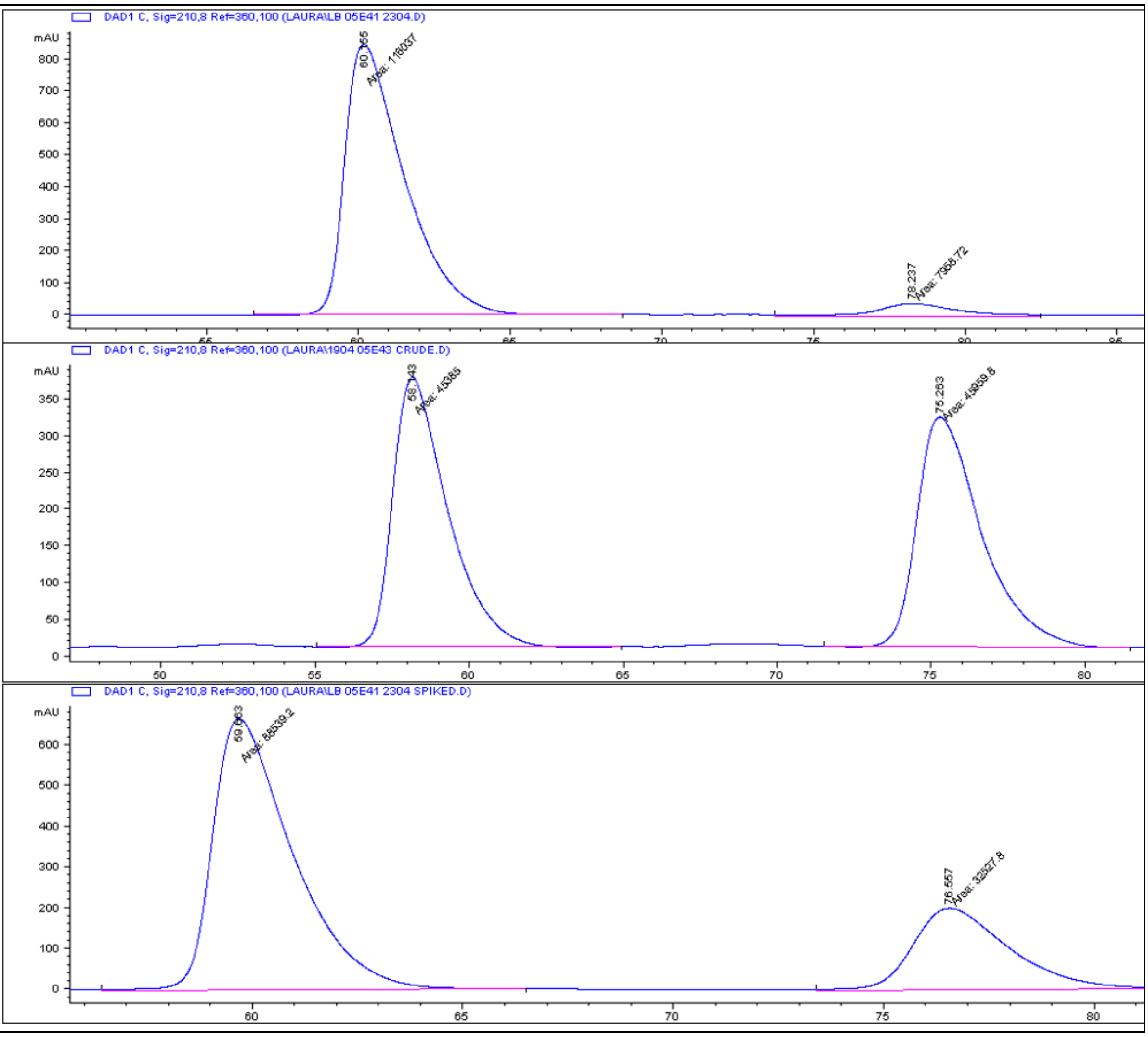

DAD1 C, Sig=210,8 Ref=360,100 (LAURA\LB 05E41 2304.D)
mAU
78.237
Area: 7958.72
DAD1 C, Sig=210,8 Ref=360,100 (LAURA\1904 05E43 CRUDE.D)
mAU
Area: 45385
75.263
Area: 45959.8
DAD1 C, Sig=210,8 Ref=360,100 (LAURA\LB 05E41 2304 SPIKED.D)
mAU
Area: 88539.2
76.557
Area: 32527.8

8c	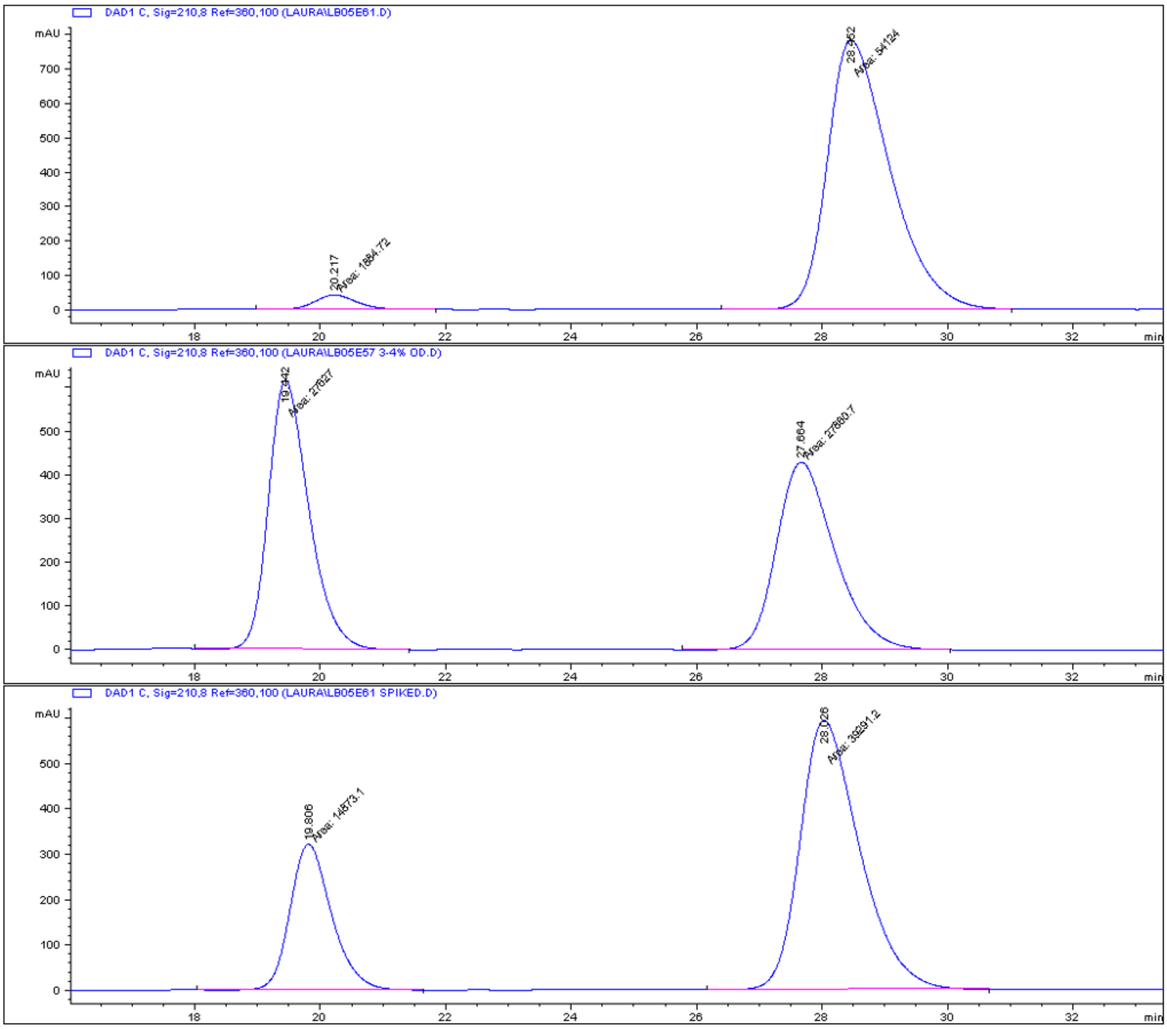

DAD1 C, Sig=210,8 Ref=360,100 (LAURA\LB05E61.D)
DAD1 C, Sig=210,8 Ref=360,100 (LAURA\LB05E57 3-4% OD.D)
DAD1 C, Sig=210,8 Ref=360,100 (LAURA\LB05E61 SPIKED.D)
mAU
min

8d	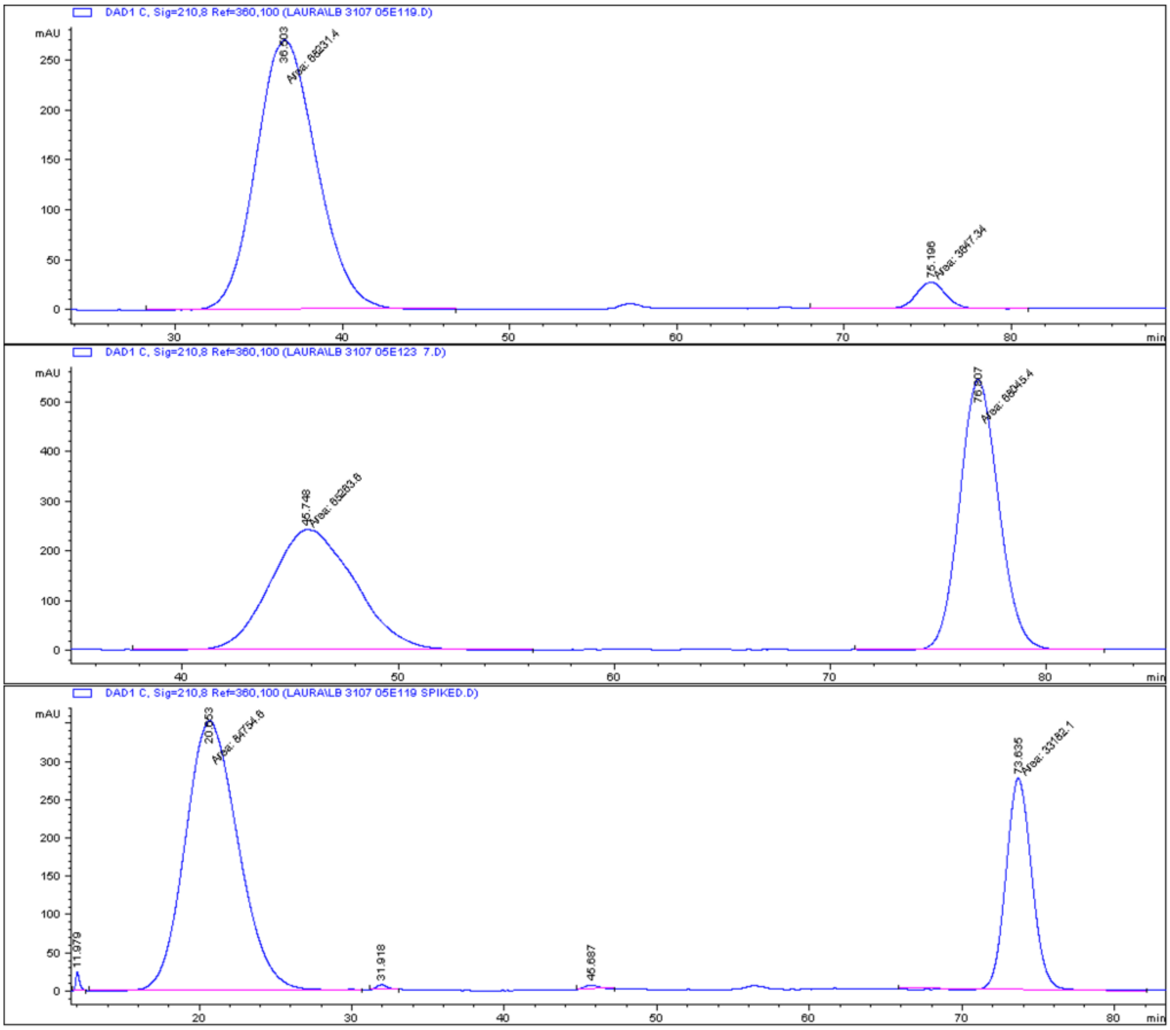

DAD1 C, Sig=210,8 Ref=360,100 (LAURA\LB 3107 05E119.D)
mAU
75.196
Area: 3647.34
min
DAD1 C, Sig=210,8 Ref=360,100 (LAURA\LB 3107 05E123 7.D)
mAU
45.748
min
DAD1 C, Sig=210,8 Ref=360,100 (LAURA\LB 3107 05E119 SPIKED.D)
mAU
11.979
31.918
45.687
73.635
Area: 33182.1
min

8e

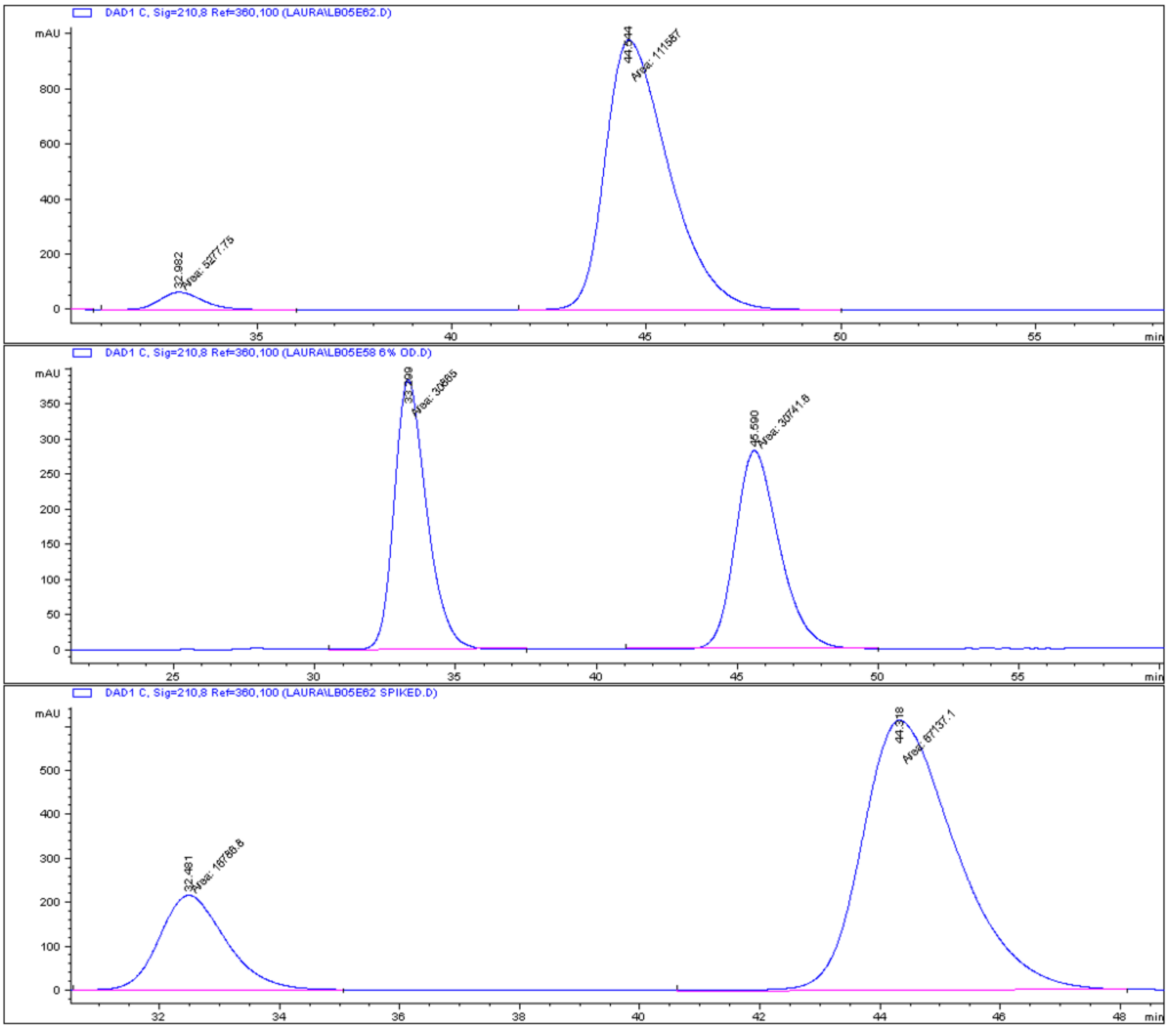
DAD1 C, Sig=210,8 Ref=360,100 (LAURA\LB05E62.D)
mAU
32.982
Area: 5277.75
Area: 111567
min
DAD1 C, Sig=210,8 Ref=360,100 (LAURA\LB05E58 6% OD.D)
mAU
Area: 30665
45.590
Area: 30741.8
min
DAD1 C, Sig=210,8 Ref=360,100 (LAURA\LB05E62 SPIKED.D)
mAU
32.481
44.318
Area: 67137.1
min

8f	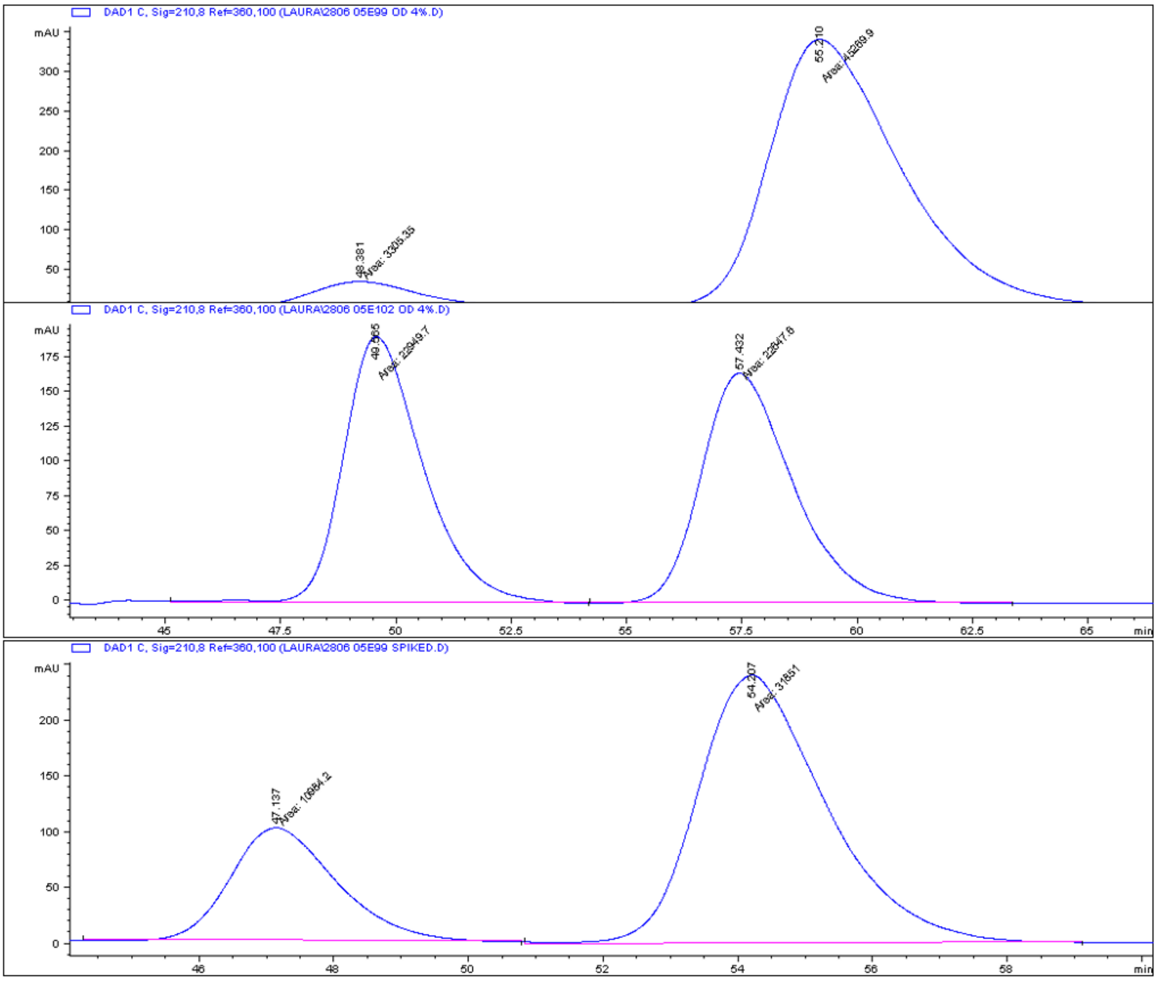

DAD1 C, Sig=210,8 Ref=360,100 (LAURA\2806 05E99 OD 4%.D)
DAD1 C, Sig=210,8 Ref=360,100 (LAURA\2806 05E102 OD 4%.D)
DAD1 C, Sig=210,8 Ref=360,100 (LAURA\2806 05E99 SPIKED.D)
mAU
min

8g	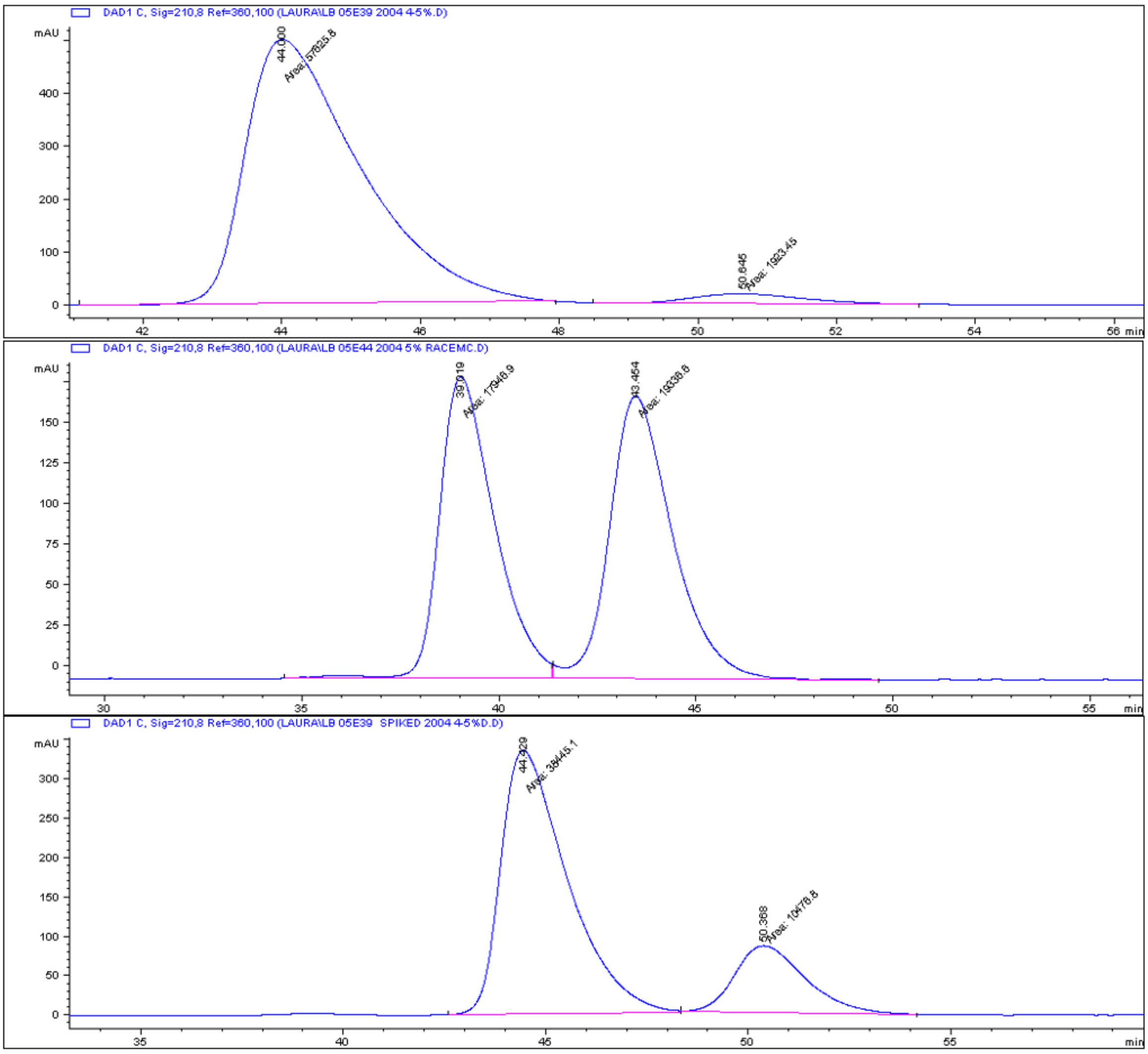DAD1 C, Sig=210,8 Ref=360,100 (LAURA\LB 05E39 2004 4-5%.D)<br>mAU<br>min<br>50.645<br>DAD1 C, Sig=210,8 Ref=360,100 (LAURA\LB 05E44 2004 5% RACEMC.D)<br>mAU<br>min<br>DAD1 C, Sig=210,8 Ref=360,100 (LAURA\LB 05E39 SPIKED 2004 4-5%D.D)<br>mAU<br>min<br>50.368

8h	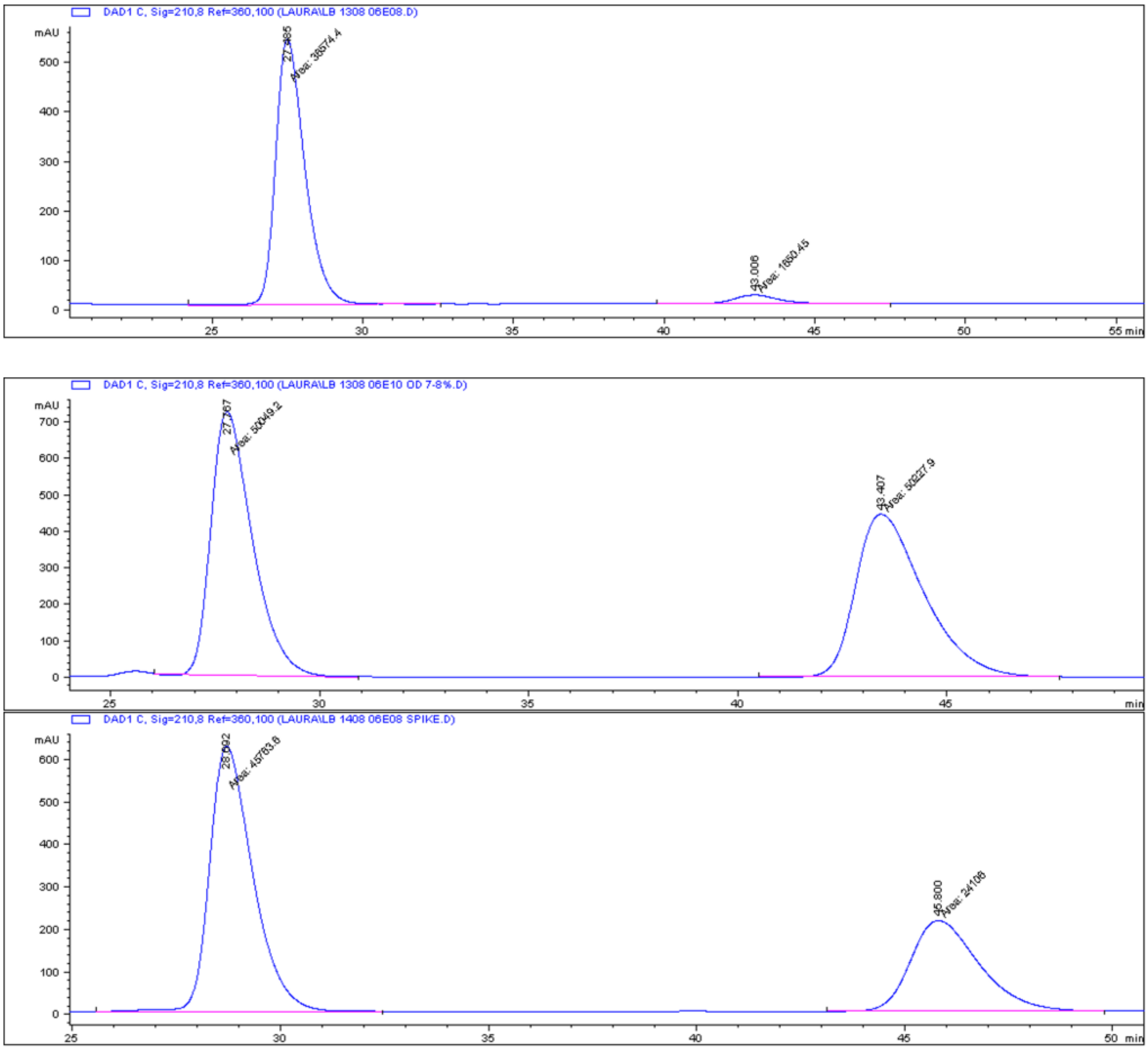DAD1 C, Sig=210,8 Ref=360,100 (LAURA\LB 1308 06E08.D)<br>Area: 38574.4<br>Area: 1650.45<br>DAD1 C, Sig=210,8 Ref=360,100 (LAURA\LB 1308 06E10 OD 7-8%.D)<br>Area: 50049.2<br>43.407<br>Area: 50227.9<br>DAD1 C, Sig=210,8 Ref=360,100 (LAURA\LB 1408 06E08 SPIKE.D)<br>Area: 45763.6<br>Area: 24106<br>mAU<br>min

8i	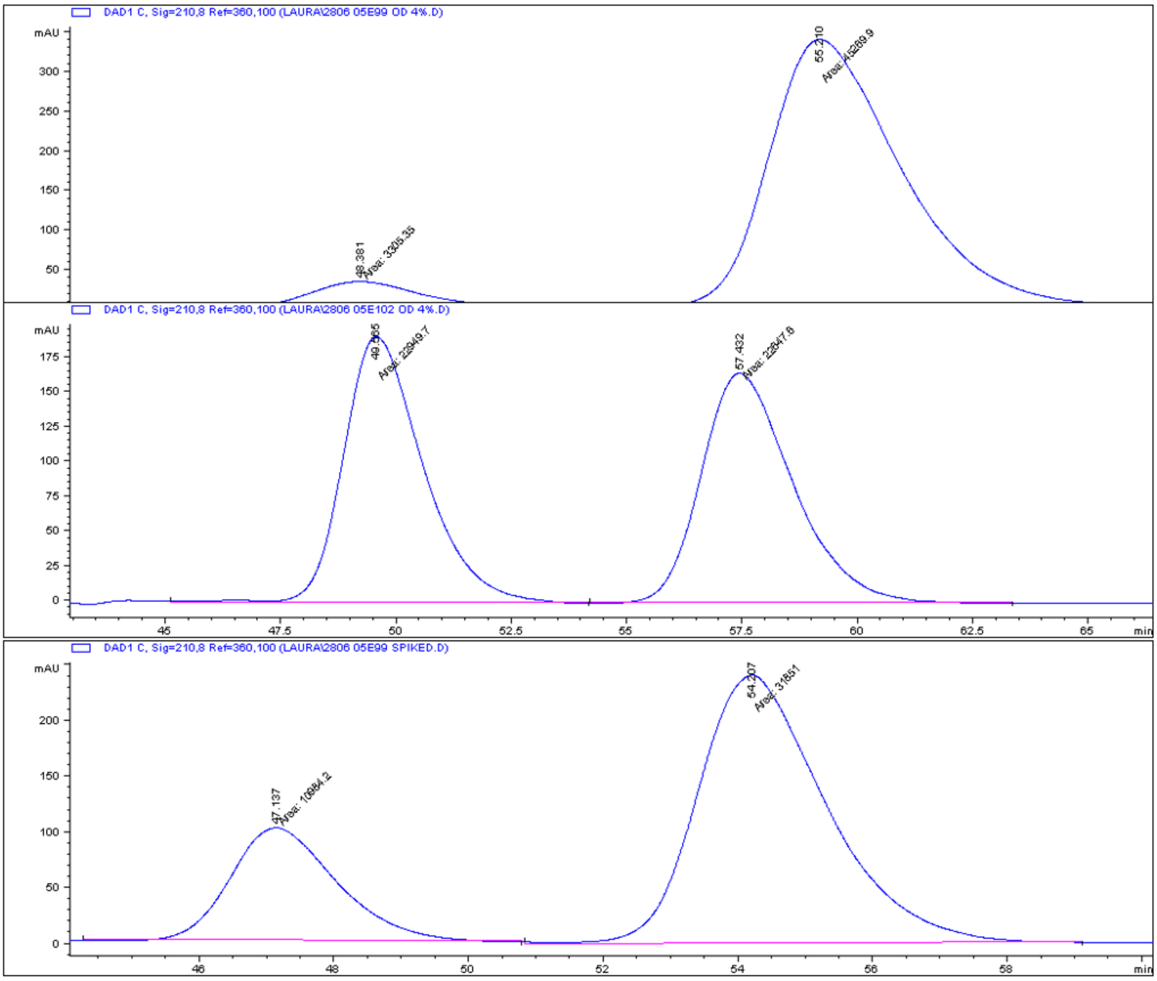

DAD1 C, Sig=210,8 Ref=360,100 (LAURA\2806 05E99 OD 4%.D)
55.210
48.381
DAD1 C, Sig=210,8 Ref=360,100 (LAURA\2806 05E102 OD 4%.D)
57.432
Area: 22647.6
DAD1 C, Sig=210,8 Ref=360,100 (LAURA\2806 05E99 SPIKED.D)
47.137
Area: 31651
mAU
min

8j	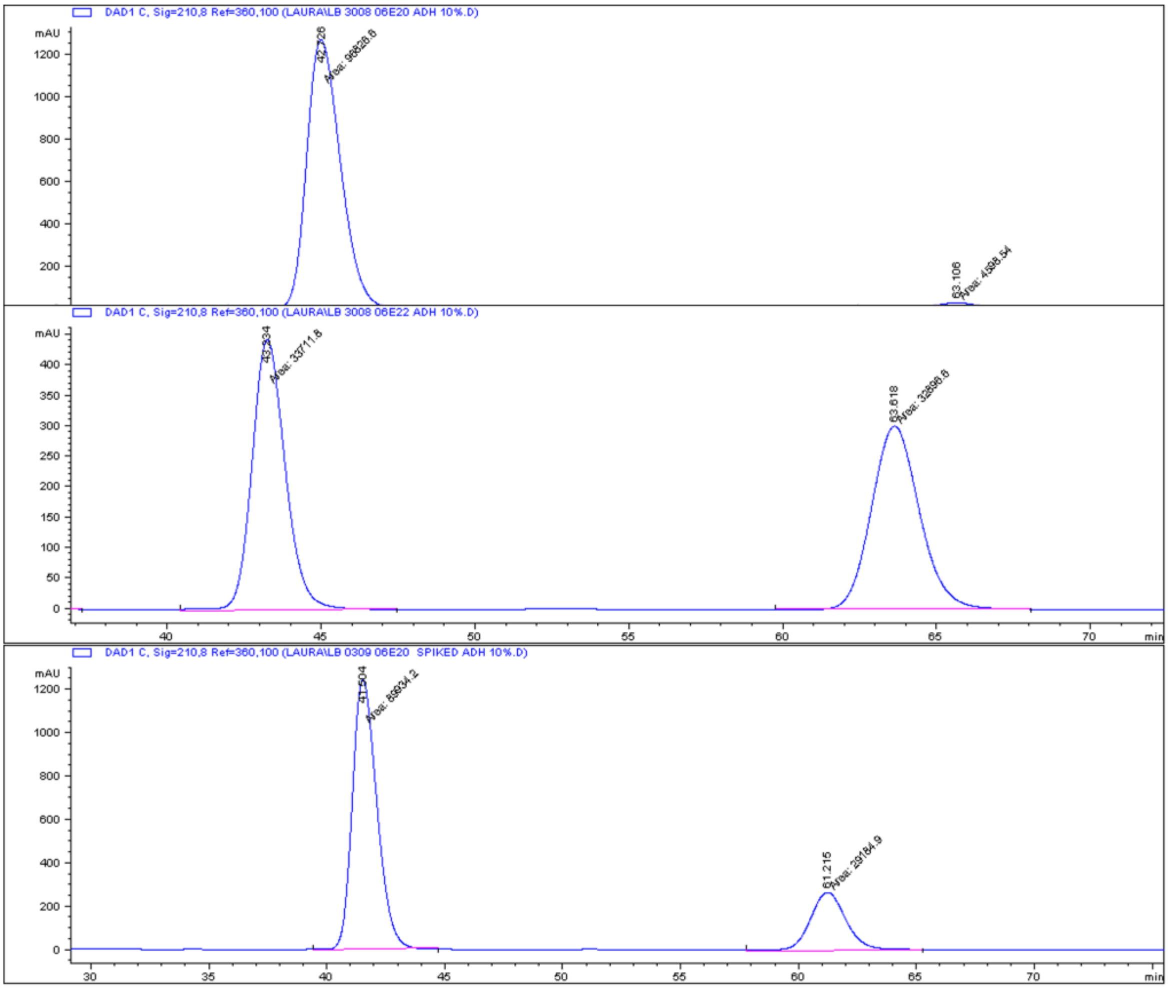

DAD1 C, Sig=210,8 Ref=360,100 (LAURA\LB 3008 06E20 ADH 10%.D)
mAU
DAD1 C, Sig=210,8 Ref=360,100 (LAURA\LB 3008 06E22 ADH 10%.D)
mAU
DAD1 C, Sig=210,8 Ref=360,100 (LAURA\LB 0309 06E20 SPIKED ADH 10%.D)
mAU
min

8k	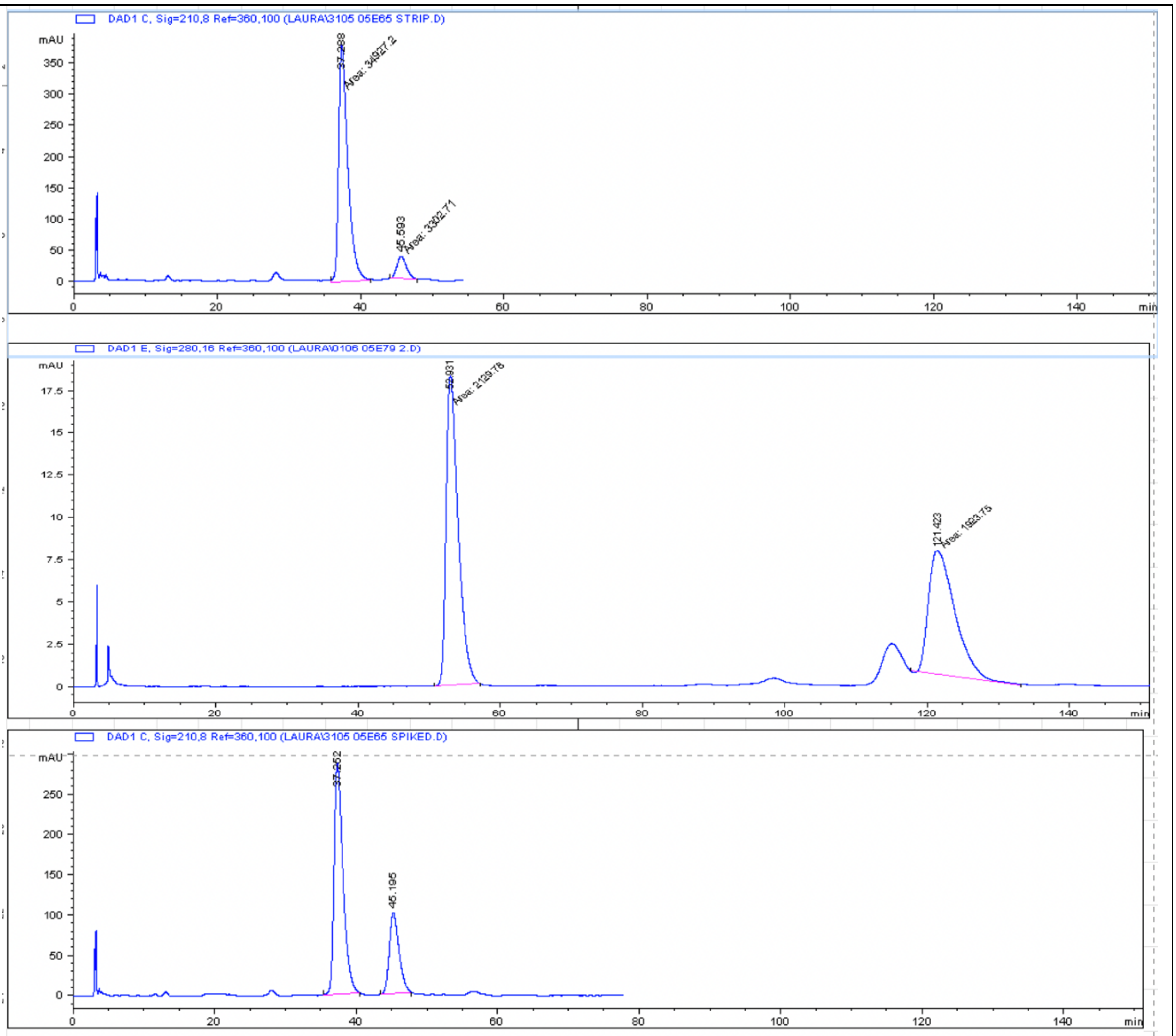

5. Synthesis and characterization of camphorsulfonyl derivative of 8a

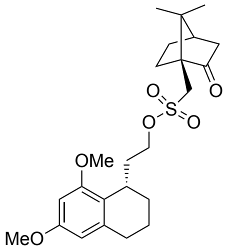

S18

2-((*S*)-6,8-dimethoxy-1,2,3,4-tetrahydronaphthalen-1-yl)ethyl ((1*S*,4*R*)-7,7-dimethyl-2-oxobicyclo[2.2.1]heptan-1-yl)methanesulfonate S18 : To a solution of 2-(6,8-dimethoxy-1,2,3,4-tetrahydronaphthalen-1-yl)ethan-1-ol 8a (0.065 g, 0.28 mmol), dry trimethylamine (0.066 mL, 0.48 mmol) in dry THF (1.4 mL) at 0 °C was added (*S*)- (+)-10-camphorsulfonyyl chloride. The solution was warmed to room temperature and stirred for 24 h. The reaction was quenched with saturated sodium bicarbonate (aq) (2.0 mL) and the layers separated. The aqueous phase was extracted with EtOAc (x3). The combined organic phases were dried over $MgSO_4$.The crude mixture was purified using silica gel on column chromatography (hex/EtOAc, 77.5:22.5) to yield 2-((S)-6,8-dimethoxy-1,2,3,4-tetrahydronaphthalen-1-yl)ethyl (7,7-dimethyl-2-oxobicyclo[2.2.1]heptan-1-yl)methanesulfonate as a clear oil (0.07 g, 57 % yield). The product was recrystallized by dissolving a small portion in DCM and diluting with MeOH.

$[\alpha]_D^{20}$ +9.5 (c 0.2, MeOH)), m.p: 75.4-76.6 °C,^{1}H NMR (400 MHz, $CDCl_3$) δ 6.28 (d, 1 H, J = 2.3 Hz, d-aromatic), 6.21 (d, 1 H, J = 2.3, f-aromatic) 4.44-4.32 (m, 2 H, 1-CH_2), 3.79 (s, 3 H, 7-OMe), 3.77 (s, 3 H, 8-OMe), 3.64 (d, 1 H, J = 15.1 Hz, 9-C**H**H), 3.08-3.03 (m, 1 H, 3-CH), 2.99 (d, 1 H, J = 15.1 Hz, 9-CH**H**), 2.78-2.65 (m, 2 H, 6-CH_2), 2.56-2.49 (m, 1 H), 2.43-2.36 (m, 1 H), 2.13-2.02 (m, 3 H), 1.95 (d, 1 H, J = 18.5 Hz), 1.85-1.63 (m, 6 H), 1.47-1.40 (m, 1 H), 1.13 (s, 3 H, 17-CH_3), 0.89 (s, 3 H, 18-CH_2) ppm; ^{13}C NMR (101 MHz, $CDCl_3$) δ 214.67 (11-C), 158.65 (q-aromatic), 158.29 (q-aromatic), 138.69 (a-aromatic), 121.54 (b-aromatic), 104.54 (f-aromatic), 96.29 (d-aromatic), 70.10 (1-C), 58.12 (q-C), 55.37 (8-OMe), 55.34 (7-OMe), 48.06 (q-C), 46.75 (9-C), 42.94, 42.67, 34.15, 29.89, 28.20 (3-C), 27.04, 26.89, 25.01, 20.02 (17-C), 19.85 (18-C), 18.28 ppm; IR (Diamond) υ 2935, 2838, 1745, 1605, 1394, 1198, 856 cm^{-1} HRMS (ES-Tof) Exact mass calculated for $C_{24}H_{34}SO_6$ [M+H]+ 473.1974, Found 473.1959

Camphorsulfonate derivative **S18** was recrystallized from minimal DCM and hexane. Its single crystal was analysed by X-Ray crystallography using a copper source. The crystal data has not been deposited on CCDC due to the disordered

nature of one of the molecules in the unit cell, but another crystal structure within this manuscript which also confirms the stereochemistry has been.

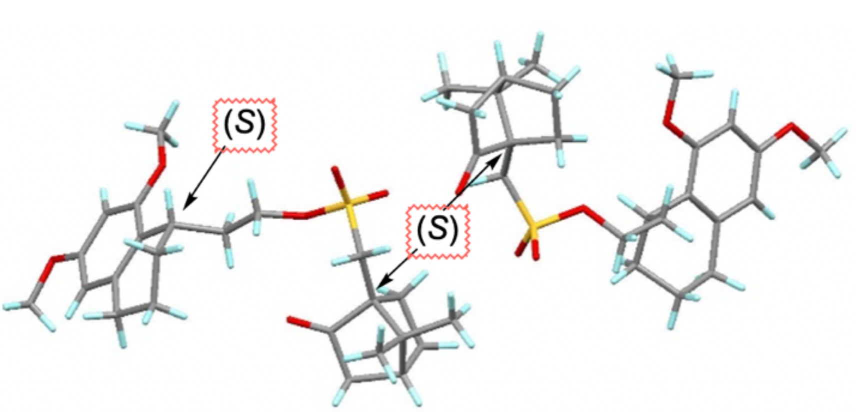

6. Synthesis and characterization of cannabinoid systems

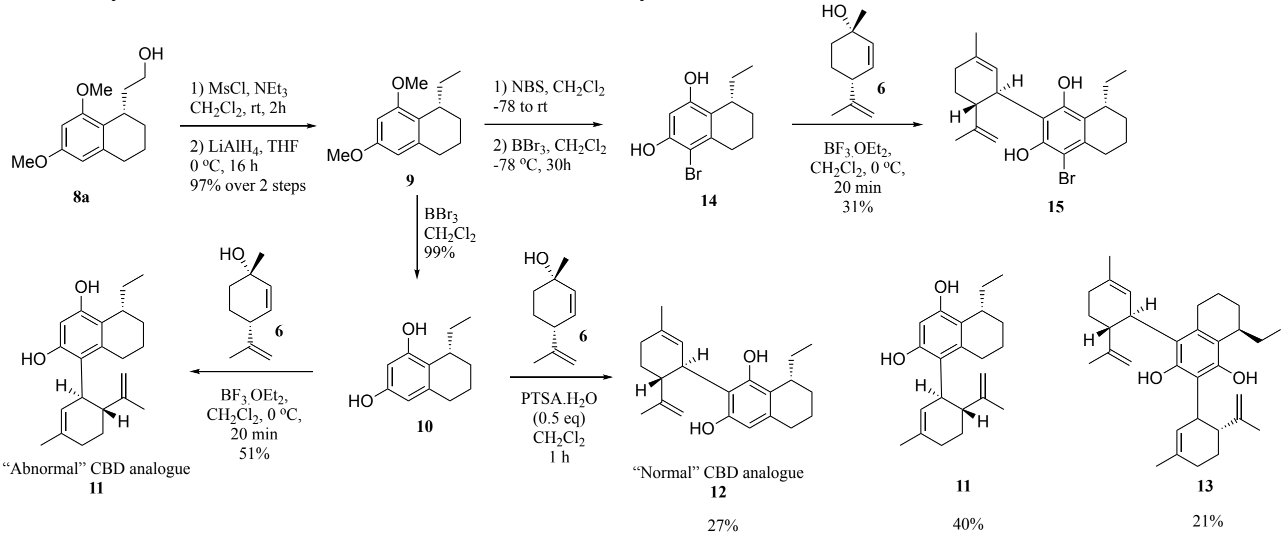

(*R*)-1-ethyl-6,8-dimethoxy-1,2,3,4-tetrahydronaphthalene 9 : To a solution of (*S*)-2-(6,8-dimethoxy-1,2,3,4-tetrahydronaphthalen-1-yl)ethan-1-ol **8a** (1.12 g, 4.76 mmol), dry trimethylamine (2.65 mL, 19.02 mmol) in dry DCM (47.5 mL) was added mesyl chloride (1.41 mL, 18.07 mmol) dropwise. The reaction was quenched with 1 M HCl (50 mL) after 2 h of stirring at room temperature. The layers were separated and the aqueous extracted with EtOAc (x2). The combined organic layers were washed with sodium bicarbonate (aq) and dried over Na_2SO_4. After the removal of solvent under reduced pressure, the crude material was dissolved in dry THF (47.5 mL) and cooled to 0 °C. $LiAlH_4$ (1.19 g, 31.39 mmol) was added to the cooled solution before the temperature was raised to rt. The solution was stirred overnight and quenched at 0 °C with distilled water (1 mL) dropwise, 15 % NaOH (aq) (1 mL) and distilled water (3 mL). The mixture was warmed to room temperature and stirred for 15 min. $MgSO_4$ was added and stirring continued for a further 2 h. The mixture was filtered and solvent removed under reduced pressure to yield 1-ethyl-6,8-dimethoxy-1,2,3,4-tetrahydronaphthalene (1.0 g, 97 % yield) as an orange oil which required no further purification.

$[\alpha]_D^{20}$ +11.0 (c 0.2, MeOH) ^{1}H NMR (400 MHz, $CDCl_3$) δ 6.21 (d, 1 H, J = 2.3 Hz, d-aromatic), 6.13 (d, 1 H, J = 2.3 Hz, f-aromatic), 3.73-3.69 (m, 6 H, 7-OMe and 8-OMe), 2.75-2.68 (m, 1 H, 3-CH), 2.67-2.59 (m, 2 H, 6-CH_2), (m,1 H, 4-C**H**H), 1.76-1.46 (m, 4 H, 2-C**H**H, 4-CH**H**, 5-CH_2), 1.32-1.20 (m, 1 H, 2-CH**H**), 0.87 (t, 3 H, J = 7.4 Hz, 1-CH_3) ppm; ^{13}C NMR (101 MHz, $CDCl_3$) δ 158.52 (c-aromatic), 158.20 (e-aromatic), 138.49 (a-aromatic), 123.70 (b-aromatic), 104.35 (f-aromatic), 96.30 (d-aromatic), 55.34 (7-OMe), 55.33 (8-OMe), 33.27 (3-C), 30.25 (6-C), 27.17 (2-C), 25.36 (4-C), 18.24 (5-C), 12.70 (1-C) ppm; IR (Diamond) υ 2958, 2933, 2869, 1605, 1590, 1486, 1458, 861 cm^{-1} Exact mass calculated for $C_{14}H_{20}O_2$ [M+H]+ 221.1542, Found 221.1550

(*R*)-5-bromo-1-ethyl-6,8-dimethoxy-1,2,3,4-tetrahydronaphthalene **14** : To a solution of (*R*)-1-ethyl-6,8-dimethoxy-1,2,3,4-tetrahydronaphthalene **9** (0.33 g, 1.50 mmol) in anhydrous DCM (35.0 mL) at -78 °C was added N-bromosuccinimide (0.27 g, 1.50 mmol) in one portion. After 1 h of stirring at -78 °C, the temperature of the solution was raised to room temperature. After 2 h the reaction was quenched with saturated sodium bicarbonate solution (aq)

and the organic layer washed with 5 % sodium thiosulphate solution (aq) before being dried over $MgSO_4$. The resultant thick orange oil taken forward without further purification (3.3 g, 72 % yield).

$[\alpha]_D^{20}$ +9.0 (c 0.2, DCM) ^{1}H NMR (400 MHz, $CDCl_3$) δ 6.39 (s, 1 H, d-aromatic), 3.89 (s, 3 H, 8-OMe), 3.83 (s, 3 H, 7-OMe), 2.93-2.82 (m, 2 H, 3-CH and 6-C**H**H), 2.59-2.50 (m, 1 H, 6-CH**H**), 1.94-1.88 (m, 1 H, 4-C**H**H), 1.80-1.73 (m, 2 H, 5-CH_2), 1.65-1.57 (m, 1 H, 2-C**H**H), 1.53-1.44 (m, 1 H, 4-CH**H**), 1.37-1.29 (m, 1 H, 2-CH**H**), 0.85 (t, 3 H, J = 7.4 Hz, 1-CH_3) ppm; ^{13}C NMR (101 MHz, $CDCl_3$) δ 157.17 (c-aromatic), 154.25 (e-aromatic), 137.78 (a-aromatic), 125.71 (b-aromatic), 105.50 (f-aromatic), 94.38 (d-aromatic), 56.60 (8-OMe), 55.64 (7-OMe), 33.49 (3-C), 30.91 (6-C), 26.89 (2-C), 24.42 (4-C), 17.91 (5-C), 12.65 (1-C) ppm; IR (Diamond) υ 2952, 2933, 2870, 2836, 1591, 804 cm^{-1} HRMS (ES-Tof) Exact mass calculated for $C_{14}H_{19}BrO_2$ [M+H]+ 299.0647, Found 299.0645

(*R*)-4-bromo-8-ethyl-5,6,7,8-tetrahydronaphthalene-1,3-diol 15: To a solution of (*R*)-5-bromo-1-ethyl-6,8-dimethoxy-1,2,3,4-tetrahydronaphthalene **14** (0.31 g, 1.05 mmol) in dry DCM (2.8 mL) at -78 °C was added BBr_3 (1.0 M, 2.84 mL, 2.84 mmol) dropwise. The temperature was raised to room temperature and the reaction stirred overnight. After 30 h, the reaction was quenched with ice and the layers separated. The aqueous was extracted with DCM (x3) and EtOAc (x2). The organic layers were combined and dried over $MgSO_4$. The crude material was purified using silica gel on column chromatography (Hex/EtOAc, 7:3) to yield 4-bromo-8-ethyl-5,6,7,8-tetrahydronaphthalene-1,3-diol as a brown crystalline solid (0.13 g, 31% yield).

$[\alpha]_D^{20}$ +32.3 (c 0.2, DCM), m.p: 123.4-124.6 °C ^{1}H NMR (400 MHz, $CDCl_3$) δ 6.40 (s, 1 H, d-aromatic), 5.49 (s, 1 H, e-OH), 4.73 (s, 1 H, c-OH), 2.85-2.78 (m, 1 H, 6-C**H**H), 2.74 (dtd, 1 H, J = 8.0, 5.5 and 3.0 Hz, 3-CH), 2.57-2.48 (m, 1 H, 6-CH), 1.98-1.91 (m, 1 H, 4-C**H**H), 1.84-1.74 (m, 2 H, 5-CH_2), 1.72-1.62 (m, 1 H, 2-C**H**H), 1.55-1.49 (m, 1 H, 4-CH**H**), 1.40 (ddq, 1 H, J = 14.3, 10.43, 7.2 Hz, 2-CH**H**), 0.99 (t, 1 H, J = 7.4 Hz, 1-CH_3) ppm; ^{13}C NMR (101 MHz, $CDCl_3$) δ 153.57 (c-aromatic), 150.46 (e-aromatic), 137.15 (a-aromatic), 123.21 (b-aromatic), 104.66 (f-aromatic), 100.70 (d-aromatic), 33.74 (3-C), 30.77 (6-C), 26.73 (2-C), 24.42 (4-C), 17.74 (5-C), 12.58 (1-C) ppm; IR (Diamond) υ 3464, 3415, 2959, 2935, 2866, 1592, 835, 820 cm^{-1} HRMS (ES-Tof) Exact mass calculated for $C_{12}H_{17}NO_2$ [M+H]+ 269.0177, Found 269.0187

(*R*)-8-ethyl-5,6,7,8-tetrahydronaphthalene-1,3-diol 10 : BBr_3 (1.0 M, 10.12 mL, 10.12 mmol) was added dropwise over 4 mins to a solution of 1-ethyl-6,8-dimethoxy-1,2,3,4-tetrahydronaphthalene (0.86 g, 3.89 mmol) in dry DCM at -78 °C. The reaction temperature was raised to 0 °C after 30 min. After 24 h the temperature of the reaction was raised to room temperature due to incomplete consumption of starting material. After a further 3 h of stirring, the reaction was quenched at 0 °C with MeOH (25 mL). The layers were separated and the aqueous extracted with EtOAc (x3). The combined organic layers were washed with saturated $NaHCO_3$ (aq), distilled water, brine and dried over $MgSO_4$. The crude mixture was purified using silica gel on column chromatography (Hex/EtOAc/DCM, 65:25:10) to yield 8-ethyl-5,6,7,8-tetrahydronaphthalene-1,3-diol as a brown oil (0.74 g, 99 % yield).

^{1}H NMR (400 MHz, $CDCl_3$) δ 6.15 (s, 2 H, d and f-aromatic), 4.83-4.72 (m, 2 H, 7 and 8-OH), 42.71-2.60 (m, 3 H, 3-CH and 6-CH_2), 1.96-1.90 (m, 1 H, 4-C**H**H), 1.83-1.58 (m ,4 H, 2-C**H**H, 4-CH**H**, 5-CH_2), 1.48-1.37 (m, 1 H, 2-CH**H**), 0.99 (t, 3 H, J = 7.6 Hz, 1-CH_3) ppm; ^{13}C NMR (101 MHz, $CDCl_3$) δ 154.50 (c-aromatic), 153.91 (e-aromatic), 139.55 (a-aromatic), 121.12 (b-aromatic), 107.90 (f-aromatic), 100.57 (d-aromatic), 33.48 (3-C), 29.83 (6-C), 27.03 (2-C), 25.44 (4-C), 18.09 (5-C), 12.59 (1-C) ppm; HRMS (Es-Tof) Exact mass calculated for $C_{12}H_{16}O_2$ [M+H]+191.1067, Found 191.1072 IR (Diamond) υ 3478, 3243, 2960, 2937, 2870, 1608, 1590, 839 cm^{-1}

(*R*)-8-ethyl-4-((1*R*,6*R*)-3-methyl-6-(prop-1-en-2-yl)cyclohex-2-en-1-yl)-5,6,7,8-tetrahydronaphthalene-1,3-diol 11 : BF_3OEt_2 METHOD : To a solution of 8-ethyl-5,6,7,8-tetrahydronaphthalene-1,3-diol (0.14 g, 0.70 mmol) and $BF_3.OEt_2$ (0.009 mL, 0.07 mmol) in dry DCM (2.5 mL) at 0 °C was added (1S, 4R)-1-methyl-4-(prop-1-en-2-yl)cyclohex-2-en-1-ol (0.11 g, 0.7 mmol) dropwise in dry DCM (1.0 mL) over 1 min. The reaction was quenched after 20 min with saturated sodium bicarbonate solution (aq) (3.5 mL). The layers were separated and the aqueous was extracted with DCM (x2). The combined organic layers were combined and dried over $MgSO_4$. The crude material was purified using silica gel on column chromatography (the sample was dry loaded, gradient, Hex/ Et_2O, 97.5:2.5, 9:1, 65:35) to yield a brown solid (0.16 g, 51 % yield).

$[\alpha]_D^{20}$ -40.0 (c 0.2, DCM), m.p: 48.0-49.5 °C ^{1}H NMR (400 MHz, $CDCl_3$) δ 6.19 (s, 1 H, 3'-CH), 6.02 (s, 1 H, 2'-OH), 5.57 (s, 1 H, 2-CH), 4.62-4.61 (m, 1 H, 10-CHH), 4.52 (s, 1 H, 4'-OH), 4.44 (s, 1 H, 10-CHH), 3.62-3.59 (m, 1 H, 3-CH), 2.73-2.62 (m, 2 H, 1''-CHH and 4''-CH), 2.50-2.38 (m, 2 H, 1''-CHH and 4-CH), 2.24-2.16 (m, 1 H, 6-CHH), 2.10-2.04 (m, 1 H, 6-CHH), 1.89-1.63 (m, 9 H, 2''-CH_2, 3''-CHH, 5-CH_2, 5''-CHH and 7-CH_3), 1.53-1.35 (m, 5 H, 3''-CHH, 5''-CHH and 9-CH_3), 0.98 (t, 3 H, J = 7.5 Hz, 6''-CH_3) ppm; ^{13}C NMR (101 MHz, $CDCl_3$) δ 153.84 (2'-C), 152.60 (4'-C), 147.99 (8-C), 139.43 (1-C), 137.28 (6'-C), 125.15 (2-C), 120.91 (5'-C), 120.15 (1'-C), 111.41 (10-C), 102.11 (3'-C), 44.96 (4-C), 39.67 (3-C), 33.95 (4''-C), 30.26 (6-C), 28.19 (5-C), 27.74 (1''-C), 27.03 (5''-C), 24.53 (3''-C), 23.76 (7-C), 21.38 (9-C), 18.32 (2''-C), 12.68 (6''-C) ppm; IR (Diamond) υ 3425, 2982, 2958, 2929, 2869, 2832, 1642, 890, 862 cm^{-1} HRMS (Es-Tof) Exact mass calculated for $C_{22}H_{30}O_2$ [M+H]$^+$327.2324, Found 327.232

(*R*)-8-ethyl-2-((1*R*,6*R*)-3-methyl-6-(prop-1-en-2-yl)cyclohex-2-en-1-yl)-5,6,7,8-tetrahydronaphthalene-1,3-diol 13 and **(*R*)-8-ethyl-4-((1*R*,6*R*)-3-methyl-6-(prop-1-en-2-yl)cyclohex-2-en-1-yl)-5,6,7,8-tetrahydronaphthalene-1,3-diol 11 : TsOH METHOD :** To an ice-cold suspension of (*R*)-8-ethyl-5,6,7,8-tetrahydronaphthalene-1,3-diol **10** (0.51 g, 2.66 mmol) and *p*-toluenesulfonic acid monohydrate (253 mg, 1.33 mmol) in anhydrous dichloromethane (16 mL) was added a solution of (1*S*,4*R*)-menthadienol (404 mg, 2.66 mmol) in anhydrous dichloromethane (5 mL) over 1 h and the mixture was stirred for 10 min at 0 °C. The reaction mixture was quenched with saturated aqueous sodium bicarbonate (15 mL) and the layers were separated. The aqueous layer was extracted with dichloromethane (2 × 15 mL) and the combined organic layers were washed with saturated brine (50 mL), dried ($MgSO_4$) and concentrated to give an off-white foam. This material was purified using a Biotage Isolera automated chromatography system under normal phase conditions (silica column, gradient of 0 → 100 % diethyl ether in petrol) with detection at 270 nm to give normal CBD cyclohexyl analogue **12** (486 mg, 27 %), as a white solid and abnormal CBD cyclohexyl analogue **11** (0.72 g, 40 %), as a white solid. Compound **13** is a proposed but un-isolated structure based on the LC-MS profile.

^{1}H NMR (500 MHz, $CDCl_3$) δ 6.28 – 5.81 (2 H, m, 3, 7), 5.60 – 5.51 (1 H, s, 10), 4.79 – 4.40 (3 H, m, 8, 17), 3.91 – 3.79 (1 H, s, 9), 2.78 – 2.54 (3 H, s, 19', 19'', 22), 2.45 – 2.31 (1 H, s, 14), 2.31 – 2.16 (1 H, m, 12'), 2.14 – 2.05 (1 H, d, *J* 15.3, 12''), 1.95 – 1.86 (1 H, d, *J* 14.2, 13''), 1.86 – 1.69 (6 H, s, 13', 15, 20), 1.69 – 1.49 (6 H, m, 18, 21, 23''), 1.46 – 1.29 (1 H, m, 23'), 0.99 – 0.91 (3 H, t, *J* 7.4, 24).; ^{13}C NMR (126 MHz, $CDCl_3$) δ 153.73, 153.45, 151.40, 150.03, 149.43, 140.23, 140.05, 136.40, 124.18, 122.93, 122.79, 120.20, 114.09, 113.72, 110.78, 110.71, 109.45, 107.42, 46.00, 38.38, 37.31, 33.80, 33.56, 30.46, 30.36, 29.33, 28.40, 26.75, 26.62, 25.60, 24.95, 23.80, 22.62, 21.20, 20.56, 20.45, 17.87, 12.61.

^{1}H NMR (500 MHz, d6-DMSO) δ 8.33 (1 H, s br, H-8), 6.91 (1 H, s br, H-7), 5.98 (1 H, s, H-3), 5.21 (1 H, m, H-10), 4.48 (1 H, m, H-17'), 4.43 (1 H, m, H-17''), 3.90 (1 H, m, H-9), 2.87 (1 H, m, H-14), 2.75 (1 H, m, H-22), 2.53 – 2.48* (2 H, m, H-19',19''), 2.16 (1 H, m, H-12'), 1.97 (1 H, m, H-12''), 1.81 (1 H, m, H-21'), 1.76 – 1.46 (11 H, m, H-13',13'', H-15, H-18, H-20',20'', H-21'', H-23'), 1.25 (1 H, m, H-23''), 0.89 (3 H, t, *J* 7.3, H-24). *masked underneath DMSO peak.
^{13}C NMR (126 MHz, d6-DMSO) δ 54.07 (C-2), 153.77 (C-6), 149.18 (C-16), 134.59 (C-4), 132.53 (C-11), 126.87 (C-10), 120.35 (C-5), 115.62 (C-1), 110.26 (C-17), 107.71 (C-3), 44.87 (C-14), 36.60 (C-9), 33.05 (C-22), 30.71 (C-12), 29.74 (C-13), 29.45 (C-19), 27.02 (C-23), 25.75 (C-21), 23.74 (C-15), 19.85 (C-18), 18.25 (C-20), 12.50 (C-24).

7. NMR Spectra

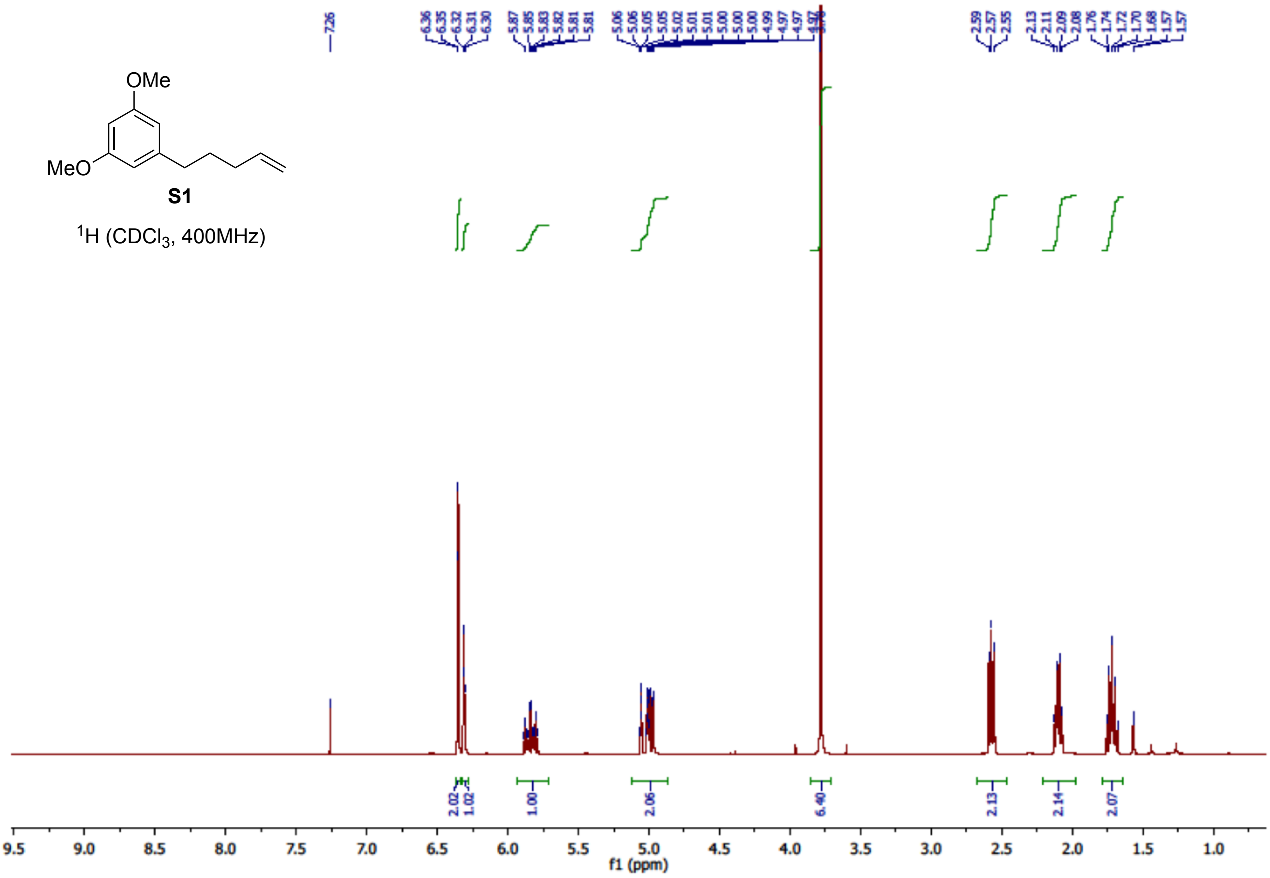

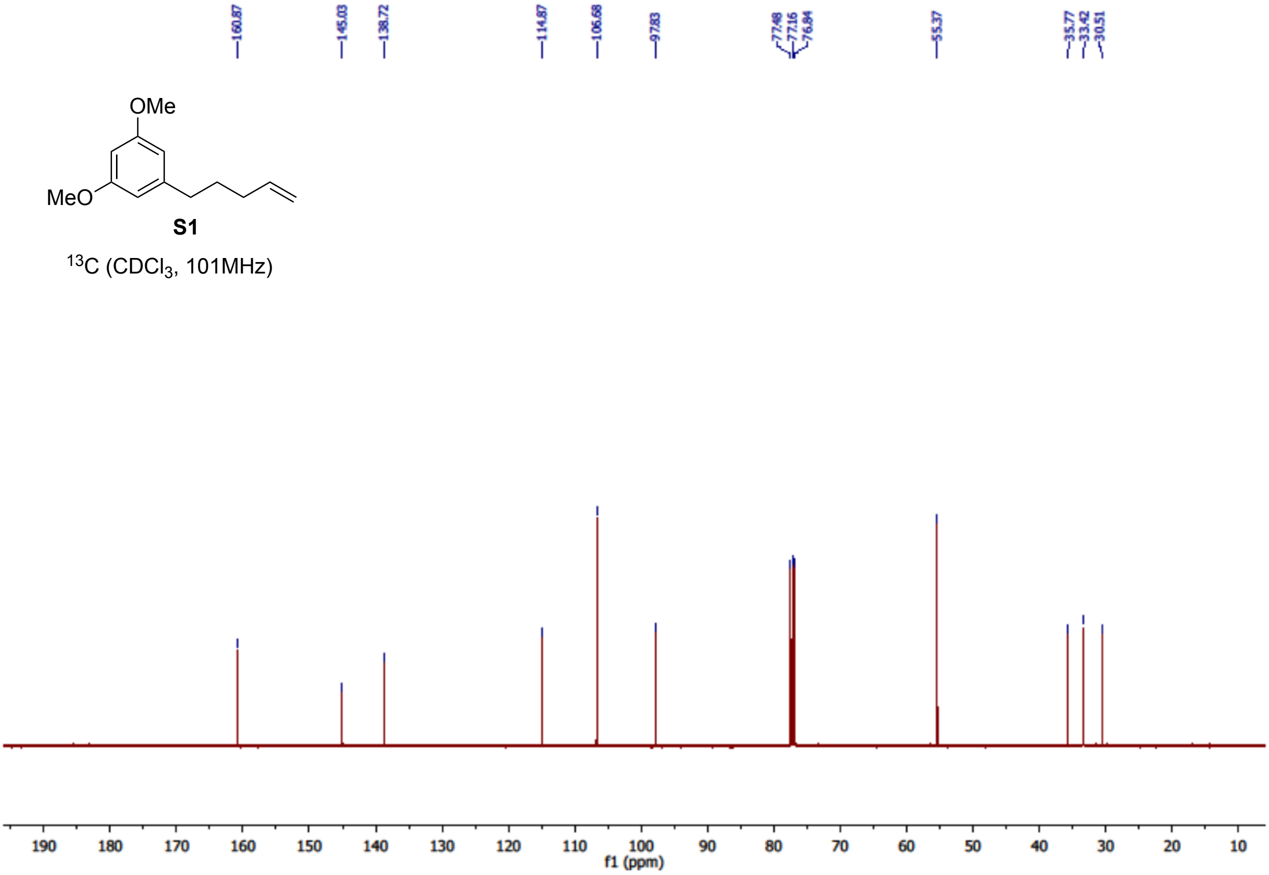

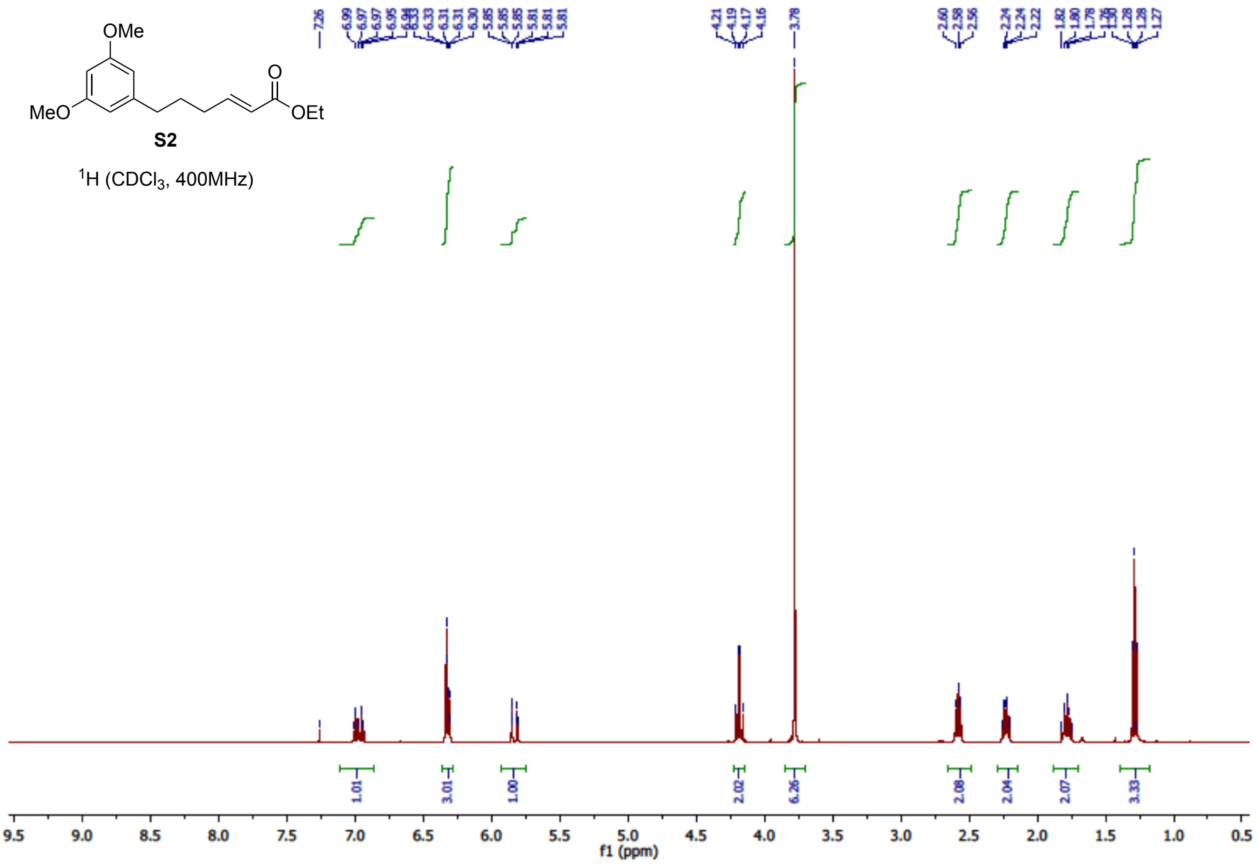
OMe
MeO
O
OEt
S2
1H (CDCl3, 400MHz)
f1 (ppm)

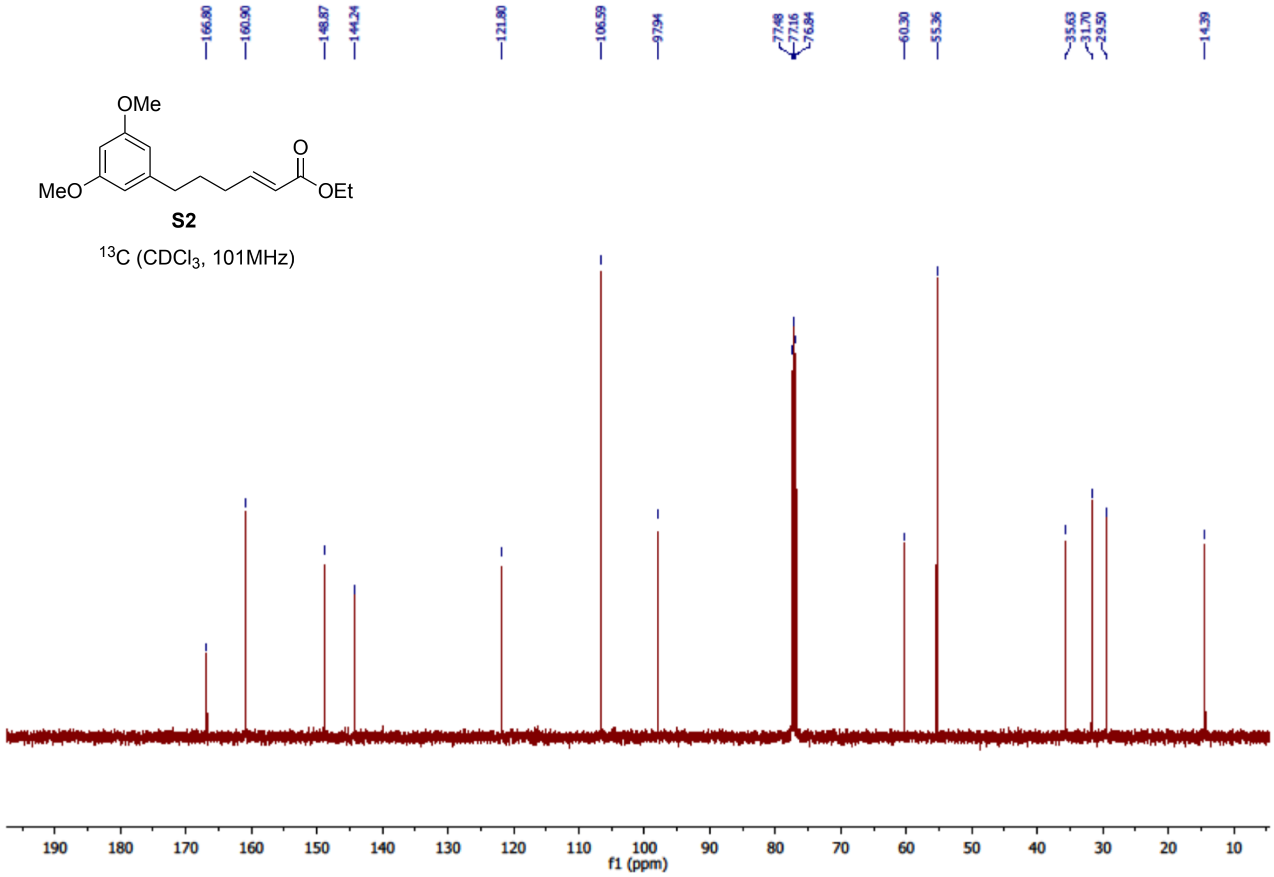
OMe
MeO
O
OEt
S2
13C (CDCl3, 101MHz)
166.80
160.90
148.87
144.24
121.80
106.59
97.94
77.48
77.16
76.84
60.30
55.36
35.63
31.70
29.50
14.39
f1 (ppm)

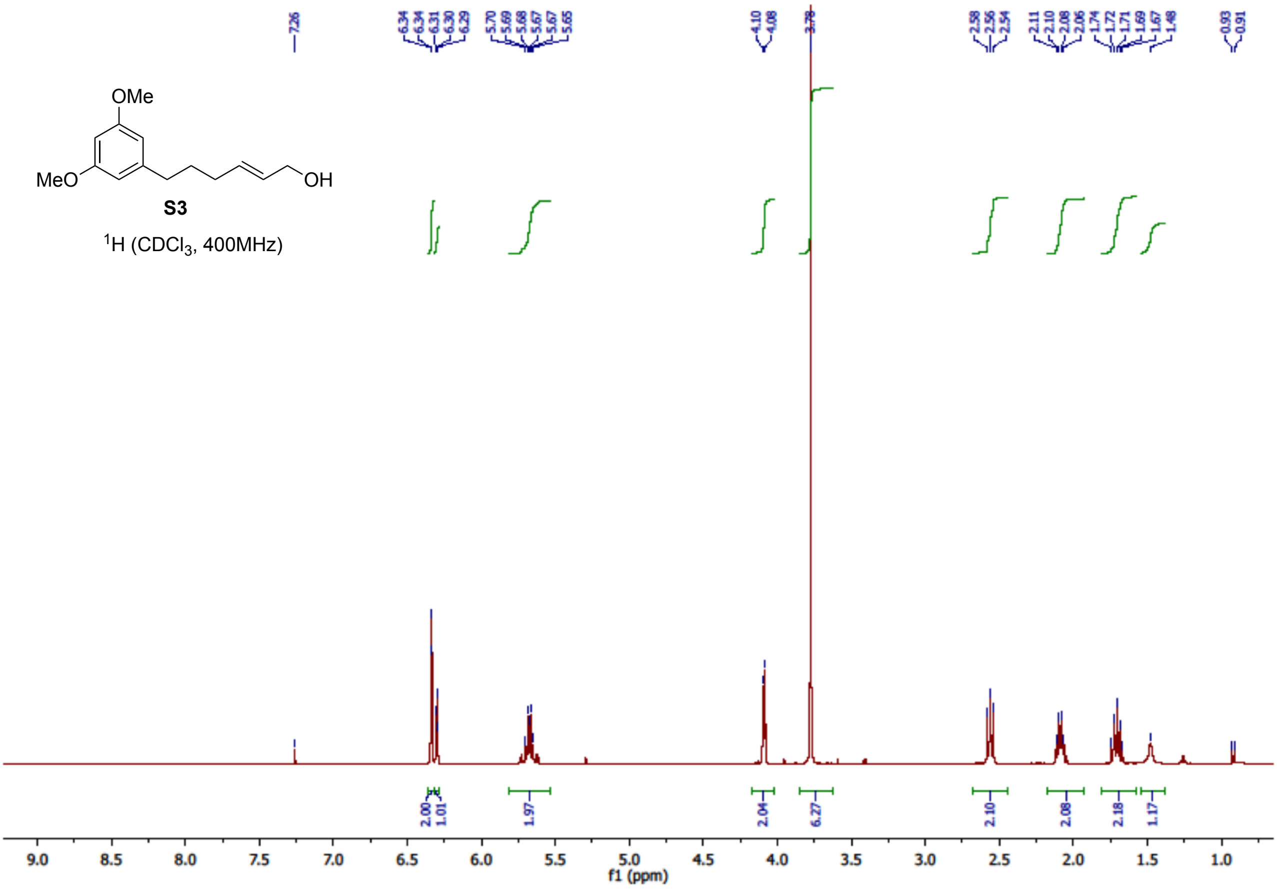
OMe
MeO
OH
S3
1H (CDCl3, 400MHz)
f1 (ppm)

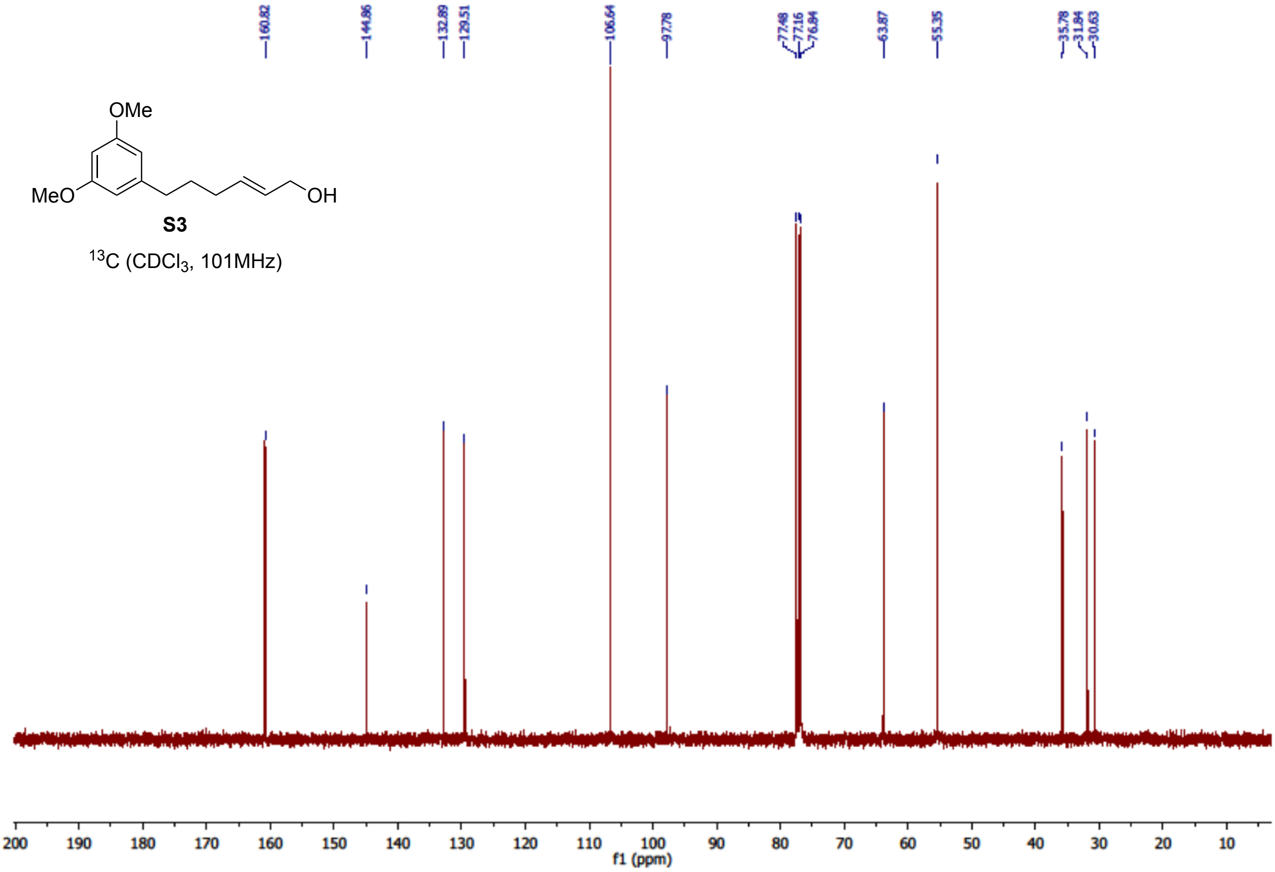
OMe
MeO
OH
S3
13C (CDCl3, 101MHz)
f1 (ppm)

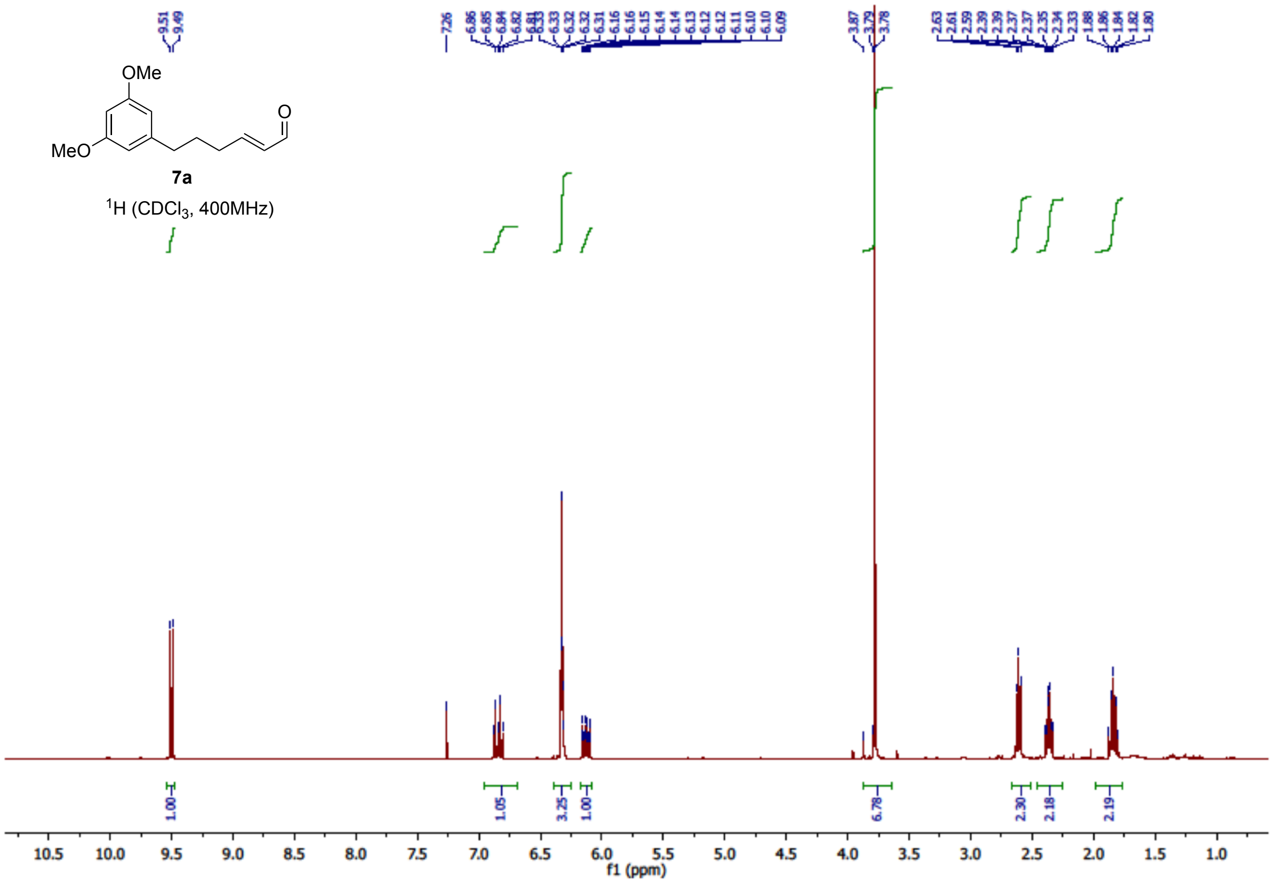
OMe
MeO
O
7a
^{1}H (CDCl$_3$, 400MHz)
f1 (ppm)

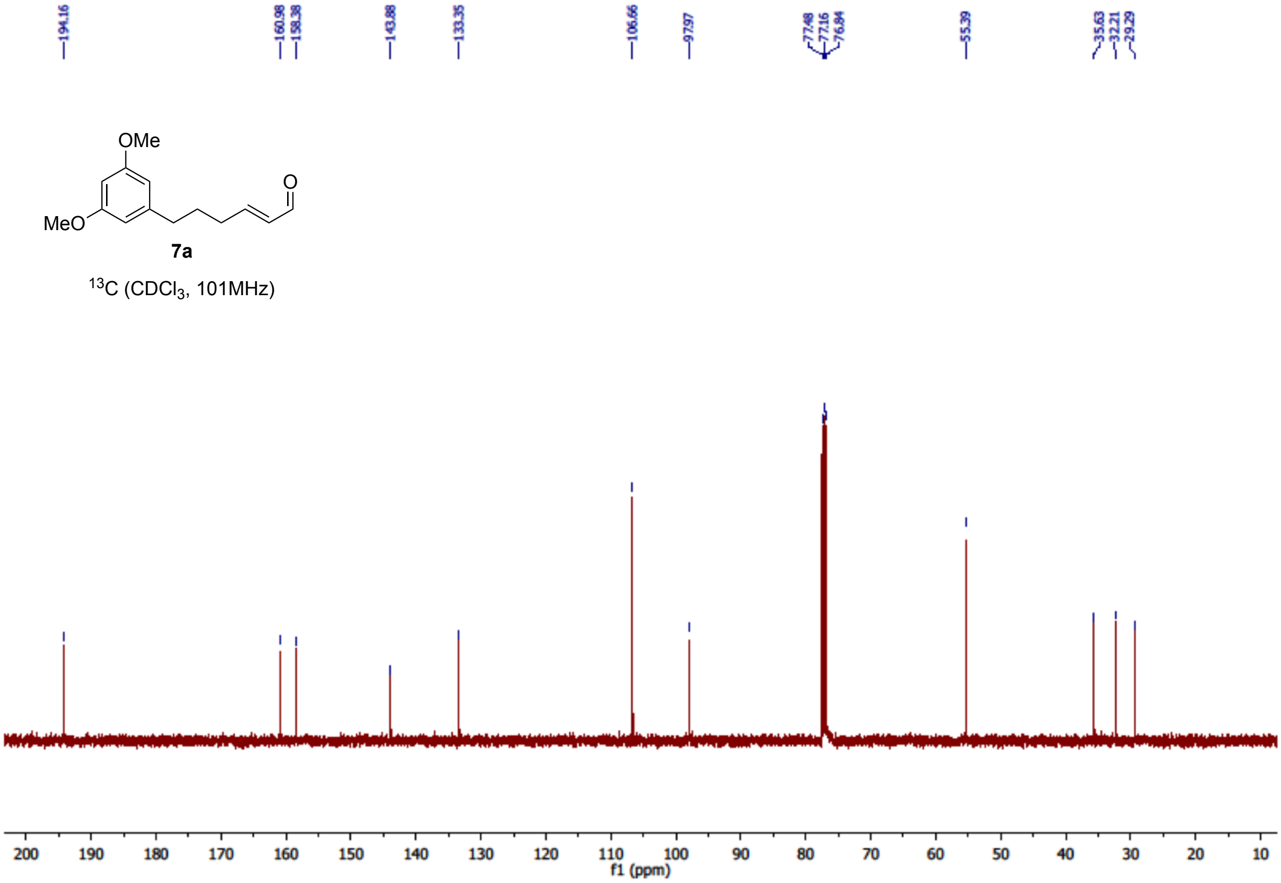
OMe
MeO
O
7a
^{13}C (CDCl$_3$, 101MHz)
f1 (ppm)

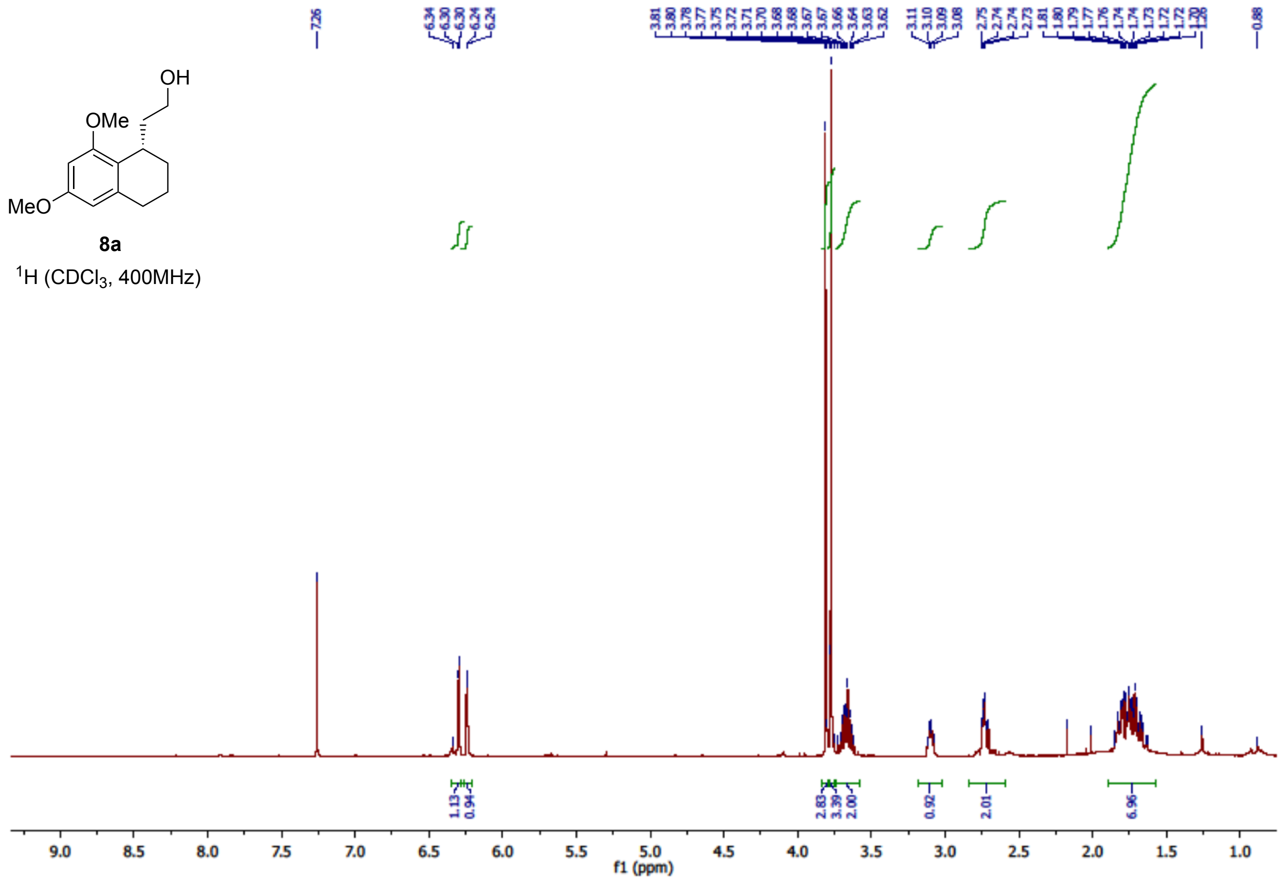

OH
OMe
MeO
8a
^{1}H (CDCl3, 400MHz)
f1 (ppm)

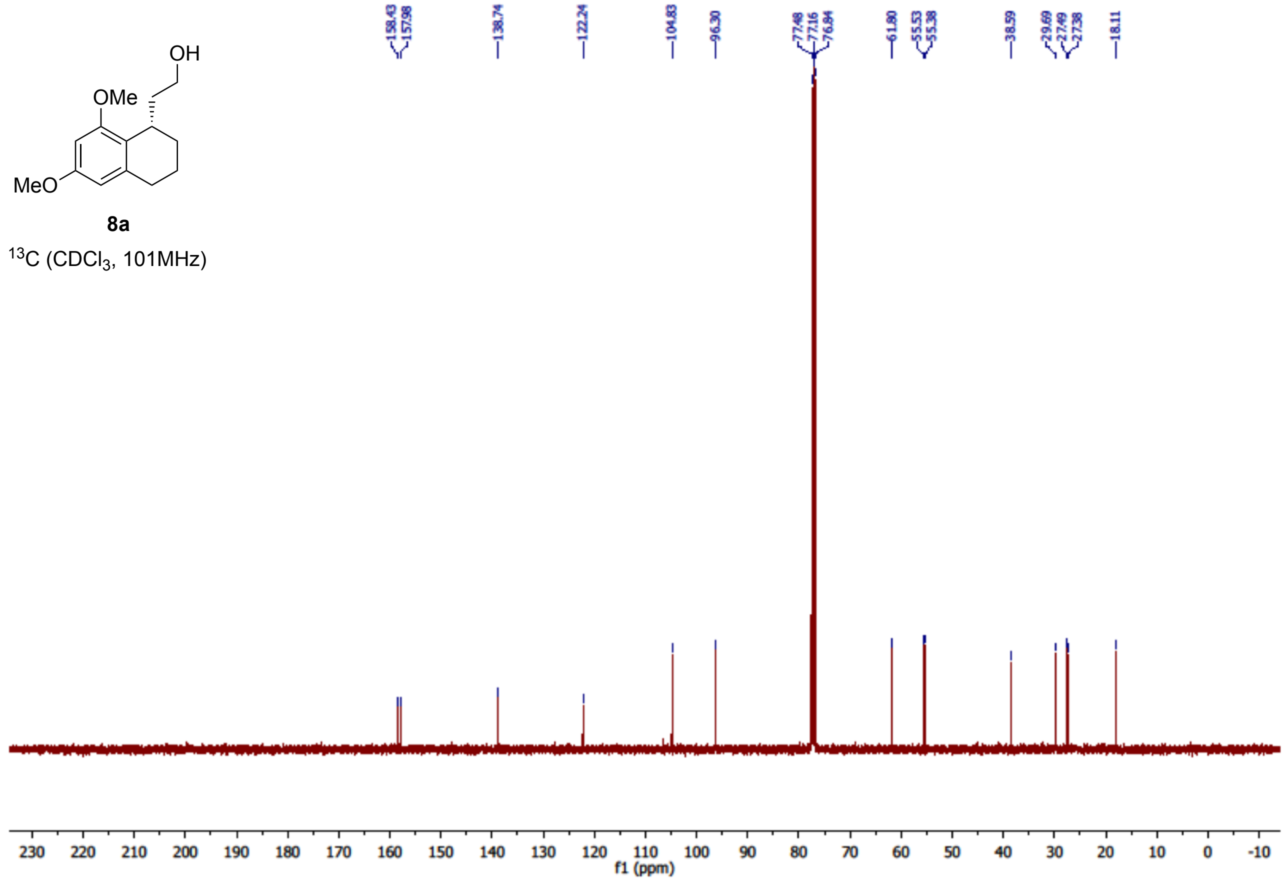

OH
OMe
MeO
8a
^{13}C (CDCl3, 101MHz)
f1 (ppm)

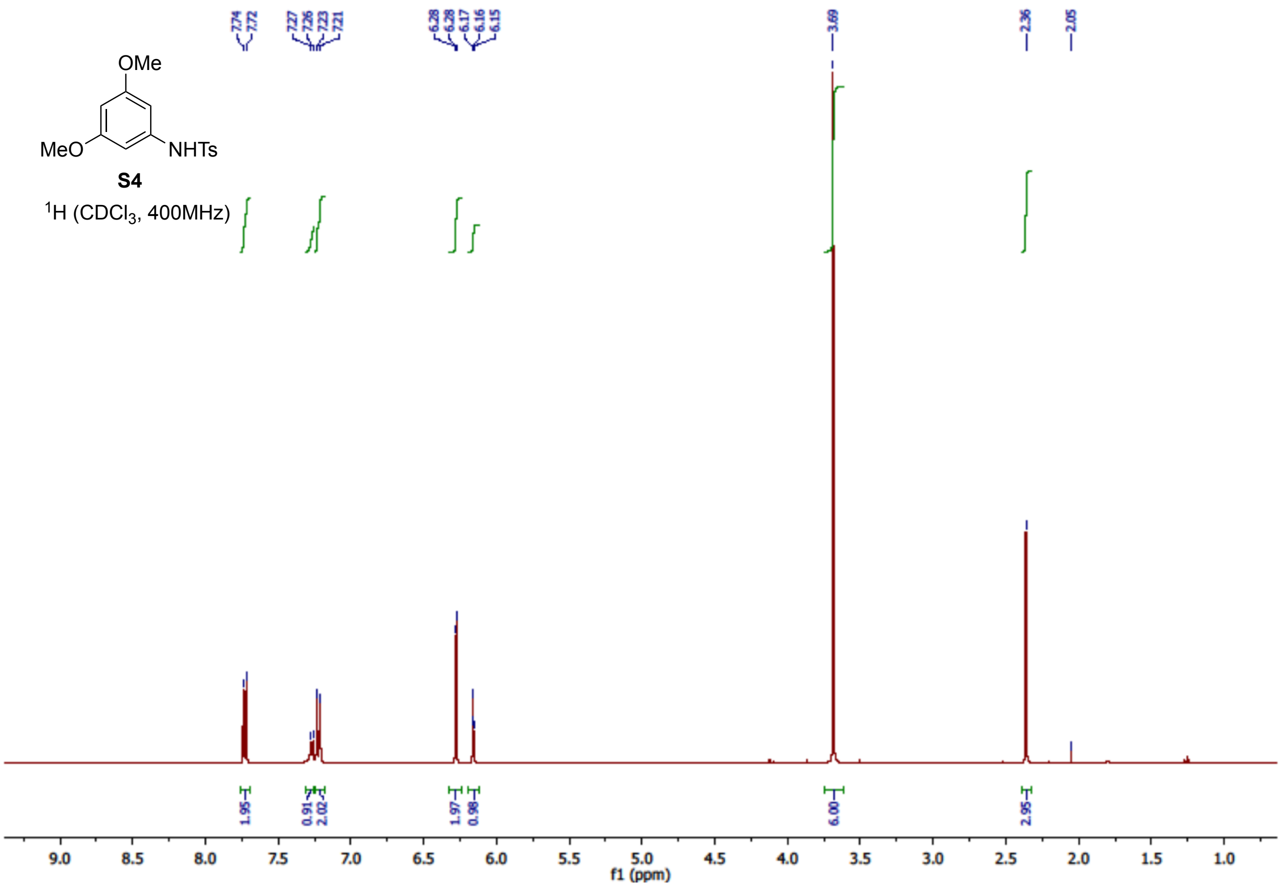
OMe
MeO
NHTs
S4
1H (CDCl3, 400MHz)
7.74 7.72 7.27 7.26 7.23 7.21 6.28 6.28 6.17 6.16 6.15 3.69 2.36 2.05
1.95 0.91 2.02 1.97 0.98 6.00 2.95
9.0 8.5 8.0 7.5 7.0 6.5 6.0 5.5 5.0 4.5 4.0 3.5 3.0 2.5 2.0 1.5 1.0
f1 (ppm)

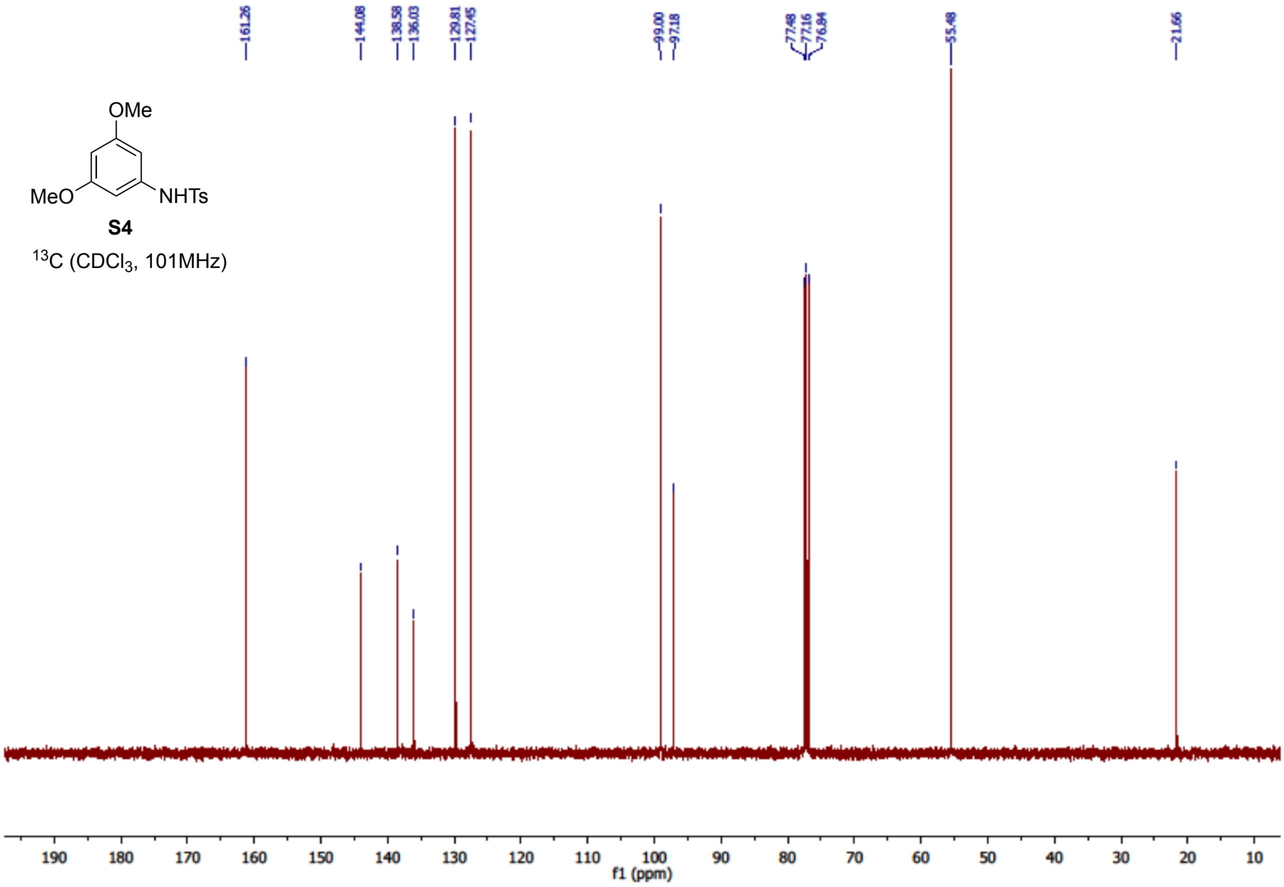
OMe
MeO
NHTs
S4
13C (CDCl3, 101MHz)
161.26 144.08 138.58 136.03 129.81 127.45 99.00 97.18 77.48 77.16 76.84 55.48 21.66
190 180 170 160 150 140 130 120 110 100 90 80 70 60 50 40 30 20 10
f1 (ppm)

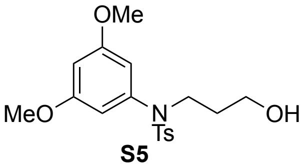

S5

^{1}H ($CDCl_3$, 400MHz)

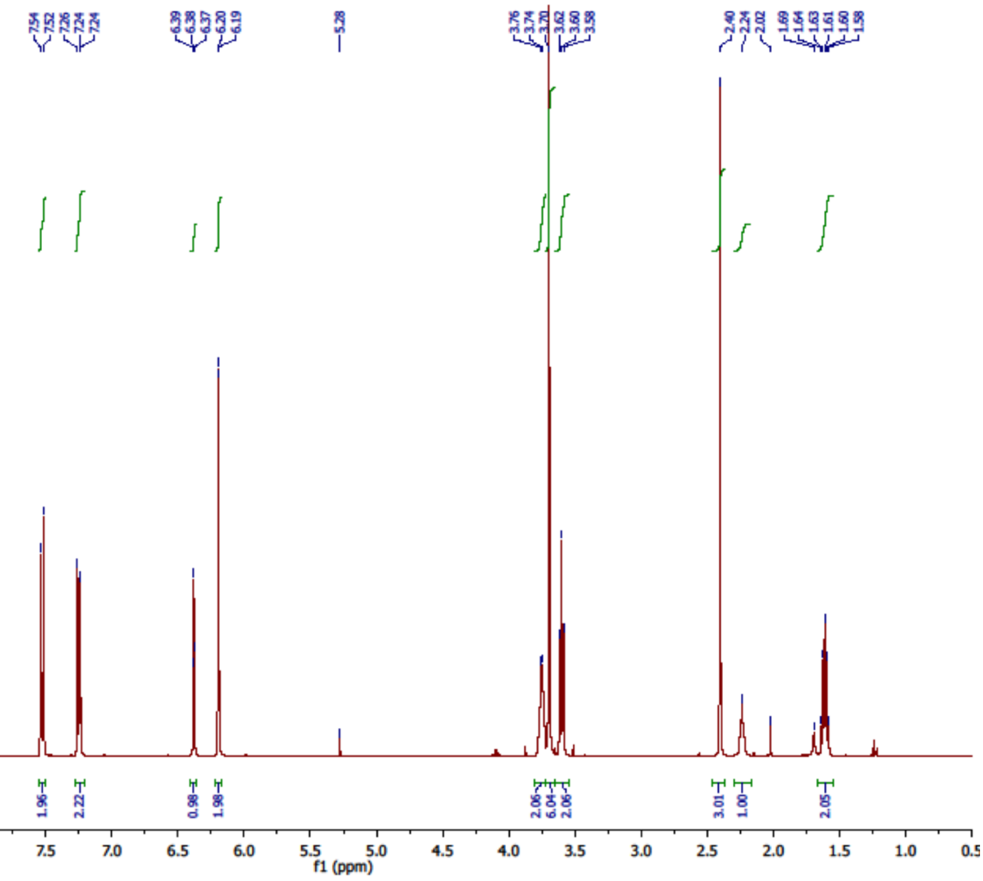

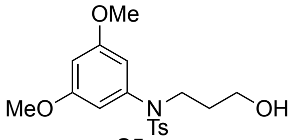

S5

^{13}C ($CDCl_3$, 101MHz)

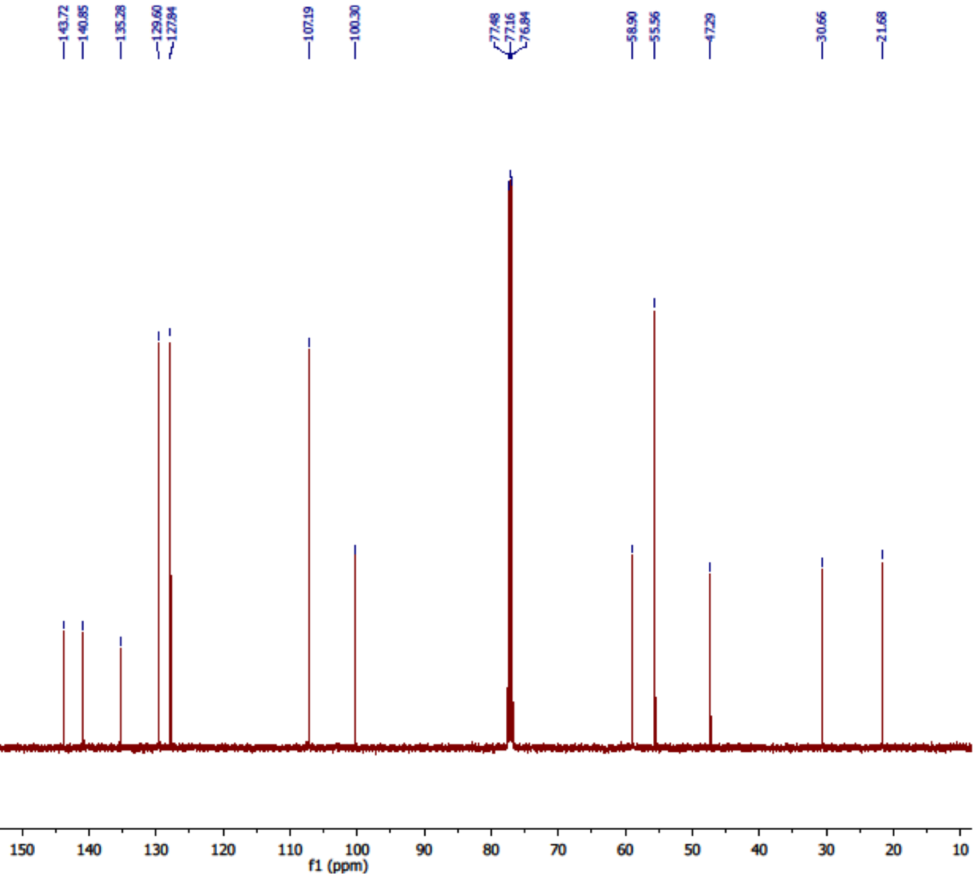

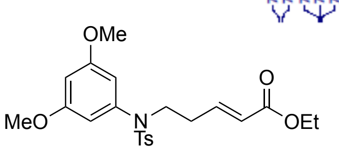

S6

^{1}H ($CDCl_3$, 400MHz)

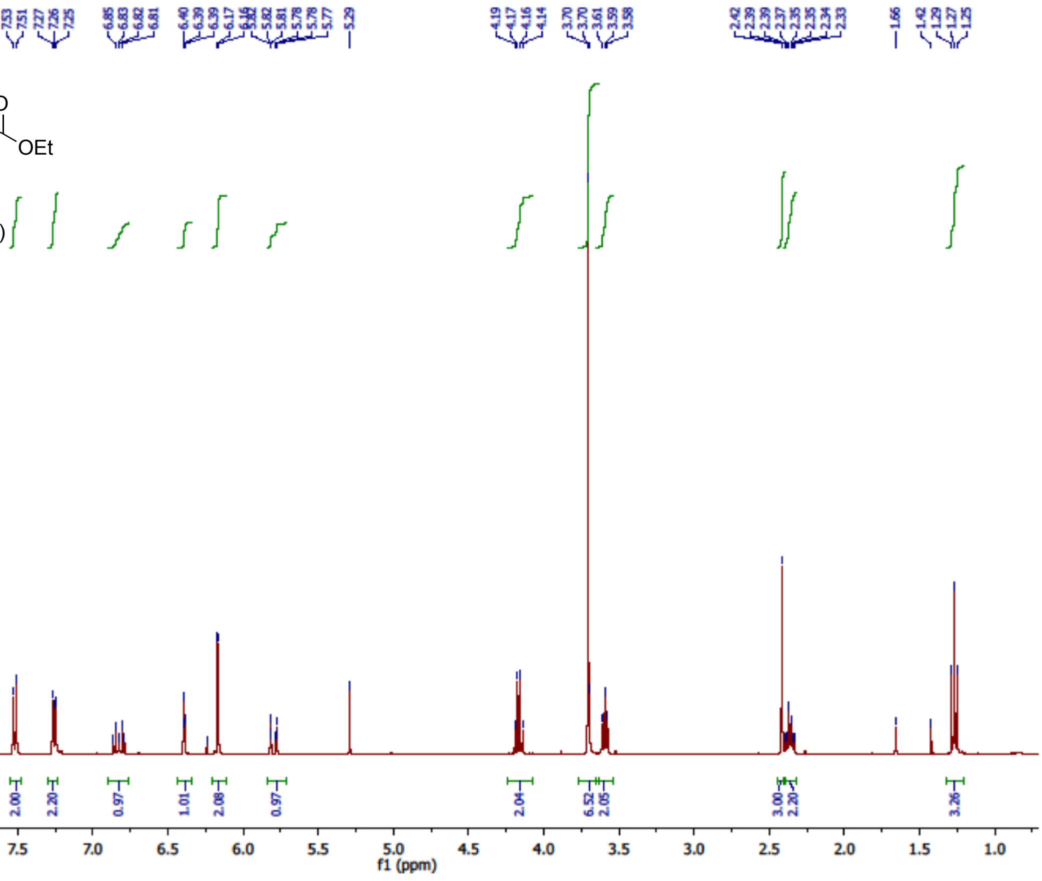

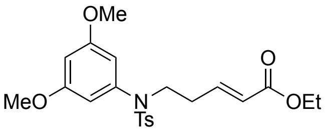

S6

^{13}C ($CDCl_3$, 101MHz)

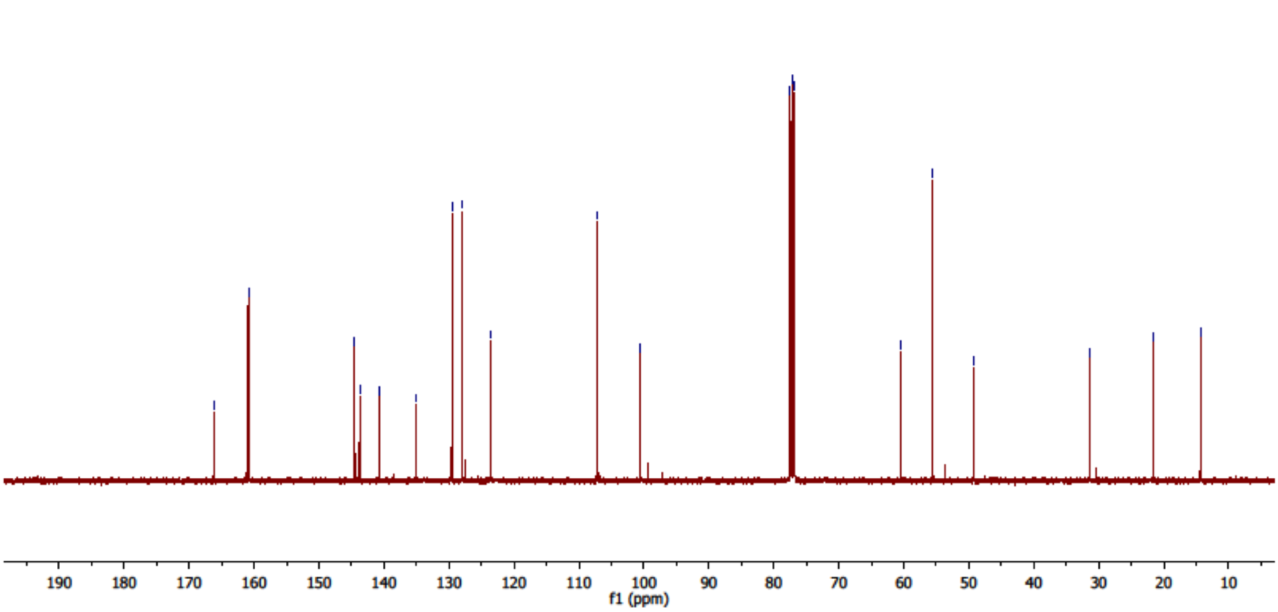

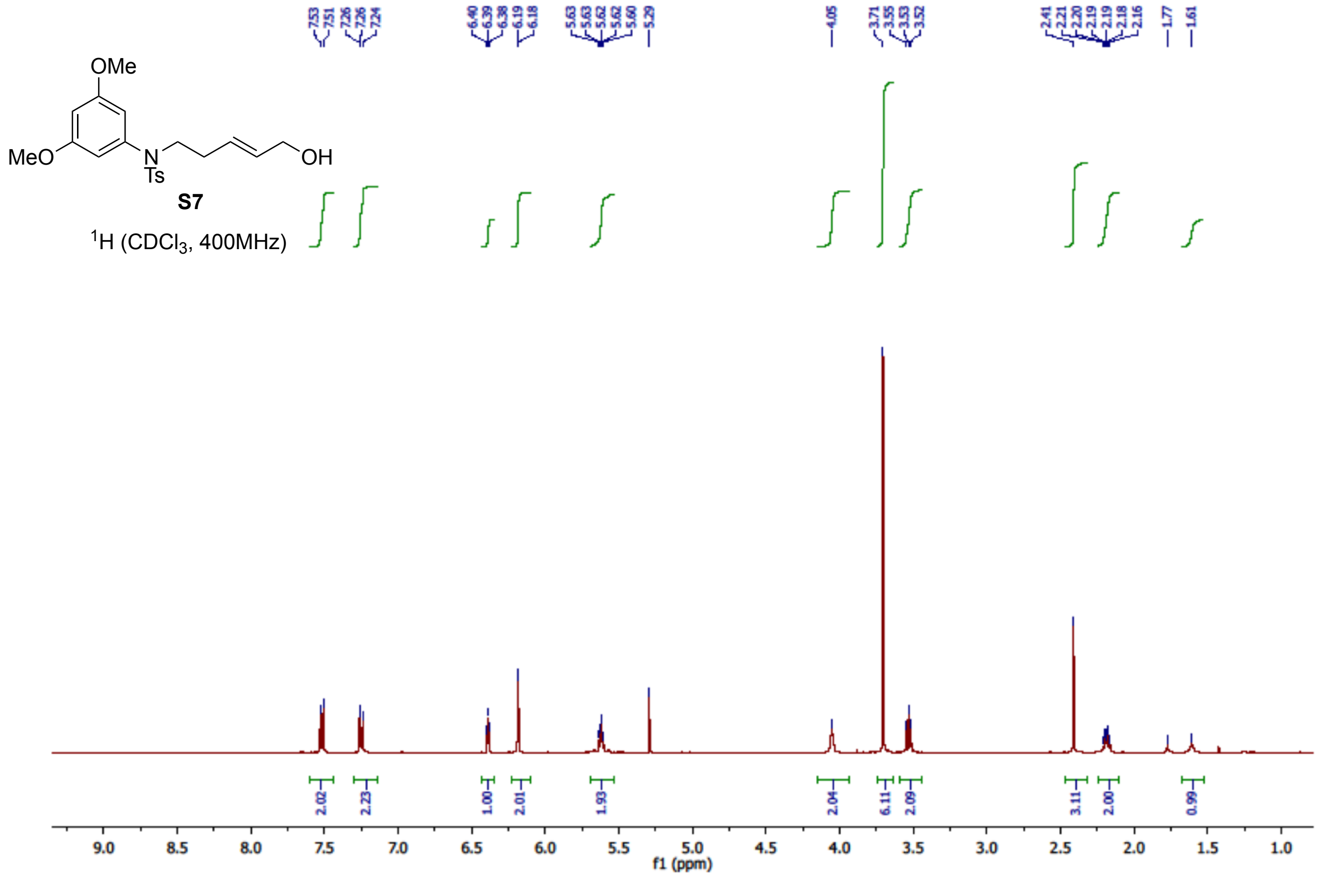

S7

^{1}H ($CDCl_3$, 400MHz)

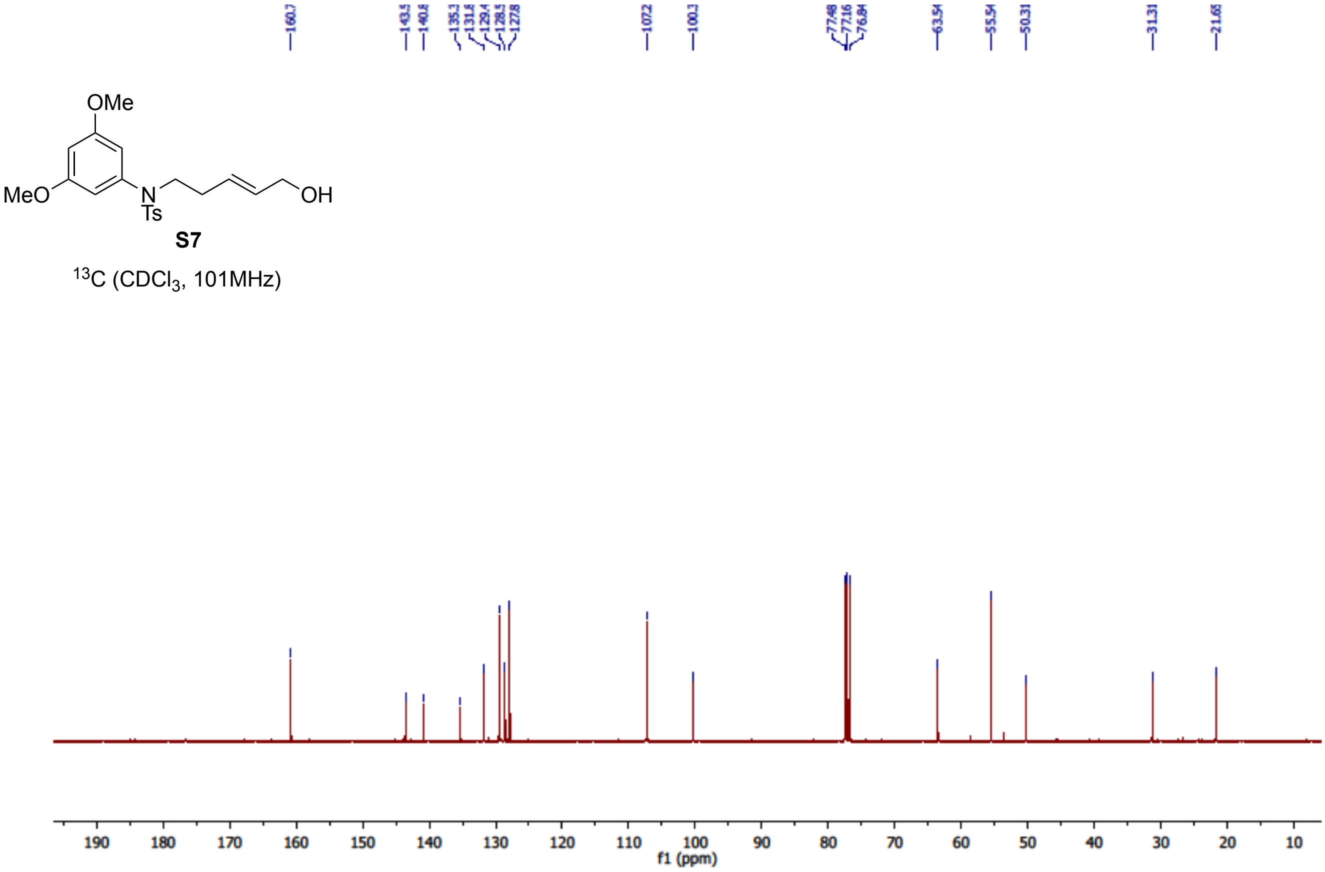

S7

^{13}C ($CDCl_3$, 101MHz)

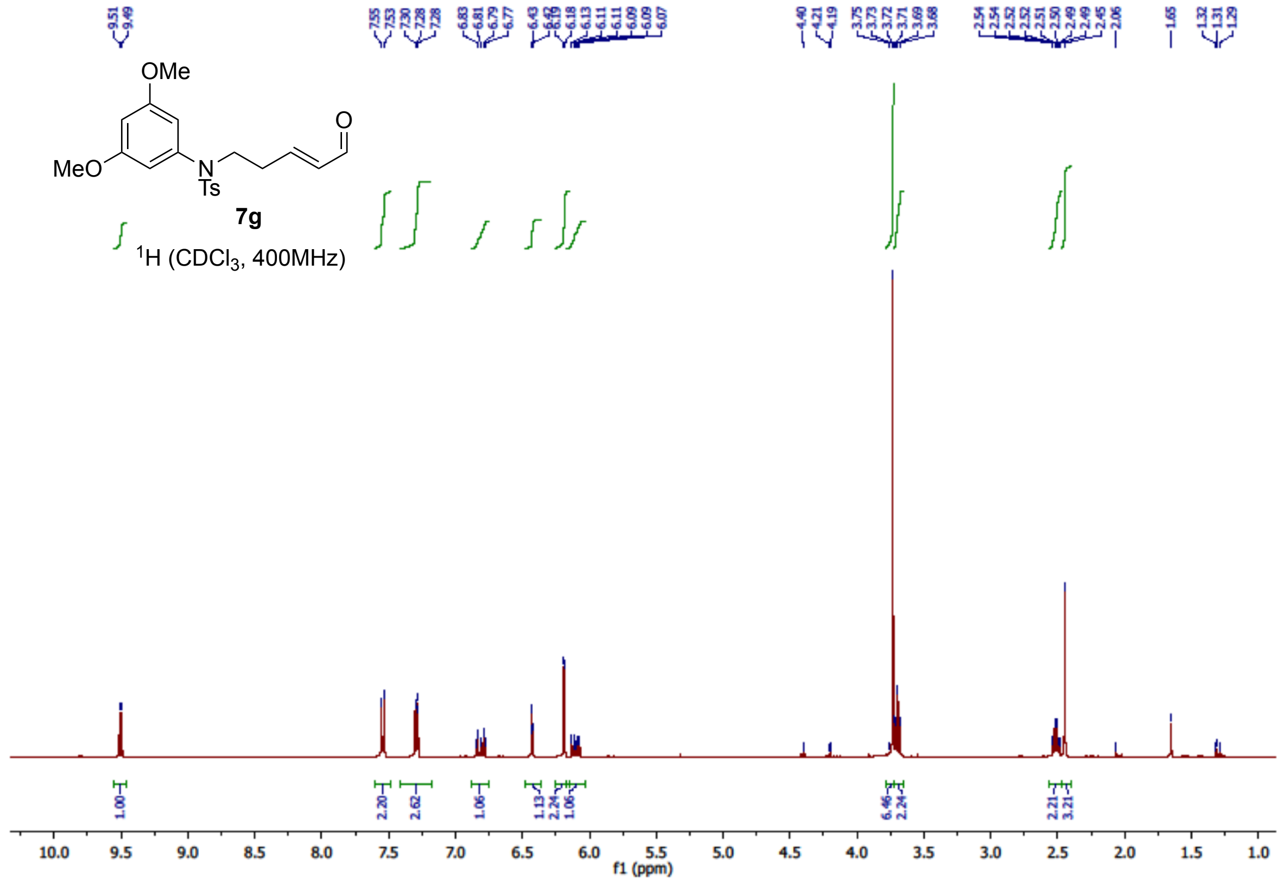
OMe
MeO
N
Ts
O
7g
1H (CDCl3, 400MHz)
f1 (ppm)

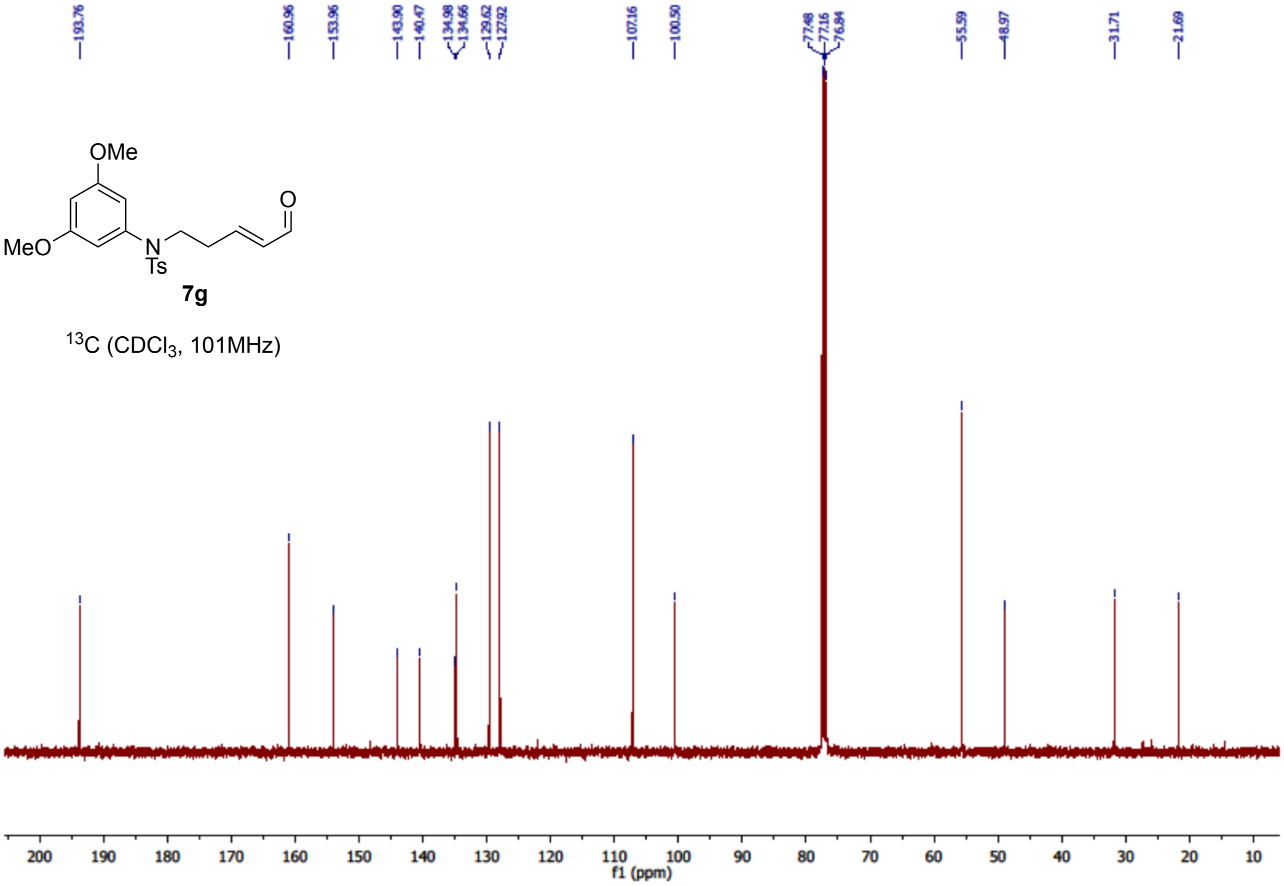
OMe
MeO
N
Ts
O
7g
13C (CDCl3, 101MHz)
f1 (ppm)

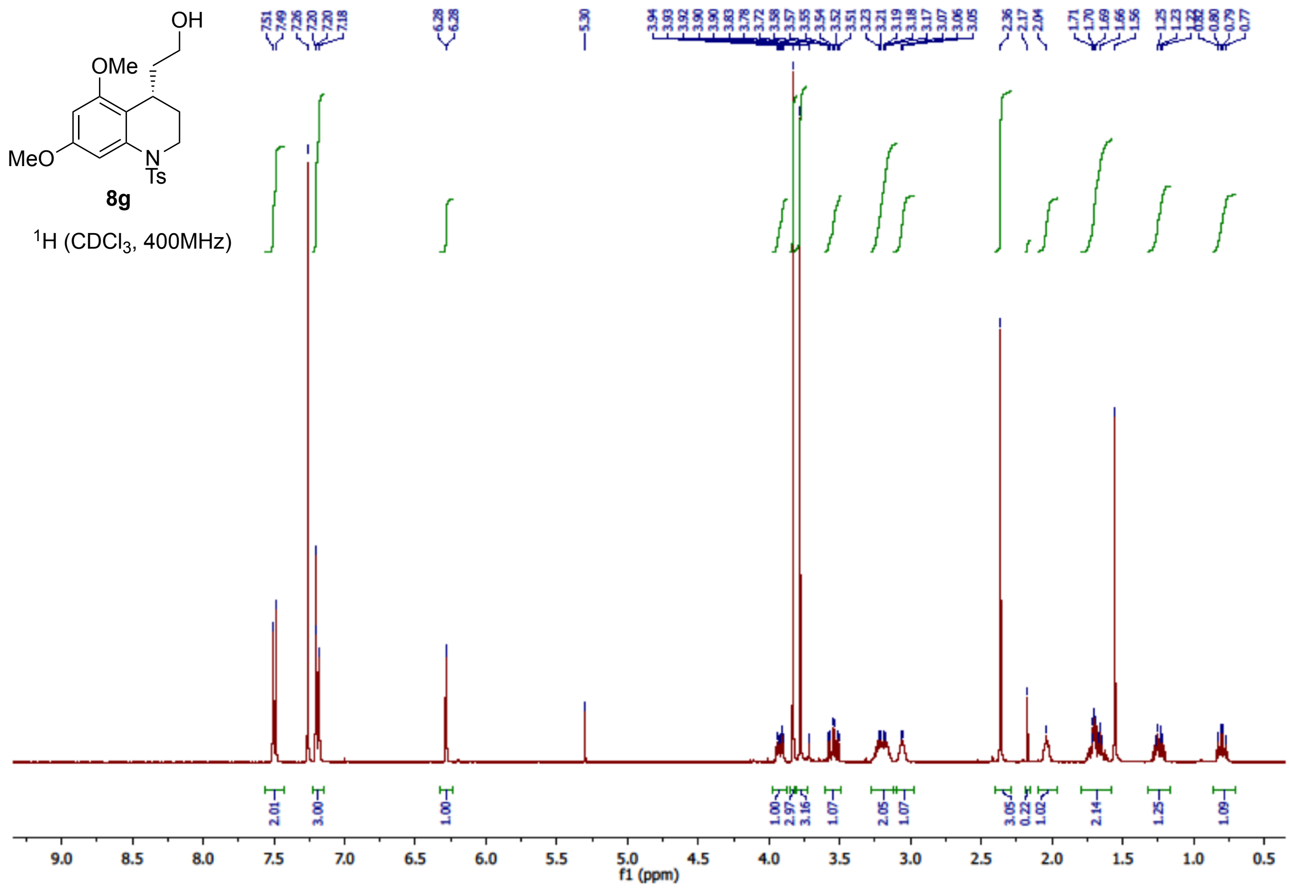
OH
OMe
MeO
N
Ts
8g
1H (CDCl3, 400MHz)
f1 (ppm)

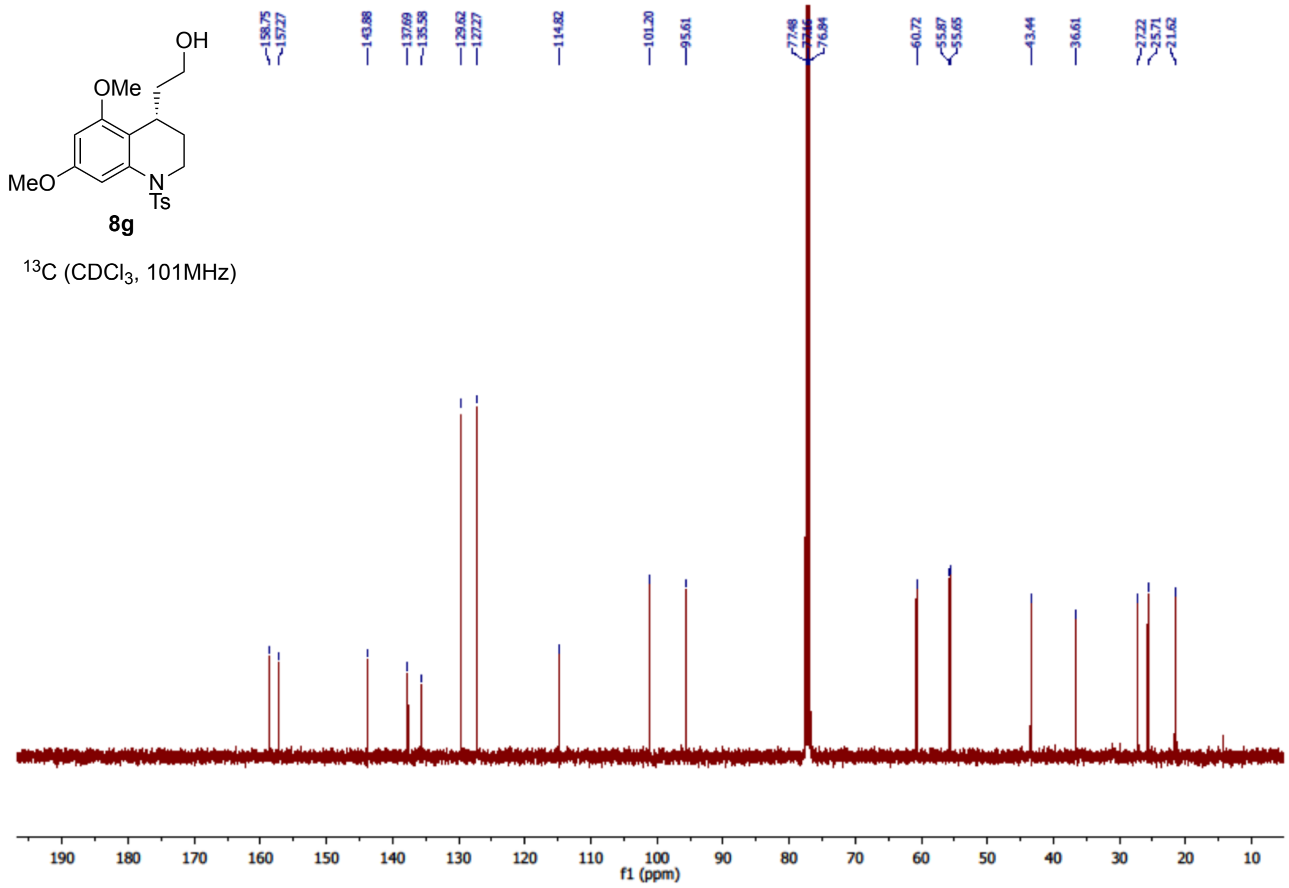
OH
OMe
MeO
N
Ts
8g
13C (CDCl3, 101MHz)
f1 (ppm)

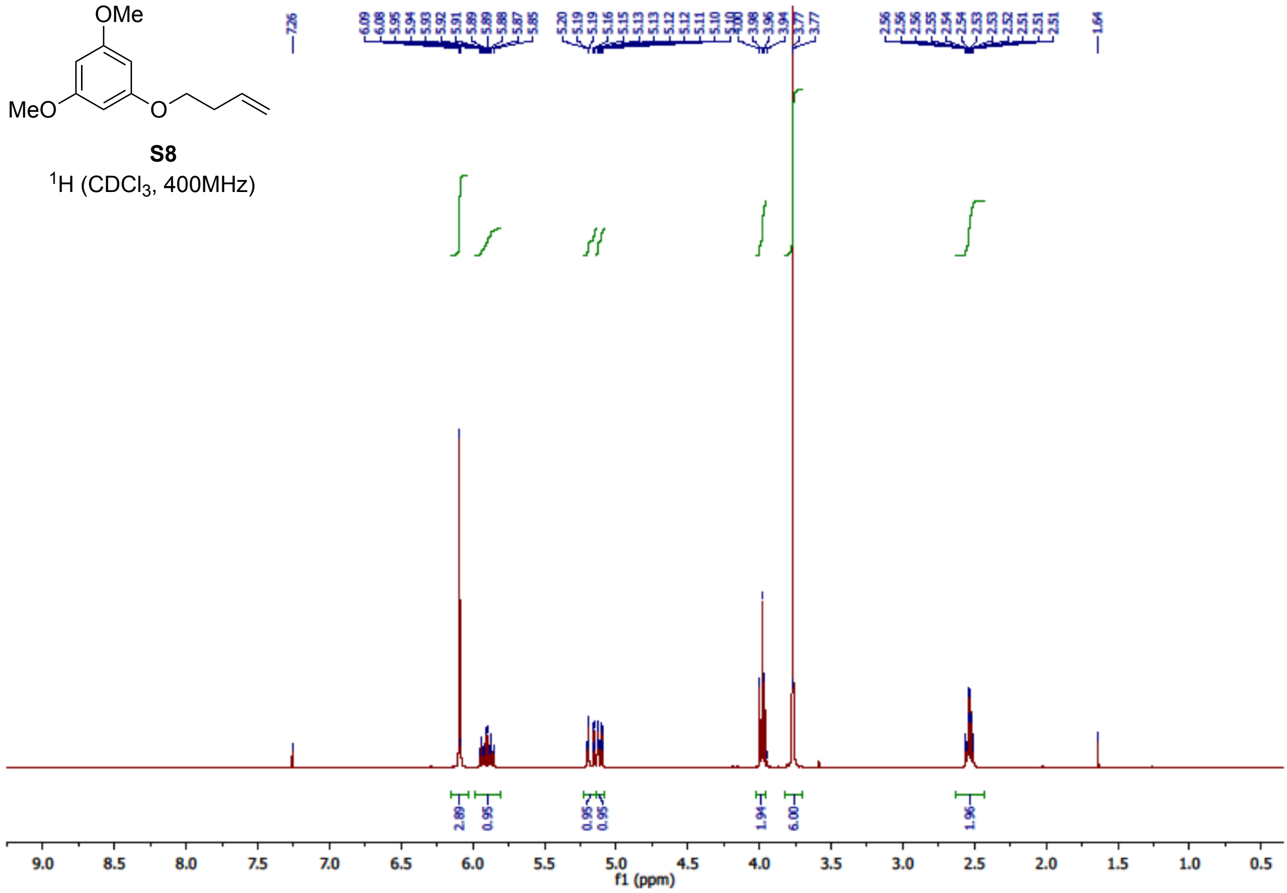

S8

^{1}H ($CDCl_3$, 400MHz)

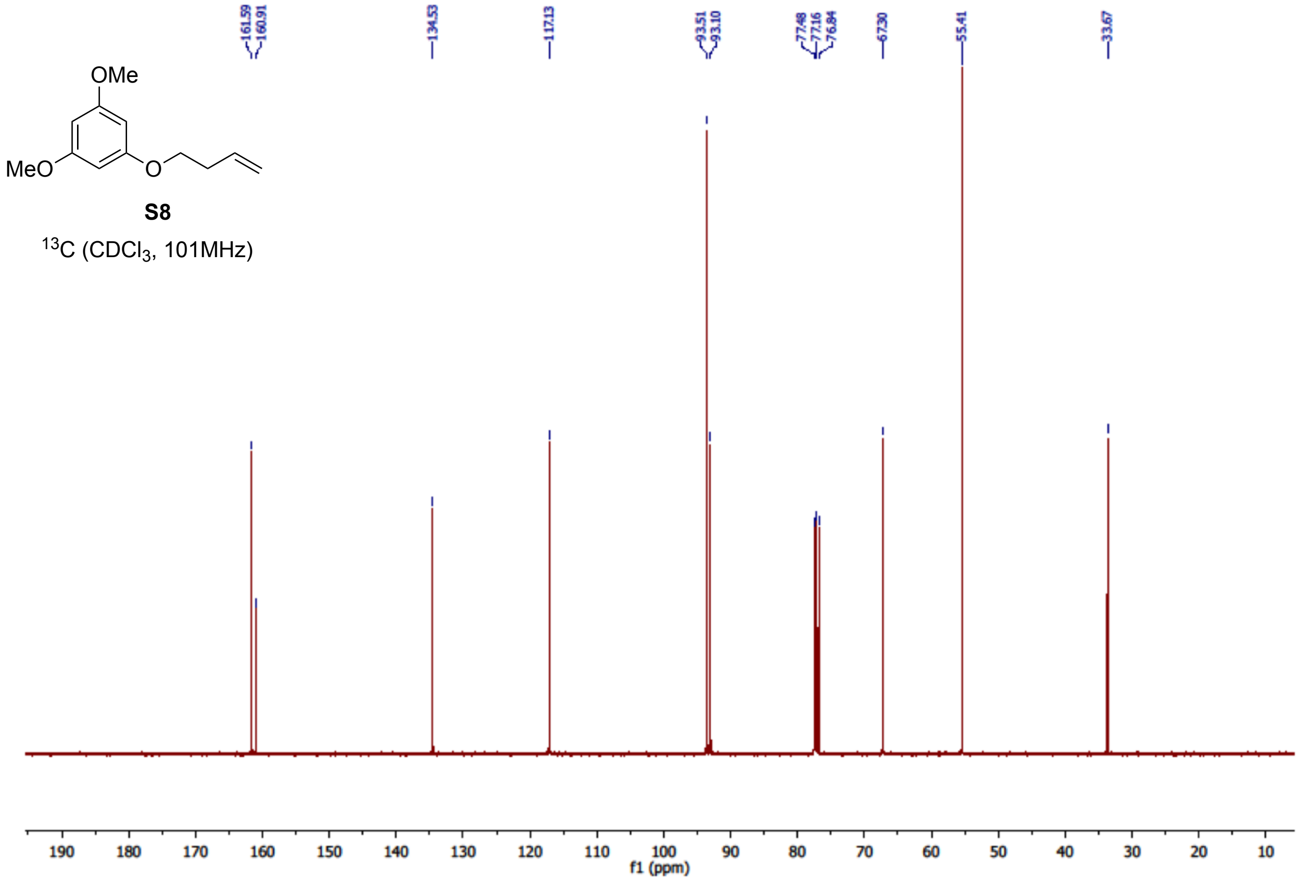

S8

^{13}C ($CDCl_3$, 101MHz)

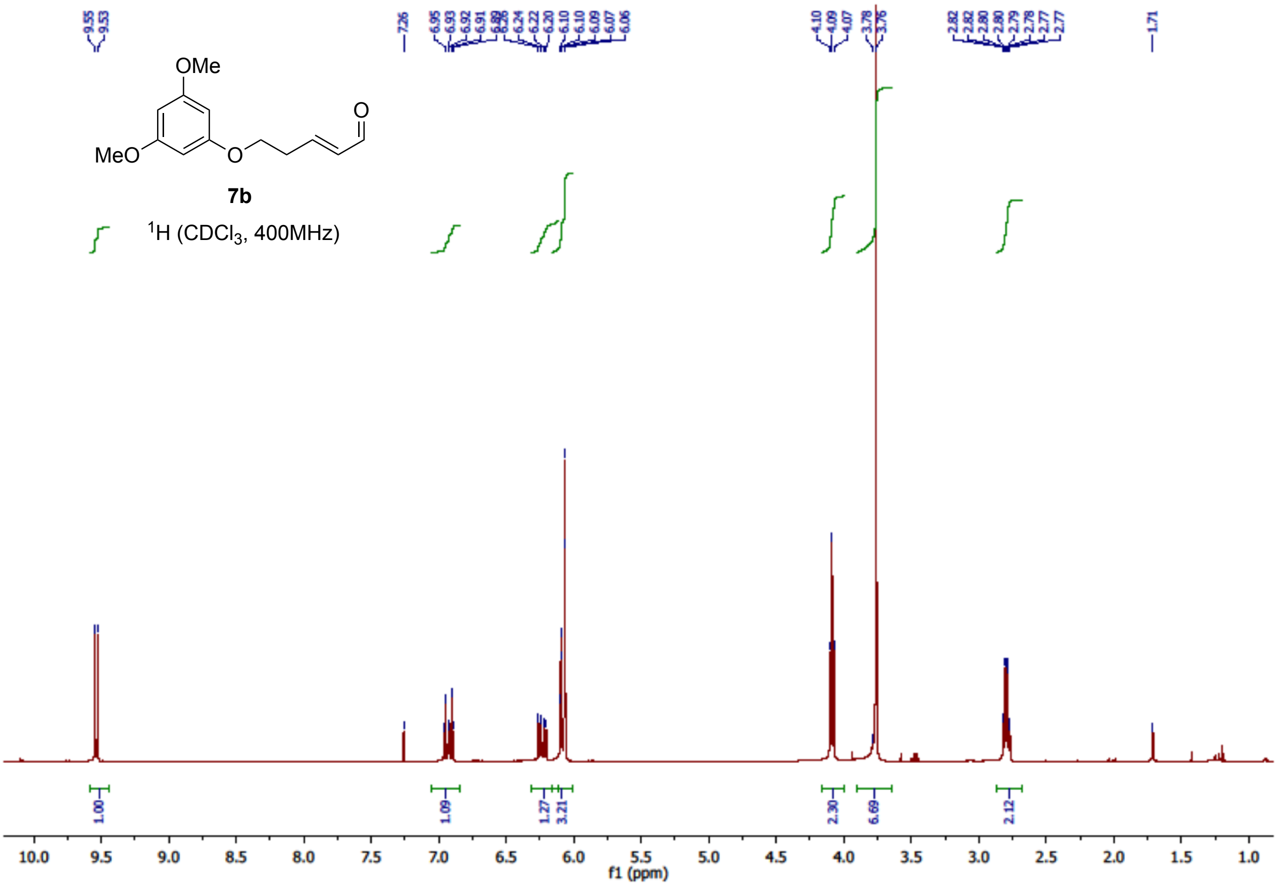
OMe
MeO
O
O
7b
1H (CDCl3, 400MHz)
f1 (ppm)

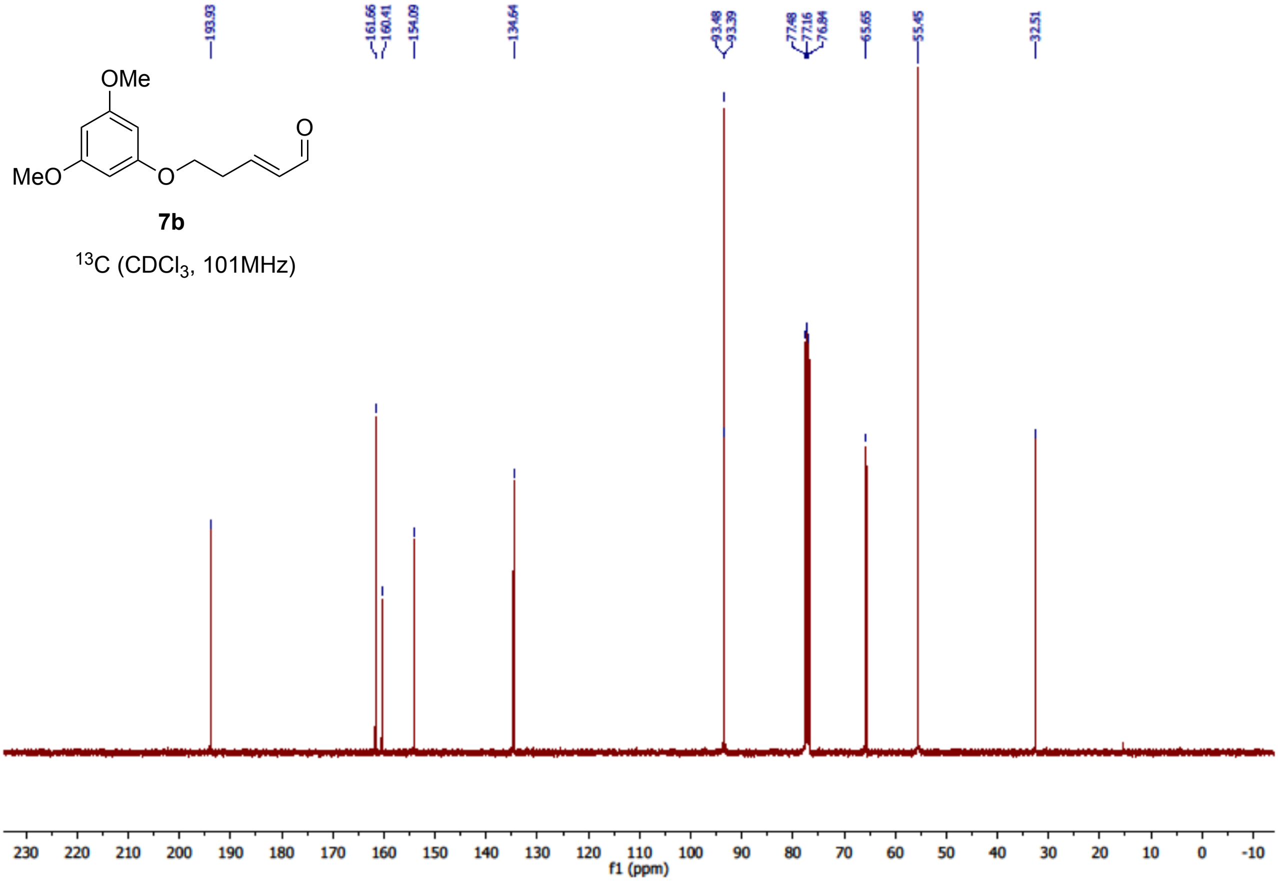
OMe
MeO
O
O
7b
13C (CDCl3, 101MHz)
f1 (ppm)

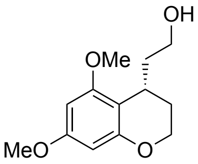

8b

^1H ($CDCl_3$, 400MHz)

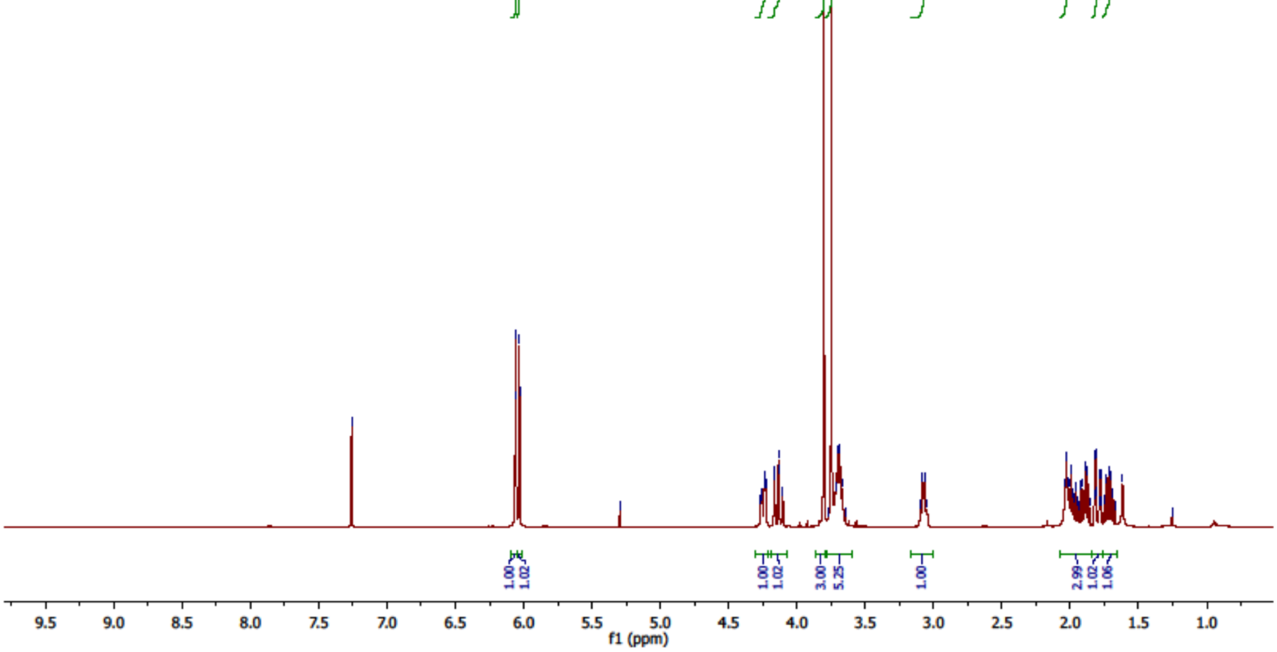

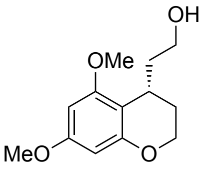

8b

^{13}C ($CDCl_3$, 101MHz)

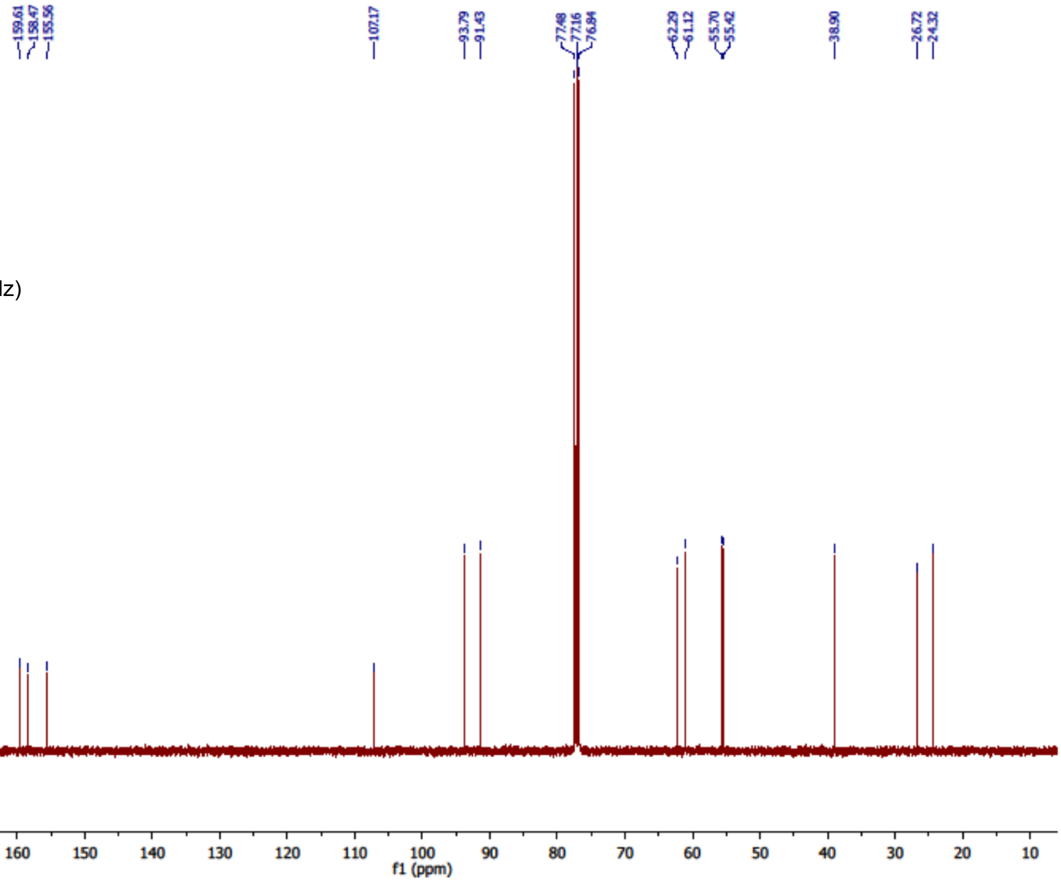

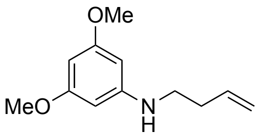

S9

^{1}H ($CDCl_3$, 400MHz)

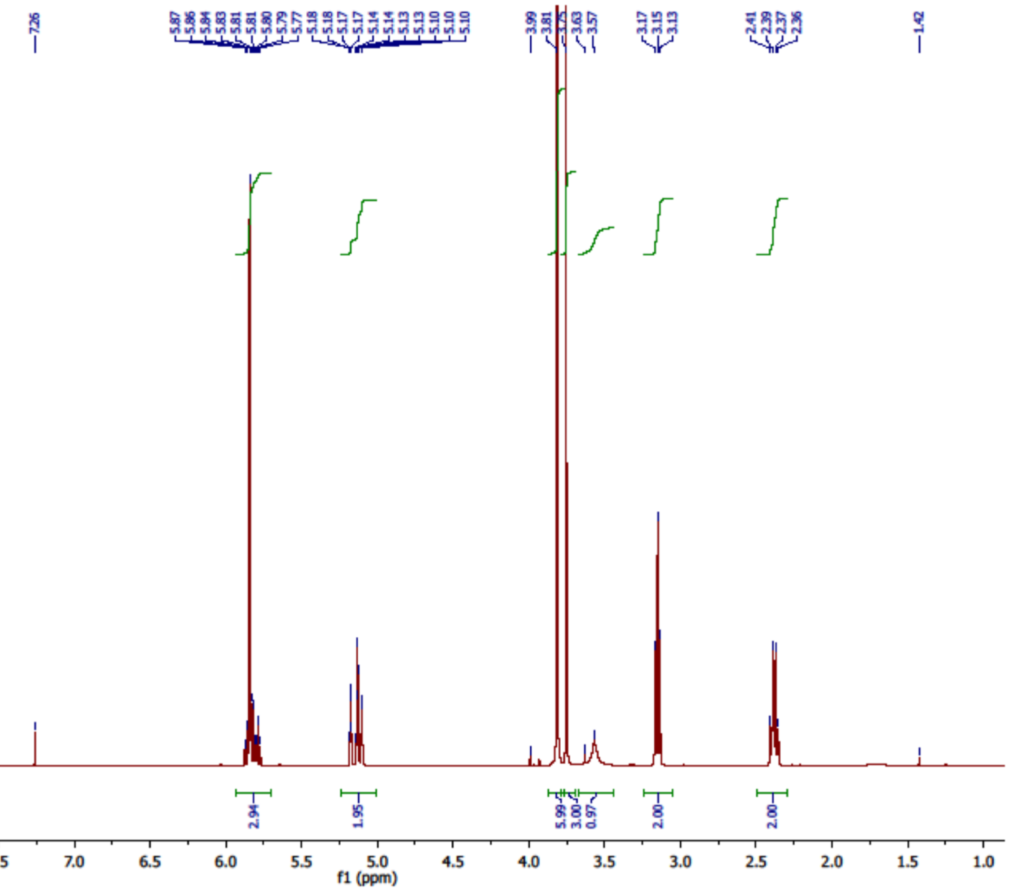

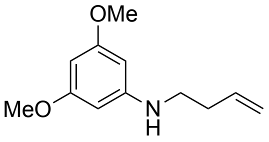

S9

^{13}C ($CDCl_3$, 101MHz)

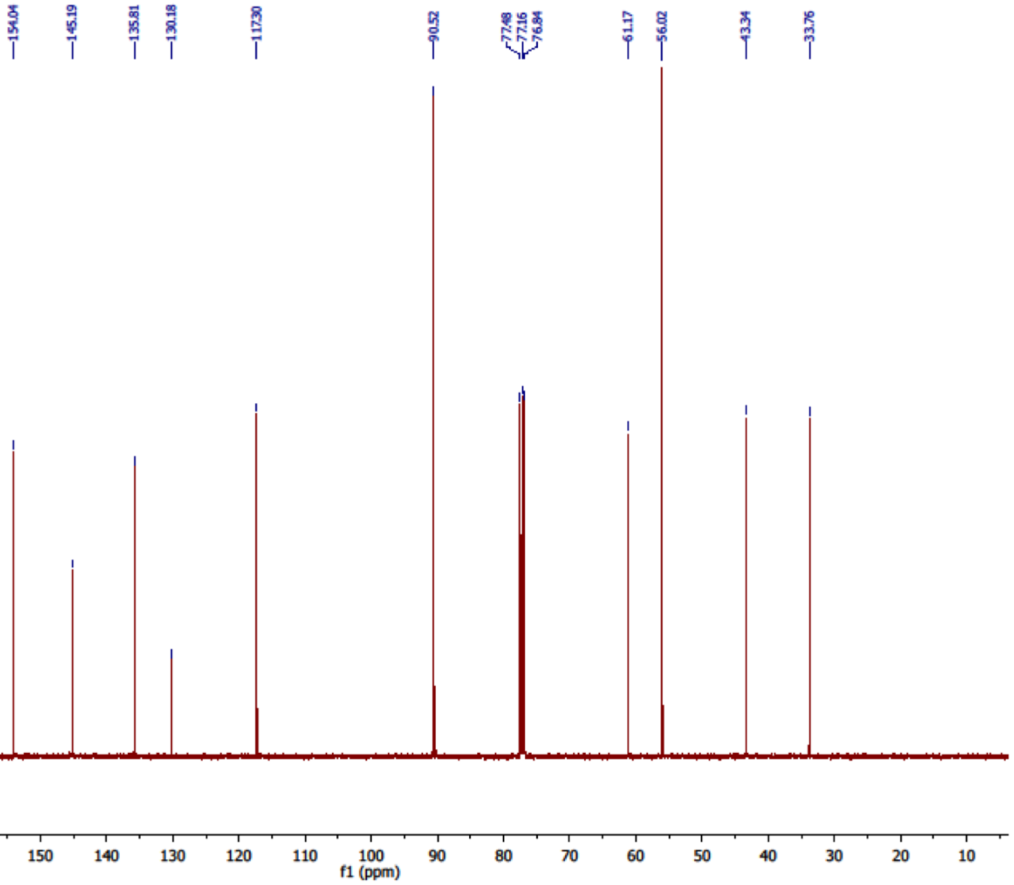

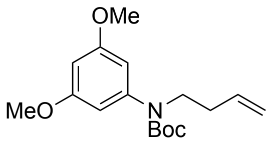

S10

^{1}H ($CDCl_3$, 400MHz)

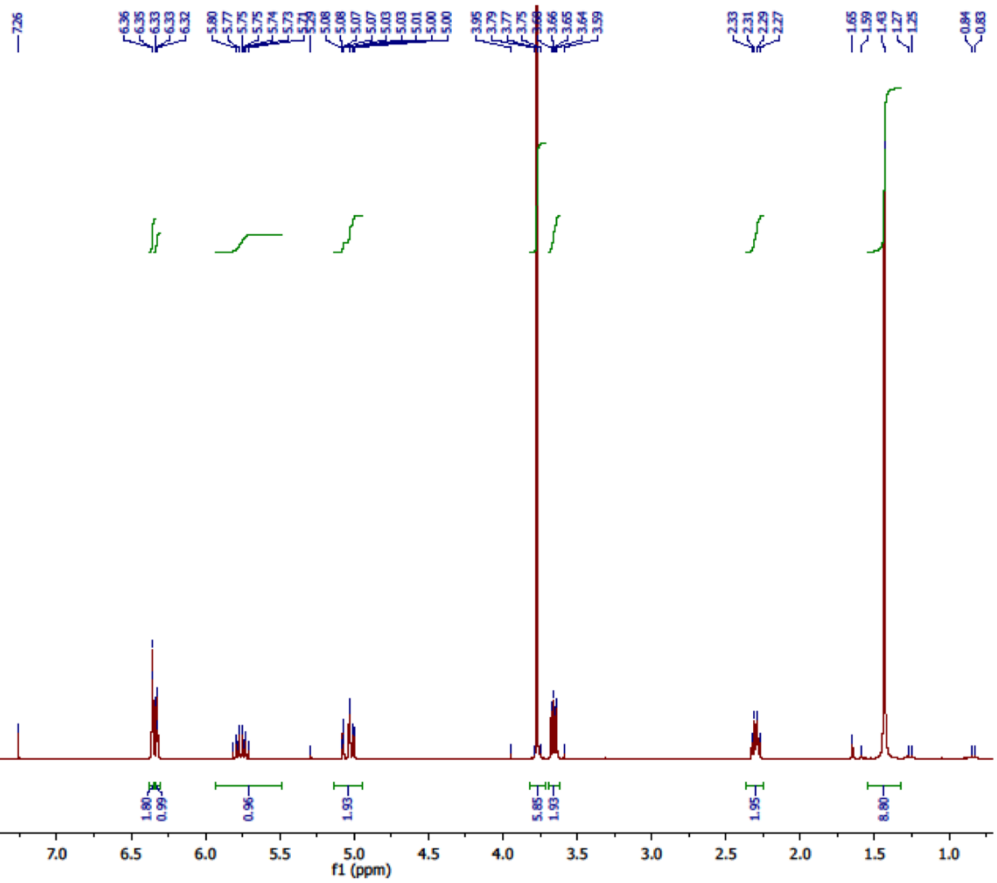

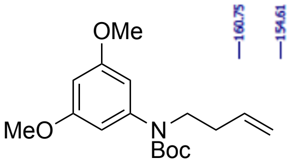

S10

^{13}C ($CDCl_3$, 101MHz)

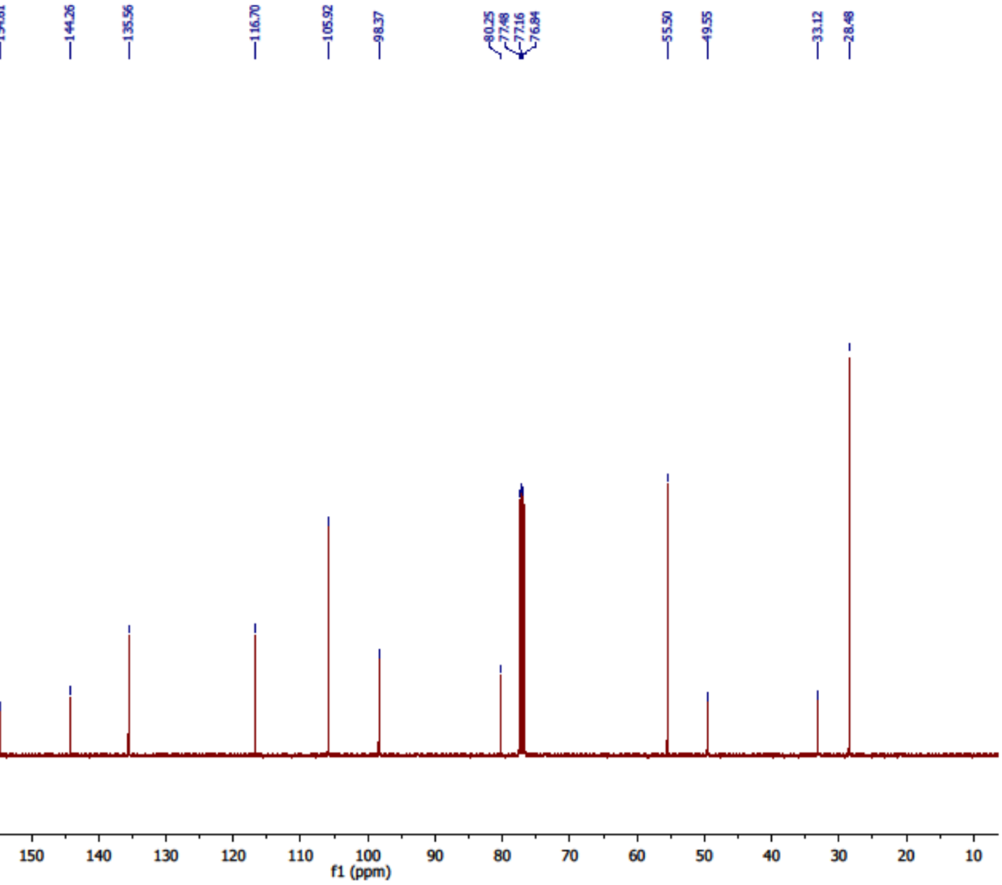

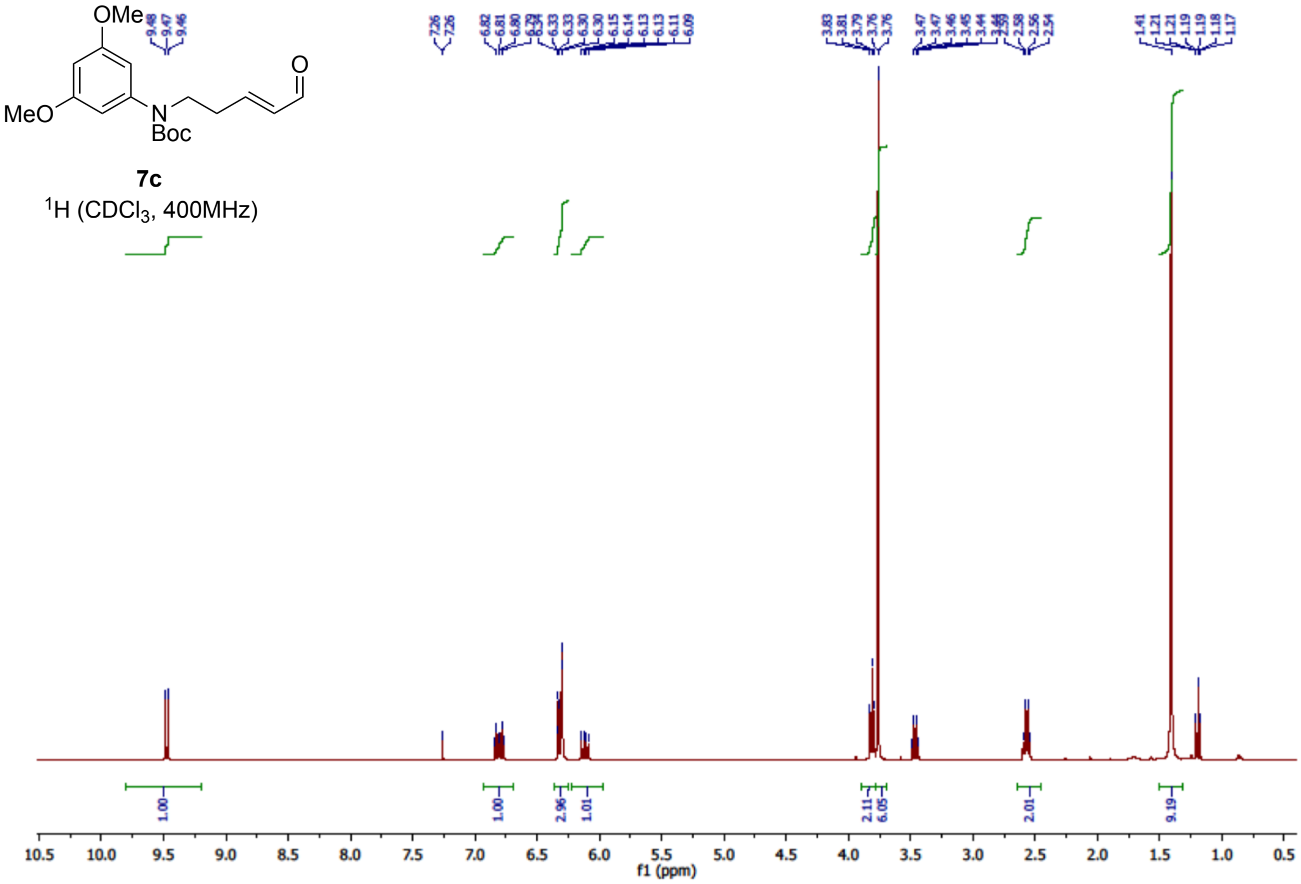

7c

^{1}H ($CDCl_3$, 400MHz)

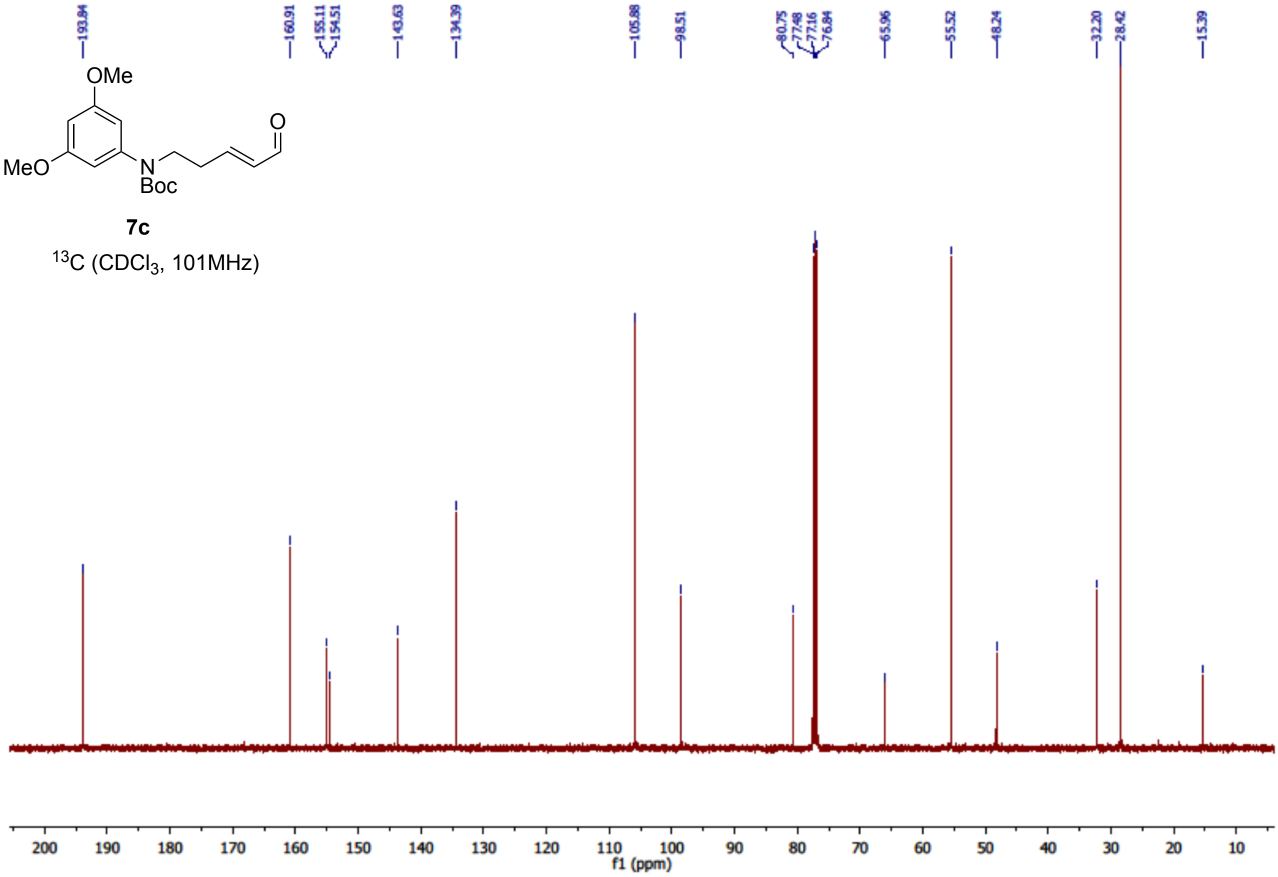

7c

^{13}C ($CDCl_3$, 101MHz)

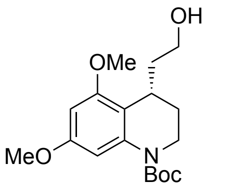

8c

^{1}H ($CDCl_3$, 400MHz)

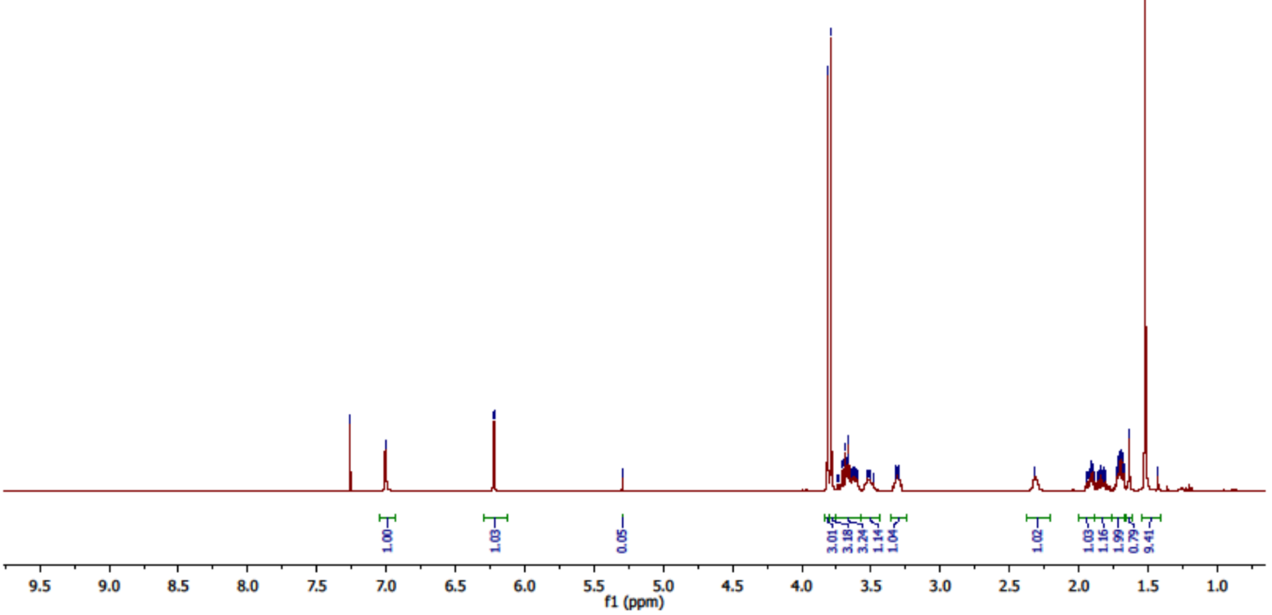

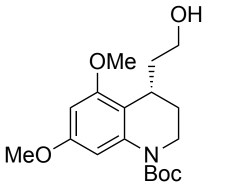

8c

^{13}C ($CDCl_3$, 101MHz)

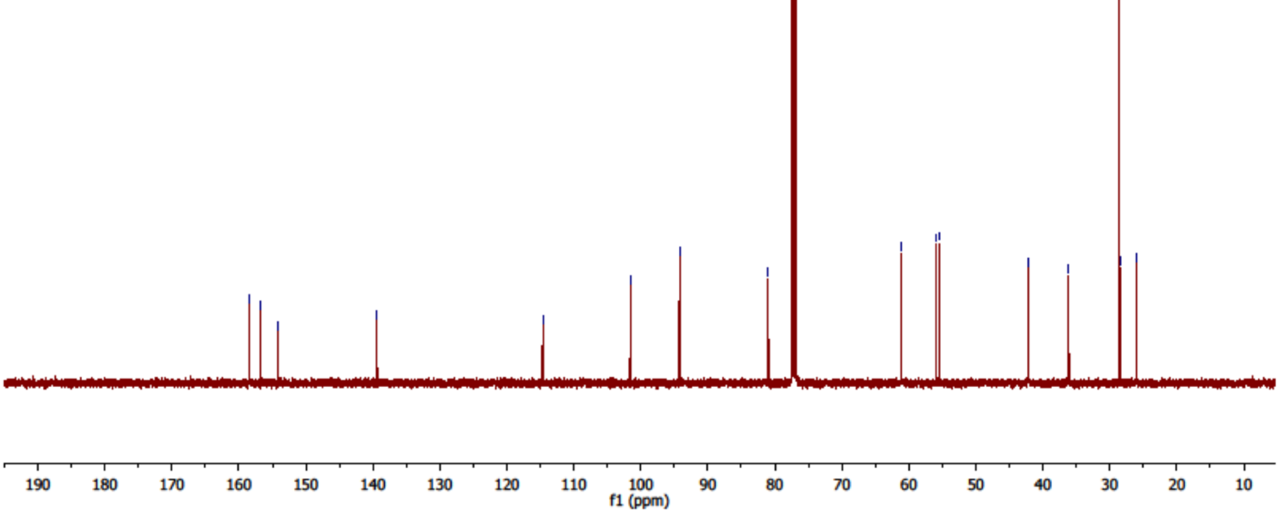

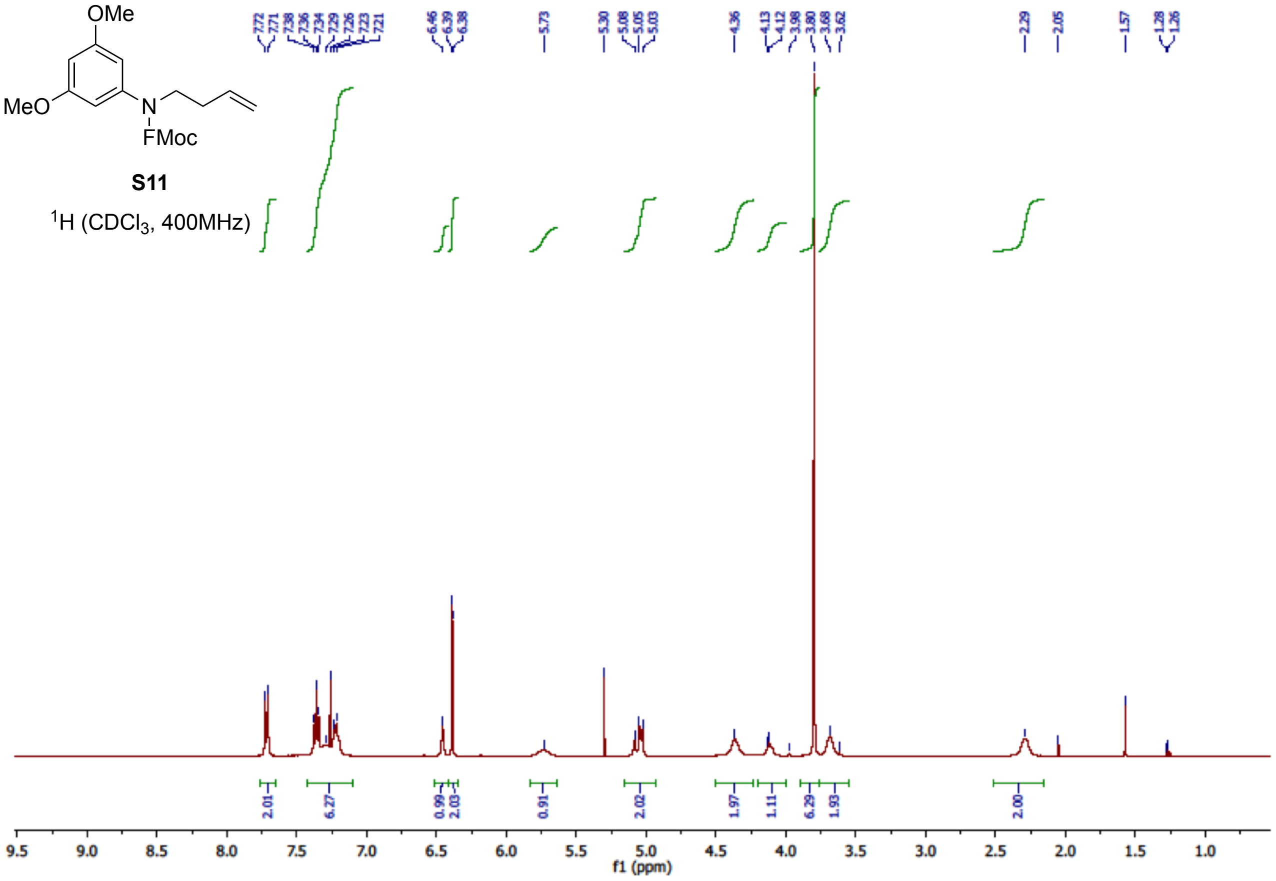

S11

^{1}H ($CDCl_3$, 400MHz)

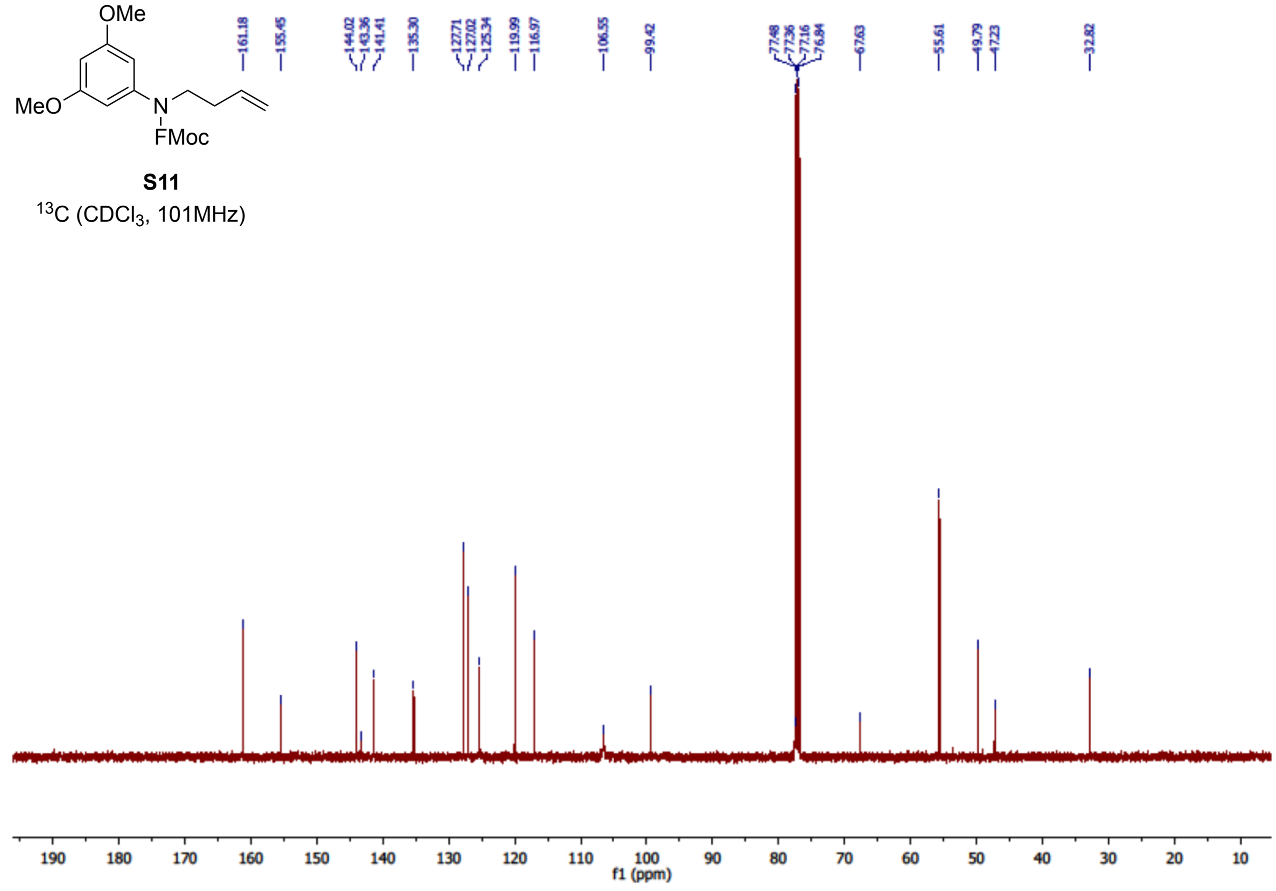

S11

^{13}C ($CDCl_3$, 101MHz)

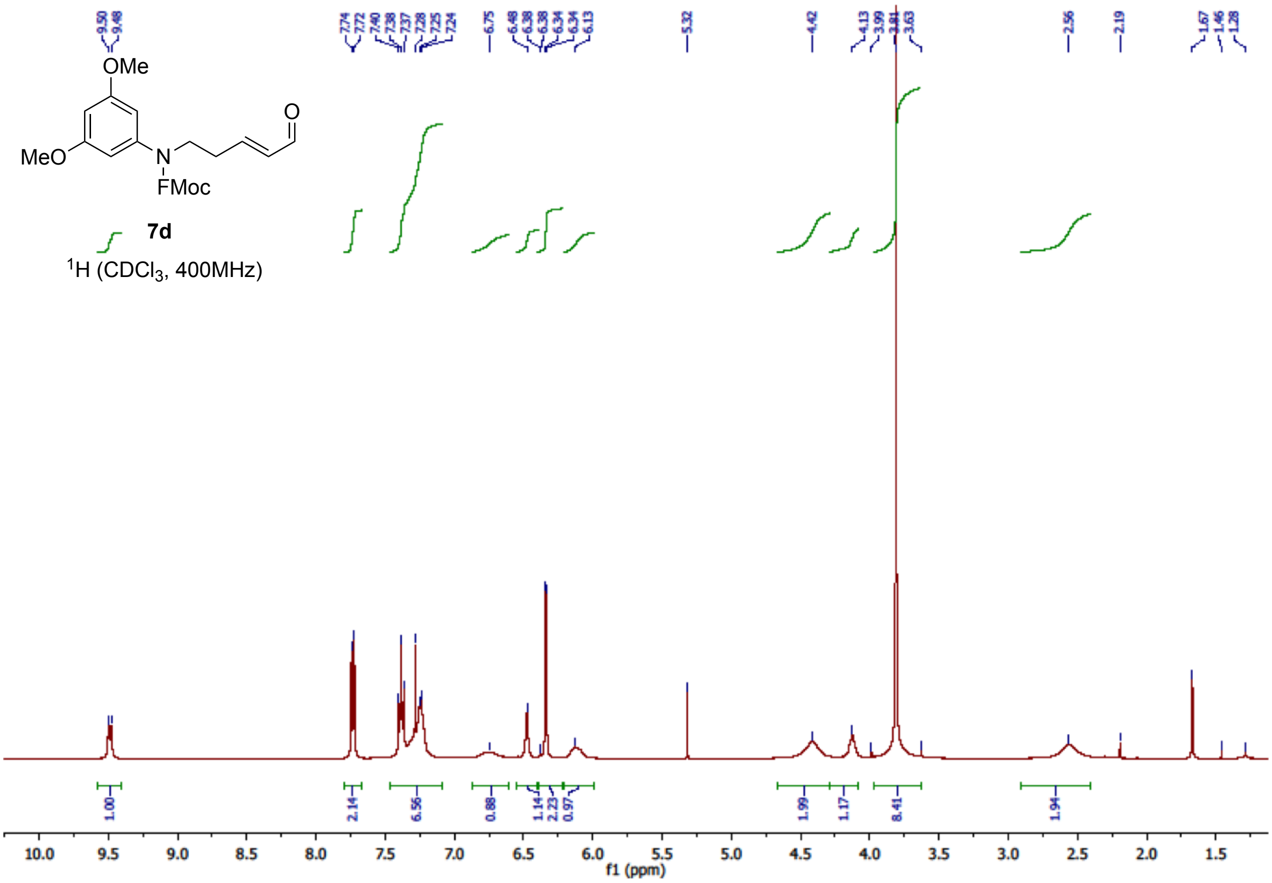
OMe
MeO
N
FMoc
O
7d
1H (CDCl3, 400MHz)
f1 (ppm)

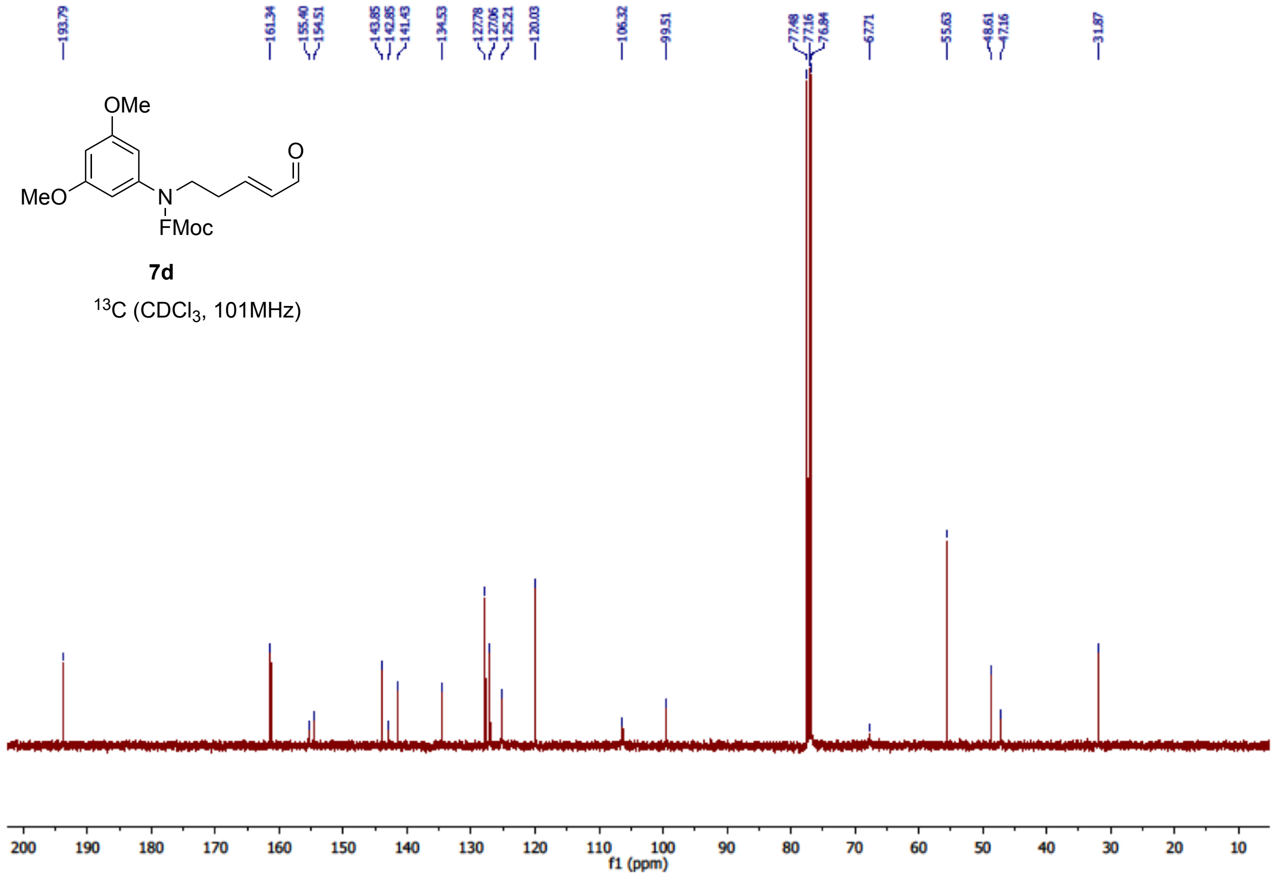
OMe
MeO
N
FMoc
O
7d
13C (CDCl3, 101MHz)
f1 (ppm)

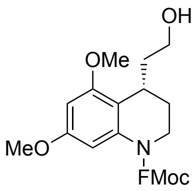

8d

^{1}H ($CDCl_3$, 400MHz)

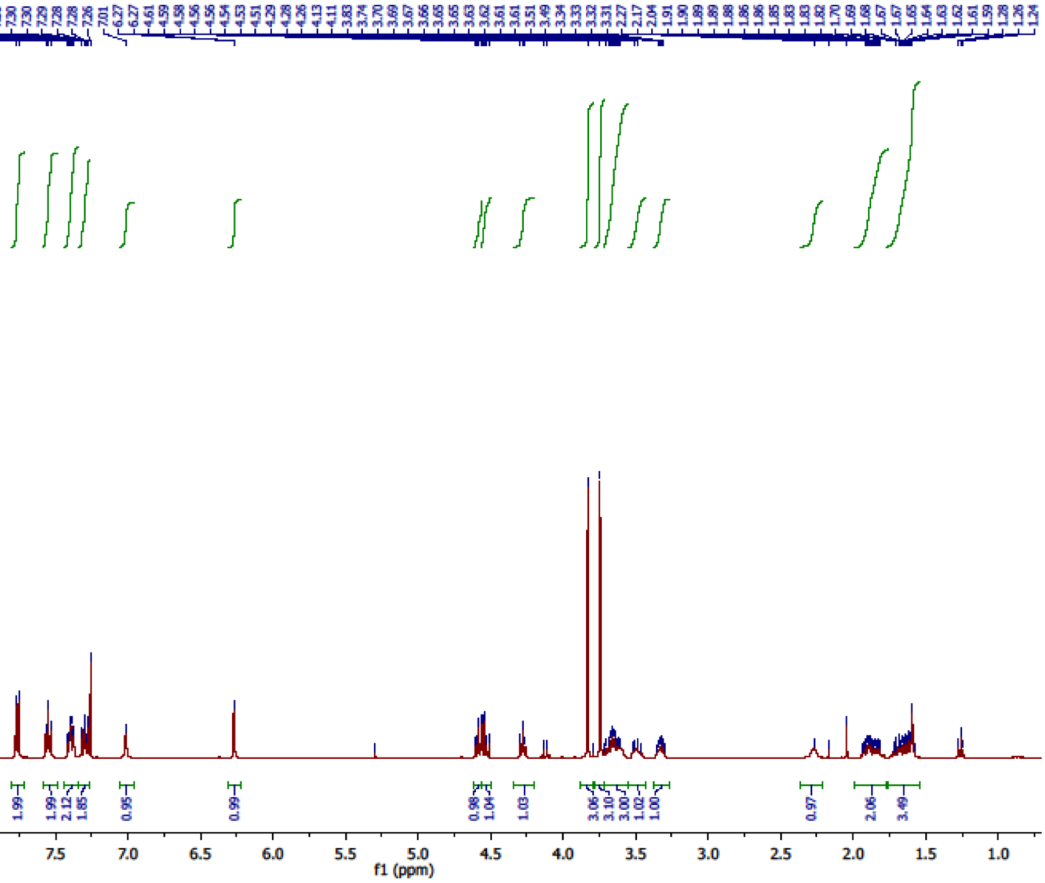

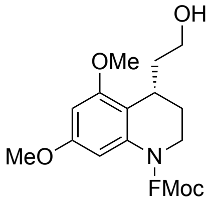

8d

^{13}C ($CDCl_3$, 101MHz)

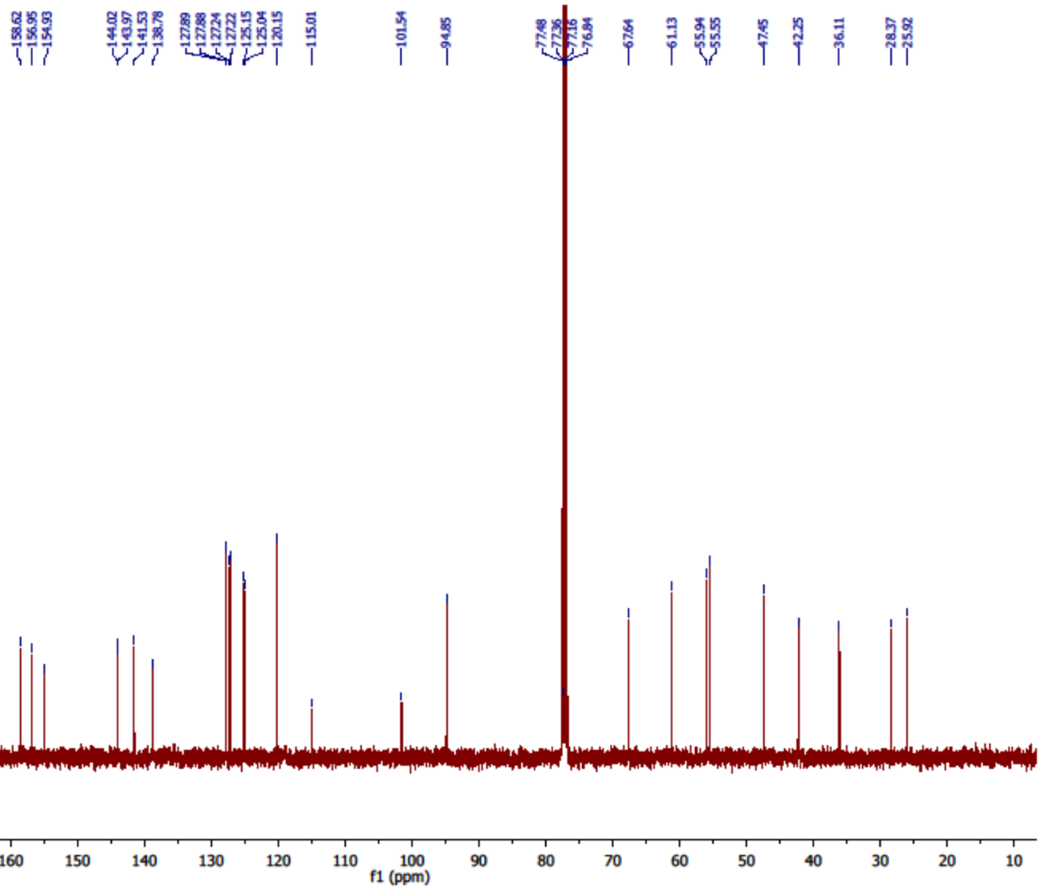

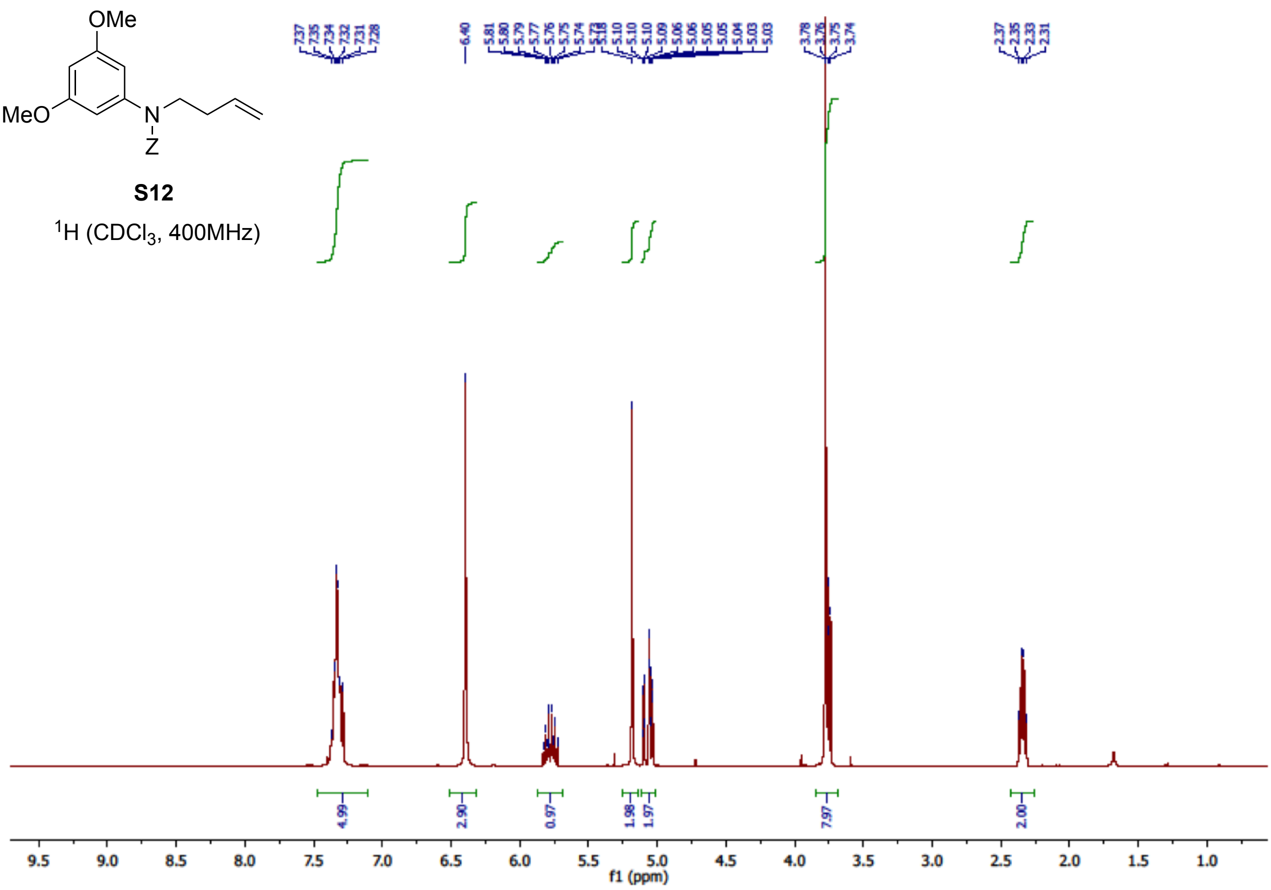

S12

^{1}H ($CDCl_3$, 400MHz)

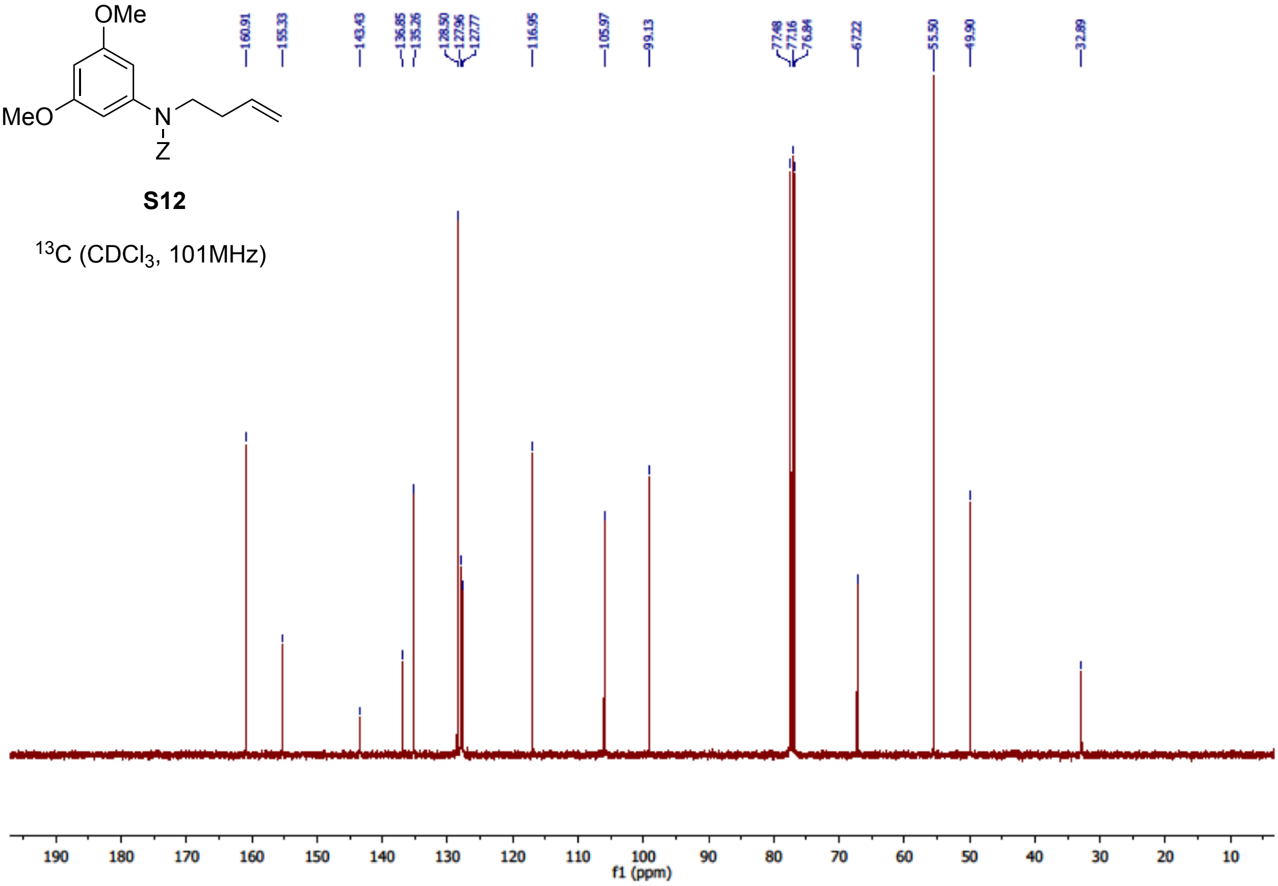

S12

^{13}C ($CDCl_3$, 101MHz)

7e

^{1}H ($CDCl_3$, 400MHz)

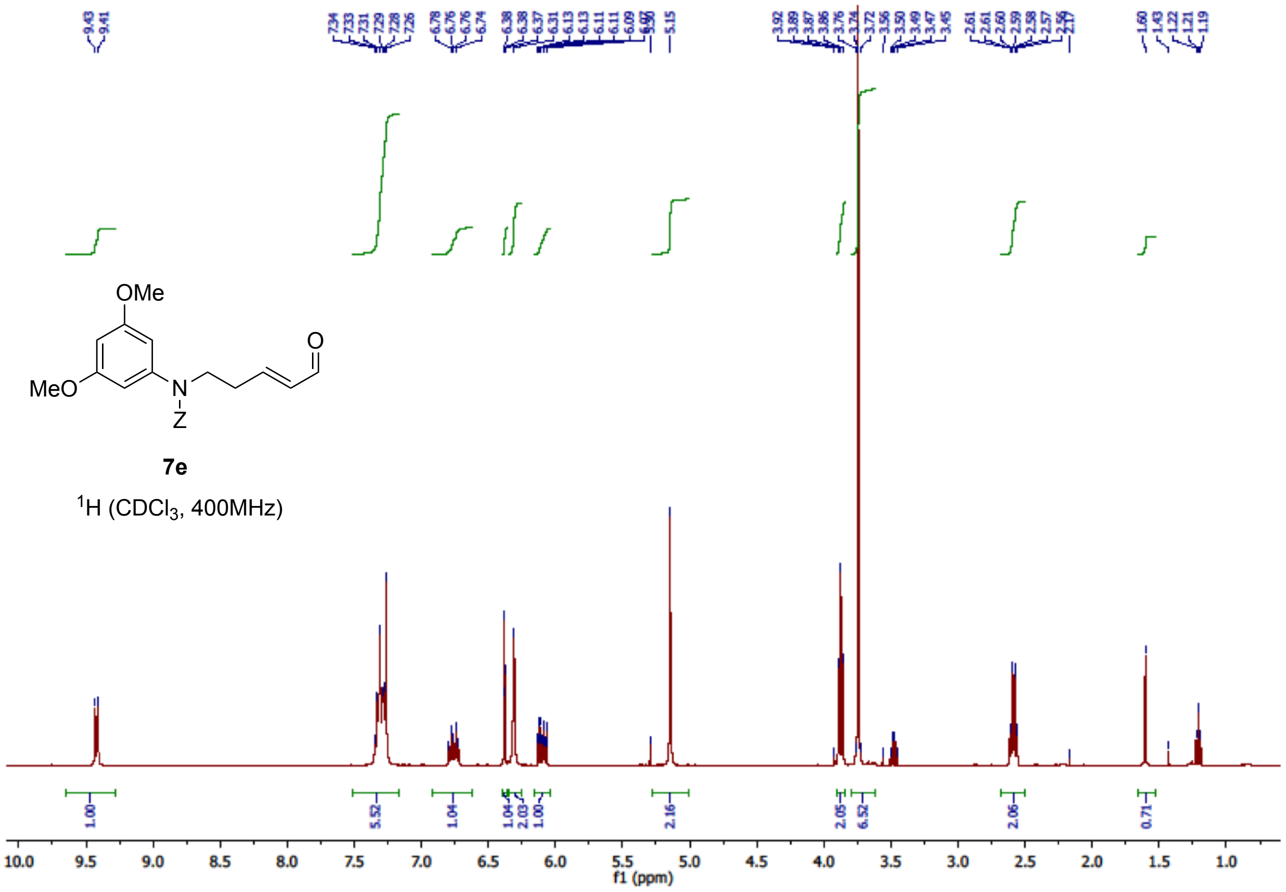

7e

^{13}C ($CDCl_3$, 101MHz)

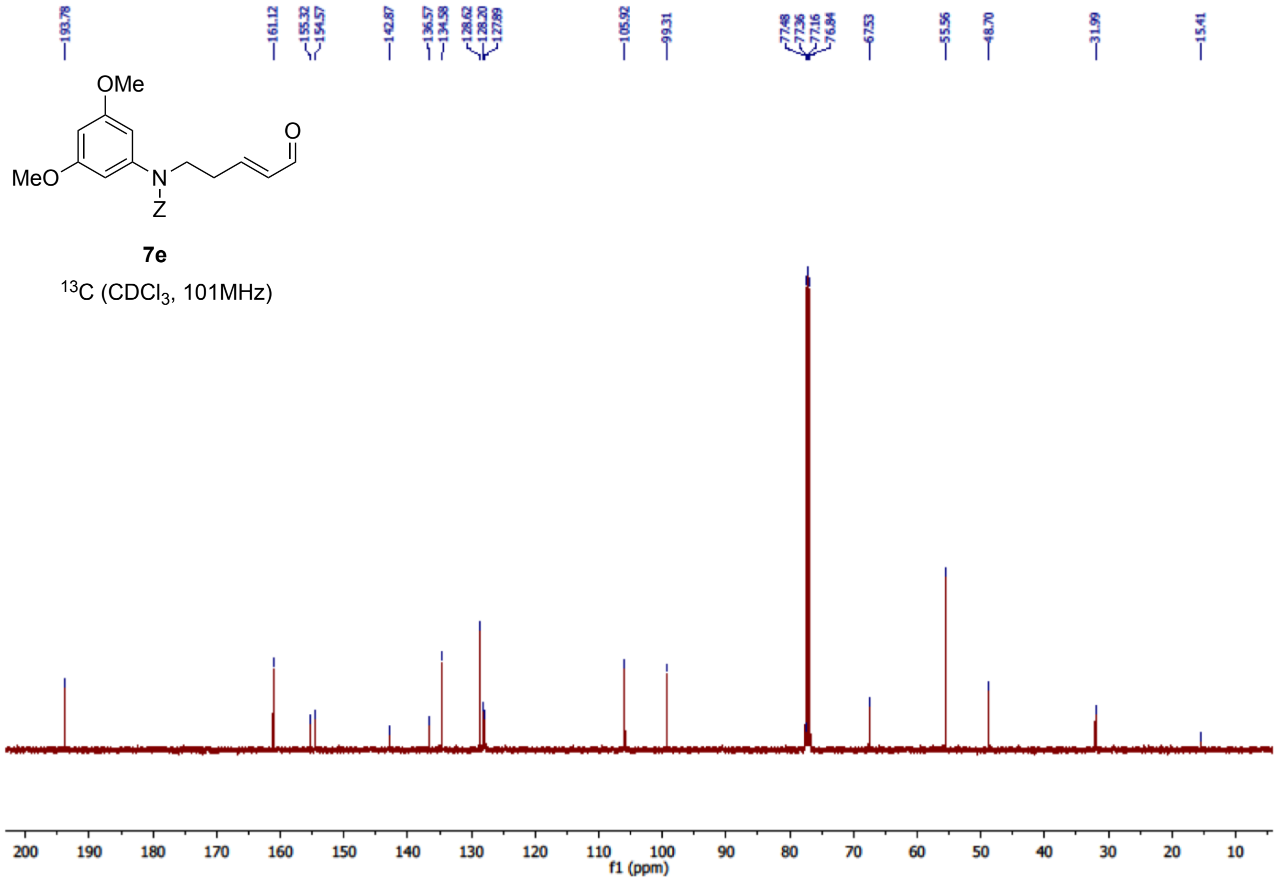

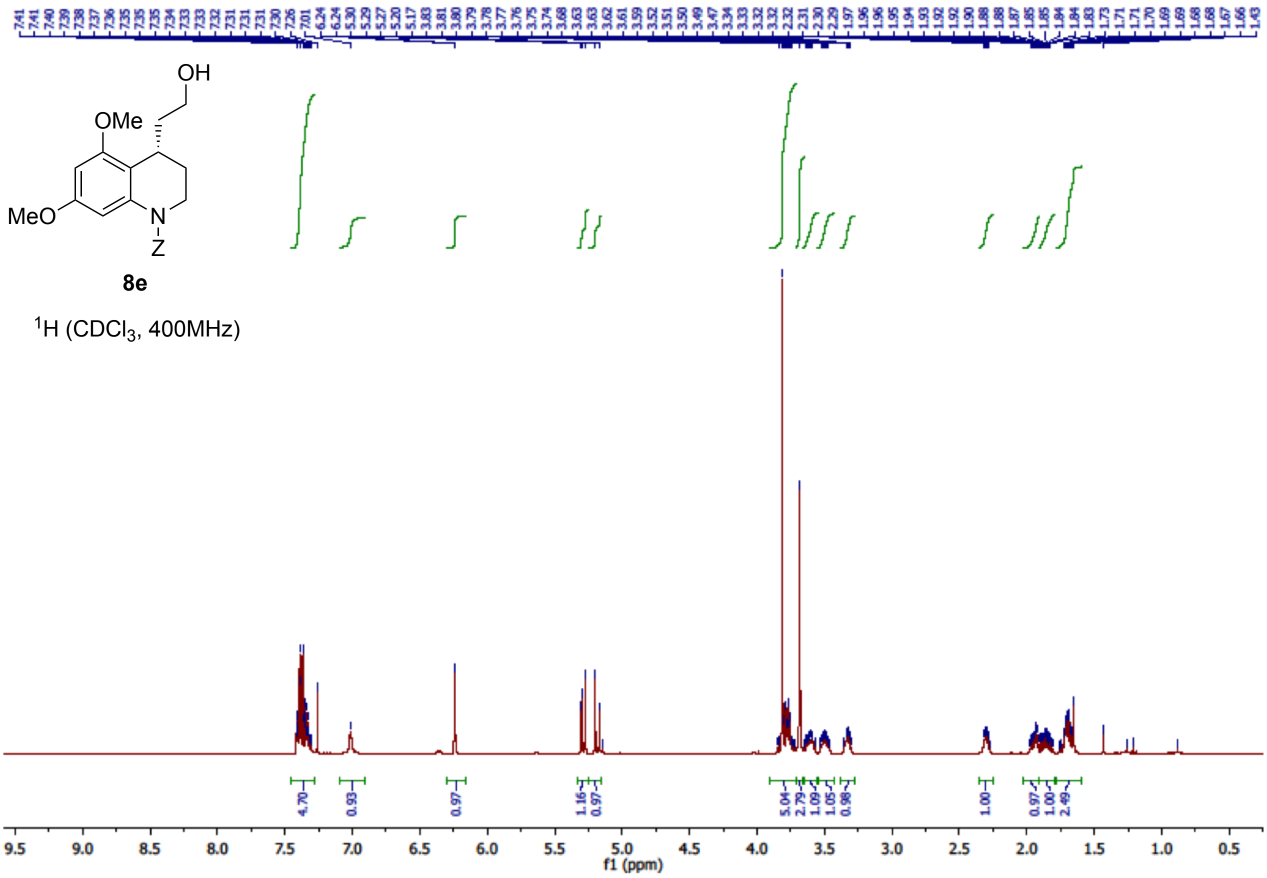
OH
OMe
MeO
N
Z
8e
1H (CDCl3, 400MHz)
4.70
0.93
0.97
1.16
0.97
5.04
2.79
1.09
1.05
0.98
1.00
0.97
1.00
2.49
f1 (ppm)

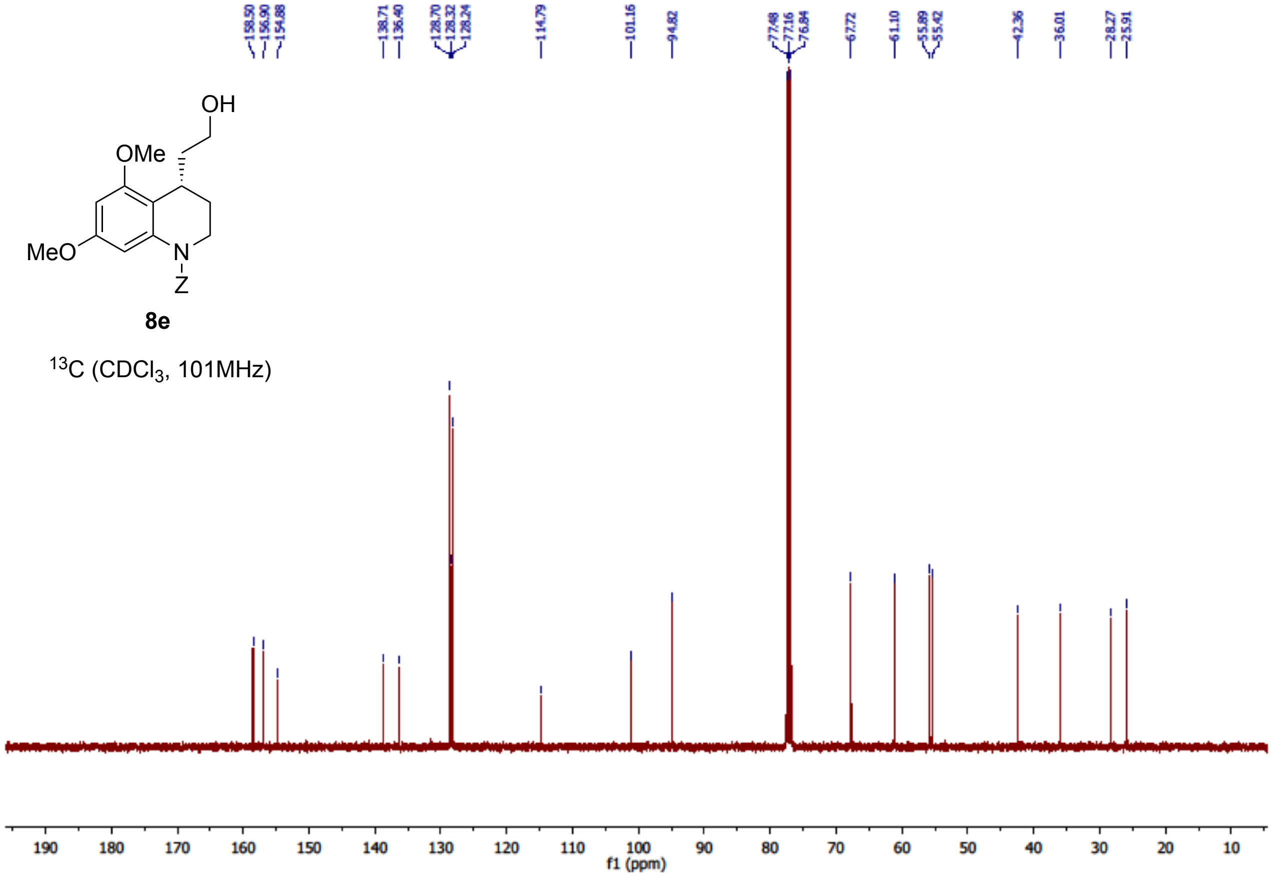
158.50
156.90
154.88
138.71
136.40
128.70
128.32
128.24
114.79
101.16
94.82
77.48
77.16
76.84
67.72
61.10
55.89
55.42
42.36
36.01
28.27
25.91
OH
OMe
MeO
N
Z
8e
13C (CDCl3, 101MHz)
f1 (ppm)

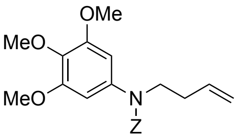

S13

^{1}H ($CDCl_3$, 400MHz)

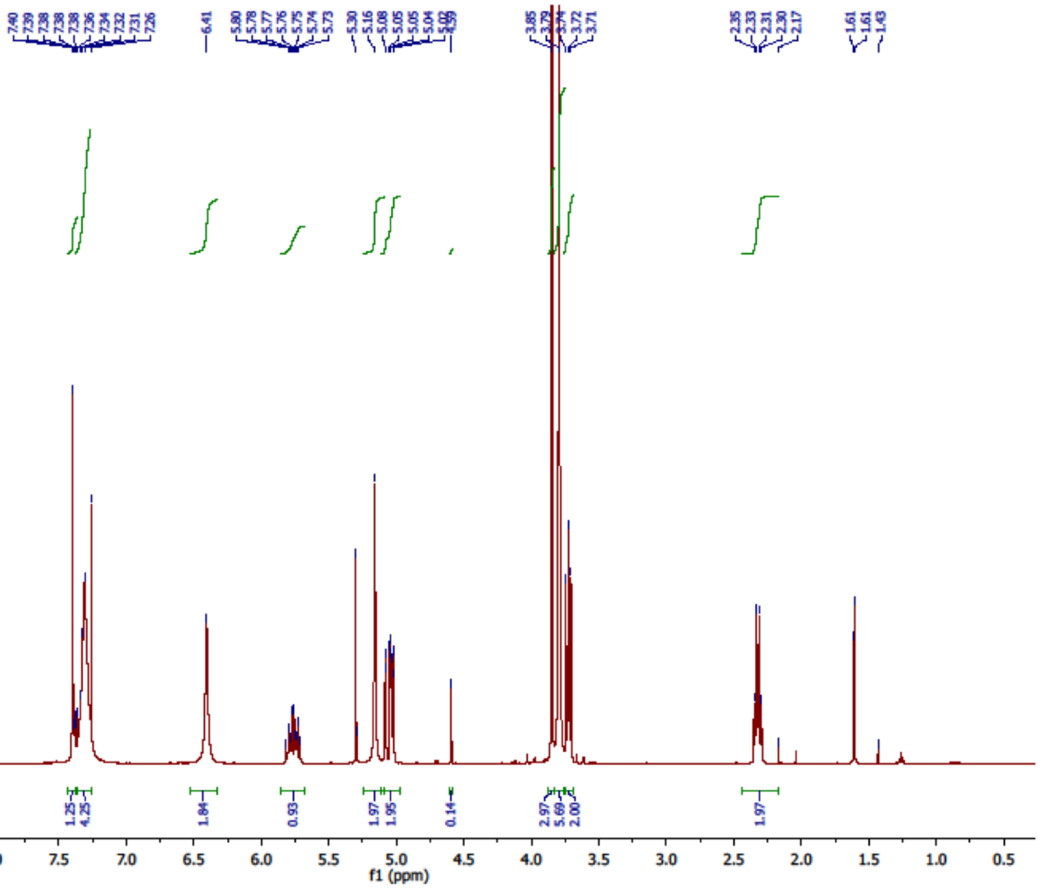

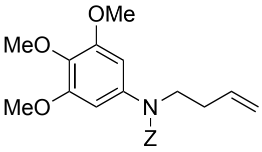

S13

^{13}C ($CDCl_3$, 101MHz)

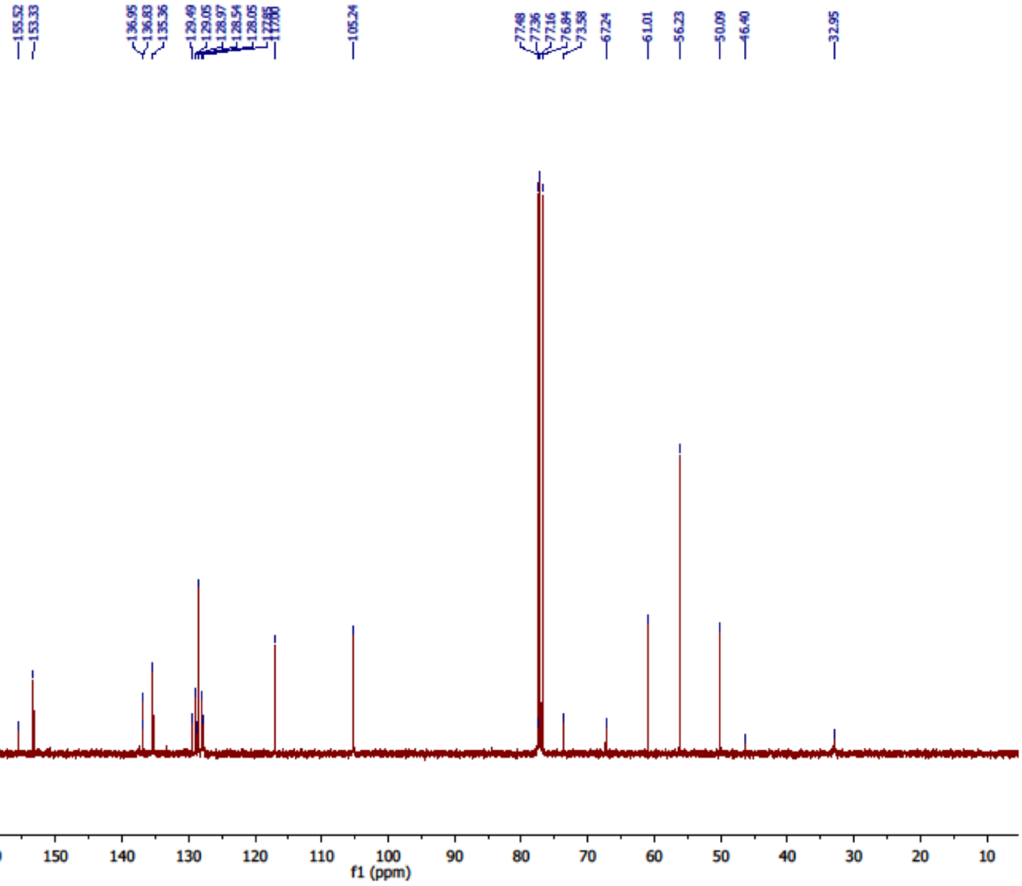

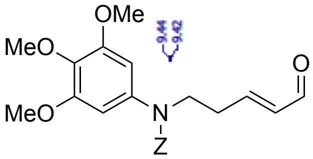

7f

^{1}H (CDCl$_3$, 400MHz)

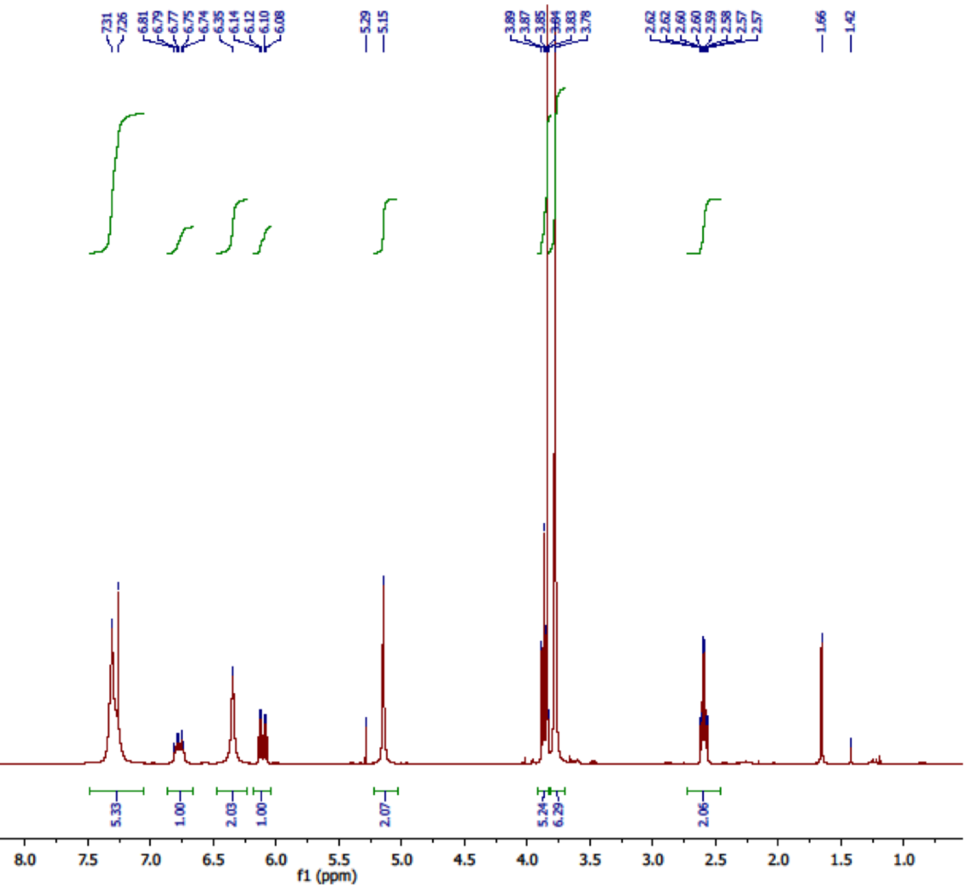

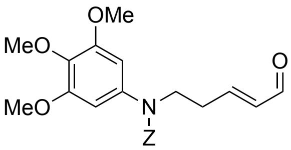

7f

^{13}C (CDCl$_3$, 101MHz)

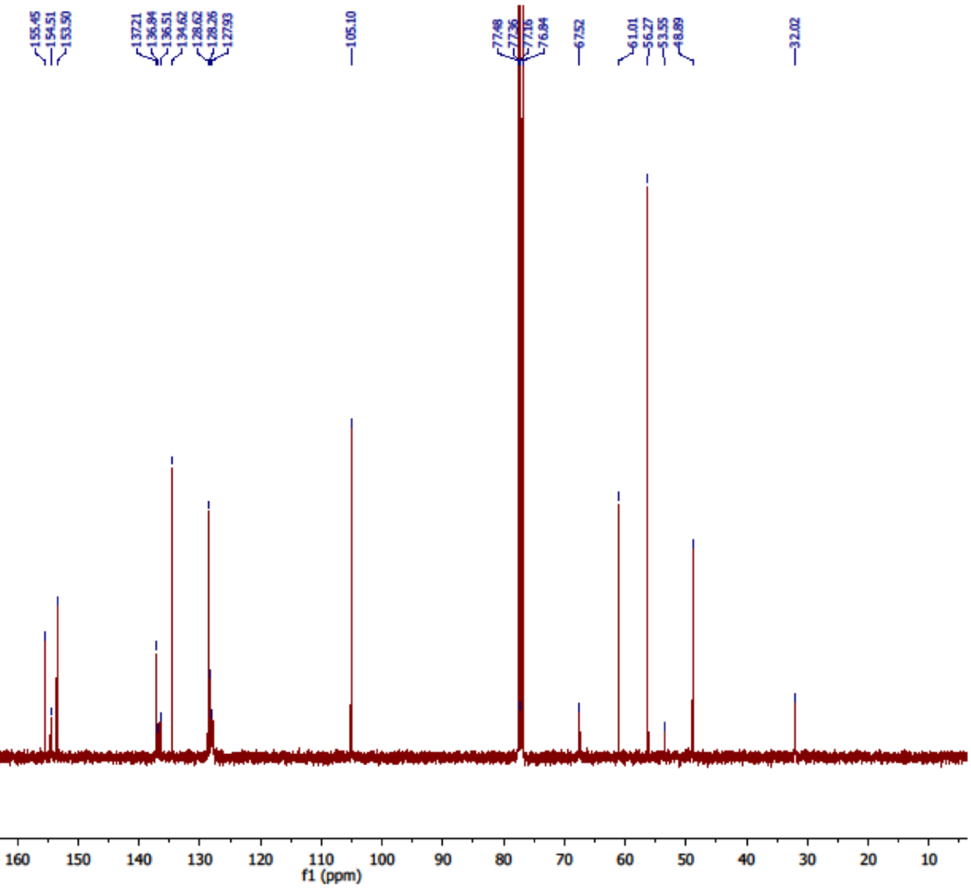

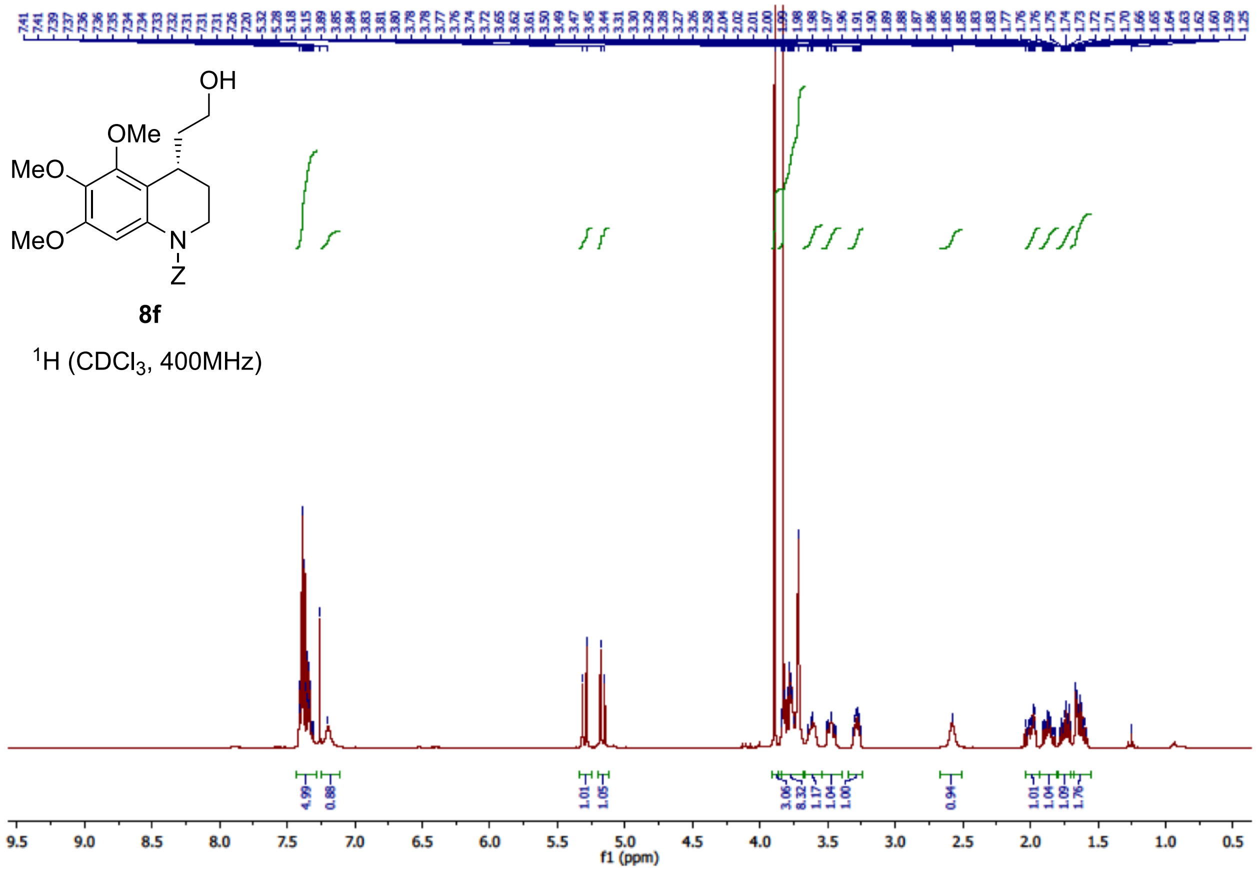
OH
OMe
MeO
MeO
N
Z
8f
^{1}H (CDCl$_3$, 400MHz)
f1 (ppm)

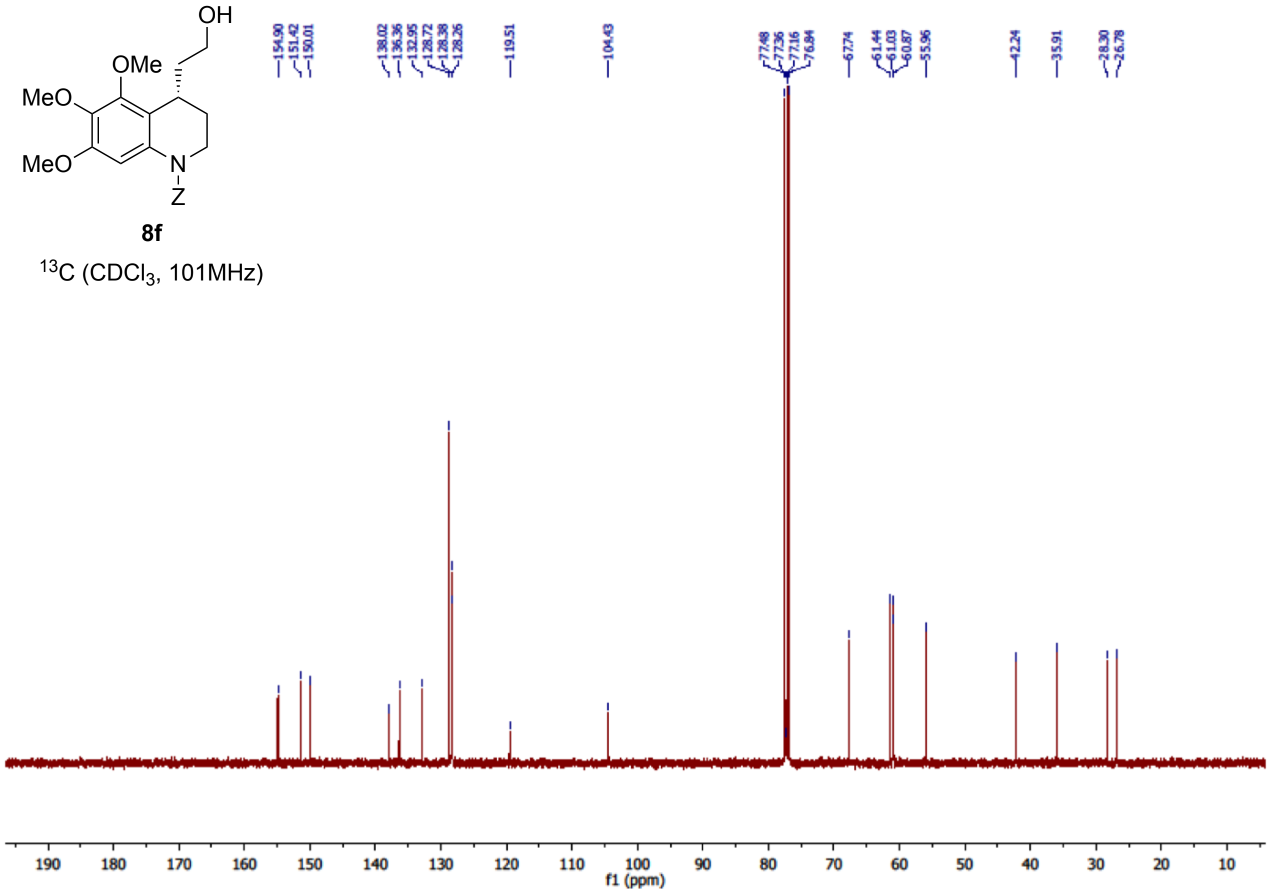
OH
OMe
MeO
MeO
N
Z
8f
^{13}C (CDCl$_3$, 101MHz)
154.90
151.42
150.01
138.02
136.36
132.95
128.72
128.38
128.26
119.51
104.43
77.48
77.36
77.16
76.84
67.74
61.44
61.03
60.87
55.96
42.24
35.91
28.30
26.78
f1 (ppm)

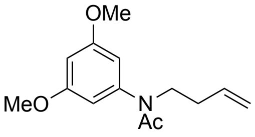

S14

^{1}H ($CDCl_3$, 400MHz)

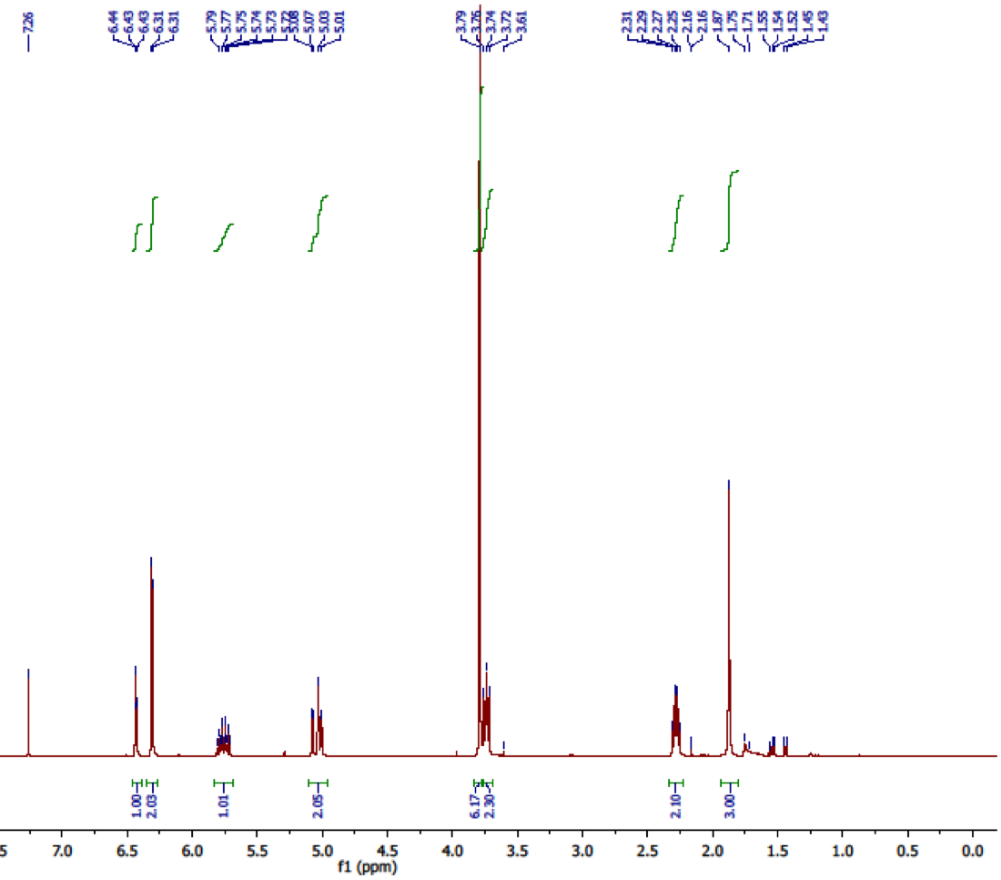

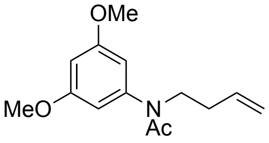

S14

^{13}C ($CDCl_3$, 101MHz)

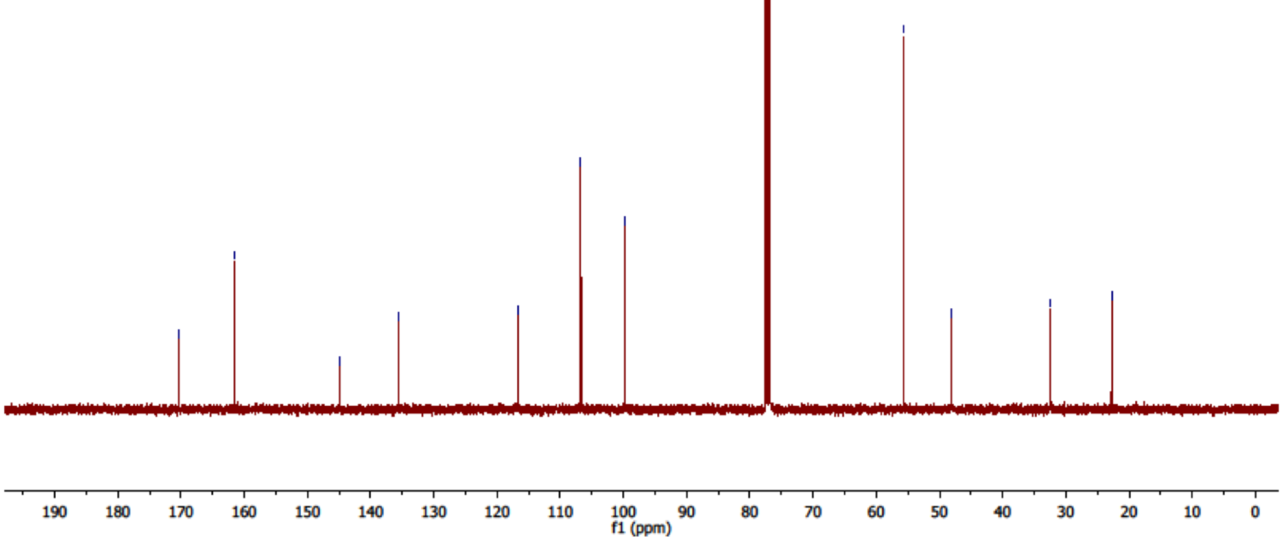

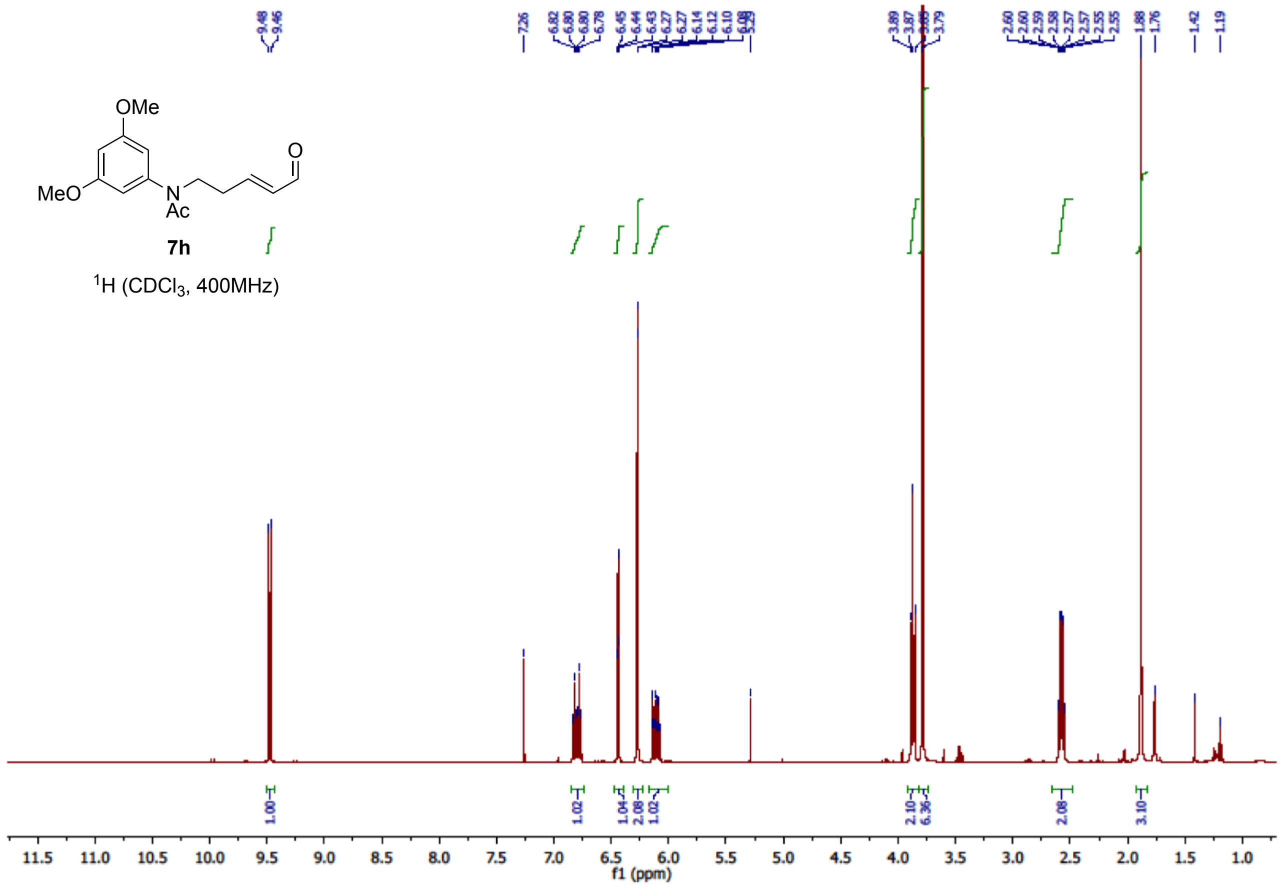
OMe
MeO
N
Ac
O
7h
^{1}H (CDCl$_3$, 400MHz)
f1 (ppm)

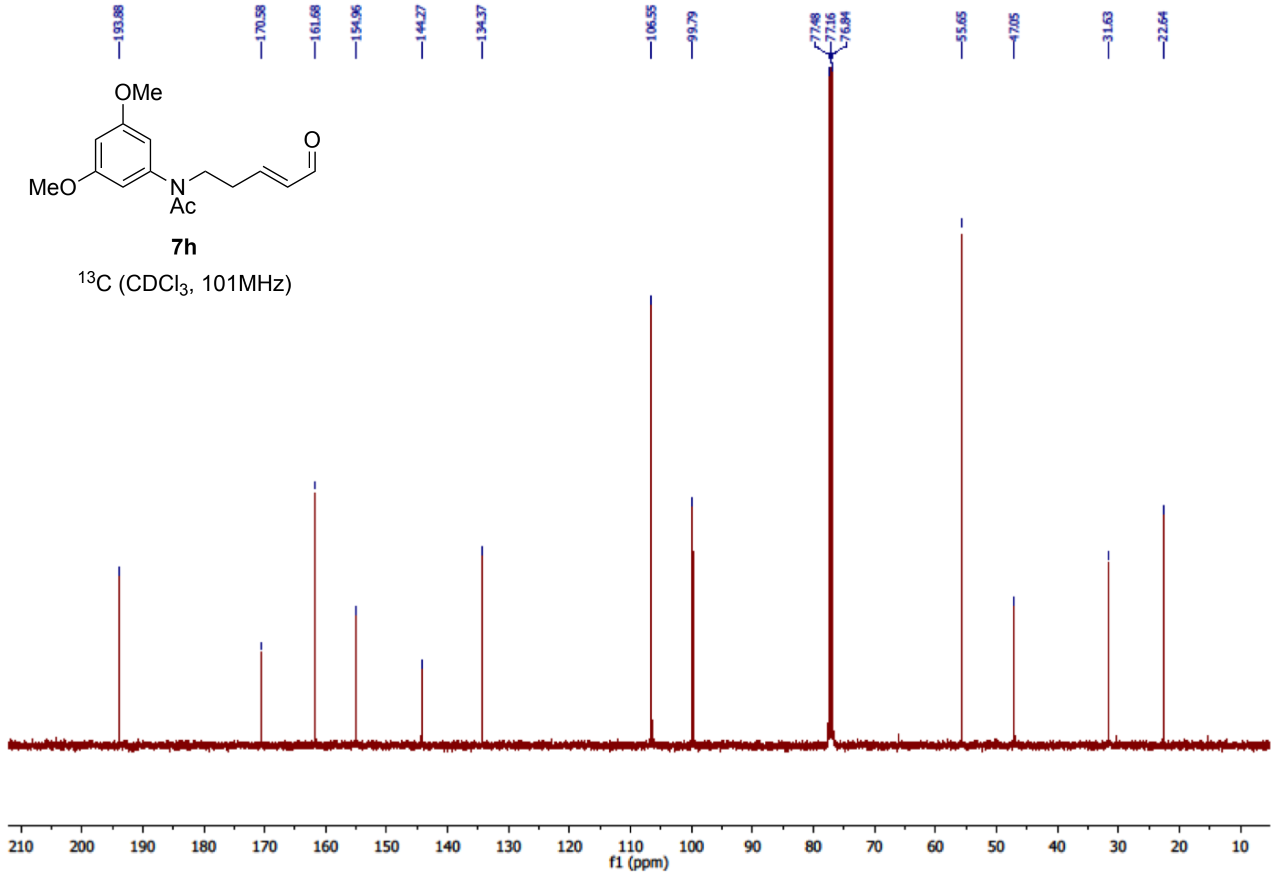
OMe
MeO
N
Ac
O
7h
^{13}C (CDCl$_3$, 101MHz)
f1 (ppm)

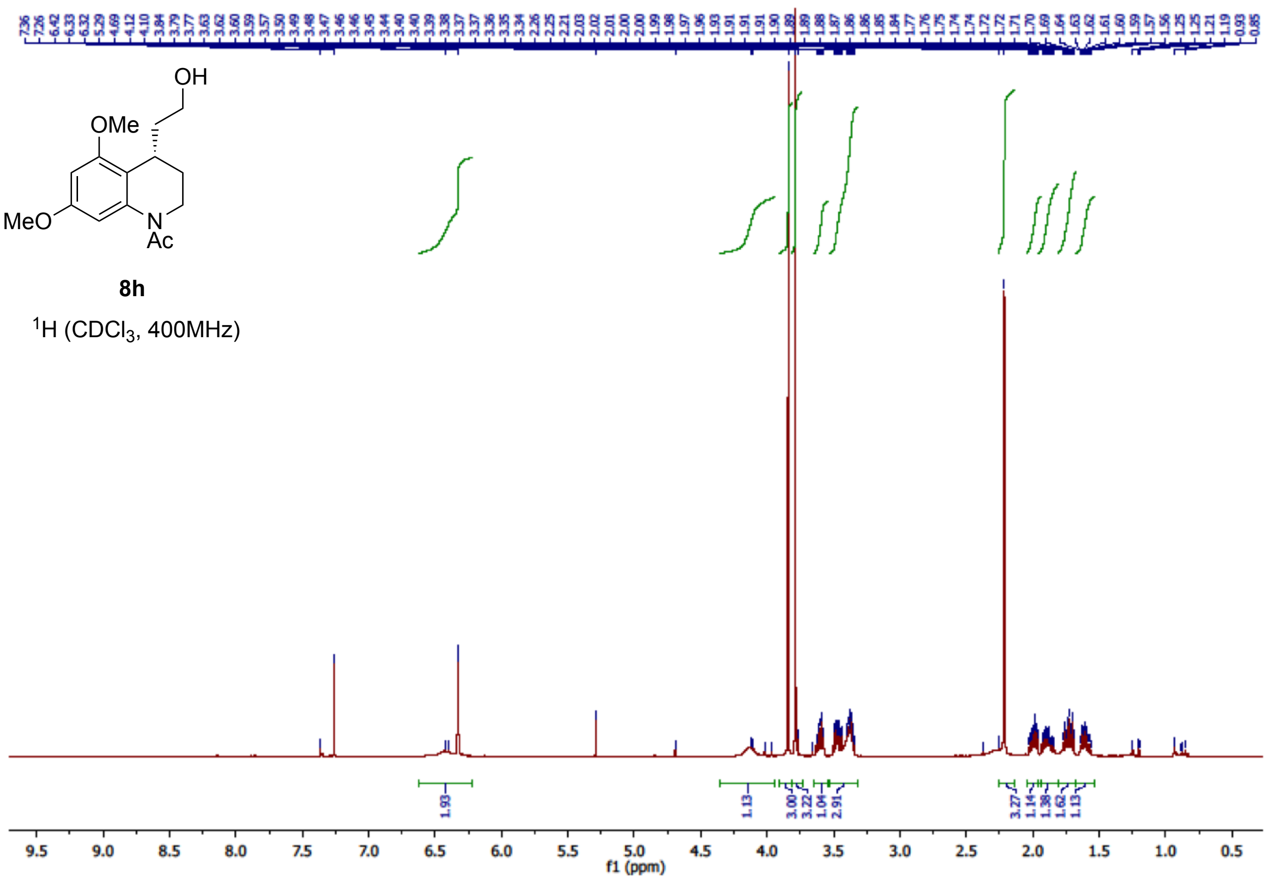

8h

^{1}H ($CDCl_3$, 400MHz)

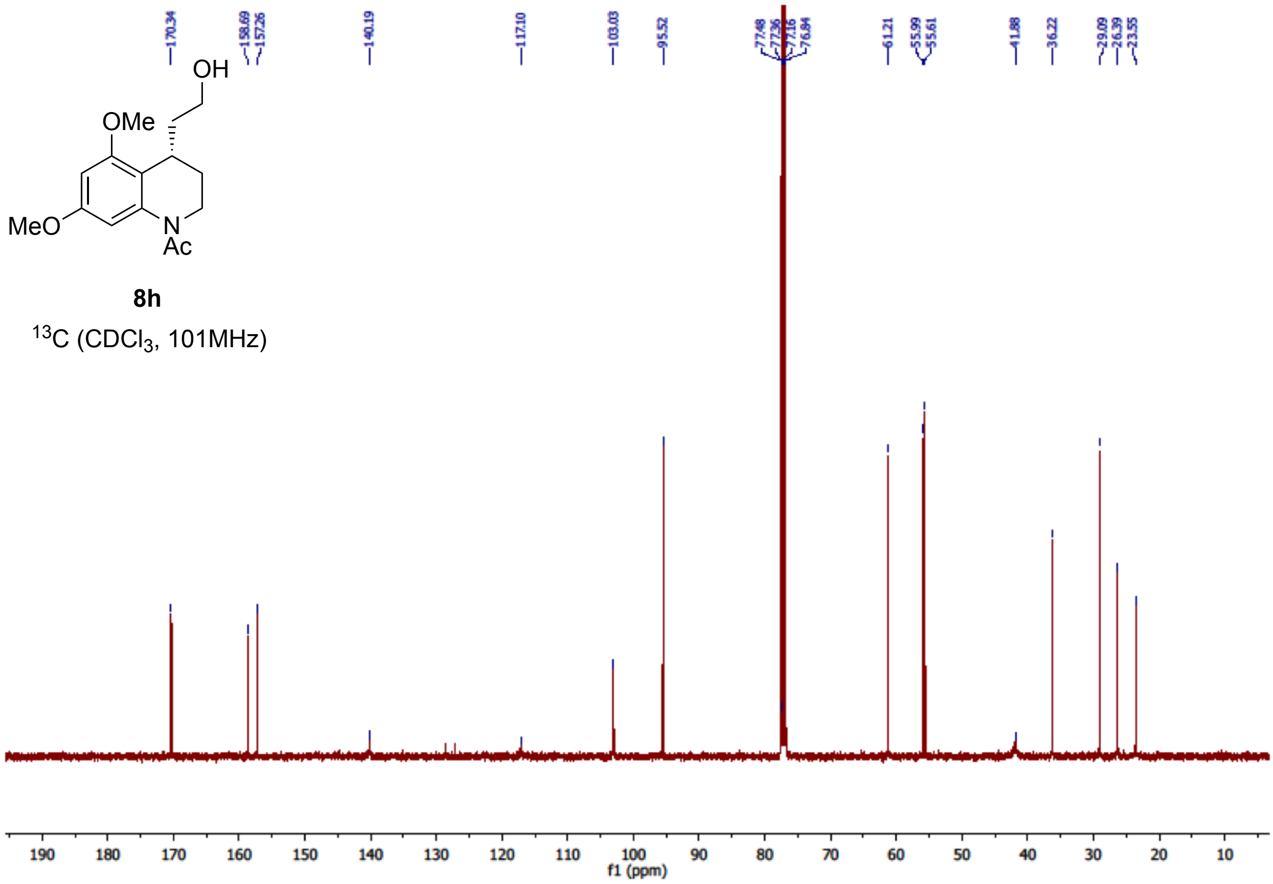

8h

^{13}C ($CDCl_3$, 101MHz)

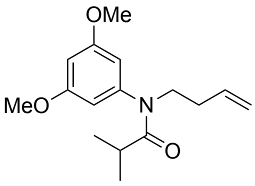

S15

^{1}H ($CDCl_3$, 400MHz)

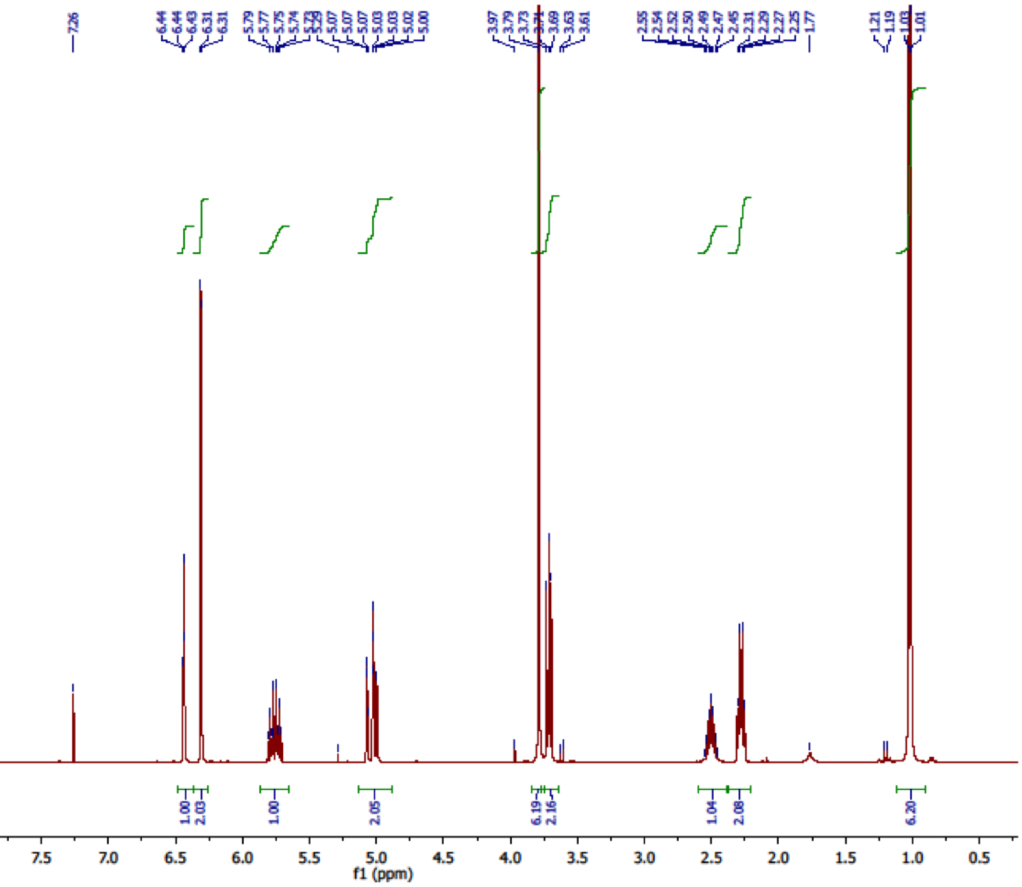

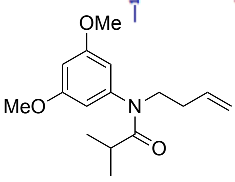

S15

^{13}C ($CDCl_3$, 101MHz)

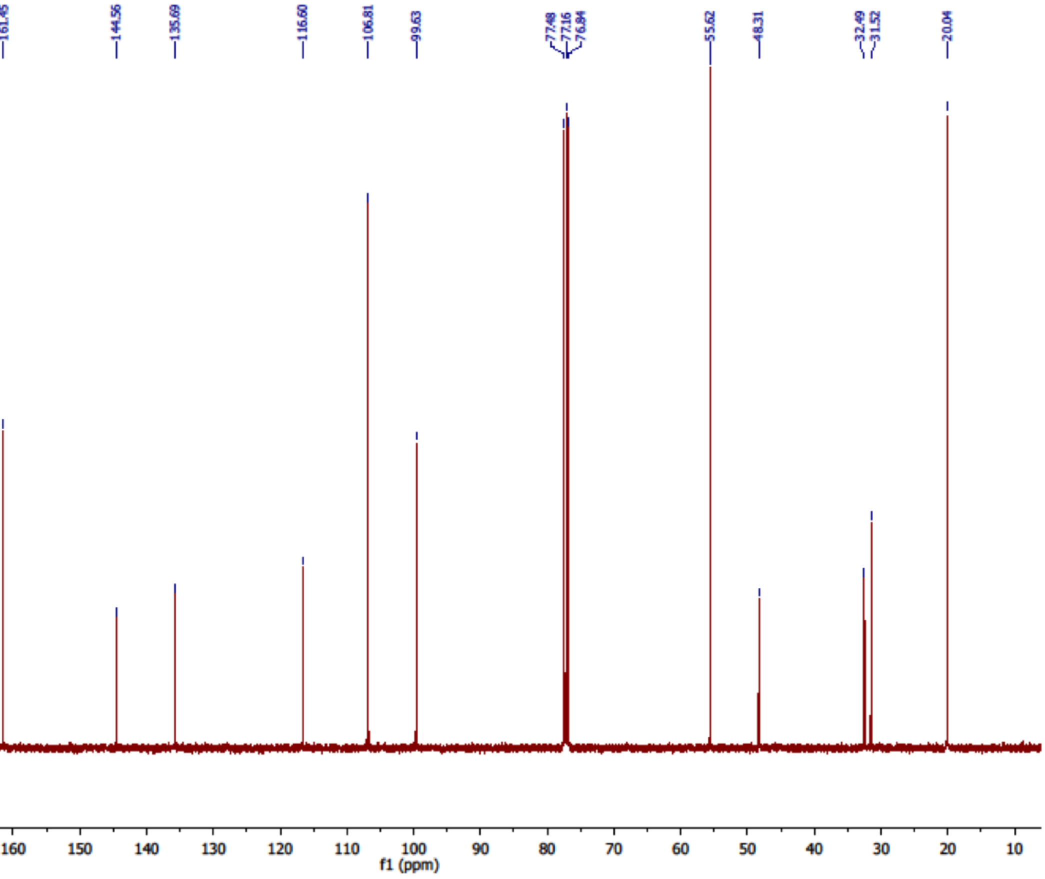

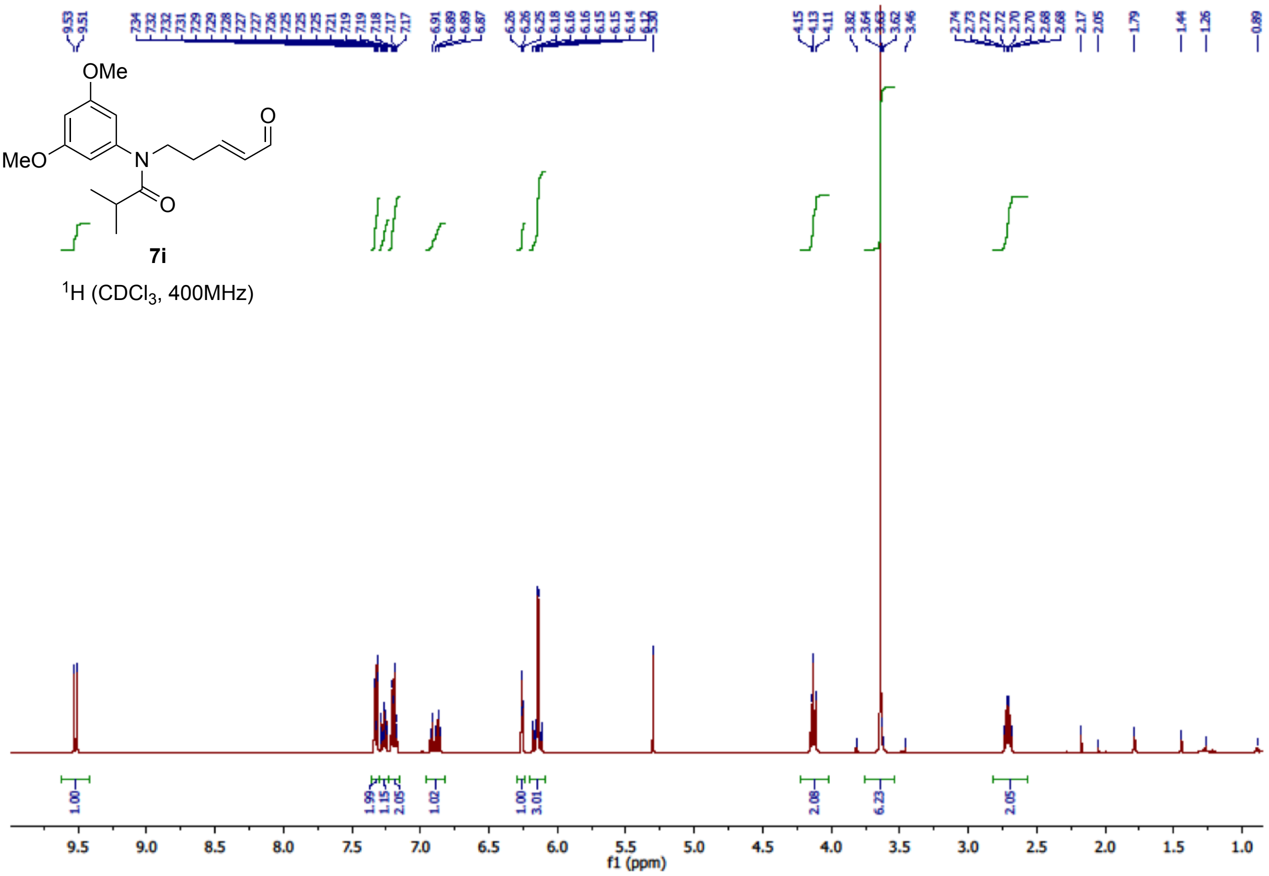

7i

^{1}H (CDCl$_3$, 400MHz)

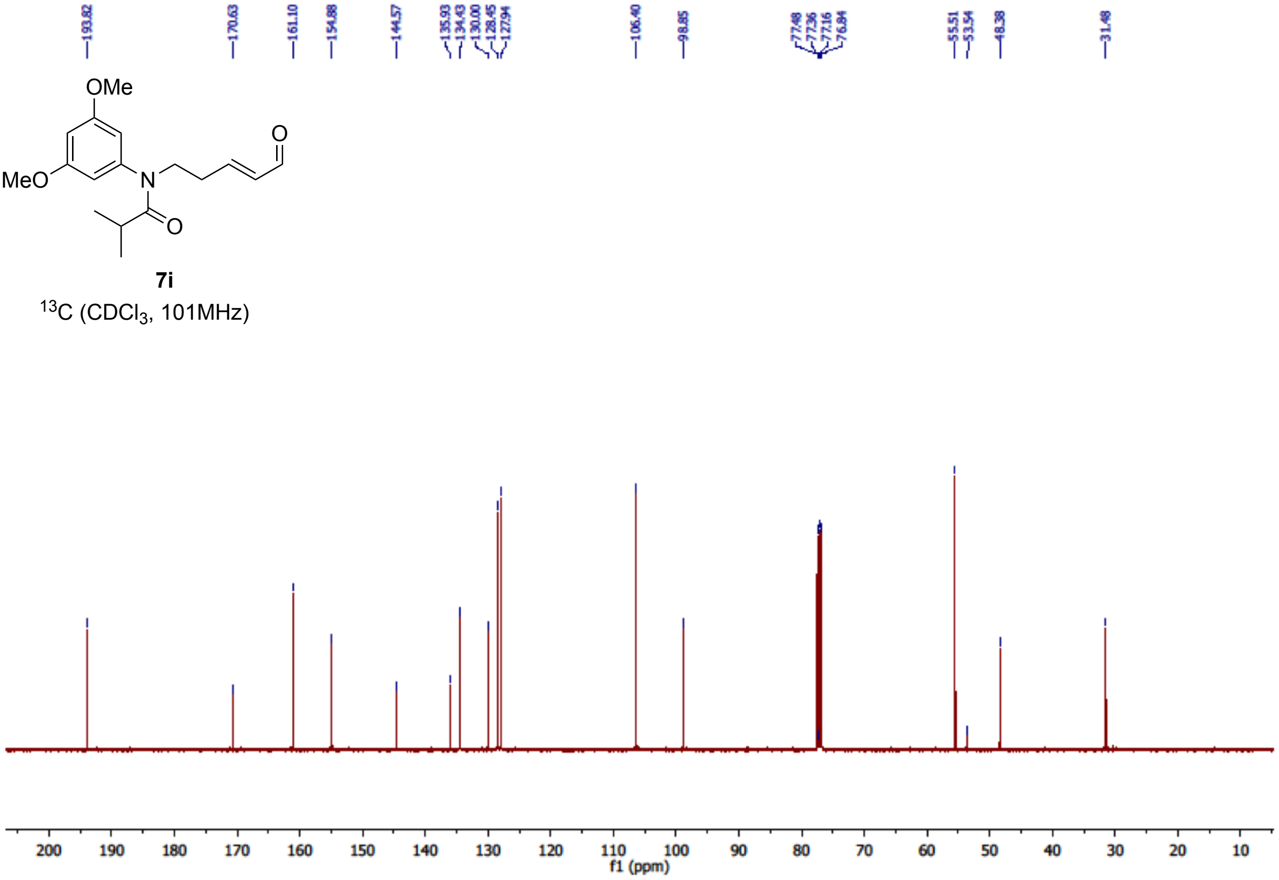

7i

^{13}C (CDCl$_3$, 101MHz)

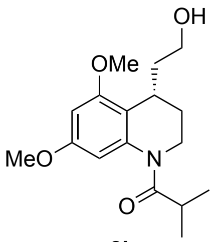

8i

^{1}H ($CDCl_3$, 400MHz)

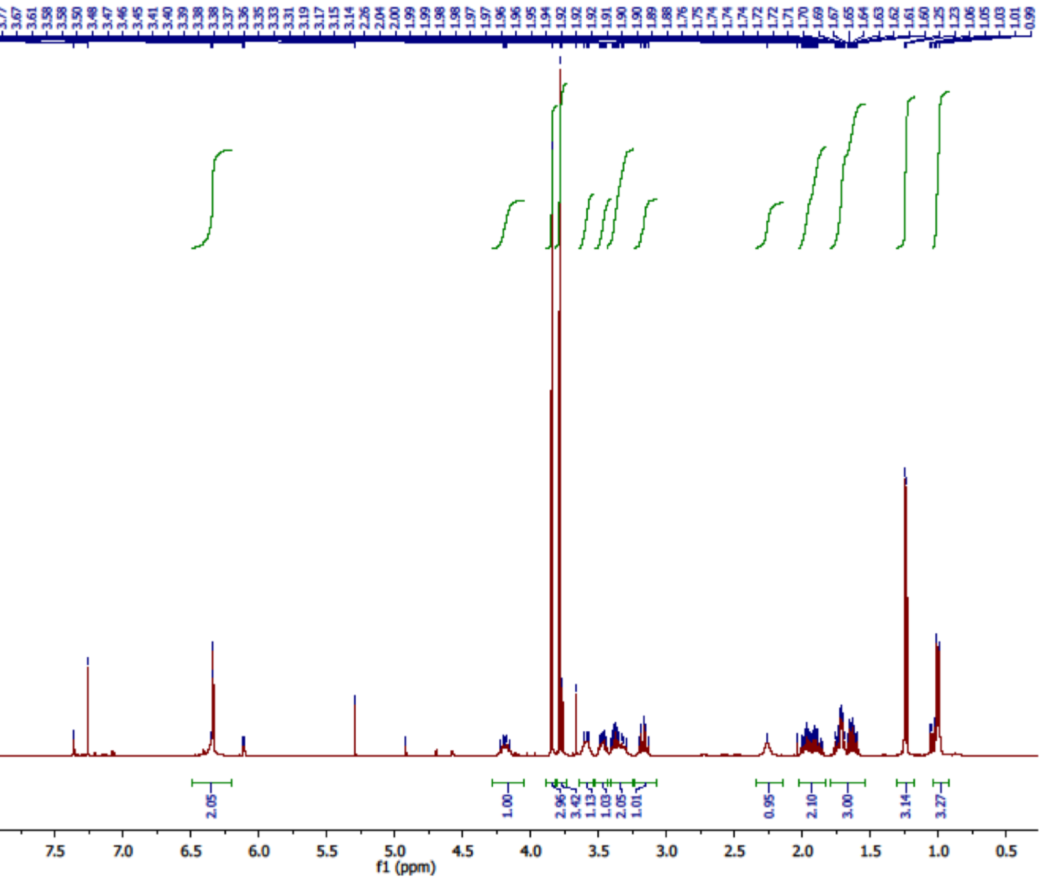

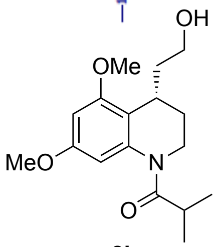

8i

^{13}C ($CDCl_3$, 101MHz)

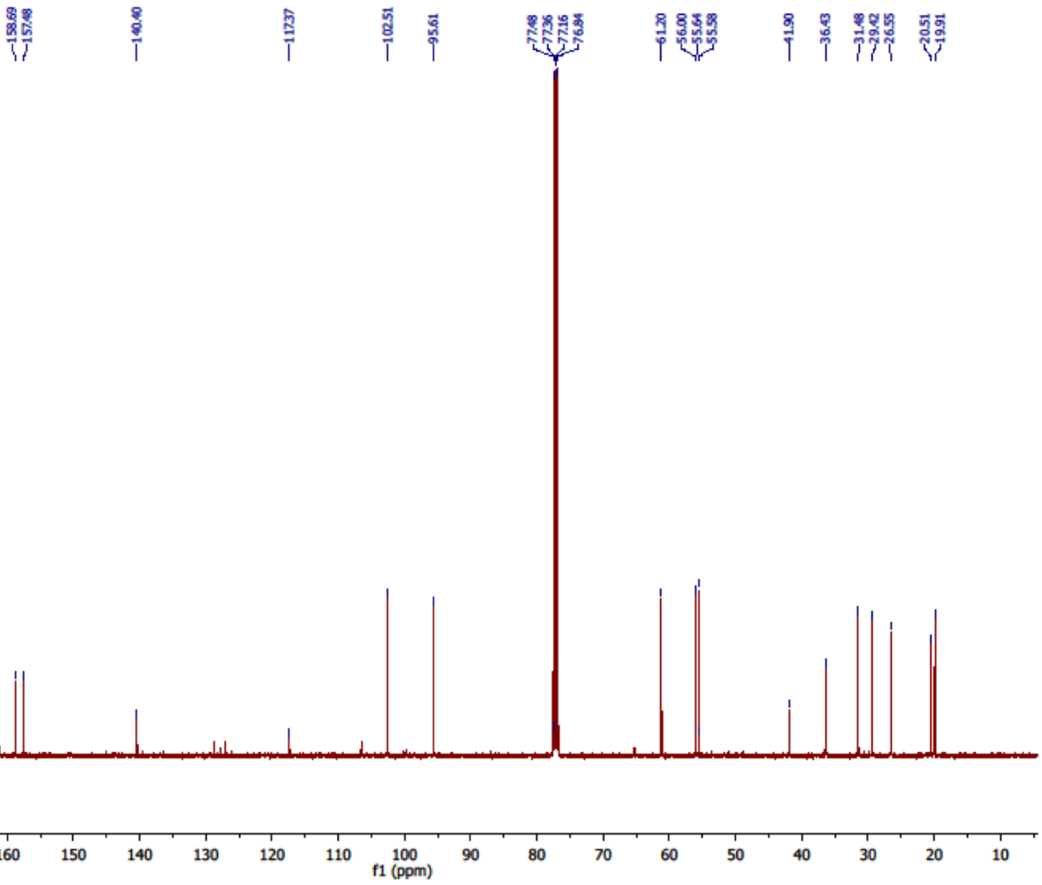

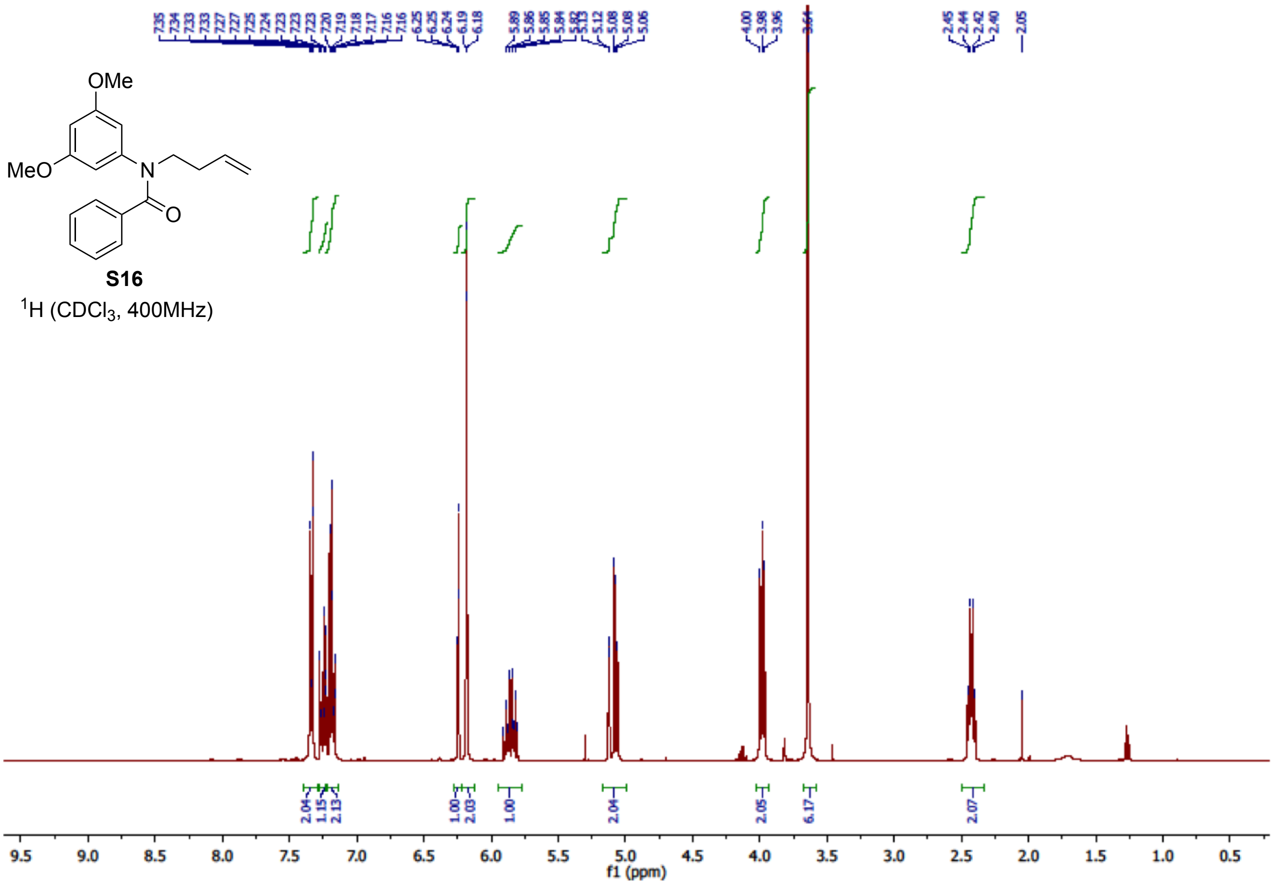
OMe
MeO
N
O
S16
1H (CDCl3, 400MHz)
f1 (ppm)

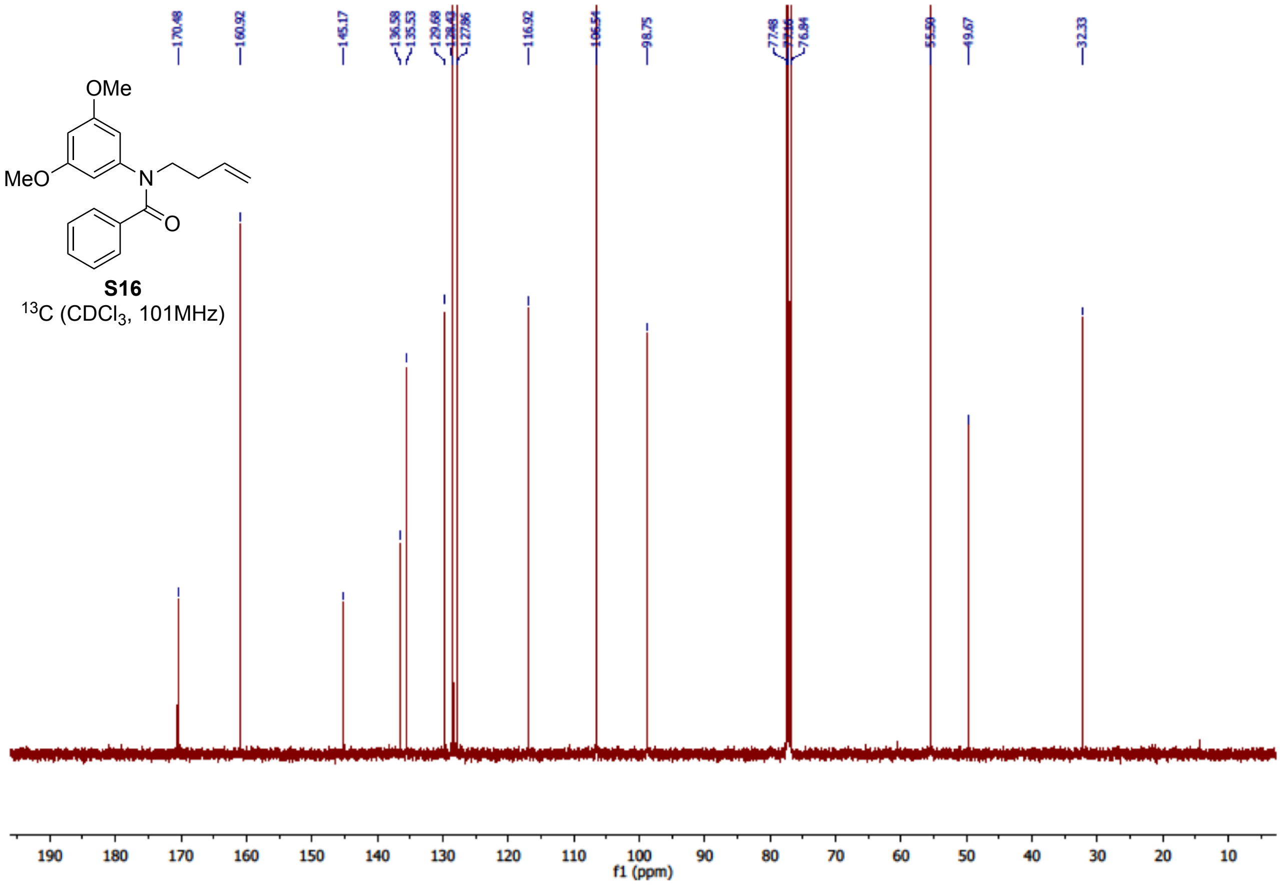
OMe
MeO
N
O
S16
13C (CDCl3, 101MHz)
f1 (ppm)

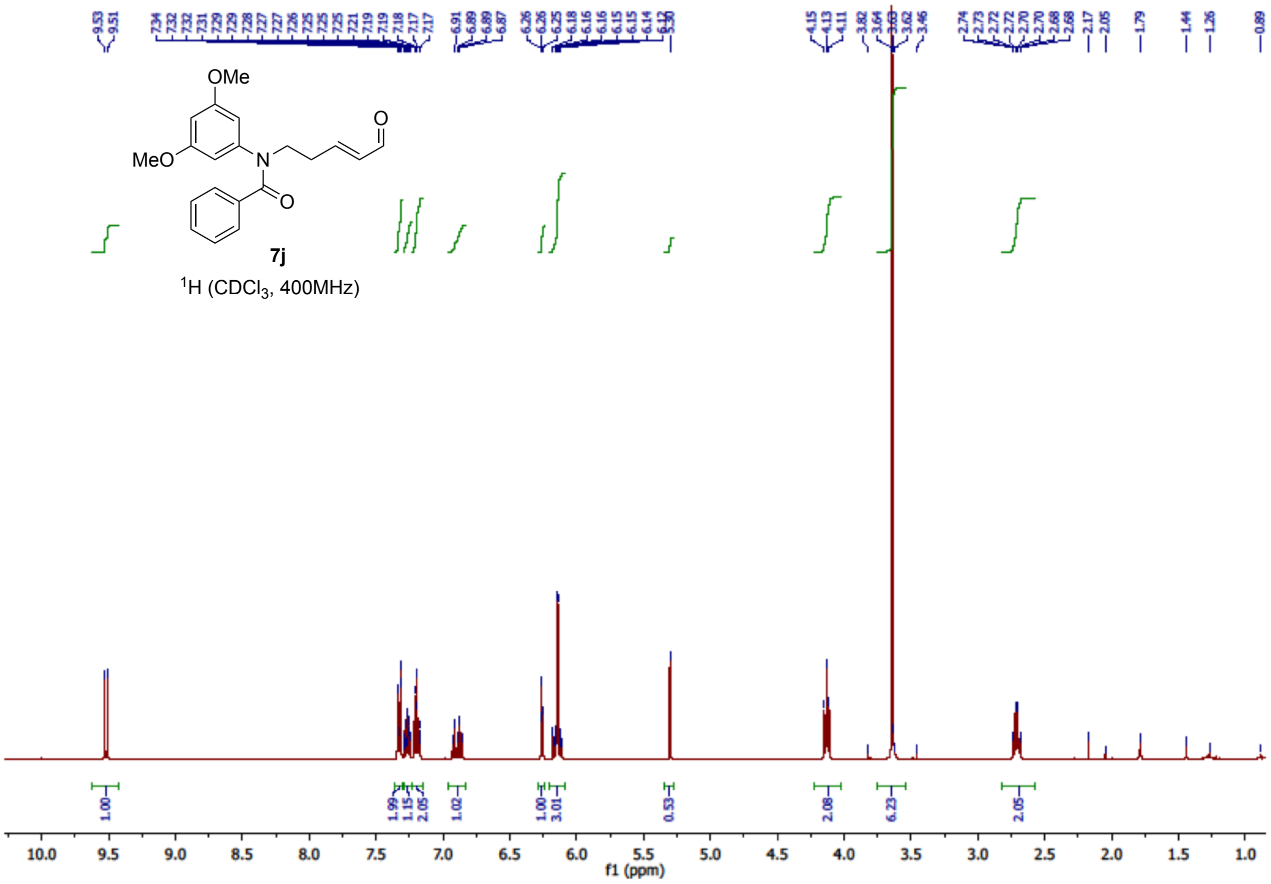
OMe
MeO
N
O
O
7j
^1H (CDCl3, 400MHz)
f1 (ppm)

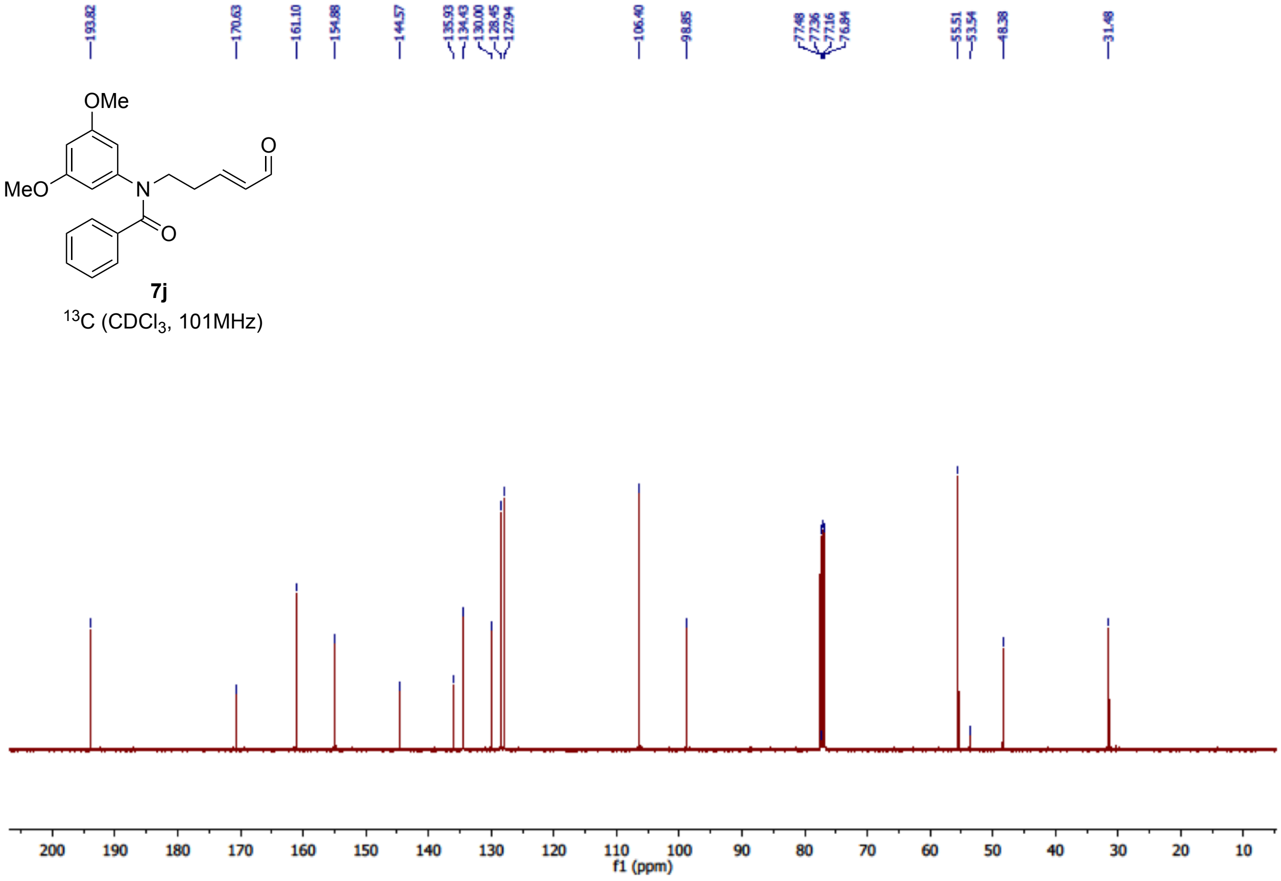
OMe
MeO
N
O
O
7j
^{13}C (CDCl3, 101MHz)
f1 (ppm)

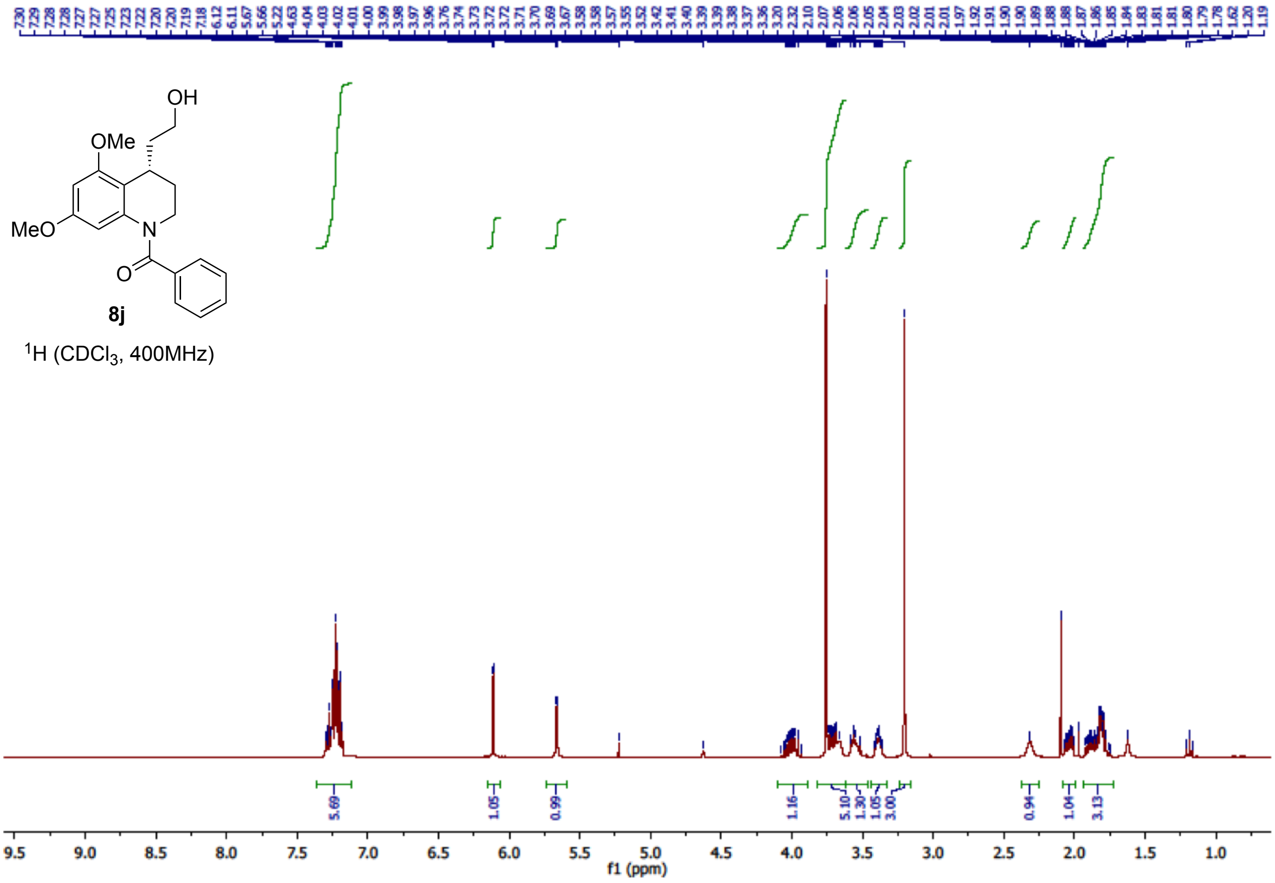

8j

^{1}H ($CDCl_3$, 400MHz)

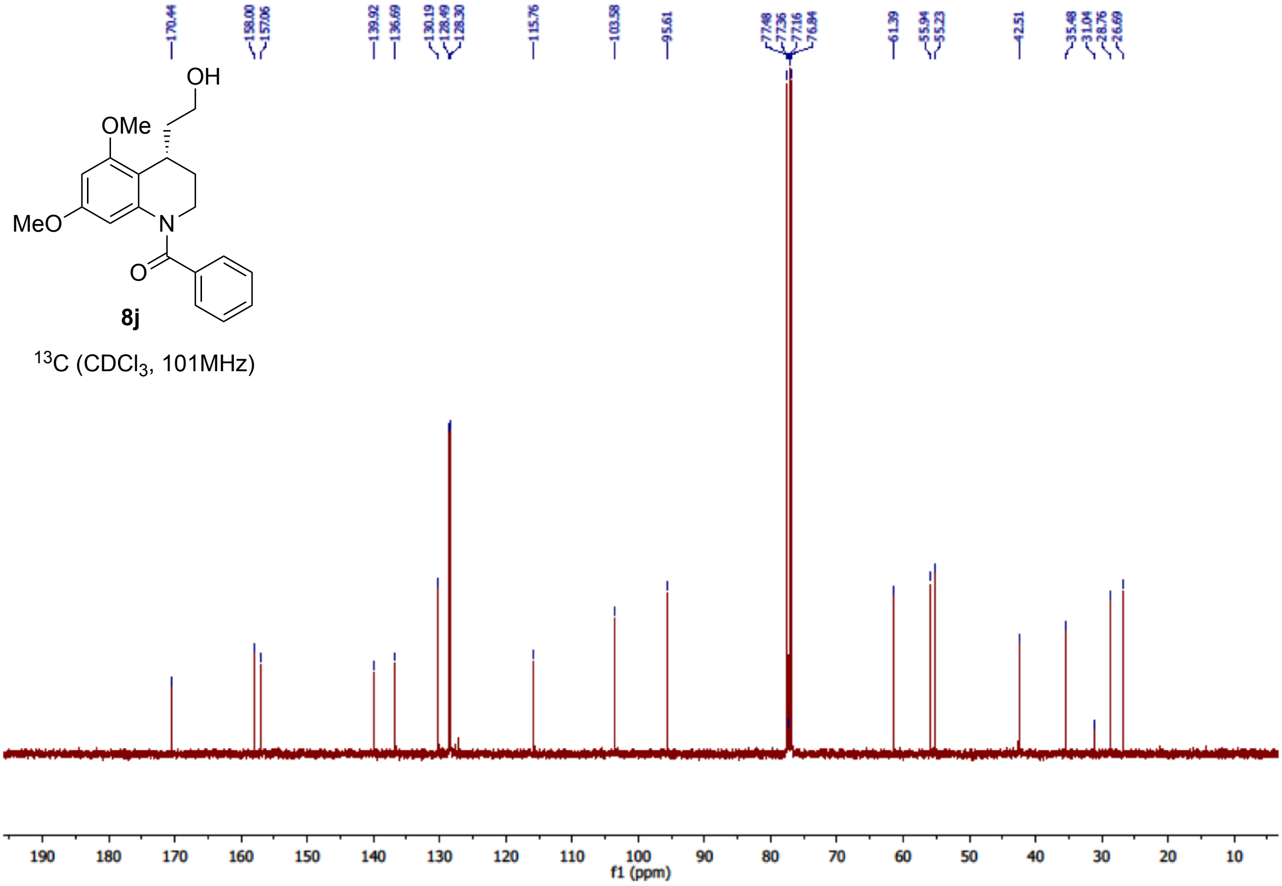

8j

^{13}C ($CDCl_3$, 101MHz)

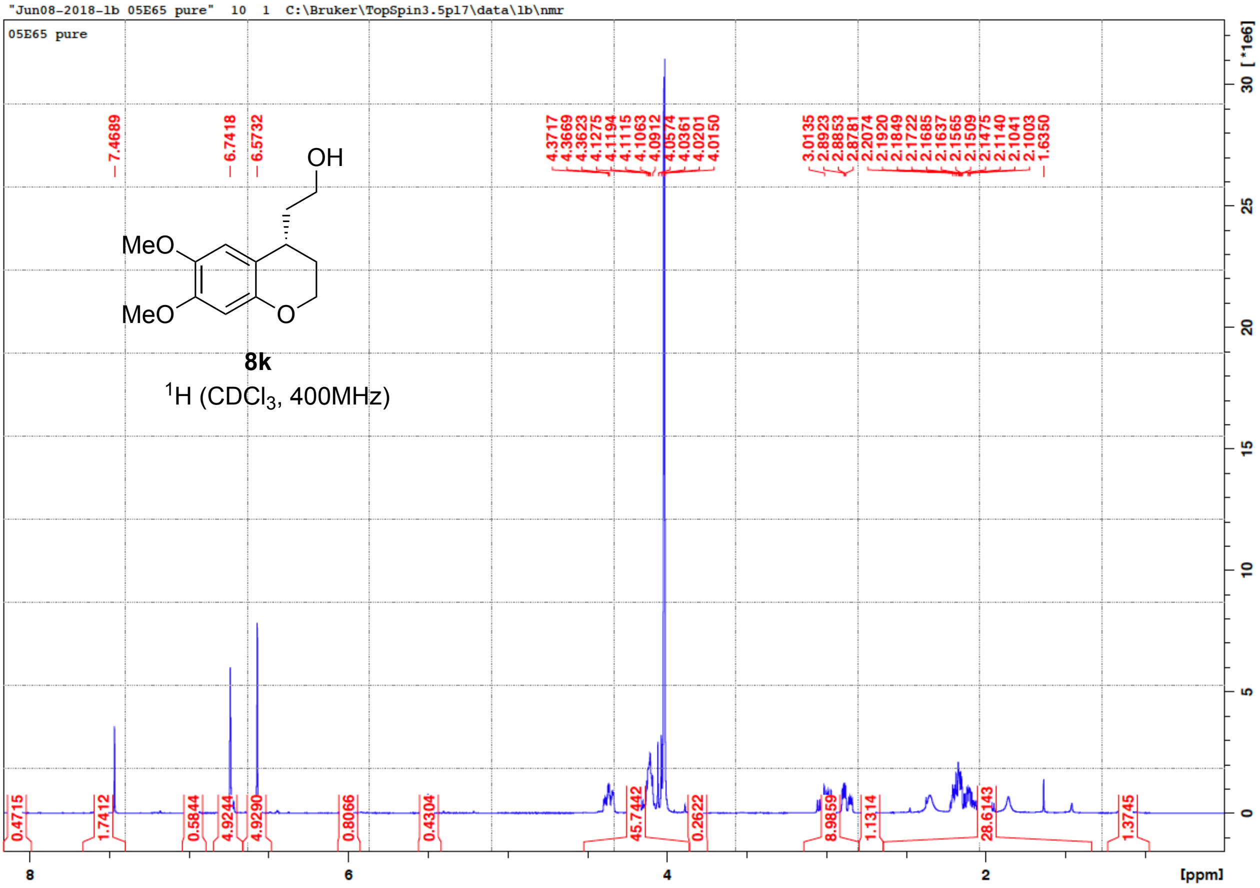
"Jun08-2018-lb 05E65 pure" 10 1 C:\Bruker\TopSpin3.5pl7\data\lb\nmr
05E65 pure
7.4689
6.7418
6.5732
4.3717
4.3669
4.3623
4.1275
4.1194
4.1115
4.1063
4.0912
4.0574
4.0361
4.0201
4.0150
3.0135
2.8923
2.8853
2.8781
2.2074
2.1920
2.1849
2.1722
2.1685
2.1637
2.1565
2.1509
2.1475
2.1140
2.1041
2.1003
1.6350
OH
MeO
MeO
O
8k
¹H (CDCl₃, 400MHz)
0.4715
1.7412
0.5844
4.9244
4.9290
0.8066
0.4304
45.7442
0.2622
8.9859
1.1314
28.6143
1.3745
[ppm]
[*1e6]

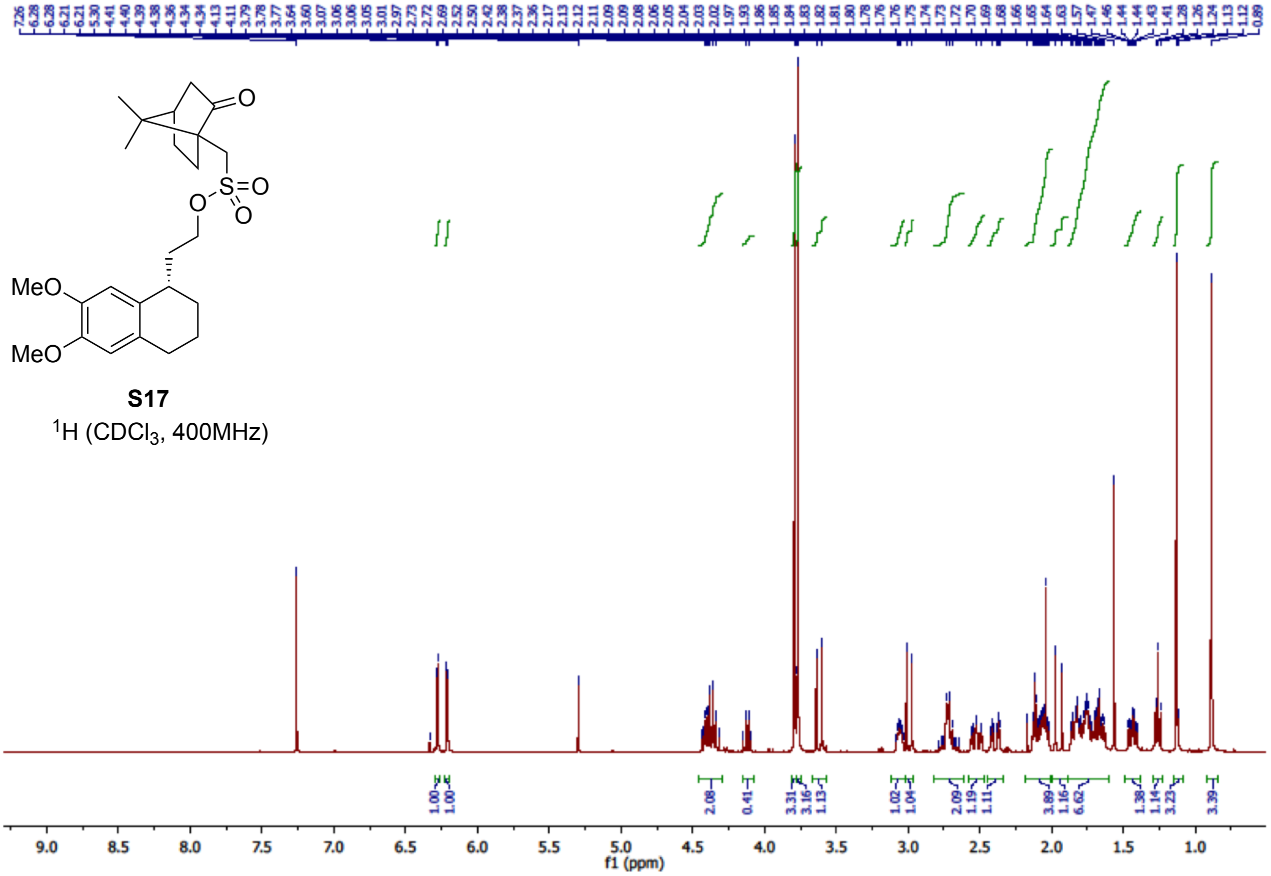
MeO
MeO
S17
1H (CDCl3, 400MHz)
f1 (ppm)

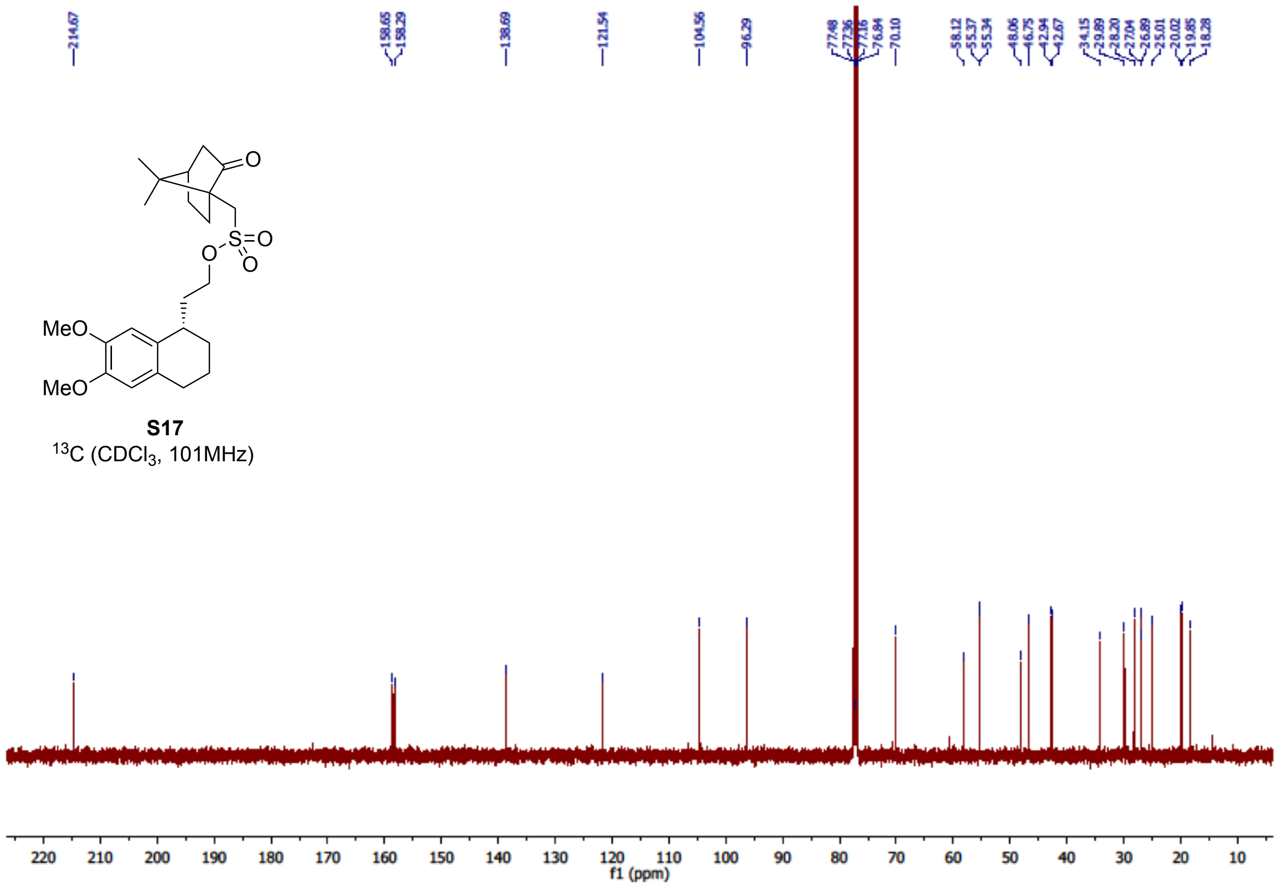
MeO
MeO
S17
13C (CDCl3, 101MHz)
f1 (ppm)

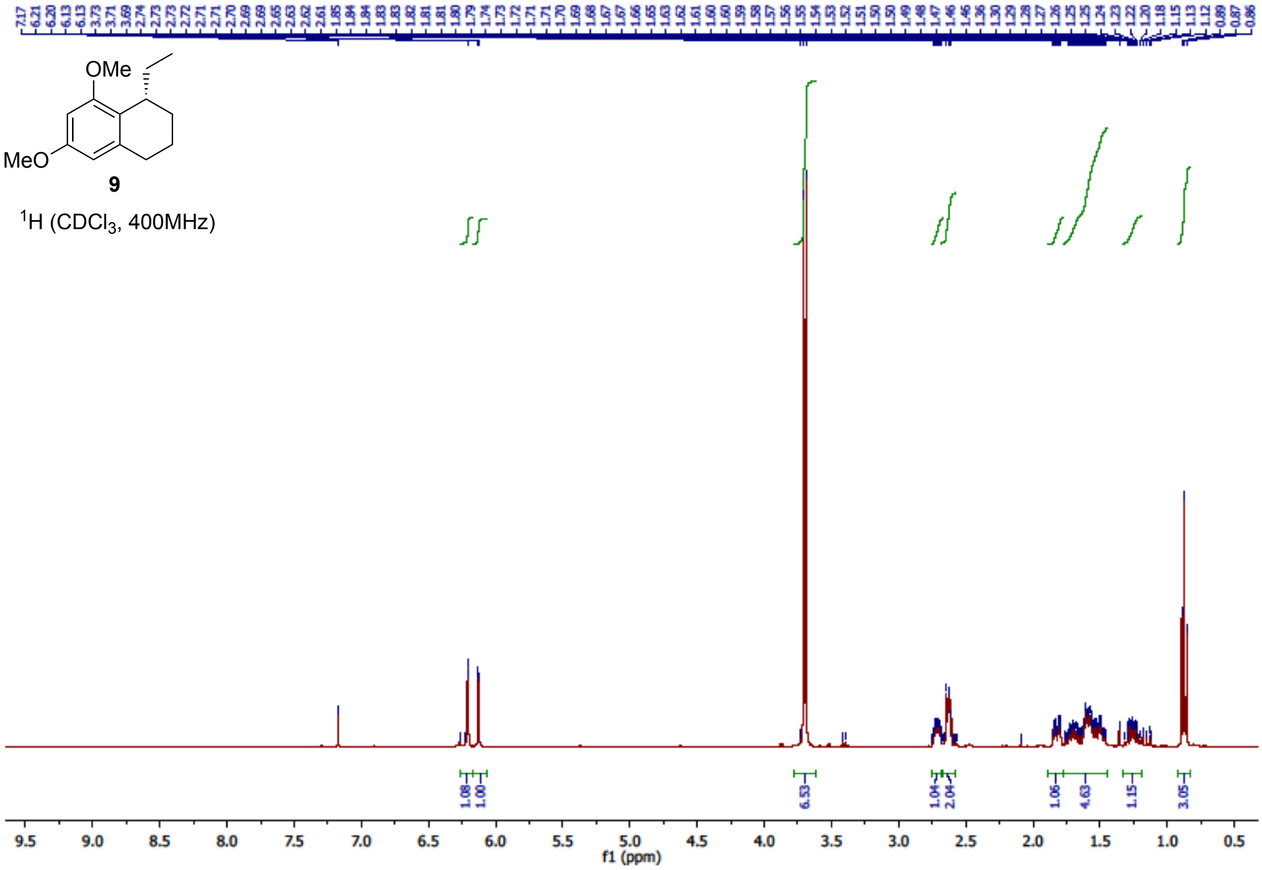
7.17 6.21 6.20 6.13 6.13 3.73 3.71 3.69 2.74 2.73 2.73 2.72 2.71 2.71 2.70 2.69 2.69 2.65 2.63 2.62 2.61 1.85 1.84 1.84 1.83 1.83 1.82 1.81 1.81 1.80 1.79 1.74 1.73 1.73 1.72 1.71 1.71 1.70 1.69 1.68 1.67 1.67 1.66 1.65 1.63 1.62 1.61 1.60 1.60 1.59 1.58 1.57 1.56 1.55 1.55 1.54 1.53 1.52 1.51 1.50 1.50 1.49 1.48 1.47 1.46 1.46 1.36 1.30 1.29 1.28 1.27 1.26 1.25 1.25 1.24 1.23 1.22 1.20 1.18 1.15 1.13 1.12 0.89 0.87 0.86
OMe
MeO
9
1H (CDCl3, 400MHz)
1.08 1.00 6.53 1.04 2.04 1.06 4.63 1.15 3.05
9.5 9.0 8.5 8.0 7.5 7.0 6.5 6.0 5.5 5.0 4.5 4.0 3.5 3.0 2.5 2.0 1.5 1.0 0.5
f1 (ppm)

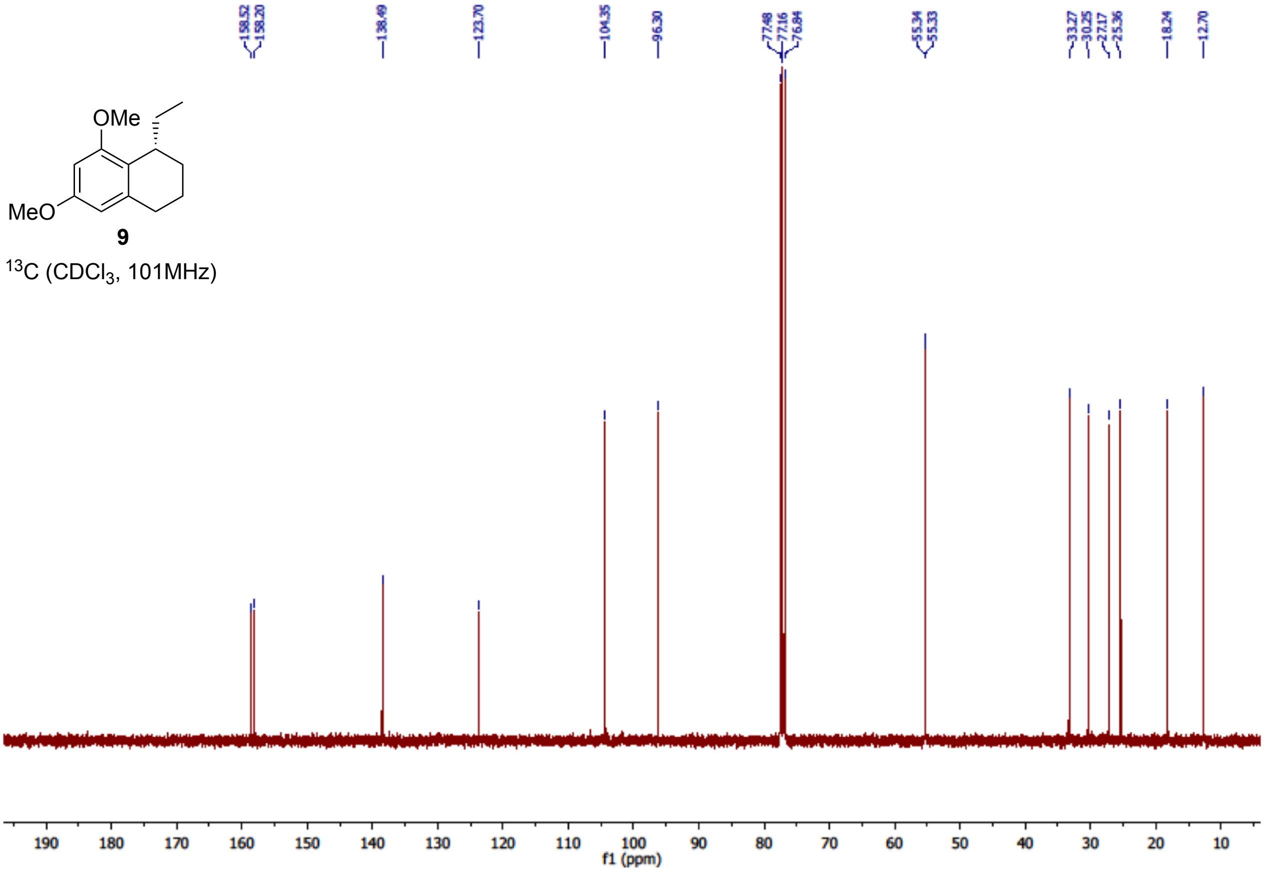
158.52 158.20 138.49 123.70 104.35 96.30 77.48 77.16 76.84 55.34 55.33 33.27 30.25 27.17 25.36 18.24 12.70
OMe
MeO
9
13C (CDCl3, 101MHz)
190 180 170 160 150 140 130 120 110 100 90 80 70 60 50 40 30 20 10
f1 (ppm)

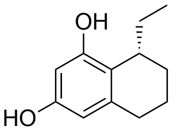

10

^{1}H ($CDCl_3$, 400MHz)

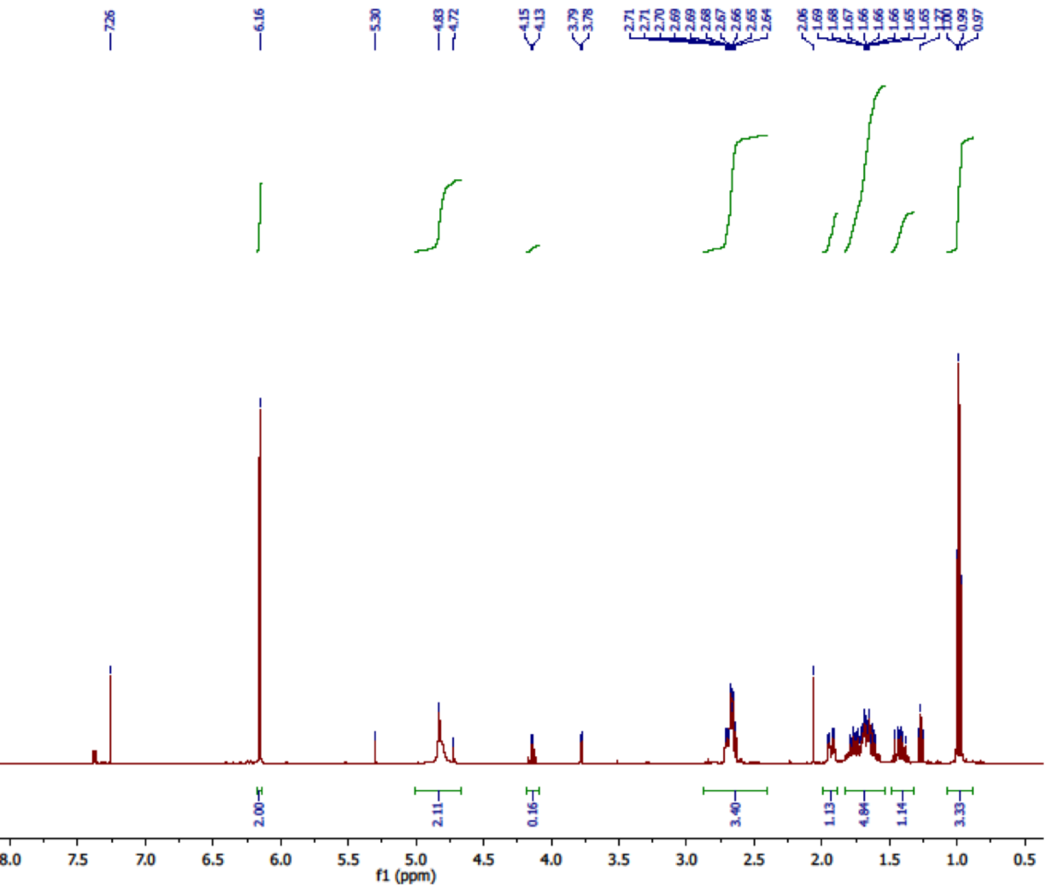

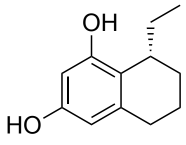

10

^{13}C ($CDCl_3$, 101MHz)

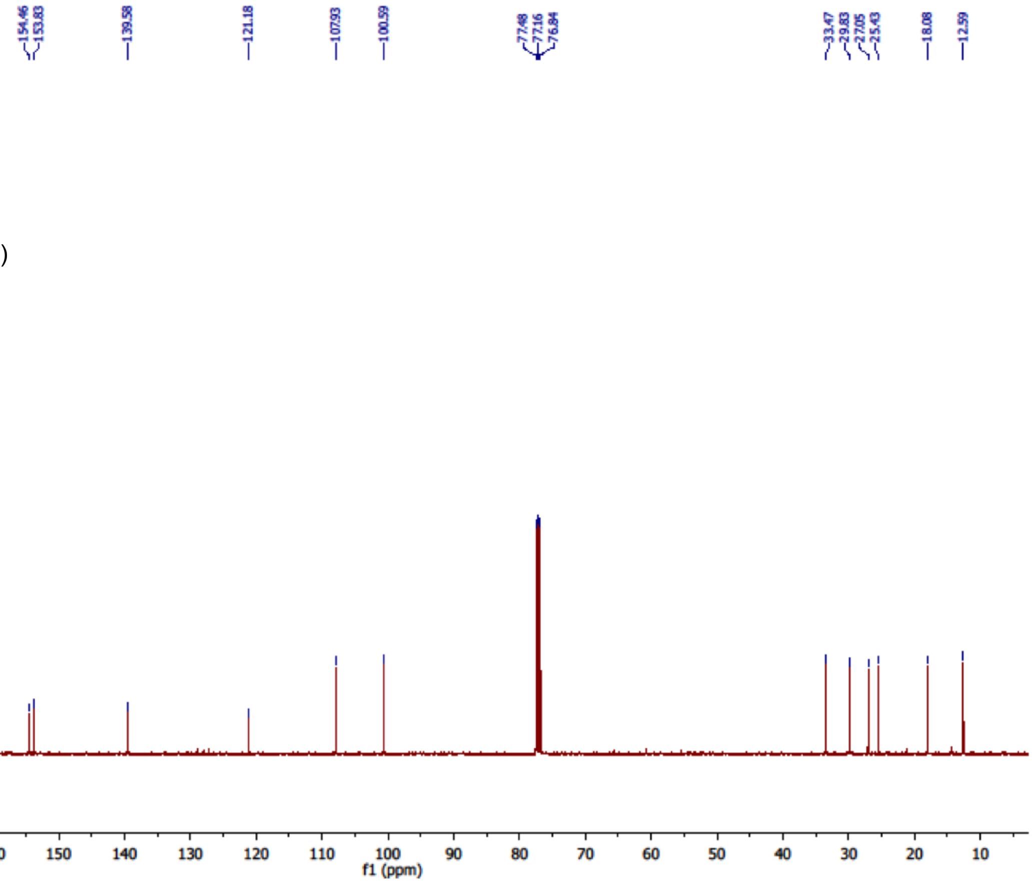

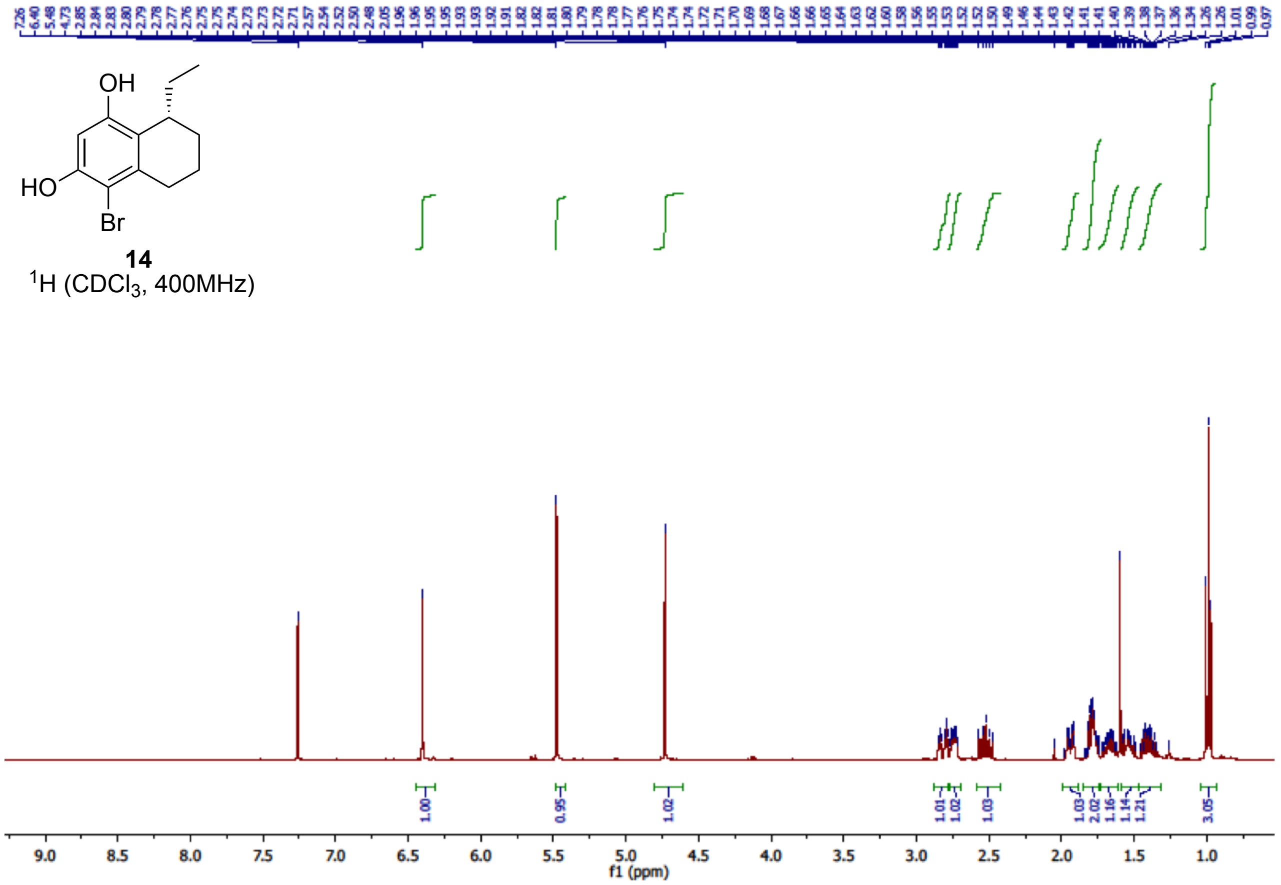
OH
HO
Br
14
1H (CDCl3, 400MHz)
1.00
0.95
1.02
1.01
1.02
1.03
1.03
2.02
1.16
1.14
1.21
3.05
f1 (ppm)

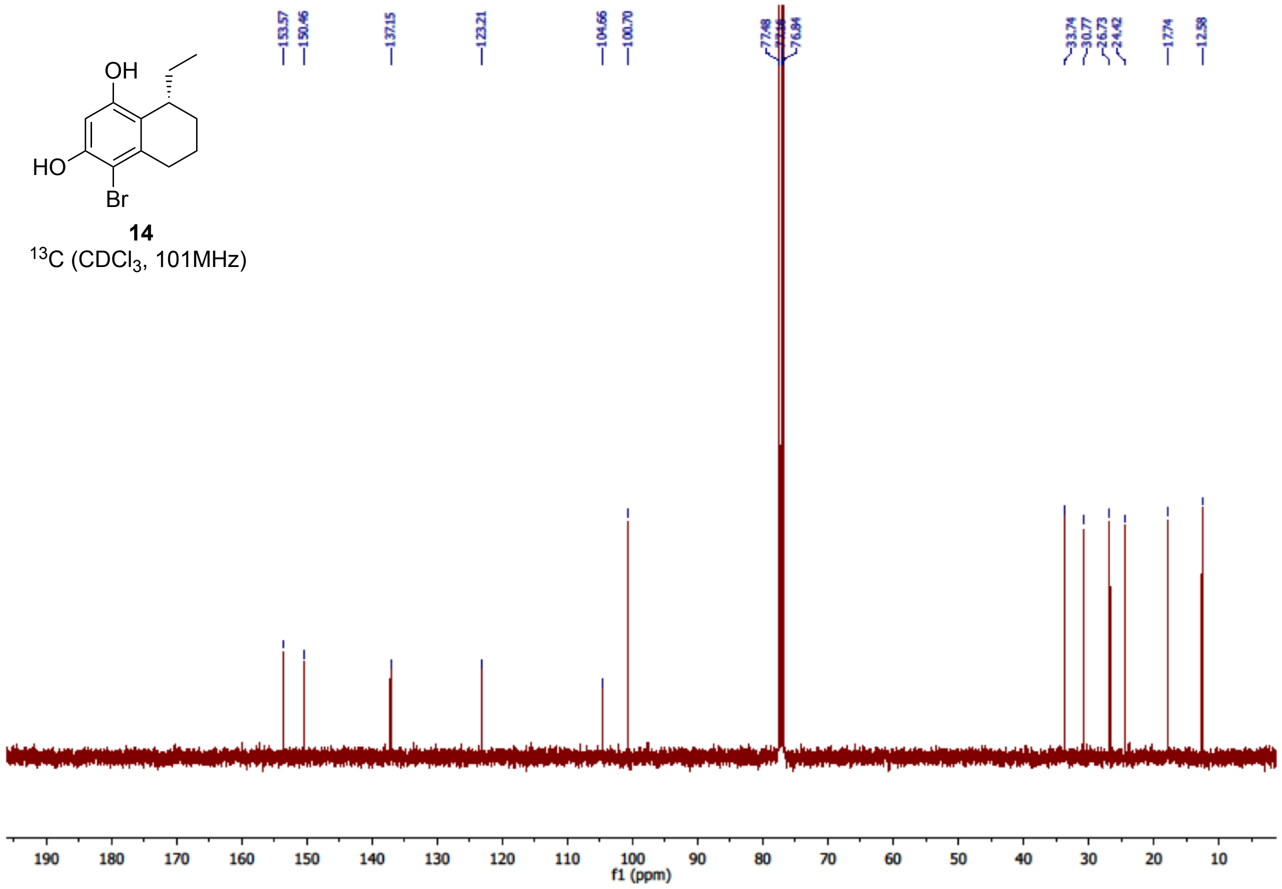
153.57
150.46
137.15
123.21
104.66
100.70
77.48
77.16
76.84
33.74
30.77
26.73
24.42
17.74
12.58
OH
HO
Br
14
13C (CDCl3, 101MHz)
f1 (ppm)

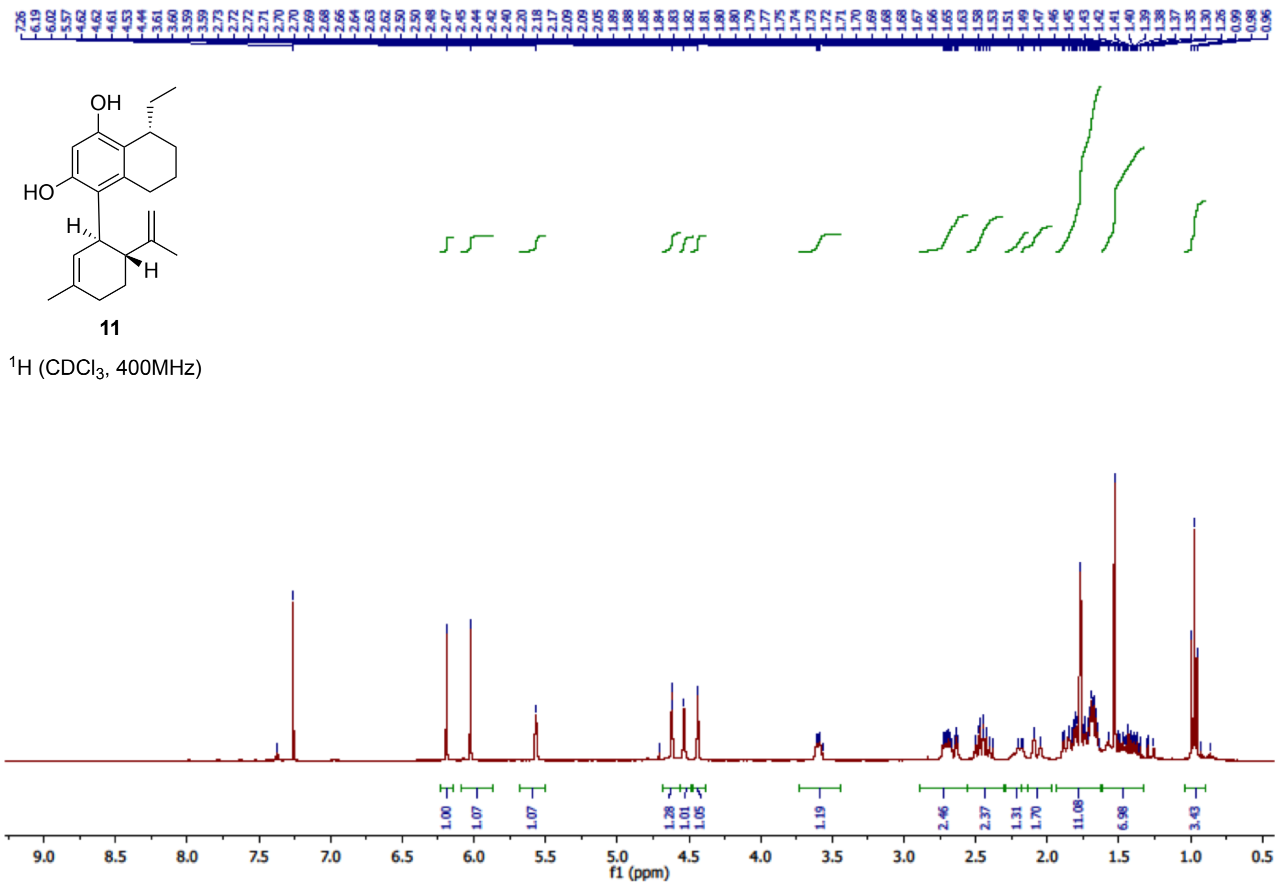

11

^{1}H (CDCl$_3$, 400MHz)

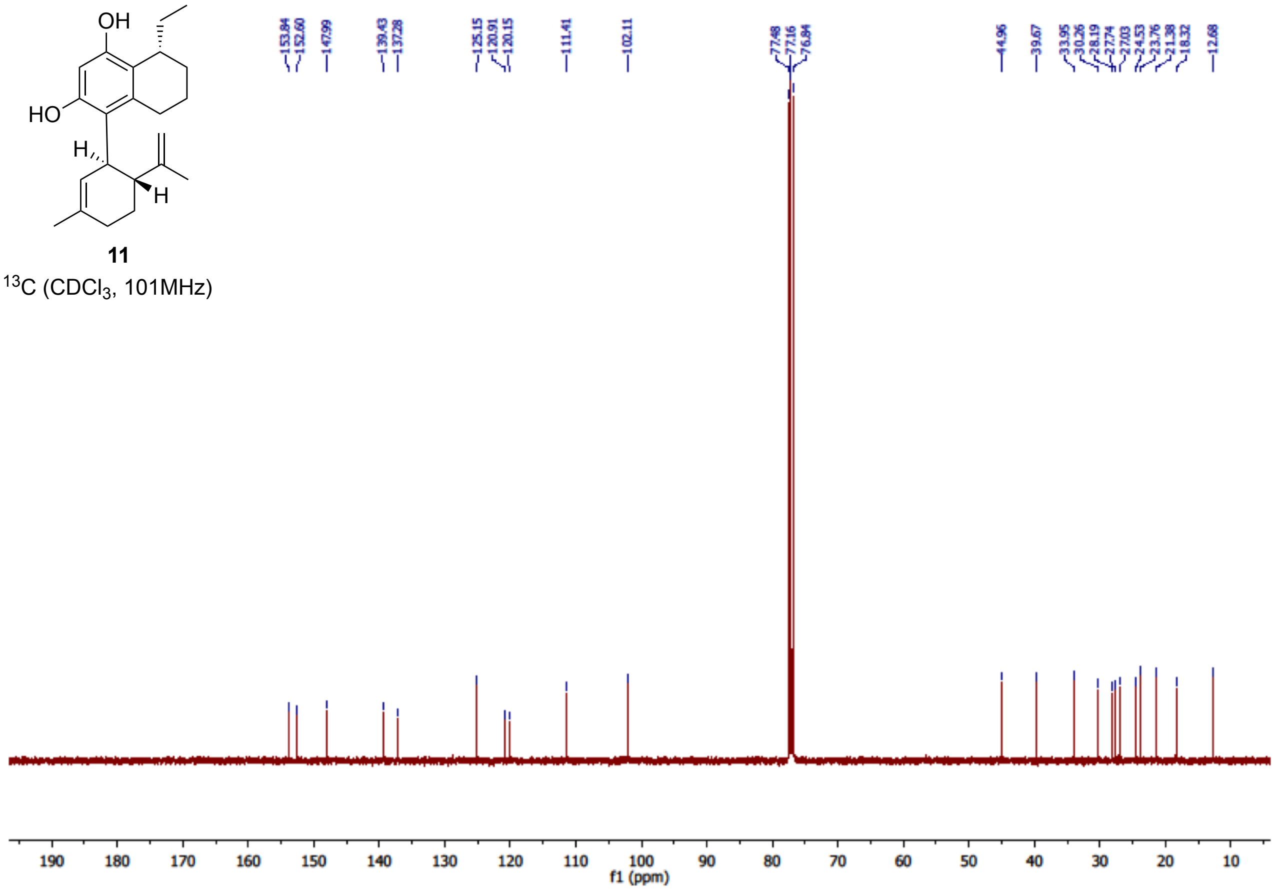

11

^{13}C (CDCl$_3$, 101MHz)

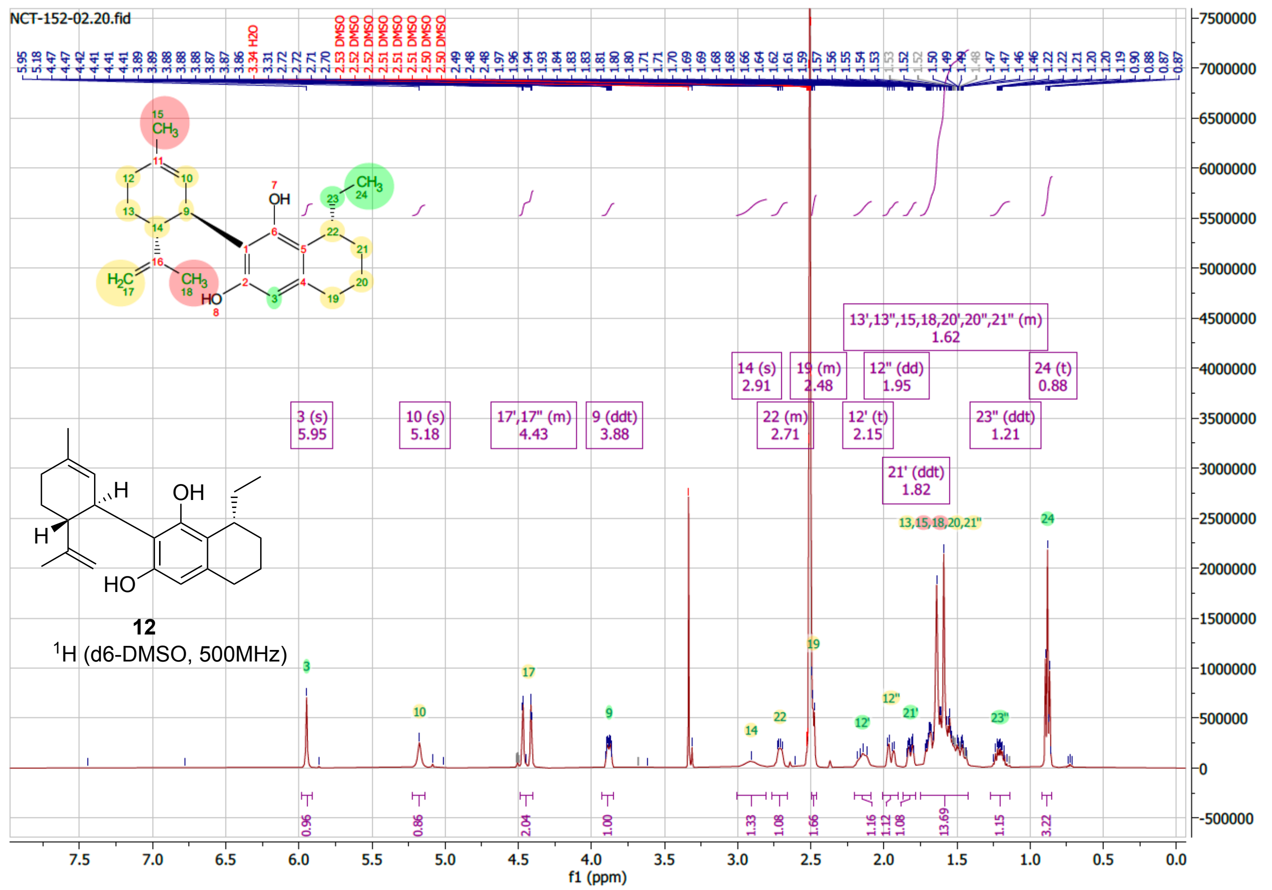
NCT-152-02.20.fid
12
1H (d6-DMSO, 500MHz)
3 (s) 5.95
10 (s) 5.18
17',17'' (m) 4.43
9 (ddt) 3.88
14 (s) 2.91
22 (m) 2.71
19 (m) 2.48
12' (t) 2.15
12'' (dd) 1.95
21' (ddt) 1.82
13',13'',15,18,20',20'',21'' (m) 1.62
23'' (ddt) 1.21
24 (t) 0.88
f1 (ppm)

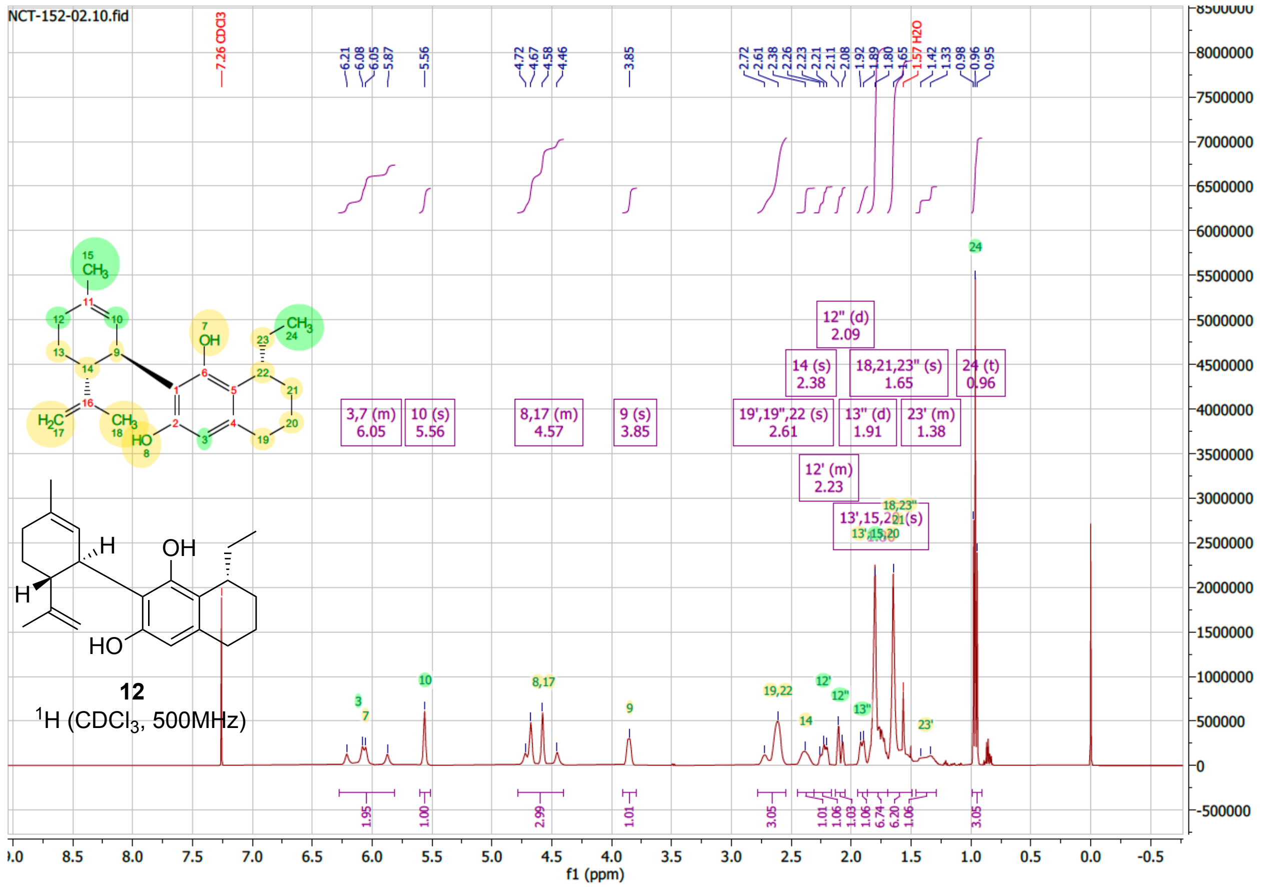
NCT-152-02.10.fid
12
1H (CDCl3, 500MHz)
3,7 (m) 6.05
10 (s) 5.56
8,17 (m) 4.57
9 (s) 3.85
19',19'',22 (s) 2.61
14 (s) 2.38
12' (m) 2.23
12'' (d) 2.09
13'' (d) 1.91
18,21,23'' (s) 1.65
23' (m) 1.38
24 (t) 0.96
f1 (ppm)

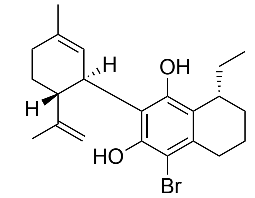

15

^{1}H (CDCl$_3$, 400MHz)

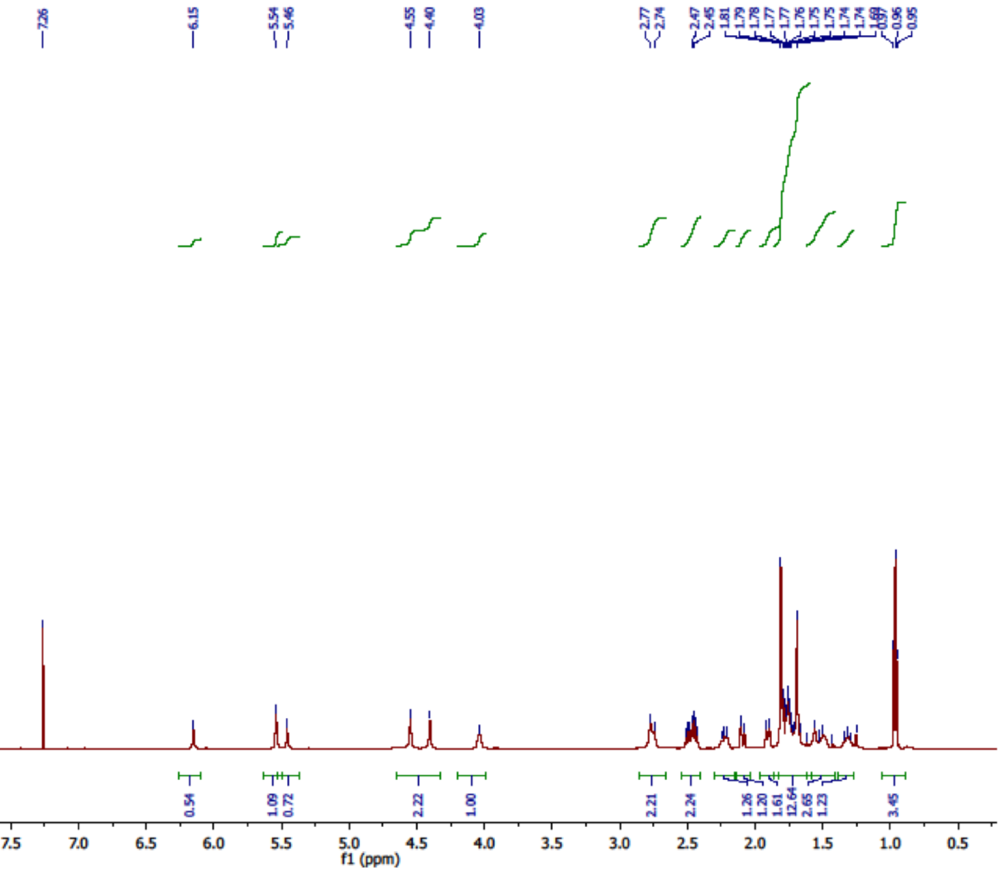

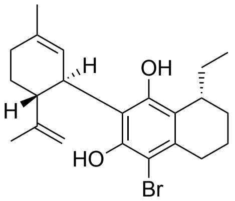

15

^{13}C (CDCl$_3$, 101MHz)

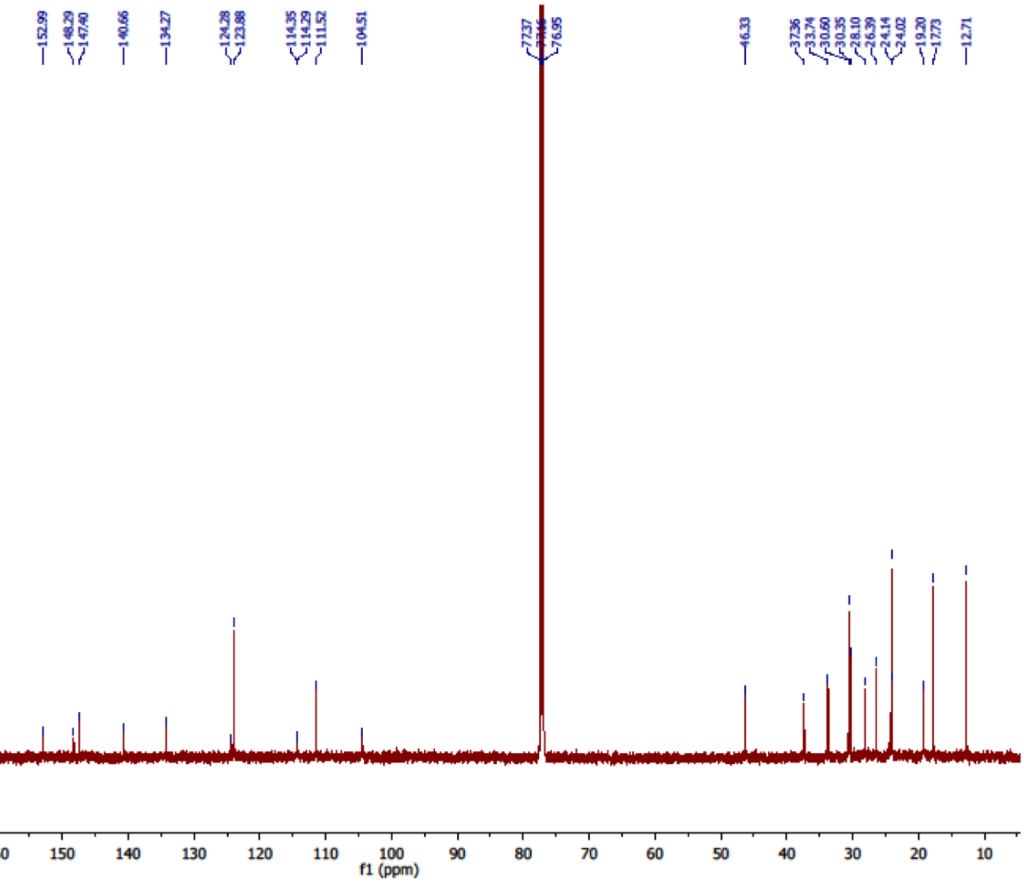

8. **XRD CheckCIF Data of Compound 17**

checkCIF/PLATON report

Structure factors have been supplied for datablock(s) LB0637

THIS REPORT IS FOR GUIDANCE ONLY. IF USED AS PART OF A REVIEW PROCEDURE FOR PUBLICATION, IT SHOULD NOT REPLACE THE EXPERTISE OF AN EXPERIENCED CRYSTALLOGRAPHIC REFEREE.

No syntax errors found. CIF dictionary Interpreting this report

Datablock: LB0637

```
Bond precision:   C-C = 0.0068 A          Wavelength=0.71073

Cell:             a=9.2254(3)    b=9.4586(3)         c=12.0829(4)
                  alpha=90       beta=110.572(4)     gamma=90
Temperature:      150 K

                 Calculated                 Reported
Volume           987.11(6)                  987.11(6)
Space group      P 21                       P 1 21 1
Hall group       P 2yb                      P 2yb
Moiety formula   C22 H29 Br O2              C22 H29 Br O2
Sum formula      C22 H29 Br O2              C22 H29 Br O2
Mr               405.35                     405.36
Dx,g cm-3        1.364                      1.364
Z                2                          2
Mu (mm-1)        2.094                      2.094
F000             424.0                      424.0
F000'            423.58
h,k,lmax         12,13,16                   12,12,16
Nref             5363[ 2835]                4558
Tmin,Tmax        0.951,0.979                0.881,1.000
Tmin'            0.901

Correction method= # Reported T Limits: Tmin=0.881 Tmax=1.000
AbsCorr = MULTI-SCAN

Data completeness= 1.61/0.85       Theta(max)= 29.245

R(reflections)= 0.0412( 4058)      wR2(reflections)= 0.0827( 4558)

S = 1.033                   Npar= 231
```

The following ALERTS were generated. Each ALERT has the format
test-name_ALERT_alert-type_alert-level.
Click on the hyperlinks for more details of the test.

Alert level B

PLAT420_ALERT_2_B D-H Bond Without Acceptor O1 --H1 . Please Check

Author Response: H atom orientation obtained from electron density, so we are confident that the orientation is correct

Alert level C

PLAT220_ALERT_2_C NonSolvent Resd 1 C Ueq(max)/Ueq(min) Range 3.2 Ratio
PLAT341_ALERT_3_C Low Bond Precision on C-C Bonds .... 0.00679 Ang.

Alert level G

PLAT007_ALERT_5_G Number of Unrefined Donor-H Atoms .... 2 Report
PLAT380_ALERT_4_G Incorrectly? Oriented X(sp2)-Methyl Moiety ..... C22 Check
PLAT791_ALERT_4_G Model has Chirality at C10 (Sohnke SpGr) R Verify
PLAT791_ALERT_4_G Model has Chirality at C13 (Sohnke SpGr) S Verify
PLAT791_ALERT_4_G Model has Chirality at C18 (Sohnke SpGr) R Verify
PLAT910_ALERT_3_G Missing # of FCF Reflection(s) Below Theta(Min). 4 Note
PLAT912_ALERT_4_G Missing # of FCF Reflections Above STh/L= 0.600 299 Note
PLAT941_ALERT_3_G Average HKL Measurement Multiplicity .... 4.5 Low
PLAT978_ALERT_2_G Number C-C Bonds with Positive Residual Density. 0 Info

0 **ALERT level A** = Most likely a serious problem - resolve or explain
1 **ALERT level B** = A potentially serious problem, consider carefully
2 **ALERT level C** = Check. Ensure it is not caused by an omission or oversight
9 **ALERT level G** = General information/check it is not something unexpected

0 ALERT type 1 CIF construction/syntax error, inconsistent or missing data
3 ALERT type 2 Indicator that the structure model may be wrong or deficient
3 ALERT type 3 Indicator that the structure quality may be low
5 ALERT type 4 Improvement, methodology, query or suggestion
1 ALERT type 5 Informative message, check

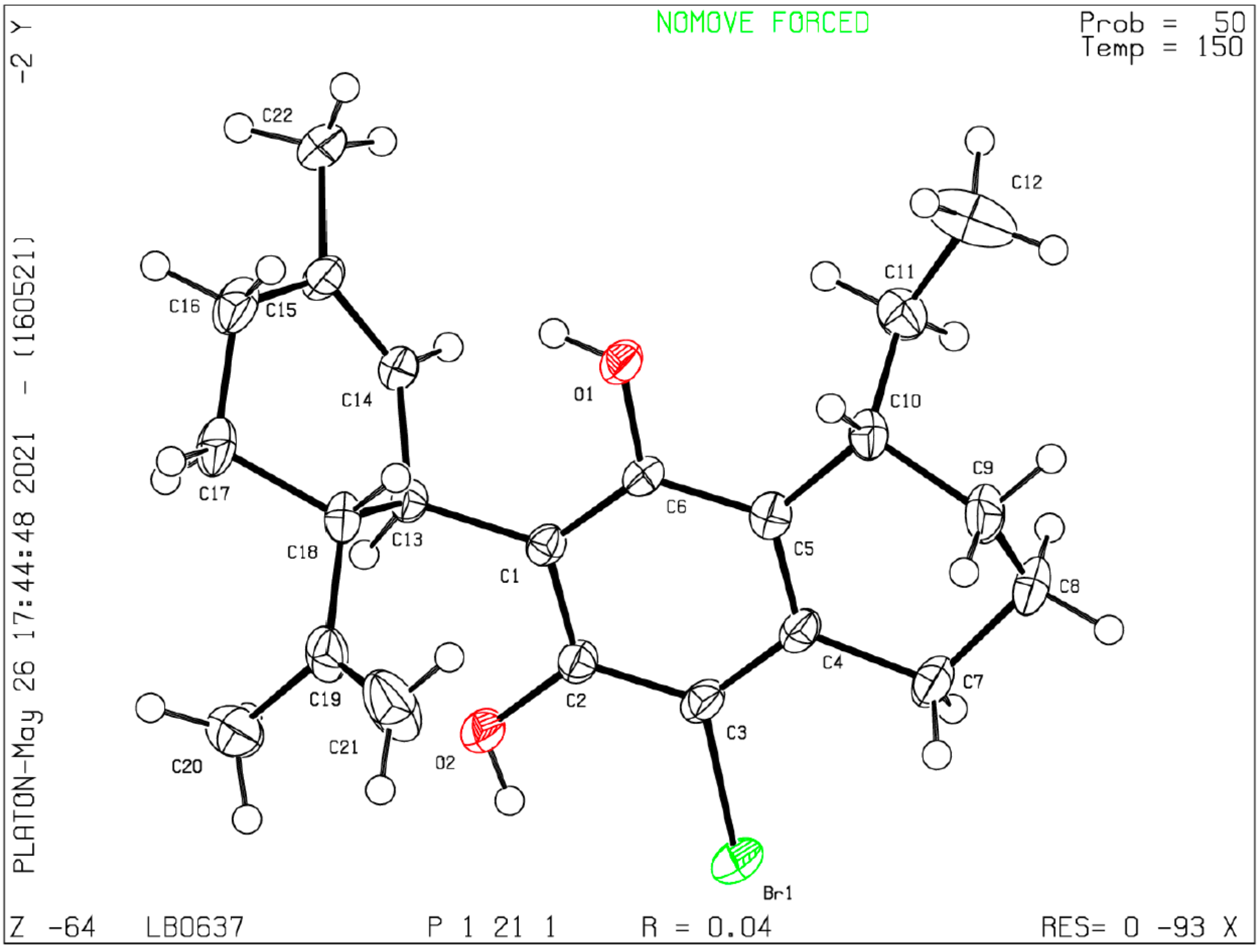

9. Unsuccessful Substrates

In the interests of balance we present below the substrates that did not cyclize, or gave minimal product.

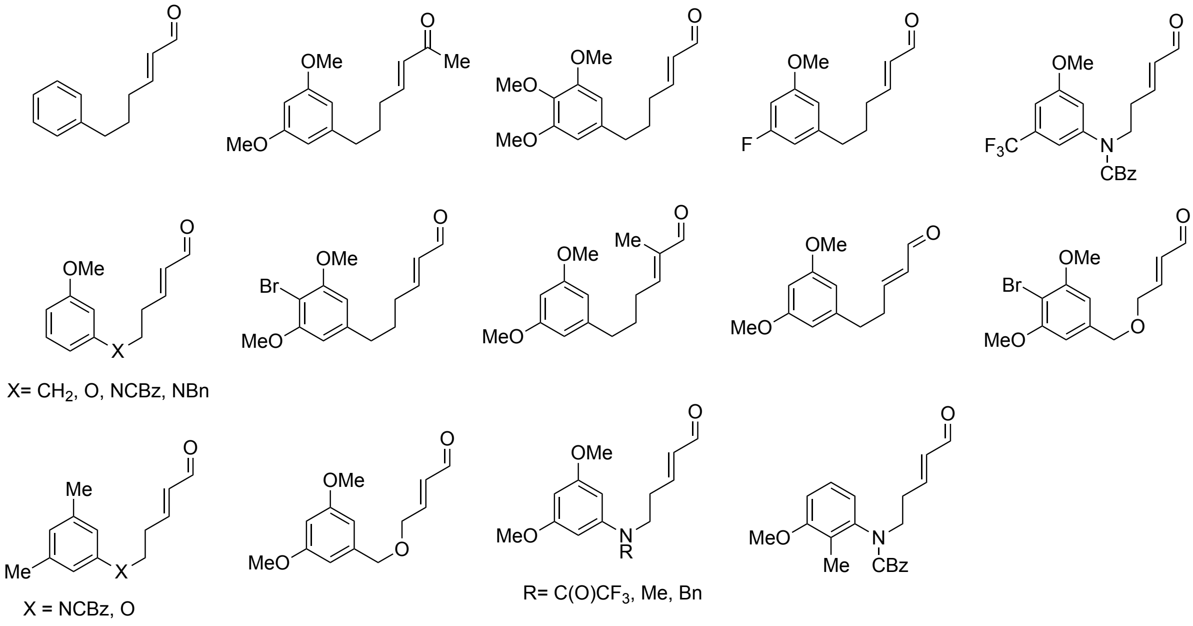

10. References

1. Lee, D.; Chang, S. *Chem. Eur. J.* **2015**, *21*, 5364
2. Mizukami, M.; Wada, K.; Sato, G.; Ishii, Y.; Kawahara, N.; Nagumo, S. *Tetrahedron*, **2013**, *69*, 4120
